# Application of chicken microarrays for gene expression analysis in other avian species

**DOI:** 10.1186/1471-2164-10-S2-S3

**Published:** 2009-07-14

**Authors:** Tamsyn M Crowley, Volker R Haring, Simon Burggraaf, Robert J Moore

**Affiliations:** 1 Australian Animal Health Laboratory, CSIRO, Geelong, Victoria, Australia; 2 Australian Poultry CRC, Armidale, New South Wales, Australia

## Abstract

**Background:**

With the threat of emerging infectious diseases such as avian influenza, whose natural hosts are thought to be a variety of wild water birds including duck, we are armed with very few genomic resources to investigate large scale immunological gene expression studies in avian species. Multiple options exist for conducting large gene expression studies in chickens and in this study we explore the feasibility of using one of these tools to investigate gene expression in other avian species.

**Results:**

In this study we utilised a whole genome long oligonucleotide chicken microarray to assess the utility of cross species hybridisation (CSH). We successfully hybridised a number of different avian species to this array, obtaining reliable signals. We were able to distinguish ducks that were infected with avian influenza from uninfected ducks using this microarray platform. In addition, we were able to detect known chicken immunological genes in all of the hybridised avian species.

**Conclusion:**

Cross species hybridisation using long oligonucleotide microarrays is a powerful tool to study the immune response in avian species with little available genomic information. The present study validated the use of the whole genome long oligonucleotide chicken microarray to investigate gene expression in a range of avian species.

## Background

Gene expression profiling utilising microarrays has become a widely used approach to elucidate biological function in host pathogen interactions. A variety of different platforms and approaches have been developed that have allowed analysis of gene expression in responses to viral infections [1-6] Prior to the sequencing of the chicken genome these microarrays consisted of cDNAs from many different sources. Sequencing of the chicken genome has increased the range of tools available to investigate gene expression including both short and long oligonucleotide arrays. These gene expression tools have far reaching benefits for the entire poultry industry, including the identification of genes that may play integral roles in determining disease resistance, productivity and quality.

However, identification of such important genes in other avian species is currently inhibited by a paucity of genomic resources. Cross-species gene-expression comparison is a powerful tool that may alleviate the lack of genomic information for a range of different avian species. By utilising existing microarrays it is possible to study gene expression in closely related species; this technique is termed cross species hybridisation (CSH). CSH has been previously used to investigate gene expression across a number of different species including humans and cattle [7], humans and primates [8], sheep and cattle [9] and a number of plant species [10]. CSH highlights the wider unconventional use of microarrays, proving that this technique can be flexible; however one must keep in mind the limits of this application, with regard to species specific genes.

In addition to studying gene expression profiles in closely related species, CSH can be used to explore comparative genomics. Comparative genomics provides an opportunity to ascertain relationships between gene function and location in a range of organisms [11]. Moreover, CSH allows insight into conservation of functional elements and the tracing of evolutionary phylogenies by way of comparing both closely and distantly related species. Comparative genomic studies in birds may help to develop detailed genomic information in a wide range of bird species. The significance of such genomic information is highlighted by the potential it offers in the study of important issues such as the recent outbreaks of avian influenza. Research on such diseases is hindered by the lack of genomic information available for many of the avian species known to be capable of infection by the virus. Alleviating this lack of detailed genomic information will inevitably assist in understanding the differences of immune responses in a range of avian species and allow better treatment and control strategies to be implemented.

We have utilised a whole genome chicken array to determine how useful it may be for the study of gene expression in other bird species. In particular we were interested to test a set of immunological gene probes on our chicken microarrays to see if they provide functionally useful results, thus revealing the utility of the arrays in cross-species studies. Here we test the utility of a whole chicken genome microarray to study immune response in other avian species by investigating spleen tissue expression patterns in a range of bird species and a comparison of spleen tissue from un-infected and H5N1 infected ducks. By understanding the strengths and limitations of cross species microarrays we will be able to elucidate the power of the arrays to address important biological issues in diverse bird species.

## Methods

### Tissue collection

**Figure 1:**
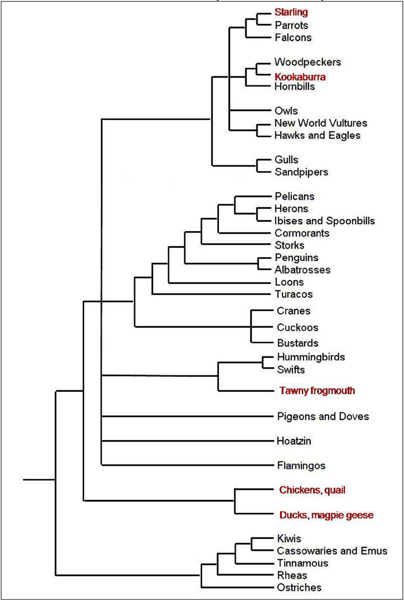
**Phylogenetic orders of birds**. Phylogenetic tree of all orders of birds displaying the relative evolutionary distance [20]. Red text indicates orders of birds that are represented in this study.

Spleen samples were collected from a wide range of avian species (Figure 1) (Chicken, *Gallus gallus*; Duck, *Anas platyrhynchos*; Starling, *Sturnus vulgaris*; Magpie goose, *Anseranas semipalmata*; Kookaburra, *Dacelo novaeguineae *and Tawny frogmouth, *Podargus strigoides*) and placed into 10 volumes of RNAlater (Ambion, USA) and stored at -20°C until RNA isolation. All control birds were at adult stage and free from clinical disease. Five-week-old Pekin ducks were challenged with a Vietnamese H5N1 strain (A/Muscovy duck/Vietnam/453/2004); each dose contained approximately 10^7.2 ^median egg infectious doses (EID_50_). Spleen samples were collected 2 days post infection. Infected samples were confirmed using viral titres (data not shown).

### Total RNA isolation and cDNA preparation

Total RNA for all samples studied was isolated using the Meridian total RNA isolation kit (Cartagen). Five micrograms of total RNA was reverse transcribed into cDNA and indirectly labelled with Cy3 using the ULS cDNA Synthesis and Labelling Kit (Kreatech Technologies). The labelled probes were concentrated using Microcon Ultracel YM-30 Columns (Amicon Bioseparations) and the quality and label incorporation of each sample was verified using the NanoDrop ND-1000 spectrophotometer (NanoDrop Technologies).

### Microarray design and hybridisation

Each Cy3 labelled spleen sample was individually hybridised to the whole genome chicken array (single colour hybridisation). This whole genome chicken microarray was printed with a MicroGrid II spotting robot in house using a set of 20,460 long oligos (65–75 nt) printed in duplicate on each array. The oligo set was designed at the Roslin Institute (Scotland, UK) based upon chicken Ensembl gene transcripts and other genomic information supplied by various research groups around the world http://www.ark-genomics.org/microarrays/bySpecies/chicken/.

Whole genome chicken arrays were pre-hybridised at 42°C for 45 min in pre-hybridisation buffer (25% (v/v) formamide, 5 × SSC, 0.1% (w/v) SDS, 10 mg/ml salmon testes DNA). Following addition of the labelled probe and hybridisation solution (25% (v/v) formamide, 5 × SSC, 0.1% (w/v) SDS, 25% (v/v) KREAblock), all arrays were incubated for 16 h at 42°C. Post hybridisation, all arrays were washed once in (2 × SSC, 0.1% (w/v) SDS) for 5 min at 42°C, once in (0.1 × SSC, 0.1% SDS) for 10 min at 25°C and three times in 0.1% SSC, each for 1 min at 25°C. For each avian species two independent spleen samples were hybridised to separate arrays to ensure reproducibility, except in the case of the H5N1 duck samples where three independent control spleen duck samples and three independent infected H5N1 spleen duck samples were hybridised.

### Microarray analysis

Post hybridisation all arrays were scanned and gene expression signals captured using an ArrayWORXe (Applied Precision) fluorescent scanner. All arrays were background corrected and arrays that had more than 0.0125% saturated spots were discarded. Each array was globally normalised; global normalisation was used as it ensures that the measured intensities are comparable across all slides. This sort of normalisation allows comparison of all arrays without biases when a majority of the spots on the arrays were giving positive hybridisation signals [12]. Subsequent statistical tests were carried out for the duck samples using GeneSpring 7.2 (Silicon Genetics) to determine all genes differentially regulated to a specified cut-off value (p = 0.05). A condition tree was constructed to explore the gene expression relationship between all duck samples. Condition trees were performed using standard correlation in GeneSpring 7.2. Spot net intensities were calculated from the median spot and background intensities with all results less than one standard deviation of the respective spot background pixel intensities ignored (set to zero). The net intensities of all arrays were then normalised such that the maximum spot intensity in each array was 10,000.

## Results

### Cross avian hybridisation of whole chicken genome arrays

**Figure 2:**
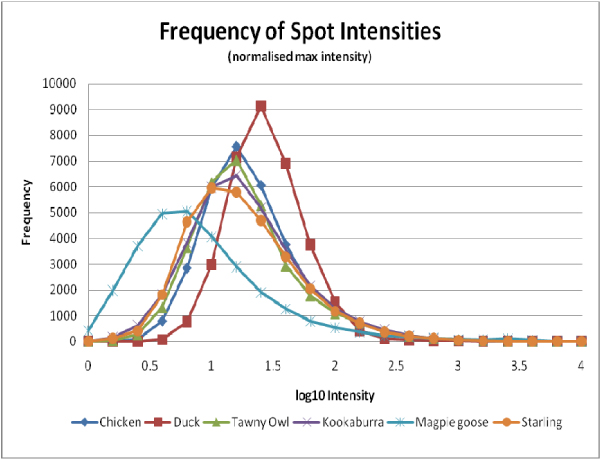
**Frequency of spot intensity**. Frequency and range of average values of net normalised signals for each avian species tested on the whole genome chicken array are shown. Each plot is the average of at least two independent hybridisations.

A range of avian samples spanning the Neognathae infraclass were used in this experiment (Figure 1). All of these avian samples were successfully hybridised to the whole genome chicken microarray and this is demonstrated in the summary statistics in Table 1. All arrays passed our quality control measures with the magpie geese samples displaying the least amount of spots higher than one standard deviation above background at 67%. As expected chicken spleen samples performed the best with 78% spots passing quality control (it is not expected that hybridising chicken to the whole chicken genome microarray will ever result in 100% spot hybridisation as not all genes in the genome will be expressed in any one sample). Background median intensities were similar for all arrays except for the duck arrays, where the values were higher. The duck spleen samples also displayed a larger net intensity and background standard deviation than all the other microarrays. The distributions of the log_10 _transformed normalised spot intensities for all control samples are plotted in Figure 2. These plots show similar trends between all samples except the magpie goose and duck arrays. The magpie goose plot shows a shift highlighting the higher number of low intensity spots on these arrays. The duck plot suggests that these samples have a greater number of high intensity spots when compared with chicken. Overall the avian microarrays displayed a significant range of spot intensities across the chicken microarray.

**Table 1:** Summary statistics for avian samples hybridised to the whole genome chicken microarray

Field	Chicken	Duck	Kookaburra	Tawny frogmouth	Magpie goose	Starling
**Maximum net intensity**	65,245	62,490	64,000	64,259	65,129	64,993
**Maximum normalised net intensity**	10,000	10,000	10,000	10,000	10,000	10,000
**Median net intensity**	98	136	90	56	72	55
**Median normalised net intensity**	15	22	14	9	11	8
Spots above 1 standard deviation* (max 43200)	33,759	32,483	32,659	31,154	28,748	31,390
Percentage of spots above 1 standard deviation*	78	75	76	72	67	73

*One standard deviation above the background intensity.

In order to further support the use of the whole chicken genome array for other avian species we specifically investigated the expression levels of a test set of chicken genes with known immunological functions. These results are shown in Table 2. Each of the avian species provided significant net signal intensity for a variety of the genes investigated. A number of the genes were more highly expressed in the other avian species when compared to chicken. The defensin gallinacin 1 gave significant net intensities for all birds studied, with particularly high values for both duck and tawny frogmouth. A number of the genes including IL-8, IFNγ, IFNλ and caspase 3 gave low or no net intensity signals across all samples. In summary, all avian samples tested here provided significant net signal intensities for a number of the chicken genes with known immunological functions.

**Table 2:** A selection of chicken immune genes present on the whole genome chicken microarray

		Net Intensity					
**Gene Name**	**oligo name**	**chicken**	**duck**	**magpie goose**	**starling**	**kookaburra**	**tawny frogmouth**

**Interleukins**							
IL-1β	RIGG20417	++	+++	++	+	++	+
IL-2	RIGG20032	+	++	+++	+	+	++
IL-6	RIGG20074	++	++	++	++	+	+++
IL-8	RIGG20034	x	++	x	++	x	x
IL-12α	RIGG20057	+++	+	++	+	++	++
IL-13	RIGG20059	+++	++	++	+	+++	++
**Interferons**							
IFNβ	RIGG20053	+++	+++	+++	+++	+++	+++
IFNγ	RIGG20054	+	++	x	+	+	x
IFNλ	RIGG20055	++	+	+	+	+	+
**Chemokines**							
K203	RIGG00459	+++	+	+++	+++	+++	+++
CX3C	RIGG00463	++	+++	++	++	+++	++
K60 (CXC chemokine)	RIGG00467	++	+++	+++	++	+	+
CXCL14	RIGG00466	+++	+++	+	++	+++	++
**Toll like receptors**							
TLR2	RIGG14974	++	++	++	++	+++	++
TLR15	RIGG14349	+++	+++	++	+++	+++	+++
TLR21	RIGG10152	++	+	+++	++	+	++
**Defensins**							
gallinacin 1	RIGG20043	+++	+++	++	+++	+++	+++
gallinacin 3	RIGG20044	+	+	+++	+	++	++
**MHC**							
MHC class I	RIGG12432	+++	+++	+++	+++	+++	+++
MHC class II	RIGG16832	++	+	++	+	+	++
**Caspases**							
caspase 1	RIGG02179	++	+++	+++	++	++	+
caspase 3	RIGG15820	x	++	+	+	x	x

Positive intensity signals are represented by '+' according to the following scale (+ = <50, ++ = 51–100 and +++ = >150. Crosses 'x' represent signals that were deemed unreliable as they did not pass quality control. All results are based on averages of at least two independent hybridisations containing dual representation of each gene.

### Differentiating between H5N1 infected and uninfected ducks

**Figure 3:**
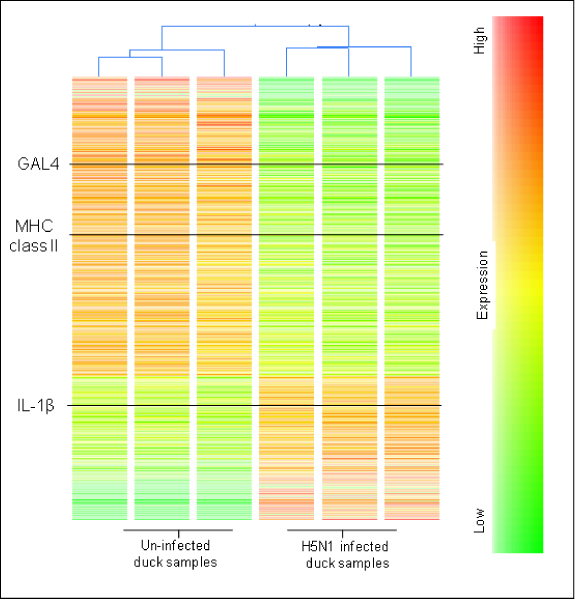
**Condition tree of H5N1 infected and uninfected ducks**. Average signal log intensities are presented, highlighting the strong relationship between the H5N1 infected samples compared to the uninfected duck controls. Immune genes of particular interest are shown in black.

A total of 2103 genes were differentially expressed (p = 0.05), 685 genes were up regulated in the H5N1 infected ducks and a further 244 genes were down regulated when compared to the uninfected controls (above 1.8 fold). Details of the specific genes regulated in this experiment can be found in the supplementary data (Additional file 1). Gene expression results are represented as a condition tree (Figure 3) and can be used to clearly differentiate the infected duck samples from the uninfected samples based on their gene expression profiles, which placed each group on separate arms of the tree. This condition tree also highlights the large number of regulated genes in the duck samples, and in particular identified key immune genes IL-1β, GAL4 and MHC class II that are regulated in the H5N1 infected samples.

## Discussion

Microarrays are a powerful tool for studying the gene expression of thousands of genes simultaneously. For species like the chicken where the whole genome has been sequenced it is possible to construct tailored whole genome arrays that allow comprehensive gene expression studies. For other avian species, such as duck, where there is little available genomic information, it is not yet possible to design specific microarrays. In this study, we explored the technical feasibility of utilising cross species hybridisations to identify genes expressed in a broad range of avian species. Our experimental strategy was three-fold. The first goal was to test the hybridisation of a number of phylogenetically diverse avian species on the whole genome chicken microarray. Our second goal was to determine if this microarray platform was able to distinguish between two different biological states in a bird species other than chicken. Thirdly, we were interested in testing the feasibility of the whole genome chicken microarray in avian immunological studies by focusing on the performance of known chicken immune genes.

As documented previously [10], long oligonucleotide microarrays are a reliable tool for CSH as they identify significantly more regulated genes at a lower false discovery rate than microarrays with short oligomers. Long oligonucleotide arrays are also considered to be an improvement over cDNA arrays as they eradicate the problem of clone misidentification and erroneous cross hybridisation [13]. The use of the long oligonucleotide whole genome chicken array as a tool in comparative genomics for other avian species has been validated in this study. We have successfully hybridised a broad range of phylogenetically distinct bird species to this microarray. Each of the avian species had a large number of spots that passed our quality control measures to the extent that there was a low degree of spot drop out (at most 11%) compared to the chicken.

Overall most of the avian samples showed a similar performance to chicken when hybridised to the chicken microarray; however there were some slight shifts in some of the expression profiles. The magpie goose arrays displayed a higher number of spots with lower signals compared with chicken and had the fewest number of genes that passed our quality control measures. This may be explained by a lack of genetic similarity between chicken and magpie goose; however with little sequence information for magpie goose we must be cautious when interpreting our results. These results highlight the potential use of this whole genome chicken long oligonucleotide microarray for gene expression studies in a wide range of avian species.

When performing CSH the degree of sequence complimentarily between probes and targets is variable. Differences in hybridisation levels can be attributed to transcript abundance levels, the presence of sequence mismatches and differences in hybridisation kinetics [13,14]. Due to these limitations, it is not possible to compare gene expression levels for particular genes across species, but comparisons within a species is valid because of the same level of sequence identity and hybridisation kinetics will apply. Thus, we present the experiment utilising the H5N1 infected ducks as a test of the validity of using a chicken microarray to distinguish between two different immune states in ducks. This serves as a tool to carry out more in depth analysis of H5N1 infection in ducks. By contrasting such results with findings in the chicken it may be possible to determine the basis for the very different outcomes of viral infection in each species [15].

The whole genome chicken microarrays were successful in differentiating the infected ducks and control ducks from one another based on their overall gene expression profiles. In addition, the chicken microarrays were able to identify a number of key immune genes that were regulated in the H5N1 infected ducks. These genes include IL-1β, MHC class II alpha chain and the defensin gene GAL4. These genes have been previously shown to be involved in a chicken's response to infection [16] and thus it is plausible that these genes play a similar role in ducks. Interestingly the sequence similarity of IL-1β in ducks and chicken is 99% and it has recently been shown to be structurally and functionally homologous [17-19]. Moreover, the use of chicken microarrays to investigate gene expression in other birds may provide a new way of identifying conserved genes with known immunological functions in other avian species. In particular, CSH may be used to investigate H5N1 infection in a range of other bird species including swans, crows, turkeys, quail and geese. Cross species hybridisation is a feasible approach because of the relatively close evolutionary distance between avian species. While CSH has great potential for use in a wide range of avian species, it must also be noted that this method of studying gene expression will neglect to capture species specific genes that are involved in the host response.

## Conclusion

This is the first study to show the benefits of CSH for avian species using a whole genome chicken long oligonucleotide microarray. We have successfully demonstrated hybridisation of a number of phylogenetically diverse bird species to this chicken array. In so doing we have been able to identify a range of immune genes that can be detected in the avian species studied. We have also demonstrated the use of this chicken array as a tool to investigate gene expression in closely related immune genes. This study provides avian immunologists with a new tool to investigate gene expression in avian species with little or no genome information.

## Competing interests

The authors declare that they have no competing interests.

## Authors' contributions

TMC performed the RNA extractions, microarray hybridizations and analyses and drafted the manuscript. VRH was instrumental in the cross species microarray analysis. SB helped with the total RNA isolation and collected the H5N1 infected duck samples. RJM is the principal investigator of the laboratory and was involved in the conceptualisation of the manuscript. All authors read and approved the final manuscript.

## Supplementary Material

Additional file 1 **All genes regulated in the duck H5N1 experiment**. all genes that were regulated greater than 1.8 fold (p = 0.05 and p = 0.01) between uninfected control duck samples and duck samples infected with H5N1. Click here for file

## References

[B1] Dar A Munir S Vishwanathan S Manuja A Griebel P Tikoo S Townsend H Potter A Kapur V Babiuk LA Transcriptional analysis of avian embryonic tissues following infection with avian infectious bronchitis virus Virus Research 2005 110 41 55 10.1016/j.virusres.2005.01.006 15845254 PMC7114260

[B2] Neiman PE Burnside J Elsaesser K Hwang H Clurman BE Kimmel R Delrow J Analysis of gene expression, copy number and palindrome formation with a Dt40 enriched cDNA microarray Sub-Cellular Biochemistry 2006 40 245 56 17623909 10.1007/978-1-4020-4896-8_14

[B3] Ruby T Whittaker C Withers DR Chelbi-Alix MK Morin V Oudin A Young JR Zoorob R Transcriptional profiling reveals a possible role for the timing of the inflammatory response in determining susceptibility to a viral infection J Virol 2006 80 9207 16 16940532 10.1128/JVI.00929-06 PMC1563900

[B4] Sarmento L Afonso CL Estevez C Wasilenko J Pantin-Jackwood M Differential host gene expression in cells infected with highly pathogenic H5N1 avian influenza viruses Veterinary Immunology and Immunopathology 2008 10.1016/j.vetimm.2008.05.021 18617273

[B5] Sarson AJ Parvizi P Lepp D Quinton M Sharif S Transcriptional analysis of host responses to Marek's disease virus infection in genetically resistant and susceptible chickens Animal Genetics 2008 39 232 40 10.1111/j.1365-2052.2008.01710.x 18371127

[B6] Sarson AJ Abdul-Careem MF Zhou H Sharif S Transcriptional analysis of host responses to Marek's disease viral infection Viral Immunology 2006 19 747 58 10.1089/vim.2006.19.747 17201670

[B7] Adjaye J Herwig R Herrmann D Wruck W Benkahla A Brink TC Nowak M Carnwath JW Hultschig C Niemann H Lehrach H Cross-species hybridisation of human and bovine orthologous genes on high density cDNA microarrays BMC Genomics 2004 5 83 15511299 10.1186/1471-2164-5-83 PMC535340

[B8] Wallace JC Korth MJ Paeper B Proll SC Thomas MJ Magness CL Iadonato SP Nelson C Katze MG High-density rhesus macaque oligonucleotide microarray design using early-stage rhesus genome sequence information and human genome annotations BMC Genomics 2007 8 28 17244361 10.1186/1471-2164-8-28 PMC1790710

[B9] Kijas JW Menzies M Ingham A Sequence diversity and rates of molecular evolution between sheep and cattle genes Animal Genetics 2006 37 171 4 10.1111/j.1365-2052.2005.01399.x 16573533

[B10] Bar-Or C Bar-Eyal M Gal TZ Kapulnik Y Czosnek H Koltai H Derivation of species-specific hybridization-like knowledge out of cross-species hybridization results BMC Genomics 2006 7 110 16677401 10.1186/1471-2164-7-110 PMC1482311

[B11] Griffin DK Robertson LB Tempest HG Vignal A Fillon V Crooijmans RP Groenen MA Deryusheva S Gaginskaya E Carre W Waddington D Talbot R Volker M Masabanda JS Burt DW Whole genome comparative studies between chicken and turkey and their implications for avian genome evolution BMC Genomics 2008 9 168 18410676 10.1186/1471-2164-9-168 PMC2375447

[B12] Xiong H Zhang D Martyniuk CJ Trudeau VL Xia X Using generalized procrustes analysis (GPA) for normalization of cDNA microarray data BMC Bioinformatics 2008 9 25 18199333 10.1186/1471-2105-9-25 PMC2275243

[B13] Tsai S Mir B Martin AC Estrada JL Bischoff SR Hsieh WP Cassady JP Freking BA Nonneman DJ Rohrer GA Piedrahita JA Detection of transcriptional difference of porcine imprinted genes using different microarray platforms BMC Genomics 2006 7 328 17194308 10.1186/1471-2164-7-328 PMC1769376

[B14] Vallee M Robert C Methot S Palin MF Sirard MA Cross-species hybridizations on a multi-species cDNA microarray to identify evolutionarily conserved genes expressed in oocytes BMC Genomics 2006 7 113 16686947 10.1186/1471-2164-7-113 PMC1475851

[B15] MacDonald MR Veniamin SM Guo X Xia J Moon DA Magor KE Genomics of antiviral defenses in the duck, a natural host of influenza and hepatitis B viruses Cytogenet Genome Res 2007 117 195 206 10.1159/000103180 17675860

[B16] Wu YF Liu HJ Shien JH Chiou SH Lee LH Characterization of interleukin-1beta mRNA expression in chicken macrophages in response to avian reovirus Journal of General Virology 2008 89 1059 68 10.1099/vir.0.82957-0 18343850

[B17] Lamont SJ Immunogenetics and the major histocompatibility complex Veterinary Immunology and Immunopathology 1991 30 121 7 10.1016/0165-2427(91)90013-3 1781152

[B18] Hasenstein JR Zhang G Lamont SJ Analyses of Five gallinacin genes and the Salmonella enterica serovar Enteritidis response in poultry Infection and Immunity 2006 74 3375 80 10.1128/IAI.00027-06 PMC1479296 16714567

[B19] Wu YF Liu HJ Chiou SH Lee LH Sequence and phylogenetic analysis of interleukin (IL)-1beta-encoding genes of five avian species and structural and functional homology among these IL-1beta proteins Veterinary Immunology and Immunopathology 2007 116 37 46 10.1016/j.vetimm.2006.12.010 17275099

[B20] Hackett SJ Kimball RT Reddy S Bowie RC Braun EL Braun MJ Chojnowski JL Cox WA Han KL Harshman J Huddleston CJ Marks BD Miglia KJ Moore WS Sheldon FH Steadman DW Witt CC Yuri T A phylogenomic study of birds reveals their evolutionary history Science 2008 320 1763 8 10.1126/science.1157704 18583609

