# Supplementary material for: Application of chicken microarrays for gene expression analysis in other avian species
Source: BMC Genomics. 2009 Jul 14;10(Suppl 2):S3. doi: 10.1186/1471-2164-10-S2-S3 (PMC2713436; doi:10.1186/1471-2164-10-S2-S3)
Supplement: Additional file 1 — All genes regulated in the duck H5N1 experiment. all genes that were regulated greater than 1.8 fold (p = 0.05 and p = 0.01) between uninfected control duck samples and duck samples infected with H5N1. [file 1471-2164-10-S2-S3-S1.pdf]

Additional file 1 - All genes regulated in the duck H5N1 experiment

Gene Name	Fold Change	p-value	SEQUENCE	Array Description	Blast/Database Description
RIGG00153	410.6	0.00336	ACTTATCAGCTGGAGAGACTATATGCCAGGTCTGCTTTCAGATATTCCCAAATAATGAGCAACTGGAGCAG	Gallus gallus mRNA for hypothetical protein, clone 8o12	Gallus gallus mRNA for hypothetical protein, clone 8o12 / similar to GB|AAH50719.1|29792103|BC050719 ZBTB1 protein {Homo sapiens} (exp=-1; wgp=0; cg=0), partial (91%) / Gallus gallus mRNA for hypothetical protein, clone 8o12 / Hypothetical protein, clone 8o12 / RCJMB04_8o12: Hypothetical protein / PREDICTED: Gallus gallus similar to ZBTB1 protein (LOC428916), mRNA. / PREDICTED: similar to ZBTB1 protein; / Hypothetical protein, clone 8o12; / --- / Gallus gallus mRNA for hypothetical protein, clone 8o12 / Hypothetical protein, clone 8o12
RIGG10525	364	0.00826	GTCGTGCTGGATCCACCGCAATGAGTACTACGAGGTAGGGCCGTGCATTGTCCACCAGAAGTGCT		ENSGALT00000002192.1 / similar to UP|Q3M0W9_PARTE (Q3M0W9) Actin 1-9, partial (7%) / testis_EST00244 Testis cDNA Library Gallus gallus cDNA 3', mRNA sequence / Transcribed locus, weakly similar to NP_116614.1 Actin, structural protein involved in cell polarization, endocytosis, and other cytoskeletal functions; Act1p [Saccharomyces cerevisiae] / hypothetical protein / PREDICTED: Gallus gallus similar to actin-like (LOC428337), partial mRNA. / PREDICTED: similar to actin-like, partial; / --- / --- / --- / ---
RIGG12889	352.9	0.0022	CCAGTGTATGGACATAAAAGATAAATTACTTGAGCAAGCGAAGAAGCATCTTTCTGAACCCATGGTGCATGAG		ENSGALT00000008928.1 / Gallus gallus finished cDNA, clone ChEST190d17 / 603369518F1 CSEQRBN19 Gallus gallus cDNA clone ChEST274c5 5', mRNA sequence / Finished cDNA, clone ChEST970h14 / / --- / --- / --- / --- / 603369518F1 CSEQRBN19 Gallus gallus cDNA clone ChEST274c5 5', mRNA sequence / Finished cDNA, clone ChEST970h14
RIGG03748	287.8	0.002	ACAGGTAGCAACGCTAAGGCTGATTGCAGTGTTCCACCTTTACCTTCAGAAAATGCATCACATTACAGCA	Partial Contig Hit 003443.1	Partial Contig Hit 003443.1 / Gallus gallus finished cDNA, clone ChEST53p19 / Gallus gallus finished cDNA, clone ChEST53p19 / Finished cDNA, clone ChEST53p19 / --- / --- / --- / --- / --- / Gallus gallus finished cDNA, clone ChEST53p19 / Finished cDNA, clone ChEST53p19
RIGG12266	263.3	0.000353	AGTGACTCTGATATCCCTGTTGATACACCTTTAACTGGAAATTCTGTTACATCATCACCATCCAGGAACGAA		ENSGALT00000007145.1 / UP|ZCHC8_CHICK (Q5F3D1) Zinc finger CCHC domain-containing protein 8, complete / Gallus gallus mRNA for hypothetical protein, clone 21c10 / Zinc finger, CCHC domain containing 8 (ZCCHC8), mRNA / ZCCHC8, RCJMB04_21c10: Zinc finger CCHC domain-containing protein 8 / PREDICTED: Gallus gallus similar to zinc finger, CCHC domain containing 8 (LOC416862), mRNA. / PREDICTED: similar to zinc finger, CCHC domain containing 8; / Zinc finger, CCHC domain containing 8 (ZCCHC8), mRNA; / --- / Gallus gallus mRNA for hypothetical protein, clone 21c10 / Zinc finger, CCHC domain containing 8 (ZCCHC8), mRNA
RIGG11310	182	0.0126	GGCCCACTCTCCCTCCAGGAAAATCATTTTAAAACCTGCTATCAAATATACTAGACCAACTCATCTCTCCTGT		ENSGALT00000004405.1 / --- / --- / --- / / --- / --- / --- / microtubule-associated protein 2; / --- / ---

Gene Name	Fold Change	p-value	SEQUENCE	Array Description	Blast/Database Description
RIGG00905	165.3	0.00722	ACTGGCCTCAAAAGTAAGTTCAAGC TTAATTTCTTAAGTTCAGCCTCAAAC TGCTAAGCCTGGATATGTG	Weakly similar to Q86JM6 (Q86JM6) Similar to Homo sapiens (Human). NPD010	Weakly similar to Q86JM6 (Q86JM6) Similar to Homo sapiens (Human). NPD010 / Gallus gallus finished cDNA, clone ChEST109b19 / Gallus gallus finished cDNA, clone ChEST109b19 / Finished cDNA, clone ChEST985l1 / --- / --- / --- / --- / --- / Gallus gallus finished cDNA, clone ChEST109b19 / Finished cDNA, clone ChEST985l1
RIGG00204	156	0.00239	TCCCTGAAGTCTACGTTCCCACCGT CTTTGAAAATTATGTAGCAGATATTG AAGTGGATGGAAAGCAGGT	RhoA GTPase [Gallus gallus]. [Source:RefSeq;Acc:NM_204704]	RhoA GTPase [Gallus gallus]. [Source:RefSeq / UP|O93467_CHICK (O93467) GTPase cRhoA (GTP-binding protein) (RhoA GTPase), complete / Gallus gallus ras homolog gene family, member A (RHOA), mRNA / GTPase cRhoA (cRhoA) / / --- / --- / --- / --- / Gallus gallus ras homolog gene family, member A (RHOA), mRNA / GTPase cRhoA (cRhoA)
RIGG00512	147.2	0.024	AGTACTGTTGTAGCAATAGAAAACC CCATTATCTCTGTCTGGGAAGAGAC TTTCGATTTTCTGCTTTGCAGGA	Weakly similar to Q9W622 (Q9W622) Nek2A	Weakly similar to Q9W622 (Q9W622) Nek2A / Gallus gallus finished cDNA, clone ChEST1003i15 / Gallus gallus finished cDNA, clone ChEST1003i15 / Finished cDNA, clone ChEST1003i15 / / --- / --- / --- / --- / Gallus gallus finished cDNA, clone ChEST1003i15 / Finished cDNA, clone ChEST1003i15
RIGG04546	123.2	0.0119	TCCAGGTTGGGAATGGATAGCACAT TTGAAAGCAAGAGAATGTTTCCTTA GCAGCTGTAATTGATAGTGCAGT	Genome Hit Contig117.28	Genome Hit Contig117.28 / RF|NP_001012857.1|61098191|NM_001012839 potassium channel tetramerisation domain containing 9 {Gallus gallus} (exp=-1; wgp=0; cg=0), complete / Gallus gallus mRNA for hypothetical protein, clone 20e15 / Potassium channel tetramerisation domain containing 9 (KCTD9), mRNA / --- / PREDICTED: Gallus gallus similar to potassium channel tetramerisation domain containing 9 (LOC419526), mRNA. / PREDICTED: similar to potassium channel tetramerisation domain containing 9; / Potassium channel tetramerisation domain containing 9 (KCTD9), mRNA; / --- / Gallus gallus mRNA for hypothetical protein, clone 20e15 / Potassium channel tetramerisation domain containing 9 (KCTD9), mRNA
RIGG18530	116.1	0.0075	TGGGGATGAATCTTCTAGGTTGTGG TTATGAATGTGCTTTACTGGGTGTA GATGCCAATGCTTCTTAGCA	Lipoprotein lipase precursor (EC 3.1.1.34) (LPL). [Source:SWISSPROT;Acc:P11602]	Lipoprotein lipase precursor (EC 3.1.1.34) (LPL). [Source:SWISSPROT / UP|Q9PSV4_CHICK (Q9PSV4) Lipoprotein lipase , complete / Gallus gallus lipoprotein lipase (LPL), mRNA / Adipose lipoprotein lipase (EC 3.1.1.34) / LPL: Lipoprotein lipase precursor / PREDICTED: Gallus gallus lipoprotein lipase (LPL), mRNA. / PREDICTED: lipoprotein lipase precursor; / Adipose lipoprotein lipase (EC 3.1.1.34); / --- / Gallus gallus lipoprotein lipase (LPL), mRNA / Adipose lipoprotein lipase (EC 3.1.1.34)

Gene Name	Fold Change	p-value	SEQUENCE	Array Description	Blast/Database Description
RIGG10733	111.7	0.0212	GAAATGTGATTACTGTGTAAGGTGC TCTGATGTCCTTCTGTGACTGTTCC AGAAATACCGAGCAAGCTGA		ENSGALT00000002784.1 / UP|PTDSR_CHICK (Q5ZMK5) Protein PTDSR, complete / Gallus gallus mRNA for hypothetical protein, clone 1m8 / Phosphatidylserine receptor (PTDSR), mRNA / PTDSR, RCJMB04_1m8: Protein PTDSR / PREDICTED: Gallus gallus similar to PTDSR protein (LOC417355), mRNA. / PREDICTED: similar to PTDSR protein; / Phosphatidylserine receptor (PTDSR), mRNA; / --- / Gallus gallus mRNA for hypothetical protein, clone 1m8 / Phosphatidylserine receptor (PTDSR), mRNA
RIGG03002	106.3	0.00625	TACATACTGTTCATACAACGTTTTCA GACCACTTCAGCTCCCCTTCTCAAT CTCTGTGGAAGAGCCGTGC	Contig Hit 341717.1	Contig Hit 341717.1 / Gallus gallus finished cDNA, clone ChEST422d17 / --- / --- / --- / --- / --- / --- / --- / --- / ---
RIGG11899	101.8	0.0129	GTACTTAAACTGGTCATGATGTTTGA AGGAAAAGCCAATGAAAGCAACCCA AAACCTTCCGGTCCACCTC		ENSGALT00000006096.1 / similar to UP|IBA2_MOUSE (Q9EQX4) Ionized calcium-binding adapter molecule 2, partial (87%) / --- / --- / similar to ionized calcium binding adapter molecule 2 / PREDICTED: Gallus gallus similar to ionized calcium binding adapter molecule 2 (Iba2) (LOC417179), mRNA. / PREDICTED: similar to ionized calcium binding adapter molecule 2 (Iba2); / --- / --- / --- / ---
RIGG00506	90.26	0.0137	CTGGGGTCTACACTACACTCATTTC TTTATTTATTGCTTGTGGAAAAGGG GAGGATAGCCGGACAGTAGT	Genome Hit Contig10.318	Genome Hit Contig10.318 / Gallus gallus finished cDNA, clone ChEST1003c8 / Gallus gallus finished cDNA, clone ChEST1003c8 / Finished cDNA, clone ChEST1003c8 / --- / --- / --- / --- / --- / Gallus gallus finished cDNA, clone ChEST1003c8 / Finished cDNA, clone ChEST1003c8
RIGG02737	79.06	0.0453	TCCCGTGCAAAGAAGCAGGGTTGAA ATTTTCTAAAGGAGAAATTCTTCAGA TTGTAAACCGGGAAGATCCA	Similar to Q811G2 (Q811G2) Similar to membrane protein, palmitoylated	Similar to Q811G2 (Q811G2) Similar to membrane protein, palmitoylated / homologue to UP|MPP6_HUMAN (Q9NZW5) MAGUK p55 subfamily member 6 (Veli-associated MAGUK 1) (VAM-1), complete / --- / --- / hypothetical protein / PREDICTED: Gallus gallus similar to membrane protein, palmitoylated 6; protein associated with Lin7 2; VELI-associated MAGUK 1; MAGUK protein p55T (LOC420622), mRNA. / PREDICTED: similar to membrane protein, palmitoylated 6; protein associated with Lin7 2; VELI-associated MAGUK 1; MAGUK protein p55T; / --- / --- / --- / ---
RIGG09123	73.63	0.00924	GATCTGGTTGTTTCTAAAATGACTCA GCTTGTCAGCACTGCTGGATCTACC CCATCACCACTGAGGCCTT		ENSGALG00000012566.1 / similar to UP|Q59EX7_HUMAN (Q59EX7) Synapsin-3 variant (Fragment), partial (39%) / 603158320F1 CSEQRBL03 Gallus gallus cDNA clone ChEST176g5 5', mRNA sequence / Transcribed locus, weakly similar to XP_515096.1 PREDICTED: similar to synapsin III isoform IIIa [Pan troglodytes] / --- / --- / --- / --- / synapsin III; / 603158320F1 CSEQRBL03 Gallus gallus cDNA clone ChEST176g5 5', mRNA sequence / Transcribed locus, weakly similar to XP_515096.1 PREDICTED: similar to synapsin III isoform IIIa [Pan troglodytes]

Gene Name	Fold Change	p-value	SEQUENCE	Array Description	Blast/Database Description
RIGG02849	69.94	0.025	AGATGCCTCCTTGTGACCATTATAC TAAAGAGCTCTTTGTTTCAGGCTCAT GTACAGTCAGGAATGGTTC	Weakly similar to O70305 (O70305) Ataxin-2	Weakly similar to O70305 (O70305) Ataxin-2 / Gallus gallus finished cDNA, clone ChEST397b4 / Gallus gallus finished cDNA, clone ChEST397b4 / Finished cDNA, clone ChEST397b4 / --- / --- / --- / --- / --- / Gallus gallus finished cDNA, clone ChEST397b4 / Finished cDNA, clone ChEST397b4
RIGG12250	69.12	0.0369	AAACTGGACACTCACTTTGGGCAAC AAGTGGAAGACCTTCTTTGAAACAG TCCACGTCTATTTGCGGAGC		ENSGALT00000007079.1 / similar to UP|FAM5B_HUMAN (Q9C0B6) Protein FAM5B precursor (BMP/retinoic acid-inducible neural-specific protein 2) (DBCCR1-like protein 2), partial (62%) / Gallus gallus mRNA for BMP/retinoic acid-inducible neural-specific protein (BRINP gene) / Deleted in bladder cancer 1 (DBC1), mRNA / --- / PREDICTED: Gallus gallus similar to BMP/retinoic acid-inducible neural-specific protein 2 (LOC424433), mRNA. / PREDICTED: similar to BMP/retinoic acid-inducible neural-specific protein 2; / Transcribed locus, strongly similar to NP_066988.1 BMP/retinoic acid-inducible neural-specific protein 2 [Homo sapiens]; / --- / 603860589F1 CSEQCHN75 Gallus gallus cDNA clone ChEST871a24 5', mRNA sequence / Transcribed locus, strongly similar to NP_066988.1 BMP/retinoic acid-inducible neural-specific protein 2 [Homo sapiens]
RIGG00129	66.42	0.0463	CTGCCCTTCACCATGCTGTAATTTAT GTAACTTCTGACCTCGTGGTGCCAA ACGGCCATTAAACGCATCC	Gallus gallus mRNA for hypothetical protein, clone 7e11	Gallus gallus mRNA for hypothetical protein, clone 7e11 / homologue to UP|IDHP_HUMAN (P48735) Isocitrate dehydrogenase [NADP], mitochondrial precursor (Oxalosuccinate decarboxylase) (IDH) (NADP(+)-specific ICDH) (IDP) (ICD-M) , partial (92%) / Gallus gallus mRNA for hypothetical protein, clone 7e11 / Similar to isocitrate dehydrogenase 2 (NADP+), mitochondrial (LOC431056), mRNA / RCJMB04_7e11: Hypothetical protein / PREDICTED: Gallus gallus similar to cytosolic NADP-dependent isocitrate dehydrogenase (LOC424112), mRNA. / PREDICTED: similar to cytosolic NADP-dependent isocitrate dehydrogenase; / --- / --- / Gallus gallus mRNA for hypothetical protein, clone 7e11 / Similar to isocitrate dehydrogenase 2 (NADP+), mitochondrial (LOC431056), mRNA
RIGG04704	65.22	0.0463	TGGACAGAGGAATTGGTTGCAAGTA TTCTGTAATGGTGGTATTCCTACTGA GCTGGCTATCTTGTATATGATAGA	Similar to Q9NV41 (Q9NV41) Hypothetical protein FLJ10936	Similar to Q9NV41 (Q9NV41) Hypothetical protein FLJ10936 / Gallus gallus finished cDNA, clone ChEST661h11 / Gallus gallus finished cDNA, clone ChEST661h11 / Finished cDNA, clone ChEST661h11 / --- / --- / --- / --- / transmembrane protein 19; / Gallus gallus finished cDNA, clone ChEST661h11 / Finished cDNA, clone ChEST661h11
RIGG10419	63.66	0.0264	AGCAGTGAATGTGTGGGGAGAAGC ACTCTTCAAAACTATTCTTAACAGCT CCAGAACTGTACGAACTTGA		ENSGALT00000001912.1 / homologue to UP|TBLX_MOUSE (Q9QXE7) F-box-like/WD-repeat protein TBL1X (Transducin beta-like 1X protein), complete / --- / --- / --- / --- / --- / --- / --- / --- / ---

Gene Name	Fold Change	p-value	SEQUENCE	Array Description	Blast/Database Description
RIGG01369	55.86	0.0389	GAAGAGGAAACTGAGAAGAAATCAGAAAACTTGGAGGTAACACCAGAAGTTCAAATTCCACAGATGGAGGA	Weakly similar to Q8T880 (Q8T880) Axonemal p83.9	Weakly similar to Q8T880 (Q8T880) Axonemal p83.9 / Gallus gallus finished cDNA, clone ChEST194g17 / Gallus gallus finished cDNA, clone ChEST194g17 / Finished cDNA, clone ChEST194g17 / --- / PREDICTED: Gallus gallus similar to cancer susceptibility candidate 1; lung adenoma susceptibility 1-like (LOC418206), mRNA. / PREDICTED: similar to cancer susceptibility candidate 1; lung adenoma susceptibility 1-like; / Transcribed locus, weakly similar to XP_216310.4 PREDICTED: similar to cancer susceptibility candidate 1 [Rattus norvegicus]; / --- / Gallus gallus finished cDNA, clone ChEST194g17 / Finished cDNA, clone ChEST194g17
RIGG04522	52.37	0.000755	ACCGCGGCTTCCTTAACTTCTGGGGCTATATCATTGTTCTGAACACTATGGTTCCCATTTCTCTCTATGTGA	Similar to AAQ19027 (AAQ19027) Possible aminophospholipid translocase	Similar to AAQ19027 (AAQ19027) Possible aminophospholipid translocase / Gallus gallus finished cDNA, clone ChEST638j6 / Gallus gallus finished cDNA, clone ChEST638j6 / Finished cDNA, clone ChEST638j6 / / --- / --- / --- / ATPase, Class I, type 8B, member 1; / Gallus gallus finished cDNA, clone ChEST638j6 / Finished cDNA, clone ChEST638j6
RIGG06779	49.88	0.0378	GATGAATCCAAAAGAGGAAAATACTCCCAGGGCTCATCCCGTCTCCTCAAGGAACTGGTGAAGGAAATGG	Weakly similar to Q90811 (Q90811) Hypothetical 28.6 kDa protein (Fragment)	Weakly similar to Q90811 (Q90811) Hypothetical 28.6 kDa protein (Fragment) / --- / Gallus gallus finished cDNA, clone ChEST939k23 / Finished cDNA, clone ChEST939k23 / --- / --- / --- / --- / --- / Gallus gallus finished cDNA, clone ChEST939k23 / Finished cDNA, clone ChEST939k23
RIGG00536	48.87	0.0442	ATCTCCTGGCGTTCTCACAGAATGCGCTCTTTGCCAATATATACCGTGTTTTGAGCCTAAAATCTGAGCC	Partial Contig Hit 336913.1	Partial Contig Hit 336913.1 / Gallus gallus finished cDNA, clone ChEST1005i15 / Gallus gallus finished cDNA, clone ChEST1005i15 / Finished cDNA, clone ChEST99d11 / --- / --- / --- / --- / --- / Gallus gallus finished cDNA, clone ChEST1005i15 / Finished cDNA, clone ChEST99d11
RIGG05346	48.45	0.0393	TAACTTTTGTCACTTTGCCTCTTGTCATCCGAGCGGCCGCGGGAGGGCAGTGGTGCTACGACTCG	Same gene AF079890; Gallus gallus immunoglobulin light chain gene, 3' hypersensitive site 2 sequence	Same gene AF079890; Gallus gallus immunoglobulin light chain gene, 3' hypersensitive site 2 sequence / Gallus gallus finished cDNA, clone ChEST746f14 / Gallus gallus finished cDNA, clone ChEST746f14 / Finished cDNA, clone ChEST746f14 / hypothetical protein / --- / --- / --- / --- / Gallus gallus finished cDNA, clone ChEST746f14 / Finished cDNA, clone ChEST746f14
RIGG11540	35.11	0.0124	GTGCCAAGGATTCTGATGAACTGTGATTTGAAGAGTAGTTTCAGATTTGAAGTGGTCCTGGCATAAAGCT		ENSGALT00000005070.1 / Gallus gallus finished cDNA, clone ChEST608o23 / Gallus gallus finished cDNA, clone ChEST608o23 / Finished cDNA, clone ChEST608o23 / --- / PREDICTED: Gallus gallus similar to F-box and leucine-rich repeat protein 8 (LOC415701), mRNA. / PREDICTED: similar to F-box and leucine-rich repeat protein 8; / Finished cDNA, clone ChEST608o23; / --- / Gallus gallus finished cDNA, clone ChEST608o23 / Finished cDNA, clone ChEST608o23

Gene Name	Fold Change	p-value	SEQUENCE	Array Description	Blast/Database Description
RIGG00159	34.86	0.0107	AATGACCCCATTCCCAGCTTATGATGACTAAATGCAGAGTTTGTGAGTTGTGGATTGCAGATCTGAAATTC	Gallus gallus mRNA for hypothetical protein, clone 9g24	Gallus gallus mRNA for hypothetical protein, clone 9g24 / RF|NP_001025831.1|71897217|NM_001030660 ras homolog gene family, member F {Gallus gallus} (exp=-1; wgp=0; cg=0), complete / Gallus gallus mRNA for hypothetical protein, clone 9g24 / Ras homolog gene family, member F (in filopodia) (RHOF), mRNA / RCJMB04_9g24: Hypothetical protein / --- / --- / Ras homolog gene family, member F (in filopodia) (RHOF), mRNA; / --- / Gallus gallus mRNA for hypothetical protein, clone 9g24 / Ras homolog gene family, member F (in filopodia) (RHOF), mRNA
RIGG04528	33.08	0.0414	ACAGAGAAGCAAACTCCCATTGAACAGATGCTCTTATGTGCTTTTGCAGTCTTTGCTGTTCAGAGCGTCT	Weakly similar to Q95JC9 (Q95JC9) Basic proline-rich protein	Weakly similar to Q95JC9 (Q95JC9) Basic proline-rich protein / Gallus gallus finished cDNA, clone ChEST151e15 / --- / --- / --- / --- / --- / --- / --- / ---
RIGG19977	31.84	0.0408	ATAGTGCAGGTAGCTTGGCATTGGACCTACTGTAATGTGGGCACTTACAGTACTGTTAGATAAAG	mi-RNA	mi-RNA: gga-mir-20b / --- / --- / --- / --- / --- / --- / --- / --- / --- / ---
RIGG11790	27.56	0.00929	GCGTTTTGGCTGCCTCTGTCAGGGATCTGTGCAACTCAGGGAGCAAACCCAAGCTGCTTTGAAGC		ENSGALT00000005800.1 / similar to UP|Q7SFQ1_NEUCR (Q7SFQ1) Predicted protein, partial (8%) / --- / --- / --- / --- / --- / --- / Fanconi anemia, complementation group F; / 603836158F1 CSEQRBN10 Gallus gallus cDNA clone ChEST822c16 5', mRNA sequence / Transcribed locus, weakly similar to NP_073562.1 Fanconi anemia, complementation group F [Homo sapiens]
RIGG01018	26.53	0.00383	TCTCCATTCGAACAGATAACCGAGGGTTTCTTTCACTGCAGTATATGATCAGGAATGAAGATGGACAGATT	Similar to Q9QWZ3 (Q9QWZ3) RAD1	Similar to Q9QWZ3 (Q9QWZ3) RAD1 / Gallus gallus finished cDNA, clone ChEST132c10 / gPGC_EST03525 Embryonic gonadal PGC cDNA Library Gallus gallus cDNA 5', mRNA sequence / Finished cDNA, clone ChEST132c10 / similar to checkpoint protein / --- / --- / Finished cDNA, clone ChEST132c10; / RAD1 homolog (S. pombe); / gPGC_EST03525 Embryonic gonadal PGC cDNA Library Gallus gallus cDNA 5', mRNA sequence / Finished cDNA, clone ChEST132c10
RIGG05045	25.6	0.0229	ACCCAAATGAAAGAGCTTTACAGTGTAACAGTGGTTGACAAAGGCAGTTTAATTCCTTCCAGCAAGATGC	Weakly similar to Q8BV52 (Q8BV52) Protein-tyrosine phosphatase	Weakly similar to Q8BV52 (Q8BV52) Protein-tyrosine phosphatase / Gallus gallus finished cDNA, clone ChEST704b12 / Gallus gallus finished cDNA, clone ChEST704b12 / Finished cDNA, clone ChEST704b12 / --- / --- / --- / --- / --- / Gallus gallus finished cDNA, clone ChEST704b12 / Finished cDNA, clone ChEST704b12
RIGG04588	23.47	0.0303	GCTCCTGATGGAGTAACTTGCTAAAGGAGAGTGTTTCTGTAAAGTTTTGCTTTGGCAGGGTTTTCATGGG	Genome Hit Contig148.48	Genome Hit Contig148.48 / --- / Gallus gallus finished cDNA, clone ChEST648p18 / Finished cDNA, clone ChEST648p18 / --- / --- / --- / --- / --- / Gallus gallus finished cDNA, clone ChEST648p18 / Finished cDNA, clone ChEST648p18

Gene Name	Fold Change	p-value	SEQUENCE	Array Description	Blast/Database Description
RIGG19797	23.17	0.0406	CACTCTCTGACGGAGGCTGACATCC ACGAGCTGGCACGGAAGACAGATG GCTACTCTGGGGCTGA		ENSGALT00000028255.1 / similar to UP|Q66IY7_XENLA (Q66IY7) MGC84050 protein, partial (44%) / Gallus gallus mRNA for hypothetical protein, clone 24f7 / Vacuolar protein sorting 4 homolog B (S. cerevisiae) (VPS4B), mRNA / similar to vacuolar protein sorting factor 4A; SKD1-homolog; vacuolar sorting protein 4; vacuolar protein sorting 4A (yeast homolog), partial / PREDICTED: Gallus gallus similar to SKD1 (LOC420901), mRNA. / PREDICTED: similar to SKD1; / --- / --- / --- / ---
RIGG09681	20.57	0.04	TACATCAGCAGCTTTGTCACTCAGC ACAAATGAGACAACCAGGTCAAATG TTCGAGTGACTGTGACTGCA		ENSGALG00000017271.1 / --- / --- / --- / --- / --- / --- / --- / --- / --- / ---
RIGG01073	19.56	0.00962	GAACATCAAATCAAATAATTACATGC TGTTACATCCTTGTTGCTGTTTGCTC ATCCACCCACAGCCATTCC	Genome Hit Contig20.18	Genome Hit Contig20.18 / --- / Gallus gallus finished cDNA, clone ChEST144i18 / Finished cDNA, clone ChEST144i18 / --- / --- / --- / --- / --- / Gallus gallus finished cDNA, clone ChEST144i18 / Finished cDNA, clone ChEST144i18
RIGG00998	17.38	0.0161	AATCCGAGAAACTGGACTTCAAAGA TAAGGTGCAATCTAAAATTGGGTCC TTAGATAACATCAGCCACGT	Similar to Q91WK4 (Q91WK4) Microtubule-associated protein tau	Similar to Q91WK4 (Q91WK4) Microtubule-associated protein tau / Gallus gallus finished cDNA, clone ChEST129a5 / 603139517F1 CSEQCHL15 Gallus gallus cDNA clone ChEST129a5 5', mRNA sequence / Finished cDNA, clone ChEST129a5 / --- / --- / --- / --- / --- / Gallus gallus finished cDNA, clone ChEST843h3 / Finished cDNA, clone ChEST843h3
RIGG09089	16.77	0.0375	TCAGTTGTCTCCACTCGACCTTTATC CATCATTAAAGATGTCTTGTCAATTA GACGATCTCAACATGCGATGG		ENSGALG00000012270.1 / similar to UP|Q5T097_HUMAN (Q5T097) Utrophin (Homologous to dystrophin), partial (22%) / 603771141F1 CSEQRBN14 Gallus gallus cDNA clone ChEST704b11 5', mRNA sequence / Transcribed locus, weakly similar to NP_000100.2 dystrophin Dp427c isoform [Homo sapiens] / --- / --- / Finished cDNA, clone ChEST986m10; / utrophin (homologous to dystrophin); / 603771141F1 CSEQRBN14 Gallus gallus cDNA clone ChEST704b11 5', mRNA sequence / Transcribed locus, weakly similar to NP_000100.2 dystrophin Dp427c isoform [Homo sapiens]
RIGG17455	10.76	0.0482	AGGTGGAGACTTACTGACCGGGTTT TGACCAAACTATTTCCTACTTTGTGA TTGTGCAACAAAATCTAGCTT	granzyme A [Gallus gallus]. [Source:RefSeq;Acc:NM_204457]	granzyme A [Gallus gallus]. [Source:RefSeq / UP|Q7SZC3_CHICK (Q7SZC3) Granzyme A precursor, complete / Gallus gallus mRNA for granzyme A precursor (GZMA gene) / Granzyme A (granzyme 1, cytotoxic T-lymphocyte-associated serine esterase 3) (GZMA), mRNA / / Gallus gallus granzyme A (GZMA), mRNA. / granzyme A; / Granzyme A (granzyme 1, cytotoxic T-lymphocyte-associated serine esterase 3) (GZMA), mRNA; / --- / Gallus gallus mRNA for granzyme A precursor (GZMA gene) / Granzyme A (granzyme 1, cytotoxic T-lymphocyte-associated serine esterase 3) (GZMA), mRNA

Gene Name	Fold Change	p-value	SEQUENCE	Array Description	Blast/Database Description
RIGG05869	10.06	0.000793	CACCTCCTGTGTGCAGGTAGGTAAG AGCCCTCCGGTTGTATTCACTTTCC TTCTGCCTGGAGCCT	Genome Hit Contig804.8	Genome Hit Contig804.8 / Gallus gallus finished cDNA, clone ChEST813a14 / Gallus gallus finished cDNA, clone ChEST813a14 / Finished cDNA, clone ChEST813a14 / --- / --- / --- / --- / --- / Gallus gallus finished cDNA, clone ChEST813a14 / Finished cDNA, clone ChEST813a14
RIGG02356	10	0.000502	CCTCAACTGTGTTCCTCTTCTCTGC ATAGCCCCATACATTTCATGTCCAT GTTTCCTCATTAGAATGTATG	Genome Hit Contig21.249	Genome Hit Contig21.249 / --- / Gallus gallus finished cDNA, clone ChEST331j10 / Finished cDNA, clone ChEST331j10 / --- / --- / --- / --- / --- / Gallus gallus finished cDNA, clone ChEST331j10 / Finished cDNA, clone ChEST331j10
RIGG15311	7.366	0.0265	GTGTAGATTTCATTGCAGCCACACA TTTCTCTACAGATTTGCAGACCACAA ACAACTTCCAGGCTTTCCT		ENSGALT00000015937.1 / --- / --- / --- / --- / PREDICTED: Gallus gallus similar to RIKEN cDNA 2310057J18 (LOC421712), mRNA. / PREDICTED: similar to RIKEN cDNA 2310057J18; / --- / --- / --- / ---
RIGG11385	7.206	0.0315	AGTCTGCAAACCTTACTCCATTGCC TTCAGTAGGACTACGTGCATGAGTC AGATTTGCATAGATTGTATTTTAC		ENSGALT00000004601.1 / Gallus gallus finished cDNA, clone ChEST464g7 / gPGC_EST00889 Embryonic gonadal PGC cDNA Library Gallus gallus cDNA 5', mRNA sequence / Finished cDNA, clone ChEST464g7 / --- / --- / --- / --- / --- / --- / ---
RIGG17049	6.942	0.0193	CTGTGTGCTGTCATACCTCTACTGC TGTTCAGTGTCCTTTAACAAGCTGTA TTGTGAAGTTAGAAGTGGTT		ENSGALT00000020840.1 / --- / Gallus gallus finished cDNA, clone ChEST795a3 / Finished cDNA, clone ChEST748e22 / similar to MGC82474 protein, partial / PREDICTED: Gallus gallus similar to glucosaminyl (N-acetyl) transferase 2 isoform A; I beta-1,6-N-acetylglucosaminyltransferase; blood group Ii; N-acetyllactosaminide beta-1,6-N-acetylglucosaminyltransferase; Ii blood group; glucosaminyl (N-acetyl) transf / PREDICTED: similar to glucosaminyl (N-acetyl) transferase 2 isoform A; I beta-1,6-N-acetylglucosaminyltransferase; blood group Ii; N-acetyllactosaminide beta-1,6-N-acetylglucosaminyltransferase; Ii blood group; glucosaminyl (N-acetyl) transferase 5 ..., p; / --- / --- / --- / ---
RIGG00141	6.915	0.0298	ACTCTTCAATGAAATGCTTCAGAGG GATTTTGGTGTCAGAATTTATAAAGC ACTGATTTCCCTCCCAGAGAG	Gallus gallus partial mRNA for hypothetical protein, clone 7o17	Gallus gallus partial mRNA for hypothetical protein, clone 7o17 / homologue to GB|AAH26036.1|34191778|BC026036 CCAR1 protein {Homo sapiens} (exp=-1; wgp=0; cg=0), complete / Gallus gallus partial mRNA for hypothetical protein, clone 7o17 / Hypothetical protein, clone 7o17 / RCJMB04_7o17: Hypothetical protein (Fragment) / --- / --- / --- / --- / Gallus gallus partial mRNA for hypothetical protein, clone 7o17 / Hypothetical protein, clone 7o17
RIGG15033	6.636	0.0207	CCTATCCCTCTATTTACCTATCTGTT TCTTTATCCATTTATTCACTTTATTCA CCGGCAGAGTCTCGGGGCT		ENSGALT00000015205.1 / --- / --- / --- / --- / --- / --- / --- / --- / --- / ---
RIGG01059	6.539	0.0172	CCTGTCCAGTCACCAGATTTACTGT ATTGAAAACAAAGCACTGTCCTCAC TAGTCTTTAGCTGAATGTTTGC	Similar to O42110 (O42110) ORF2	Similar to O42110 (O42110) ORF2 / DNA-dependent protein kinase catalytic subunit [Gallus gallus] / Gallus gallus finished cDNA, clone ChEST141a8 / Finished cDNA, clone ChEST141a8 / similar to ORF2 / --- / --- / --- / --- / Gallus gallus finished cDNA, clone ChEST141a8 / Finished cDNA, clone ChEST141a8

Gene Name	Fold Change	p-value	SEQUENCE	Array Description	Blast/Database Description
RIGG03613	6.466	0.0192	CTTCATCTCTGGGTTGTCTGACCAATTAATTCCACCAGTTATGATGAAGCAACTTTATGAATTATCCCCAGCTCG	Similar to Q9D0V5 (Q9D0V5) 1110065L07Rik protein	Similar to Q9D0V5 (Q9D0V5) 1110065L07Rik protein / Gallus gallus finished cDNA, clone ChEST527e23 / Gallus gallus mRNA for hypothetical protein, clone 17d11 / Abhydrolase domain containing 13 (ABHD13), mRNA / RCJMB04_17d11: Hypothetical protein / PREDICTED: Gallus gallus similar to 1110065L07Rik protein (LOC418763), mRNA. / PREDICTED: similar to 1110065L07Rik protein; / Abhydrolase domain containing 13 (ABHD13), mRNA; / --- / Gallus gallus mRNA for hypothetical protein, clone 17d11 / Abhydrolase domain containing 13 (ABHD13), mRNA
RIGG06621	6.415	0.0218	CTTAGGGGAATTCTTGCATCTTTTGCTTTTGTTGTGCATGAGTCACTTCAAAATAAATGTGCAGCATGGC	Genome Hit Contig93.55	Genome Hit Contig93.55 / Gallus gallus finished cDNA, clone ChEST918e14 / Gallus gallus finished cDNA, clone ChEST918e14 / Finished cDNA, clone ChEST918e14 / --- / --- / --- / --- / --- / Gallus gallus finished cDNA, clone ChEST918e14 / Finished cDNA, clone ChEST918e14
RIGG12285	6.114	0.0164	TGGACCTATCCATGTCTCCCTCTATGACTTGTCTTCTCTAGACAGCTTTGAGGAAAACTCTGTGCTGGAG		ENSGALT00000007195.1 / --- / Gallus gallus transient receptor potential cation channel, subfamily V, member 1 (TRPV1), mRNA / Vanilloid receptor-like protein / similar to Trpv2 protein / --- / --- / --- / transient receptor potential cation channel, subfamily V, member 2; / --- / ---
RIGG17567	6.048	0.021	CAGCCCAGGCATTTGCTAAGCGTTACTTGACTGATCTTATTCCCTCAGTCTTCAACAATCTTCACGAGAG		ENSGALT00000022341.1 / similar to UP|Q86UC2_HUMAN (Q86UC2) Radial spokehead-like 2, partial (48%) / testis_EST04229 Testis cDNA Library Gallus gallus cDNA 3', mRNA sequence / Transcribed locus, weakly similar to XP_315905.2 ENSANGP00000013556 [Anopheles gambiae str. PEST] / --- / PREDICTED: Gallus gallus similar to radial spokehead-like 2; radial spoke protein 3 (LOC421663), mRNA. / PREDICTED: similar to radial spokehead-like 2; radial spoke protein 3; / --- / --- / --- / ---
RIGG04671	6.035	0.0143	CTTCCTCTTGTCTTCTAATTTGACTGTATGAATTGCAGTGTATTCTGTGTTCGGTTTGGAGTCACCTGTGT	Genome Hit Contig248.43	Genome Hit Contig248.43 / UP|Q7N3L6_PHOLL (Q7N3L6) PTS system, mannose-specific IID component (EIID-MAN) (Mannose-permease IID component) (Phosphotransferase enzyme II, D component) (EII-M-MAN), partial (5%) / Gallus gallus finished cDNA, clone ChEST659a13 / Finished cDNA, clone ChEST659a13 / --- / --- / --- / --- / --- / Gallus gallus finished cDNA, clone ChEST659a13 / Finished cDNA, clone ChEST659a13
RIGG07421	5.941	0.00701	GGTGATGTGGAGGATGGGAAGTGGGGAGACTGTTTTCTGTAGTGTTATCCAATTGAGTAGTTTCAGTGAG		Contig_242_reverse / similar to UP|Q3GRK8_9GAMM (Q3GRK8) Cobalamin-5-phosphate synthase CobS, partial (8%) / --- / --- / --- / --- / --- / --- / --- / --- / ---

Gene Name	Fold Change	p-value	SEQUENCE	Array Description	Blast/Database Description
RIGG06239	5.821	0.0413	AGGTTAACTAGGGAAAAGAGTATGTATATGTGTGCAGTGGTTTCTTTCCTACCTCTGATGTTACAGCCTGAC	Weakly similar to MYSE_DICDI (Q03479) Myosin IE heavy chain	Weakly similar to MYSE_DICDI (Q03479) Myosin IE heavy chain / Gallus gallus finished cDNA, clone ChEST865e5 / Gallus gallus finished cDNA, clone ChEST865e5 / Finished cDNA, clone ChEST865e5 / RCJMB04_6o17: Hypothetical protein / --- / --- / --- / --- / Gallus gallus finished cDNA, clone ChEST865e5 / Finished cDNA, clone ChEST865e5
RIGG06974	5.727	0.00648	CTACTTACAGTTAAGACAGTATCTCAGACCTTGCATTGTCCCTACTTGCAATCAGTTCTGTTGGATGCAG	Contig Hit 333438.2	Contig Hit 333438.2 / Gallus gallus finished cDNA, clone ChEST971o7 / --- / --- / --- / --- / --- / --- / --- / --- / ---
RIGG02903	5.393	0.0453	ATCGGGCTGCAGTATGTCCTTTCTCTTGTGTTGAAGTTTGTTCTTCCTAATGCTCGTCTTCTCTAAAATT	Weakly similar to Q8NHR2 (Q8NHR2) Similar to RIKEN cDNA 4921522K05 gene	Weakly similar to Q8NHR2 (Q8NHR2) Similar to RIKEN cDNA 4921522K05 gene / Gallus gallus finished cDNA, clone ChEST403f19 / Gallus gallus finished cDNA, clone ChEST403f19 / Finished cDNA, clone ChEST403f19 / --- / PREDICTED: Gallus gallus similar to hypothetical protein MGC26710 (LOC417012), mRNA. / PREDICTED: similar to hypothetical protein MGC26710; / --- / --- / Gallus gallus finished cDNA, clone ChEST403f19 / Finished cDNA, clone ChEST403f19
RIGG03984	5.323	0.0357	TGACCGGCTAAAGAAAGTTTGTTATCCTGCAGTCTAAGTGTATTGTACTTACCAGCAGTGTTTTCGGTTTG	Genome Hit Contig37.167	Genome Hit Contig37.167 / --- / gPGC_EST10467 Embryonic gonadal PGC cDNA Library Gallus gallus cDNA 5', mRNA sequence / Finished cDNA, clone ChEST569b12 / --- / --- / --- / --- / --- / gPGC_EST10467 Embryonic gonadal PGC cDNA Library Gallus gallus cDNA 5', mRNA sequence / Finished cDNA, clone ChEST569b12
RIGG06526	5.305	0.00334	TGCAGTGGTCACATTACATAAAGTTAAGCATAGAAGACCTATACGGCTCTCTTTGTTGTCAGGTATCCCTT	Contig Hit 355297.2	Contig Hit 355297.2 / Gallus gallus finished cDNA, clone ChEST908o9 / 603949740F1 CSEQCHN03 Gallus gallus cDNA clone ChEST908o9 5', mRNA sequence / Finished cDNA, clone ChEST908o9 / --- / --- / --- / --- / --- / 603949740F1 CSEQCHN03 Gallus gallus cDNA clone ChEST908o9 5', mRNA sequence / Finished cDNA, clone ChEST908o9
RIGG11767	5.064	0.0318	GCACTTGAATCTTGATCCAGTATCTAAGCTTCCACCAGTTATACGACAGACAATCTCCACTGCTTTAATACCTG		ENSGALT00000005752.1 / --- / --- / --- / similar to hypothetical protein FLJ13231 / --- / hypothetical protein LOC65250; / --- / --- / --- / ---
RIGG05887	5.058	0.00537	CCCCATGCTGGATGGTTGTCACACACCTTACACCTGAAGTAATGCACTTTGGTTAACTTTGGTAGCGCAG	Weakly similar to Q8R3R9 (Q8R3R9) RIKEN cDNA 2700086I23 gene	Weakly similar to Q8R3R9 (Q8R3R9) RIKEN cDNA 2700086I23 gene / homologue to UP|PFD1_PONPY (Q5RAM7) Prefoldin subunit 1, partial (97%) / --- / --- / / PREDICTED: Gallus gallus similar to prefoldin 1 (LOC416142), mRNA. / PREDICTED: similar to prefoldin 1; / --- / --- / --- / ---
RIGG14183	4.992	0.00923	CTAAGTTTGACTCAGCCTACATACCAGTGACACTTCCTAACATTCAAAGGCCTGCATTAATGACAACCCT		ENSGALT00000012812.1 / --- / --- / --- / --- / --- / --- / --- / --- / --- / ---

Gene Name	Fold Change	p-value	SEQUENCE	Array Description	Blast/Database Description
RIGG04023	4.969	0.0381	AATCTGGAAAGACGGCTTTTGTGAC AGTAGCGACAGGAAATGATTTCTCT TTCTTGTCCTTCCCTCCTGC	Weakly similar to PA2Q_TRIFL (Q8JIG0) Phospholipase A2 isozyme cPLA-B'(A) pre	Weakly similar to PA2Q_TRIFL (Q8JIG0) Phospholipase A2 isozyme cPLA-B'(A) pre / --- / Gallus gallus finished cDNA, clone ChEST575g1 / Finished cDNA, clone ChEST575g1 / hypothetical protein / --- / --- / --- / --- / Gallus gallus finished cDNA, clone ChEST575g1 / Finished cDNA, clone ChEST575g1
RIGG02033	4.965	0.0464	GTGACAGCATTGACCCTCTGAAGTC GCTCATTTTCCTCCTCTGCTTTACCA TGGTTGTGGCCTTAATAGG	Similar to Q9H0Y0 (Q9H0Y0) Hypothetical protein	Similar to Q9H0Y0 (Q9H0Y0) Hypothetical protein / Gallus gallus finished cDNA, clone ChEST275l19 / gPGC_EST07219 Embryonic gonadal PGC cDNA Library Gallus gallus cDNA 5', mRNA sequence / Finished cDNA, clone ChEST803k18 / --- / PREDICTED: Gallus gallus similar to APG10 autophagy 10-like (LOC427322), mRNA. / PREDICTED: similar to APG10 autophagy 10-like; / --- / --- / --- / ---
RIGG13910	4.937	0.0123	CGACTCCTGAACACAACTGAATTAA TAAGATTCATCGTCTACTTCACTACG GAACAGGGTTTGCTTTCCC		ENSGALT00000012018.1 / Gallus gallus finished cDNA, clone ChEST495a8 / Gallus gallus mRNA for hypothetical protein, clone 7j3 / Hypothetical protein, clone 7j3 / --- / --- / --- / Hypothetical protein, clone 7j3; / --- / Gallus gallus mRNA for hypothetical protein, clone 7j3 / Hypothetical protein, clone 7j3
RIGG10058	4.899	0.00324	CCCTTCTTCCTCATTGCCACCGTCA TCCAGCTCTTCTACCGCGGGCGCAT CTGGAACATCCTCCT		ENSGALT00000000896.1 / homologue to UP|Q811Y6_RAT (Q811Y6) Hyaluronan synthase 3 (Fragment), partial (64%) / Gallus gallus hyaluronan synthase 2 (HAS2), mRNA / Hyaluronan synthase 2 (HAS2) / HAS2: Hyaluronan synthase 2 / Gallus gallus hyaluronan synthase 2 (HAS2), mRNA. / hyaluronan synthase 2; / Hyaluronan synthase 2 (HAS2); / --- / --- / ---
RIGG17261	4.822	0.0163	CACAGAGCCACATCCCATCCTTGTC CTCTCCCCACATGCAGATCACATAT ATCCATCTCCTTGCATTTCC		ENSGALT00000021507.1 / --- / Gallus gallus mRNA for hypothetical protein, clone 9p17 / Similar to immunoglobulin-like receptor CHIR-B2 precursor (LOC425534), mRNA / --- / --- / --- / --- / --- / --- / ---
RIGG08878	4.802	0.0133	CTGCACATTTTACCATCCAACTATTG CTGTCCCTCCACGCCATGCCTTGAA ATGGACTCGAACTCAAACC		ENSGALG00000010622.1 / RF|NP_001012604.1|60302818|NM_001012586 nuclear protein UKp68 {Gallus gallus} (exp=-1; wgp=0; cg=0), complete / Gallus gallus mRNA for hypothetical protein, clone 4a10 / Zinc finger CCCH-type containing 14 (ZC3H14), mRNA / RCJMB04_4a10: Hypothetical protein / PREDICTED: Gallus gallus similar to nuclear protein UKp68 isoform 1 (LOC423399), mRNA. / PREDICTED: similar to nuclear protein UKp68 isoform 1; / Zinc finger CCCH-type containing 14 (ZC3H14), mRNA; / --- / Gallus gallus mRNA for hypothetical protein, clone 4a10 / Zinc finger CCCH-type containing 14 (ZC3H14), mRNA
RIGG01035	4.723	0.00365	TACAAATGGTTTACATGAGATGTGA AGGATTTCCCGAGGAGCAGAACTGA AAGCAGCGGCAGTGAGAGGG	Genome Hit Contig45.134	Genome Hit Contig45.134 / Gallus gallus finished cDNA, clone ChEST135f17 / Gallus gallus finished cDNA, clone ChEST135f17 / Finished cDNA, clone ChEST135f17 / --- / --- / --- / --- / --- / Gallus gallus finished cDNA, clone ChEST135f17 / Finished cDNA, clone ChEST135f17

Gene Name	Fold Change	p-value	SEQUENCE	Array Description	Blast/Database Description
RIGG17132	4.628	0.0189	TAAAGAAATGGTTTAGCATTCTTCCAAAACCCACTTCCATCCAATCAGATAACGGCTCTCATTTCACCTC	pol-like protein ENS-3 [Gallus gallus]. [Source:RefSeq;Acc:NM_204632]	pol-like protein ENS-3 [Gallus gallus]. [Source:RefSeq / UP|Q98ST1_CHICK (Q98ST1) Pol-like protein ENS-3, complete / Gallus gallus pol-like protein ENS-3 (ENS-3), mRNA / Pol-like protein ENS-3 (ens-3) / ens-3: Pol-like protein ENS-3 / Gallus gallus pol-like protein ENS-3 (ENS-3), mRNA. / pol-like protein ENS-3; / Pol-like protein ENS-3 (ens-3); / --- / Gallus gallus pol-like protein ENS-3 (ENS-3), mRNA / Pol-like protein ENS-3 (ens-3)
RIGG14870	4.595	0.00533	CAACTACGAGCAGTTCATCCCCGCAGACTCATTCATTCACGTCGATGACTTCAGTTCCCTGGCAGATTTG	CFTII. [Source:SPTREMBL;Acc:Q8UWB9]	CFTII. [Source:SPTREMBL / UP|Q8UWB9_CHICK (Q8UWB9) CFTII, complete / Gallus gallus fucosyltransferase 7 (alpha (1,3) fucosyltransferase) (FUT7), mRNA / Fucosyltransferase 7 (alpha (1,3) fucosyltransferase) (FUT7), mRNA / CFTII: CFTII / PREDICTED: Gallus gallus similar to CFTII (LOC427782), mRNA. / PREDICTED: similar to CFTII; / Fucosyltransferase 7 (alpha (1,3) fucosyltransferase) (FUT7), mRNA; / --- / Gallus gallus fucosyltransferase 7 (alpha (1,3) fucosyltransferase) (FUT7), mRNA / Fucosyltransferase 7 (alpha (1,3) fucosyltransferase) (FUT7), mRNA
RIGG10805	4.558	0.0465	AGAATGTTTCACATTTGCTTCGATGTGCAAAGAAGAGGTAGACAGGTACAACTTTGTTATGCTGGCCTTG		ENSGALT00000003010.1 / similar to GB|AAH43595.1|28175796|BC043595 RFT1 homolog {Homo sapiens} (exp=-1; wgp=0; cg=0), partial (48%) / RJA088F06.ab1 RJbrain Gallus gallus cDNA 5', mRNA sequence / Transcribed locus, weakly similar to XP_562163.1 ENSANGP00000025925 [Anopheles gambiae str. PEST] / / --- / --- / --- / RFT1 homolog (S. cerevisiae); / --- / ---
RIGG03490	4.55	0.0316	AGAGTCTTCTTCAACCAAAGTGATATTCTGATTCTGTAACACCGTCTTTTCCACACTGGAGTTGATCTCAGTG	Contig Hit 341775.3	Contig Hit 341775.3 / Gallus gallus finished cDNA, clone ChEST502k7 / Gallus gallus finished cDNA, clone ChEST502k7 / Finished cDNA, clone ChEST502k7 / --- / --- / --- / --- / --- / Gallus gallus finished cDNA, clone ChEST502k7 / Finished cDNA, clone ChEST502k7
RIGG07797	4.524	0.0239	AAAATAATTACGTTCACGAAGAACAACCAATTTCAGGCTCTGTTACAATATGCTGACCCCATGAGTGCTC		ENSGALG00000001962.1 / RF|NP_001026106.1|71896363|NM_001030935 polypyrimidine tract-binding protein 1 {Gallus gallus} (exp=-1; wgp=0; cg=0), complete / Gallus gallus mRNA for hypothetical protein, clone 3b17 / Polypyrimidine tract binding protein 1 (PTBP1), mRNA / RCJMB04_3b17: Hypothetical protein / --- / --- / Polypyrimidine tract binding protein 1 (PTBP1), mRNA; / --- / Gallus gallus mRNA for hypothetical protein, clone 3b17 / Polypyrimidine tract binding protein 1 (PTBP1), mRNA
RIGG15228	4.504	0.000587	AGGAGTGTTCAACATCGATAGTTCAGAGCCCAGTCCTTATGTCAGAGCTTCTTTCTCTCTGCCTTCTCCA		ENSGALT00000015723.1 / similar to UP|Q5E9N4_BOVIN (Q5E9N4) Alpha-aminoadipate aminotransferase, partial (65%) / --- / --- / / PREDICTED: Gallus gallus similar to alpha-aminoadipate aminotransferase; L-kynurenine/alpha-aminoadipate aminotransferase; kynurenine aminotransferase II (LOC428728), mRNA. / PREDICTED: similar to alpha-aminoadipate aminotransferase; L-kynurenine/alpha-aminoadipate aminotransferase; kynurenine aminotransferase II; / --- / --- / --- / ---

Gene Name	Fold Change	p-value	SEQUENCE	Array Description	Blast/Database Description
RIGG02837	4.5	0.0442	GGAGCCTCACCAGAAACAATTAGTTCAGCTAAGTTTGATTGATGAGCAGAAGACTTTCTTCCTTCTTTGCAAG	Same gene AC140940; Gallus gallus chromosome UNK clone TAM32-30H10, complete sequence	Same gene AC140940; Gallus gallus chromosome UNK clone TAM32-30H10, complete sequence / --- / Gallus gallus finished cDNA, clone ChEST641h14 / Finished cDNA, clone ChEST641h14 / --- / --- / --- / --- / --- / Gallus gallus finished cDNA, clone ChEST641h14 / Finished cDNA, clone ChEST641h14
RIGG16262	4.447	0.00607	AGTGCCATGTTACTTACCCTTGCCGTGTCCTTTCTATGTAAGCAACCTAATAAATTGTTCAGTGAGAGGC		ENSGALT00000018590.1 / similar to UP|Q4RY83_TETNG (Q4RY83) Chromosome 3 SCAF14978, whole genome shotgun sequence. (Fragment), partial (21%) / 603602869F1 CSEQCHN54 Gallus gallus cDNA clone ChEST580i18 5', mRNA sequence / Transcribed locus, strongly similar to XP_422063.1 PREDICTED: similar to phospholipase C delta 3; PLC delta3 [Gallus gallus] / --- / PREDICTED: Gallus gallus similar to Phospholipase C, delta 1 (LOC420416), mRNA. / PREDICTED: similar to Phospholipase C, delta 1; / --- / --- / --- / ---
RIGG11679	4.427	0.0185	AAGGCCGGTTCCTCTGTTCGCATCTTTATCTACACAAGTGGATATCATGGAGATTTTGTGAGATCTAAAGC		ENSGALT00000005494.1 / --- / --- / --- / --- / --- / --- / --- / --- / --- / ---
RIGG19034	4.408	0.0157	TTCTCAAAAGCTTGACCTGAAGAATATCTCAAGGCAACGGTTGACATGGAAGTTGATTTGTGATGATGCG		ENSGALT00000026232.1 / Gallus gallus finished cDNA, clone ChEST696k17 / Gallus gallus finished cDNA, clone ChEST696k17 / Finished cDNA, clone ChEST696k17 / --- / --- / --- / --- / --- / Gallus gallus finished cDNA, clone ChEST696k17 / Finished cDNA, clone ChEST696k17
RIGG14023	4.406	0.0265	ATGTCTCCAAGCAACAACGTGGTTCCCATTCATGTCCCTCCAACCACTGAAAACAAACCAAAGATGCAGT		ENSGALT00000012312.1 / UP|RB11A_CHICK (Q5ZJN2) Ras-related protein Rab-11A, complete / Gallus gallus mRNA for hypothetical protein, clone 16p4 / RAB11A, member RAS oncogene family (RAB11A), mRNA / RAB11A, RCJMB04_16p4: Ras-related protein Rab-11A / PREDICTED: Gallus gallus similar to RAB11a, member RAS oncogene family (LOC415544), mRNA.PREDICTED: Gallus gallus similar to RAB11a, member RAS oncogene family (LOC431549), partial mRNA. / PREDICTED: similar to RAB11a, member RAS oncogene family;PREDICTED: similar to RAB11a, member RAS oncogene family, partial; / RAB11A, member RAS oncogene family (RAB11A), mRNA; / --- / Gallus gallus mRNA for hypothetical protein, clone 16p4 / RAB11A, member RAS oncogene family (RAB11A), mRNA
RIGG03450	4.39	0.000239	TACTCCATTGGCACTGTTGGCACTCAGGATGTGGACTGCGACTTCATTGATTTTGCTTATGTCCGTGTCT	Similar to Q9D8Z6 (Q9D8Z6) 9430023L20Rik protein (RIKEN cDNA 9430023L2	Similar to Q9D8Z6 (Q9D8Z6) 9430023L20Rik protein (RIKEN cDNA 9430023L2 / homologue to UP|Q6DE58_XENLA (Q6DE58) MGC80090 protein, complete / 603533603F1 CSEQCHN53 Gallus gallus cDNA clone ChEST491f1 5', mRNA sequence / Finished cDNA, clone ChEST498a18 / --- / --- / --- / --- / --- / 603533603F1 CSEQCHN53 Gallus gallus cDNA clone ChEST491f1 5', mRNA sequence / Finished cDNA, clone ChEST498a18

Gene Name	Fold Change	p-value	SEQUENCE	Array Description	Blast/Database Description
RIGG06927	4.365	0.0303	CCGTTCCTGGGTTTTCCATTCGTTTTCTATTTGAAGGAAGAAGCAAAATAAACCAGGCTTCATCTCTCTT	Weakly similar to Q8ML38 (Q8ML38) CG8772-PE	Weakly similar to Q8ML38 (Q8ML38) CG8772-PE / Gallus gallus finished cDNA, clone ChEST593p11 / Gallus gallus mRNA for hypothetical protein, clone 23e15 / Glutaminase (GLS), mRNA / RCJMB04_23e15: Hypothetical protein / --- / --- / Glutaminase (GLS), mRNA; / --- / --- / ---
RIGG15510	4.312	0.027	GCGCACTGCTTATCCCCGTTCCCACCCAGTTTGTAATTCTCTTCCACTTCAAACGGCCAAATTAA		ENSGALT00000016442.1 / --- / --- / --- / --- / --- / --- / --- / --- / --- / ---
RIGG05922	4.275	0.0238	TTGCTTAGCTCCCCACTAAGCTCCCATTGCCTCCCAGTATTGTCTAGAGTCTTCTTGTGCAAACTCAGTG	Partial Contig Hit 034294.1	Partial Contig Hit 034294.1 / Gallus gallus finished cDNA, clone ChEST81f11 / Gallus gallus finished cDNA, clone ChEST81f11 / Finished cDNA, clone ChEST81f11 / --- / --- / --- / --- / --- / Gallus gallus finished cDNA, clone ChEST81f11 / Finished cDNA, clone ChEST81f11
RIGG05511	4.194	0.000968	TTACCGCCGTTCTCCTCCCTTTGGTAACGTCTTTTATCTTAAGCTGTAGAAACAATATGTAGGTGCTTGAAAG	Genome Hit Contig68.178	Genome Hit Contig68.178 / GB|CR391483.1|CR391483.1 Gallus gallus finished cDNA, clone ChEST762h15 / Gallus gallus finished cDNA, clone ChEST762h15 / Finished cDNA, clone ChEST762h15 / --- / --- / --- / --- / --- / Gallus gallus finished cDNA, clone ChEST762h15 / Finished cDNA, clone ChEST762h15
RIGG13259	4.178	0.0493	ACTTCCATGCTATTGGCTGGTCAGGATGGATTATTTACCCAAGTGGATACAATGCATTTTACTGCAGAGG		ENSGALT00000010090.1 / --- / --- / --- / / --- / --- / --- / --- / --- / ---
RIGG17849	4.147	0.0467	TTAGTGTACATCGAAAAGTCACCAAACTACTGCGAGGAAGATGCTTCCACAGGAAGTGTTGGCACCCAGG	WNT-7B protein (Fragment). [Source:SPTREMBL;Acc:Q9PS88]	WNT-7B protein (Fragment). [Source:SPTREMBL / UP|Q3L254_CHICK (Q3L254) Wingless-type MMTV integration site family member 7b isoform 1, complete / Gallus gallus wingless-type MMTV integration site family member 7b isoform 2 (WNT7b) mRNA, complete cds, alternatively spliced / Wingless-type MMTV integration site family, member 7B (WNT7B), mRNA / WNT7b: Wingless-type MMTV integration site family member 7b isoform 1 / --- / --- / --- / --- / Gallus gallus wingless-type MMTV integration site family member 7b isoform 2 (WNT7b) mRNA, complete cds, alternatively spliced / Wingless-type MMTV integration site family, member 7B (WNT7B), mRNA
RIGG07791	4.141	0.00167	CCGCCACCAGCAGCACAAAATCTATACTTACACGGGATCTATCTTGGTAGCAGTGAATCCATACCAGCTG		ENSGALG00000001936.1 / --- / Gallus gallus myosin VI (MYO6), mRNA / Myosin VI (CMY6 gene) / / --- / --- / --- / myosin VIIB; / --- / ---

Gene Name	Fold Change	p-value	SEQUENCE	Array Description	Blast/Database Description
RIGG18176	4.123	0.02	TCTTGGACTTCAATCATTTCTATGGAATGCAGAAATACCATCACGAAAAGCTTGTCCAGATGCTCAAGGA		ENSGALT00000023959.1 / Gallus gallus finished cDNA, clone ChEST845f21 / gPGC_EST07264 Embryonic gonadal PGC cDNA Library Gallus gallus cDNA 5', mRNA sequence / Finished cDNA, clone ChEST845f21 / / PREDICTED: Gallus gallus similar to RIKEN cDNA B130016O10 gene (LOC427189), mRNA. / PREDICTED: similar to RIKEN cDNA B130016O10 gene; / --- / --- / gPGC_EST07264 Embryonic gonadal PGC cDNA Library Gallus gallus cDNA 5', mRNA sequence / Finished cDNA, clone ChEST845f21
RIGG00373	4.102	0.00164	CTTCACCTTGTCCAGAAGAAGCACCATGACTACTGCCTCTGAGTCCTGTTCTCTCTTATCAGCCTGGGAC	Gallus gallus mRNA for hypothetical protein, clone 1p14	Gallus gallus mRNA for hypothetical protein, clone 1p14 / RF|NP_001026363.1|71894855|NM_001031192 diacylglycerol kinase, zeta 104kDa {Gallus gallus} (exp=-1; wgp=0; cg=0), complete / Gallus gallus mRNA for hypothetical protein, clone 1p14 / Diacylglycerol kinase, zeta 104kDa (DGKZ), mRNA / RCJMB04_1p14: Hypothetical protein / --- / --- / Diacylglycerol kinase, zeta 104kDa (DGKZ), mRNA; / --- / Gallus gallus mRNA for hypothetical protein, clone 1p14 / Diacylglycerol kinase, zeta 104kDa (DGKZ), mRNA
RIGG14070	4.079	0.0134	CAGGCAGACAACAAGCTGGGATTTACCACTGGGTGACATTTACATTGAAGCAAACACACATAGTATGTATCTA		ENSGALT00000012464.1 / Gallus gallus finished cDNA, clone ChEST269h5 / Gallus gallus finished cDNA, clone ChEST269h5 / Finished cDNA, clone ChEST269h5 / --- / --- / --- / --- / --- / Gallus gallus finished cDNA, clone ChEST269h5 / Finished cDNA, clone ChEST269h5
RIGG00325	4.079	0.0107	GCATTCTGAAACTCCTATACCAATTATACTGAAGTGTAATAACTCCTTTGCAGCCAGTACTCCTGAGCTTCAGC	Gallus gallus mRNA for hypothetical protein, clone 30j20	Gallus gallus mRNA for hypothetical protein, clone 30j20 / --- / Gallus gallus mRNA for hypothetical protein, clone 30j20 / WD repeat and FYVE domain containing 1 (WDFY1), mRNA / RCJMB04_30j20: Hypothetical protein / PREDICTED: Gallus gallus similar to KIAA1435 protein (LOC424806), mRNA. / PREDICTED: similar to KIAA1435 protein; / WD repeat and FYVE domain containing 1 (WDFY1), mRNA; / --- / Gallus gallus mRNA for hypothetical protein, clone 30j20 / WD repeat and FYVE domain containing 1 (WDFY1), mRNA
RIGG17266	4.065	0.00546	AGTGCCATGTATACCTCTTGTATGTGTACCTCTTCTTTTCCTTCTCCTTTCTAATCCAGCCTTACAGCCC		ENSGALT00000021512.1 / Gallus gallus finished cDNA, clone ChEST490m16 / Gallus gallus mRNA for hypothetical protein, clone 24k6 / Similar to RIKEN cDNA 0610011N22 (LOC420970), mRNA / --- / PREDICTED: Gallus gallus similar to RIKEN cDNA 0610011N22 (LOC420970), mRNA. / PREDICTED: similar to RIKEN cDNA 0610011N22; / Similar to RIKEN cDNA 0610011N22 (LOC420970), mRNA; / --- / Gallus gallus mRNA for hypothetical protein, clone 24k6 / Similar to RIKEN cDNA 0610011N22 (LOC420970), mRNA
RIGG06790	4.043	0.00449	CAAATGAAATCCCCAGCAGTAGATCCCTCTTAAGTGTAACGCTGTGGTTTGATGAGCTATTCTTTGGGCA	Weakly similar to Q9D8U3 (Q9D8U3) 1810033M07Rik protein	Weakly similar to Q9D8U3 (Q9D8U3) 1810033M07Rik protein / Gallus gallus finished cDNA, clone ChEST941i20 / Gallus gallus finished cDNA, clone ChEST960f23 / Finished cDNA, clone ChEST942f22 / --- / PREDICTED: Gallus gallus similar to hypothetical protein FLJ32115 (LOC417942), mRNA. / PREDICTED: similar to hypothetical protein FLJ32115; / Finished cDNA, clone ChEST942f22; / --- / --- / ---

Gene Name	Fold Change	p-value	SEQUENCE	Array Description	Blast/Database Description
RIGG03661	3.997	0.0246	ATTATTTCTCAGCATTGCAGAGCTTTGCAAGAAGACCCCATCCATGCATACGTGTGGGAATGTCCTGCTG	Genome Hit Contig24.431	Genome Hit Contig24.431 / Gallus gallus finished cDNA, clone ChEST532f22 / Gallus gallus finished cDNA, clone ChEST532f22 / Finished cDNA, clone ChEST722a19 / --- / --- / --- / --- / --- / Gallus gallus finished cDNA, clone ChEST532f22 / Finished cDNA, clone ChEST722a19
RIGG15240	3.99	0.00789	TCACCTATGTTCAGTTTGCTAACGATGAATGTGACTATGGAATGGGATATGAACTGGGGATGGACCTCTT		ENSGALT00000015738.1 / UP|Q9DG21_CHICK (Q9DG21) POP1C protein, complete / Gallus gallus blood vessel epicardial substance (BVES), mRNA / BVES / --- / PREDICTED: Gallus gallus similar to hypothetical protein FLJ20534 (LOC422431), mRNA. / PREDICTED: similar to hypothetical protein FLJ20534; / --- / --- / Gallus gallus blood vessel epicardial substance (BVES), mRNA / BVES
RIGG00476	3.981	0.0211	CTCCCGGGTTGTCATTGTGTACAGTTCTGATCTGTTGTAGCTGTTGGCAGTATTAAAATGGTGAGTGCCA	Weakly similar to Q9CUN4 (Q9CUN4) 4930431J08Rik protein (Fragment)	Weakly similar to Q9CUN4 (Q9CUN4) 4930431J08Rik protein (Fragment) / Gallus gallus finished cDNA, clone ChEST1001a1 / Gallus gallus finished cDNA, clone ChEST1001a1 / Finished cDNA, clone ChEST631b23 / --- / PREDICTED: Gallus gallus similar to KIAA1571 protein (LOC424100), mRNA. / PREDICTED: similar to KIAA1571 protein; / Finished cDNA, clone ChEST631b23; / --- / Gallus gallus finished cDNA, clone ChEST1001a1 / Finished cDNA, clone ChEST631b23
RIGG08708	3.963	0.0273	GCCATTTCAATGACAATGGACAGTGTTCAGAGCATCTTCTCCTACTATGACAACTCGATGATACTCATTGGAAAT		ENSGALG00000009289.1 / --- / Gallus gallus vesicular acetylcholine transporter mRNA, complete cds / Solute carrier family 18 (vesicular acetylcholine), member 3 (SLC18A3), mRNA / Vesicular monoamine transporter (Fragment) / --- / --- / Vesicular monoamine transporter; / --- / --- / ---
RIGG12148	3.963	0.0223	GACGTTGTGTGGTATTGACAATGTAGCCTAAAGTAGACCTTACATACAGAAGCTCTTTTGATCTTCGGGC		ENSGALT00000006794.1 / RF|NP_001006532.1|57529679|NM_001006532 Ral GEF with PH domain and SH3 binding motif 2 {Gallus gallus} (exp=-1; wgp=0; cg=0), complete / Gallus gallus mRNA for hypothetical protein, clone 17i24 / Ral GEF with PH domain and SH3 binding motif 2 (RALGPS2), mRNA / RCJMB04_17i24: Hypothetical protein / PREDICTED: Gallus gallus similar to Ral-A exchange factor RalGPS2 (LOC424429), mRNA. / PREDICTED: similar to Ral-A exchange factor RalGPS2; / Ral GEF with PH domain and SH3 binding motif 2 (RALGPS2), mRNA; / --- / Gallus gallus mRNA for hypothetical protein, clone 17i24 / Ral GEF with PH domain and SH3 binding motif 2 (RALGPS2), mRNA
RIGG15745	3.953	0.0108	TGCATTGCAACAGTACCATCTGGCCTCTGTTTGTCATTACTTTGTCATACCCTCACTCCGTCTGCCCATC		ENSGALT00000017084.1 / Gallus gallus finished cDNA, clone ChEST269g13 / Gallus gallus finished cDNA, clone ChEST269g13 / Finished cDNA, clone ChEST269g13 / --- / --- / --- / --- / --- / Gallus gallus finished cDNA, clone ChEST269g13 / Finished cDNA, clone ChEST269g13

Gene Name	Fold Change	p-value	SEQUENCE	Array Description	Blast/Database Description
RIGG16439	3.94	0.0281	CCTACATCTCTGAATGTCTTAGGCCAAGGATCTGTCTTCAATTTCAAAGGCACCATAGAAAATCCCTCATT		ENSGALT00000019130.1 / acyl-Coenzyme A dehydrogenase family, member 11 [Gallus gallus] / Gallus gallus mRNA for hypothetical protein, clone 33j3 / Acyl-Coenzyme A dehydrogenase family, member 11 (ACAD11), mRNA / ACAD11, RCJMB04_33j3: Acyl-CoA dehydrogenase family member 11 / --- / PREDICTED: similar to putative acyl-CoA dehydrogenase; / --- / --- / Gallus gallus mRNA for hypothetical protein, clone 33j3 / Acyl-Coenzyme A dehydrogenase family, member 11 (ACAD11), mRNA
RIGG20364	3.892	0.0243	GACTCGAAGCCGTAGCAGTGACAAAGTCGTGCTGGTGTCTAATTATACTCAGACATTGGATCTCTTTGAA	Mus musculus RAD54 like (S. cerevisiae) (Rad54l), mRNA	Mus musculus RAD54 like (S. cerevisiae) (Rad54l), mRNA / --- / Gallus gallus putative recombination factor (GdRAD54) mRNA, partial cds / Putative recombination factor (GdRAD54) / RAD54L, RAD54: DNA repair and recombination protein RAD54-like (Fragment) / PREDICTED: Gallus gallus similar to putative recombination factor GdRad54 (LOC424611), mRNA. / PREDICTED: similar to putative recombination factor GdRad54; / Putative recombination factor (GdRAD54); / --- / --- / ---
RIGG04551	3.827	0.0402	GATTAGCAGTTACGACCTCTCGATTACCAGTTGCCCCGATCGCGATAAGCTCTGTCTTTCTCCATAGCCC	Similar to Q8JFX5 (Q8JFX5) Hypothetical protein	Similar to Q8JFX5 (Q8JFX5) Hypothetical protein / Gallus gallus finished cDNA, clone ChEST642o5 / 603211360F1 CSEQRBN13 Gallus gallus cDNA clone ChEST191g15 5', mRNA sequence / Peptidyl-tRNA hydrolase 2 (PTRH2), mRNA / --- / PREDICTED: Gallus gallus similar to Peptidyl-tRNA hydrolase 2, mitochondrial precursor (PTH 2) (Bcl-2 inhibitor of transcription) (CGI-147) (LOC417634), mRNA. / PREDICTED: similar to Peptidyl-tRNA hydrolase 2, mitochondrial precursor (PTH 2) (Bcl-2 inhibitor of transcription) (CGI-147); / Peptidyl-tRNA hydrolase 2 (PTRH2), mRNA; / --- / --- / ---
RIGG12255	3.798	0.012	ACAAGAATGGAAAACTCCTCCCAAGCACCCGTACCTGGAACTCTGTTCTGTCTTTACCTATGGGA		ENSGALT00000007098.1 / Gallus gallus finished cDNA, clone ChEST51j13 / Gallus gallus finished cDNA, clone ChEST51j13 / Finished cDNA, clone ChEST51j13 / Winged helix protein CWH-5 (Fragment) / --- / --- / Finished cDNA, clone ChEST51j13;Winged helix protein CWH-5; / --- / Gallus gallus finished cDNA, clone ChEST51j13 / Finished cDNA, clone ChEST51j13
RIGG19081	3.719	0.00633	CAAACAAGCTGACATGTGGCAAACACGTCTTAGTGCACACTTGGGTAAAGCTATTGGAGTGACCGTTGGC		ENSGALT00000026355.1 / homologue to UP|Q810S8_MOUSE (Q810S8) Tmem65 protein, partial (96%) / Gallus gallus mRNA for hypothetical protein, clone 8k18 / Similar to 4930438D12Rik protein (LOC428387), mRNA / --- / --- / --- / Similar to 4930438D12Rik protein (LOC428387), mRNA; / --- / Gallus gallus mRNA for hypothetical protein, clone 8k18 / Similar to 4930438D12Rik protein (LOC428387), mRNA

Gene Name	Fold Change	p-value	SEQUENCE	Array Description	Blast/Database Description
RIGG02558	3.717	0.00114	GTGTGAGCAGTTTACTGATGAAGTCAAGATGCTCTTGGAAAACTACAACAAGACAACTCTGCTTCTCTCCA	Similar to O75935 (O75935) Dynactin subunit	Similar to O75935 (O75935) Dynactin subunit / GB|BX932189.2|BX932189.2 Gallus gallus finished cDNA, clone ChEST361h9 / gPGC_EST03378 Embryonic gonadal PGC cDNA Library Gallus gallus cDNA 5', mRNA sequence / AT rich interactive domain 3C (BRIGHT- like) (ARID3C), mRNA / --- / --- / --- / --- / --- / gPGC_EST03378 Embryonic gonadal PGC cDNA Library Gallus gallus cDNA 5', mRNA sequence / AT rich interactive domain 3C (BRIGHT- like) (ARID3C), mRNA
RIGG02905	3.711	0.00269	AGCACCGTTCTTGCTGTTGATAACTTCAATGGGATCAAGATCAAAGGAAGGACGATCCGAGTGGACCACG	Similar to Q8R0F5 (Q8R0F5) Similar to CGI-79 protein (Similar to RNA-b	Similar to Q8R0F5 (Q8R0F5) Similar to CGI-79 protein (Similar to RNA-b / Gallus gallus finished cDNA, clone ChEST403o4 / Gallus gallus finished cDNA, clone ChEST745i13 / Finished cDNA, clone ChEST964m23 / / --- / --- / --- / --- / Gallus gallus finished cDNA, clone ChEST745i13 / Finished cDNA, clone ChEST964m23
RIGG17508	3.704	0.0113	ATGTCTTGCATCCTATCAGCCTTTGGGATCTGCATCACTACAGTTGGAATGAAATGCACAAAGTTGGGAG		ENSGALT00000022197.1 / --- / Gallus gallus finished cDNA, clone ChEST148c4 / Finished cDNA, clone ChEST148c4 / / --- / --- / --- / claudin 20; / --- / ---
RIGG18296	3.697	0.0265	GGACCTCTGTCTCCATACACAATTGAGTTTCTGCGACACCTGAGAAGCTTCTTCCAGATTATGTTTAAAA		ENSGALT00000024230.1 / Gallus gallus finished cDNA, clone ChEST862h18 / Gallus gallus finished cDNA, clone ChEST862h18 / Finished cDNA, clone ChEST862h18 / / PREDICTED: Gallus gallus similar to RNA 3-terminal phosphate cyclase-like protein (LOC427222), mRNA. / PREDICTED: similar to RNA 3-terminal phosphate cyclase-like protein; / Finished cDNA, clone ChEST862h18; / --- / Gallus gallus finished cDNA, clone ChEST862h18 / Finished cDNA, clone ChEST862h18
RIGG14088	3.688	0.0229	GAGCTTTCCATCCTACGCAGAATTCGTCACCCCAACATTGTGCACATCTTTGAGCTCATTGAGGT		ENSGALT00000012525.1 / --- / testis_EST00117 Testis cDNA Library Gallus gallus cDNA 3', mRNA sequence / Transcribed locus, weakly similar to NP_001024018.1 abnormal embryonic PARtitioning of cytoplasm family member (par-1) [Caenorhabditis elegans] / similar to serine/threonine kinase FKSG82 / PREDICTED: Gallus gallus similar to serine/threonine protein kinase SSTK (LOC429001), mRNA. / PREDICTED: similar to serine/threonine protein kinase SSTK; / Transcribed locus, weakly similar to NP_001024018.1 abnormal embryonic PARtitioning of cytoplasm family member (par-1) [Caenorhabditis elegans]; / --- / testis_EST00117 Testis cDNA Library Gallus gallus cDNA 3', mRNA sequence / Transcribed locus, weakly similar to NP_001024018.1 abnormal embryonic PARtitioning of cytoplasm family member (par-1) [Caenorhabditis elegans]

Gene Name	Fold Change	p-value	SEQUENCE	Array Description	Blast/Database Description
RIGG10423	3.673	0.0332	CACTGTTCCCTCCTCGTCTTTTGTA GCAGCTGTTTTGCAACTCAGTGCAA TGGAAAGATCGCATGGGTTG		ENSGALT00000001933.1 / Gallus gallus finished cDNA, clone ChEST1014b1 / Gallus gallus finished cDNA, clone ChEST1014b1 / Finished cDNA, clone ChEST1014b1 / similar to Chain A, Crystal Structure Of Soluble Form Of Clic4 / --- / --- / --- / --- / Gallus gallus finished cDNA, clone ChEST1014b1 / Finished cDNA, clone ChEST1014b1
RIGG03537	3.652	0.0299	GCTTTGACTTCAGCTTGCTACTGTG AAGATTTTGTTCCAGAGCTTATATGA CTTACATAACACACCAGCTGC	Similar to O75449 (O75449) P60 katanin	Similar to O75449 (O75449) P60 katanin / Gallus gallus finished cDNA, clone ChEST50p6 / Gallus gallus p60 katanin mRNA, complete cds / P60 katanin / / PREDICTED: Gallus gallus similar to katanin p60 subunit A 1 (LOC421626), mRNA. / PREDICTED: similar to katanin p60 subunit A 1; / P60 katanin; / --- / --- / ---
RIGG16817	3.639	0.0208	GCATATTACAGGCATTCCTCATTCTC CCACCGGACAAGCTATCACTGAGCG TACTCACCAAACATTGAAA		ENSGALT00000020203.1 / weakly similar to UP|POK17_HUMAN (P63136) HERV-K_11q22.1 provirus ancestral Pol protein [Includes: Reverse transcriptase (RT) ; Ribonuclease H (RNase H); Integrase (IN)] , partial (9%) / Gallus gallus finished cDNA, clone ChEST272k18 / Finished cDNA, clone ChEST272k18 / / --- / --- / --- / --- / --- / ---
RIGG02904	3.628	0.0285	TCCTGACCAGTCTCTCCATTTGGAT TTTCAGTGCACTGTTAATATGTTGTA GTACTTTCTTCCCTCCTCTTTCTT	Weakly similar to Q8K2V8 (Q8K2V8) Similar to lipopolysaccharide specific resp	Weakly similar to Q8K2V8 (Q8K2V8) Similar to lipopolysaccharide specific resp / Gallus gallus finished cDNA, clone ChEST403g18 / Gallus gallus finished cDNA, clone ChEST403g18 / Finished cDNA, clone ChEST403g18 / --- / --- / --- / --- / --- / Gallus gallus finished cDNA, clone ChEST403g18 / Finished cDNA, clone ChEST403g18
RIGG00731	3.599	0.017	TTGTATTCTAAGGCGAGGTTGGATA TGGGGAGAAGAAAGTAGTCTGGTGC AGTTTTGTGGTTCCTCATTCCTG	Similar to Q8CIA2 (Q8CIA2) Similar to mucin	Similar to Q8CIA2 (Q8CIA2) Similar to mucin / Gallus gallus finished cDNA, clone ChEST1021o18 / gPGC_EST00035 Embryonic gonadal PGC cDNA Library Gallus gallus cDNA 5', mRNA sequence / Finished cDNA, clone ChEST1021o18 / --- / --- / --- / Transcribed locus, strongly similar to XP_421035.1 PREDICTED: similar to Mucin 2 precursor (Intestinal mucin 2) [Gallus gallus]; / --- / --- / ---
RIGG00599	3.592	0.018	ACCCTCTCGCCCCACTGGTGAATAT TCTGTAACAATTTGTACAAAAGGTAA GATGGAAATGTGCTGTTAT	Genome Hit Contig2.401	Genome Hit Contig2.401 / similar to UP|Q69607_HBV (Q69607) Protein x, partial (8%) / Gallus gallus finished cDNA, clone ChEST100n9 / Finished cDNA, clone ChEST100n9 / --- / --- / --- / --- / --- / Gallus gallus finished cDNA, clone ChEST100n9 / Finished cDNA, clone ChEST100n9
RIGG18319	3.588	0.00667	CCTCCCTGTAAAGTTCTAGTATTTG GCCCTCCAGTGTCAGGCAGAACAAC TATTTGTAACCTAATTGCAA		ENSGALT00000024315.1 / --- / --- / --- / / --- / --- / --- / chromosome 6 open reading frame 199; / --- / ---
RIGG19259	3.561	0.0484	GTGTGCTCTTACCAGGTGGCTCAGA TTGCGCTCCTCTATGGCTGTCCATA TTCACATTCTCTTCATCTGC		ENSGALT00000026887.1 / similar to UP|CD99_HUMAN (P14209) CD99 antigen precursor (T-cell surface glycoprotein E2) (E2 antigen) (MIC2 protein) (12E7), partial (11%) / Gallus gallus finished cDNA, clone ChEST978c20 / Finished cDNA, clone ChEST424i22 / --- / --- / --- / --- / --- / Gallus gallus finished cDNA, clone ChEST978c20 / Finished cDNA, clone ChEST424i22

Gene Name	Fold Change	p-value	SEQUENCE	Array Description	Blast/Database Description
RIGG00794	3.539	0.0263	GATGGTACGAGGAGATGAAGGCTTATTATTCCTTATCACTGCATGCAGAAAGCGTAGTTCAGTCATTCAGGTG	Partial Contig Hit 350255.1	Partial Contig Hit 350255.1 / Gallus gallus finished cDNA, clone ChEST1027a3 / Gallus gallus vav 3 oncogene (VAV3), mRNA / GDP/GTP exchange factor VAV3 (VAV3) / --- / Gallus gallus vav 3 oncogene (VAV3), mRNA. / vav 3 oncogene; / GDP/GTP exchange factor VAV3 (VAV3); / --- / Gallus gallus vav 3 oncogene (VAV3), mRNA / GDP/GTP exchange factor VAV3 (VAV3)
RIGG02247	3.528	0.035	CCCGACAGAGTCTATTCTTGTACTGACTGCACAACAACTGCCGGTCTTTTCCAATAAACTTTCTAACCCC	Weakly similar to GA45_HUMAN (P24522) Growth arrest and DNA-damage-inducible	Weakly similar to GA45_HUMAN (P24522) Growth arrest and DNA-damage-inducible / Gallus gallus finished cDNA, clone ChEST318g14 / Gallus gallus finished cDNA, clone ChEST740k19 / Finished cDNA, clone ChEST560m2 / --- / --- / --- / --- / --- / Gallus gallus finished cDNA, clone ChEST740k19 / Finished cDNA, clone ChEST560m2
RIGG05752	3.515	0.0223	TGGCATCTCTGAAGGCTTTGTGTTAACTTGCGTACTGATTGTTCCAGATGTTCCCCAGATGTCAAAGGGA	Genome Hit Contig37.32	Genome Hit Contig37.32 / --- / Gallus gallus finished cDNA, clone ChEST797p14 / Finished cDNA, clone ChEST797p14 / --- / --- / --- / --- / --- / Gallus gallus finished cDNA, clone ChEST797p14 / Finished cDNA, clone ChEST797p14
RIGG19146	3.506	0.00608	CCAGAAAGCCATCTGAATGTCCTAGGATTTACATATCTGTGTTCTGACTGATCTTCTGTTTACTGCAGACCAC	bHLH transcription factor [Gallus gallus]. [Source:RefSeq;Acc:NM_204684]	bHLH transcription factor [Gallus gallus]. [Source:RefSeq / UP|Q9DEQ9_CHICK (Q9DEQ9) BHLH transcription factor, complete / Gallus gallus bHLH transcription factor (MESPO), mRNA / BHLH transcription factor (MESPO gene) / MESPO: BHLH transcription factor / Gallus gallus bHLH transcription factor (MESPO), mRNA. / bHLH transcription factor; / BHLH transcription factor (MESPO gene); / --- / Gallus gallus bHLH transcription factor (MESPO), mRNA / BHLH transcription factor (MESPO gene)
RIGG07068	3.465	0.0271	TGAATAGGTGCTCGCCATAACGCTCTCTTGAAGGTGTCCCTCTTCTAGAAGGGTAATTAATGCAGATTTCTTCA	Partial Contig Hit 507545.1	Partial Contig Hit 507545.1 / Gallus gallus finished cDNA, clone ChEST982b15 / Gallus gallus finished cDNA, clone ChEST982b15 / Finished cDNA, clone ChEST982b15 / --- / --- / --- / --- / --- / Gallus gallus finished cDNA, clone ChEST982b15 / Finished cDNA, clone ChEST982b15
RIGG12728	3.458	0.0316	TCAGTGCTACTGCTACATTTGTGATAAGCTAGCTTCTGAGTGCCAGATCTGGACAACTTCATCCCTCTGTC		ENSGALT00000008480.1 / --- / 603110092F1 CSEQCHL12 Gallus gallus cDNA clone ChEST55g24 5', mRNA sequence / Transcribed locus, moderately similar to XP_429439.1 PREDICTED: hypothetical protein XP_429439 [Gallus gallus] / --- / PREDICTED: Gallus gallus hypothetical gene supported by CR386965 (LOC416530), mRNA. / PREDICTED: hypothetical protein XP_429439; / --- / --- / 603110092F1 CSEQCHL12 Gallus gallus cDNA clone ChEST55g24 5', mRNA sequence / Transcribed locus, moderately similar to XP_429439.1 PREDICTED: hypothetical protein XP_429439 [Gallus gallus]
RIGG14256	3.442	0.023	TGCCCTCTCCATGTCTCCAAACAGAACCAGTTACTTTATTCCAATTTTATAGCTACCTGGAGAAGCACCAG		ENSGALT00000012999.1 / --- / --- / --- / --- / --- / --- / --- / --- / --- / ---

Gene Name	Fold Change	p-value	SEQUENCE	Array Description	Blast/Database Description
RIGG02081	3.439	0.0436	TCTTCGTCCGCCTTAAGTCCTTGAG TTACTTTGTCTTCCTTCTTTTATTTTG TCTAAAAGCCAGTTCCAAGC	Weakly similar to TMF1_HUMAN (P82094) TATA element modulatory factor (TMF)	Weakly similar to TMF1_HUMAN (P82094) TATA element modulatory factor (TMF) / Gallus gallus finished cDNA, clone ChEST281p15 / --- / --- / --- / --- / --- / --- / --- / 603373881F1 CSEQRBN20 Gallus gallus cDNA clone ChEST284b21 5', mRNA sequence / Finished cDNA, clone ChEST281p15
RIGG09912	3.401	0.0024	ACATTATCACCGTTTCTGCAAGTTTC ATGCAAAACTTAAGCCATTTCTGCAT GCAACACGATGCTGGTCG		ENSGALT00000000508.1 / Gallus gallus finished cDNA, clone ChEST740b21 / Gallus gallus finished cDNA, clone ChEST394c10 / Finished cDNA, clone ChEST527i9 / --- / --- / --- / --- / --- / Gallus gallus finished cDNA, clone ChEST394c10 / Finished cDNA, clone ChEST527i9
RIGG14022	3.401	0.00462	TTCGAAGAACAAACTGTTCATTCTGA GGGATTGTCTGGATTCTGCTGGAAG TTTCCTCCTGCTCCTTGTG		ENSGALT00000012310.1 / --- / --- / --- / --- / --- / --- / --- / --- / --- / ---
RIGG00664	3.399	0.00112	CCACGCGCCCTGTTTTAACTGTTCC GAGTAAAGTTTAGTGAATGAGAATTT GTTGATACACAGCTGTGGGA	Genome Hit Contig70.43	Genome Hit Contig70.43 / --- / Gallus gallus finished cDNA, clone ChEST1015a13 / Finished cDNA, clone ChEST1015a13 / --- / --- / --- / --- / --- / Gallus gallus finished cDNA, clone ChEST1015a13 / Finished cDNA, clone ChEST1015a13
RIGG00259	3.388	0.0216	CCCTATAAATTCCCCAATATTTCCTG CCTCCTATTCTGTTCCAGTTTTATCA TTGGTCTTTGGGTGCTGAG	Gallus gallus mRNA for hypothetical protein, clone 19p13	Gallus gallus mRNA for hypothetical protein, clone 19p13 / homologue to GB|CAG33735.1|50871722|AJ745097 immunoglobulin-like receptor CHIR-AB1 precursor {Gallus gallus} (exp=-1; wgp=0; cg=0), complete / Gallus gallus mRNA for hypothetical protein, clone 19p13 / Similar to immunoglobulin-like receptor CHIR-B2 precursor (LOC425113), mRNA / --- / --- / --- / --- / --- / Gallus gallus mRNA for hypothetical protein, clone 19p13 / Similar to immunoglobulin-like receptor CHIR-B2 precursor (LOC425113), mRNA
RIGG00600	3.368	0.0266	TGACATAGCGATCAGACTGGTGGCG GATGAACTTCTTGGTCCTCTTCTTAA CGATTTTAGGCTTCACGAG	Similar to RL3P_MOUSE (P17932) 60S ribosomal protein L32'	Similar to RL3P_MOUSE (P17932) 60S ribosomal protein L32' / homologue to UP|Q63ZV8_RAT (Q63ZV8) Ribosomal protein L32, complete / gPGC_EST02775 Embryonic gonadal PGC cDNA Library Gallus gallus cDNA 5', mRNA sequence / Finished cDNA, clone ChEST753b6 / / --- / --- / --- / --- / gPGC_EST02775 Embryonic gonadal PGC cDNA Library Gallus gallus cDNA 5', mRNA sequence / Finished cDNA, clone ChEST753b6
RIGG16797	3.365	0.0249	GCTCGAAGTTTAGCTGATGTTGCTA GAGAATATGGCTCTTCCTCCCAGTC CTTTCTAACAGAAACCAACT		ENSGALT00000020164.1 / Gallus gallus finished cDNA, clone ChEST370e1 / --- / --- / / --- / --- / --- / salvador homolog 1 (Drosophila); / --- / ---
RIGG07674	3.358	0.00142	AGGACCACCAAGGATGAGCTCACAC TACAAACCCTGACTTTTATTGGATGT GGAGTTTCCTTCTGTGCCC		ENSGALG00000001094.1 / --- / Gallus gallus finished cDNA, clone ChEST653l18 / Finished cDNA, clone ChEST653l18 / / --- / --- / --- / --- / --- / ---

Gene Name	Fold Change	p-value	SEQUENCE	Array Description	Blast/Database Description
RIGG14606	3.348	0.0344	TCAGCCGGTTCTTCGAACCCAACCAAACCAACGAGTTCCTCAATGCCATCTACATTTTGATAAGCAACAT		ENSGALT00000014051.1 / similar to UP|Q5BKJ7_XENTR (Q5BKJ7) MGC108282 protein, partial (39%) / naw36h09.y1 Chicken eye (hatched). Unnormalized (naw) Gallus gallus cDNA clone naw36h09 5', mRNA sequence / Transcribed locus, weakly similar to XP_528518.1 PREDICTED: similar to hypothetical protein FLJ20245 [Pan troglodytes] / hypothetical protein / PREDICTED: Gallus gallus similar to hypothetical protein FLJ20245 (LOC417257), mRNA. / PREDICTED: similar to hypothetical protein FLJ20245; / Transcribed locus, weakly similar to XP_528518.1 PREDICTED: similar to hypothetical protein FLJ20245 [Pan troglodytes]; / --- / --- / ---
RIGG04014	3.331	0.0441	AACGCTTTGTTTCACTCTGTCATGTCTTAAGTATGGCTGGTTCTTGCAAATCCACTCACTCTGTATTCAT	Genome Hit Contig21370.1	Genome Hit Contig21370.1 / --- / gonad_EST09021 Embryonic gonad cDNA Library Gallus gallus cDNA 5', mRNA sequence / Finished cDNA, clone ChEST573e22 / --- / --- / --- / --- / --- / --- / ---
RIGG01005	3.32	0.0209	ACTGTAGAGATTCCAGTAGTAAAGGCAGTAAGGAAAACAGCATTCTAAACCACAGTAACTTTGAGGCAGACAC	Contig Hit 035501.1	Contig Hit 035501.1 / Gallus gallus finished cDNA, clone ChEST129j17 / Gallus gallus finished cDNA, clone ChEST129j17 / Finished cDNA, clone ChEST189o19 / --- / --- / --- / --- / --- / Gallus gallus finished cDNA, clone ChEST129j17 / Finished cDNA, clone ChEST189o19
RIGG07415	3.317	0.0302	CTGCCTCTCATCATTACAACAGCAGTGAGCATAACATTCCTTTGCACTTTGTGTATATCCACTATTCTGTGCT		Contig_234_reverse / --- / gonad_EST04073 Embryonic gonad cDNA Library Gallus gallus cDNA 5', mRNA sequence / Transcribed locus / --- / --- / --- / --- / --- / gonad_EST04073 Embryonic gonad cDNA Library Gallus gallus cDNA 5', mRNA sequence / Transcribed locus
RIGG02885	3.295	0.0402	CCTATTGCTGCCACCTCACGGATGTTGTTAGTGATTGTTTGCATCTCTCTCCTTATCGCTGCTTCAGAAA	Partial Contig Hit 003845.1	Partial Contig Hit 003845.1 / Gallus gallus finished cDNA, clone ChEST3g3 / Gallus gallus finished cDNA, clone ChEST3g3 / Finished cDNA, clone ChEST3g3 / --- / --- / --- / --- / --- / Gallus gallus finished cDNA, clone ChEST3g3 / Finished cDNA, clone ChEST3g3
RIGG02909	3.285	0.00103	ACCACAAAGATCCCGACAAGCCGTATATGTTTACCCTTTCCATAAATGAACAGGGAGATTATGAAGGTAT	RIKENRP Weakly similar to PC8422	RIKENRP Weakly similar to PC8422 / Gallus gallus finished cDNA, clone ChEST404o7 / Gallus gallus finished cDNA, clone ChEST404o7 / Finished cDNA, clone ChEST404o7 / --- / --- / --- / --- / --- / Gallus gallus finished cDNA, clone ChEST404o7 / Finished cDNA, clone ChEST404o7
RIGG10049	3.283	0.00629	AAGACCCTCACATCCTGCCTCGATGCCGCTGGAAAATTCTCTGGTTTAACCAATATCGCCCATTATACGG		ENSGALT00000000875.1 / --- / --- / --- / --- / --- / --- / --- / --- / --- / ---
RIGG15263	3.28	0.00789	GGTTCAACTCACTGTGTGTGGTGGAAGGCACGCCAAAGATGTTTGAAGCAGGTGGATACTGTGAAGTGGCA		ENSGALT00000015816.1 / --- / 603761248F1 CSEQRBN21 Gallus gallus cDNA clone ChEST680o11 5', mRNA sequence / Transcribed locus, strongly similar to XP_429958.1 PREDICTED: hypothetical protein XP_429958 [Gallus gallus] / --- / --- / --- / --- / --- / 603761248F1 CSEQRBN21 Gallus gallus cDNA clone ChEST680o11 5', mRNA sequence / Transcribed locus, strongly similar to XP_429958.1 PREDICTED: hypothetical protein XP_429958 [Gallus gallus]

Gene Name	Fold Change	p-value	SEQUENCE	Array Description	Blast/Database Description
RIGG02569	3.264	0.042	TGGCATCAGAGGCGGCTTTTGTGGAGGATCTGGATGAATCGTTTAAAGAAAACCGTAAAGATGATATTTGGCTTG	Weakly similar to PDA4_RAT (P38659) Protein disulfide isomerase A4 precursor	Weakly similar to PDA4_RAT (P38659) Protein disulfide isomerase A4 precursor / Gallus gallus finished cDNA, clone ChEST362f16 / 603147720F1 CSEQCHL18 Gallus gallus cDNA clone ChEST149h6 5', mRNA sequence / Finished cDNA, clone ChEST362f16 / --- / PREDICTED: Gallus gallus similar to hypothetical protein FLJ20013 (LOC417510), mRNA. / PREDICTED: similar to hypothetical protein FLJ20013; / --- / --- / gPGC_EST06367 Embryonic gonadal PGC cDNA Library Gallus gallus cDNA 5', mRNA sequence / Transcribed locus, weakly similar to NP_938037.1 thioredoxin domain containing 10 [Mus musculus]
RIGG04508	3.243	0.00252	GCTTGTTCTGCTTCAGTCTGAATGCCCCTAAACCTCCGAGTCACCCTCAAAACTCCTTTCTTTTACTGTT	Similar to Q9CSK6 (Q9CSK6) 2310033P09Rik protein (Fragment)	Similar to Q9CSK6 (Q9CSK6) 2310033P09Rik protein (Fragment) / Gallus gallus finished cDNA, clone ChEST637c11 / Gallus gallus finished cDNA, clone ChEST637c11 / Finished cDNA, clone ChEST637c11 / --- / --- / --- / --- / --- / Gallus gallus finished cDNA, clone ChEST637c11 / Finished cDNA, clone ChEST637c11
RIGG11322	3.242	0.031	CAAGCCTATGCCATTGCCTCCTCCATCATCTCCTTCTACCTGCCTCTTGTGGTCATGGTATTTGTGTACG		ENSGALT00000004441.1 / similar to GB|AAA89068.1|1199929|RATMTA beta-2 adrenergic receptor {Rattus norvegicus} (exp=-1; wgp=0; cg=0), partial (76%) / 603219508F1 CSEQRBN10 Gallus gallus cDNA clone ChEST211p2 5', mRNA sequence / Transcribed locus, moderately similar to XP_425195.1 PREDICTED: similar to beta-2 adrenergic receptor, partial [Gallus gallus] / similar to beta-2 adrenergic receptor / PREDICTED: Gallus gallus similar to beta-2 adrenergic receptor (LOC427623), partial mRNA. / PREDICTED: similar to beta-2 adrenergic receptor, partial; / --- / --- / --- / ---
RIGG04692	3.241	0.0394	AAAATGAATCCCTGATGTTGAGAACCTCTTCTCCATCACCTGTGAAACACACAAACTCTCCCCACTTCCAGTT	Same gene AF239837; Gallus gallus nidogen 1 mRNA, partial cds	Same gene AF239837; Gallus gallus nidogen 1 mRNA, partial cds / Gallus gallus finished cDNA, clone ChEST660e3 / Gallus gallus finished cDNA, clone ChEST660e3 / Finished cDNA, clone ChEST660e3 / Nidogen 1 (Fragment) / --- / --- / --- / --- / Gallus gallus finished cDNA, clone ChEST660e3 / Finished cDNA, clone ChEST660e3
RIGG14112	3.234	0.0364	AACTCCGAACAGTCACGCTTCTAATTCCAAATCCACACGCAACATCCCTCGAAGACATACAGTAGGTGGC		ENSGALT00000012596.1 / --- / --- / --- / --- / --- / --- / --- / KIAA1217; / --- / ---
RIGG04987	3.227	0.0337	GATAGGAAATCTCTGATAATGGGCATCACTGATCCTAGCCTTCTTGTTTTGTTCAGCCATCCATCCGACC	Genome Hit Contig1.750	Genome Hit Contig1.750 / similar to UP|Q5KMC4_CRYNE (Q5KMC4) D-tyrosyl-tRNA(Tyr) deacylase, partial (7%) / Gallus gallus finished cDNA, clone ChEST698b24 / Finished cDNA, clone ChEST698b24 / --- / --- / --- / --- / --- / Gallus gallus finished cDNA, clone ChEST698b24 / Finished cDNA, clone ChEST698b24

Gene Name	Fold Change	p-value	SEQUENCE	Array Description	Blast/Database Description
RIGG14894	3.225	0.0364	AAGCTCAGTCTTTCAGGAACCGAAGATCCTCCGGCTGTGTGTGAGATTTCTCCATTAGTATCTTACTTTGG		ENSGALT00000014800.1 / similar to UP|Q28CH3_XENTR (Q28CH3) UDP-N-acteylglucosamine pyrophosphorylase 1-like 1, partial (48%) / --- / --- / similar to UDP-N-acteylglucosamine pyrophosphorylase 1-like 1 / --- / --- / --- / UDP-N-acteylglucosamine pyrophosphorylase 1-like 1; / --- / ---
RIGG03751	3.211	0.00527	AGTGTTTAATGACAGGCCTGATTTTGATACTTAGTACTGAGTCCCTGTTCCCTATCTTTGAGTCAGCCATAA	Weakly similar to Q96NN4 (Q96NN4) Hypothetical protein FLJ30499	Weakly similar to Q96NN4 (Q96NN4) Hypothetical protein FLJ30499 / Gallus gallus finished cDNA, clone ChEST540n11 / Gallus gallus finished cDNA, clone ChEST540n11 / Finished cDNA, clone ChEST540n11 / / --- / --- / Finished cDNA, clone ChEST544n23; / solute carrier family 39 (zinc transporter), member 12; / Gallus gallus finished cDNA, clone ChEST540n11 / Finished cDNA, clone ChEST540n11
RIGG20045	3.194	0.0146	GGCTCACAACATGACAATTAGACAAAGTGTATGAAAGGCCAATCCCTCTGCTATTTCATTCTCTCCTCTTG	Gallus gallus beta-defensin 4 (GAL4) gene, complete cds	Gallus gallus beta-defensin 4 (GAL4) gene, complete cds / --- / --- / --- / --- / --- / --- / --- / --- / --- / ---
RIGG03806	3.189	0.0369	CTCTACTTCATAGCTCTGGCGATTGGAACTCTCTACTCCAACGCCCTCTTCCAGCTCATTCCCGA	Similar to Q96BB3 (Q96BB3) Hypothetical protein	Similar to Q96BB3 (Q96BB3) Hypothetical protein / Gallus gallus finished cDNA, clone ChEST547g13 / 603587952F1 CSEQCHN74 Gallus gallus cDNA clone ChEST547g13 5', mRNA sequence / Finished cDNA, clone ChEST547g13 / --- / --- / --- / --- / --- / --- / ---
RIGG12679	3.185	0.0451	ATCCTGAAGGGTCTTCTATTCTTGCACACGAGAACTCCACCAATAATTCACAGAGACTTAAAATGTGACA		ENSGALT00000008375.1 / --- / --- / --- / similar to mitogen-activated protein kinase kinase kinase / --- / --- / Transcribed locus, moderately similar to XP_520699.1 PREDICTED: similar to lysine-deficient protein kinase 2; mitogen-activated protein kinase kinase kinase; serologically defined colon cancer antigen 43 [Pan troglodytes]; / WNK lysine deficient protein kinase 2; / --- / ---
RIGG12681	3.171	0.0205	TGCCCGAGGAGTATTTCAGTACAGTTTTGGCTTAATACCTTTCAGGCAGCCGATTCATACTGTTGTGGGA		ENSGALT00000008383.1 / similar to UP|Q7ZXC3_XENLA (Q7ZXC3) Dgat2l1-prov protein, partial (89%) / 4039165 1GAL - Chicken Intestinal Lymphocyte Gallus gallus cDNA clone 1GAL_10M24 5', mRNA sequence / Transcribed locus, moderately similar to NP_079374.2 monoacylglycerol O-acyltransferase 2 [Homo sapiens] / --- / PREDICTED: Gallus gallus similar to Dgat2l1-prov protein (LOC424811), mRNA. / PREDICTED: similar to Dgat2l1-prov protein; / --- / --- / --- / ---
RIGG06616	3.164	0.025	GCTCAGAATGATTTCTGCATTGACGAGCCATCTTCACACTTTGCACTTGTCTGAGCTTACTGATTCTCTC	Similar to Q8BL99 (Q8BL99) DJ202D23.2 (Fragment)	Similar to Q8BL99 (Q8BL99) DJ202D23.2 (Fragment) / Gallus gallus finished cDNA, clone ChEST917p5 / Gallus gallus finished cDNA, clone ChEST917p5 / Finished cDNA, clone ChEST917p5 / --- / --- / --- / --- / dopey family member 1; / Gallus gallus finished cDNA, clone ChEST917p5 / Finished cDNA, clone ChEST917p5

Gene Name	Fold Change	p-value	SEQUENCE	Array Description	Blast/Database Description
RIGG01107	3.154	0.0494	GGTGCTTCCAAACCCGTGTATCCTG ATACATTTTCCTGAATCAAGAGCCTA GAATAATAACACAGAGCAT	Partial Contig Hit 047697.1	Partial Contig Hit 047697.1 / Gallus gallus finished cDNA, clone ChEST14c16 / Gallus gallus finished cDNA, clone ChEST14c16 / Finished cDNA, clone ChEST14c16 / similar to TGF-beta type II receptor / --- / --- / --- / --- / Gallus gallus finished cDNA, clone ChEST14c16 / Finished cDNA, clone ChEST14c16
RIGG04324	3.142	0.0234	GTCAACAAGTTGATTGCCGTCCCTA ACCTGTTCATGTTGGAAACAGTGGA TTCTGTGAAACTGGCAGACA	Similar to BAC27804 (BAC27804) Adult male olfactory brain cDNA, RIKEN	Similar to BAC27804 (BAC27804) Adult male olfactory brain cDNA, RIKEN / Gallus gallus finished cDNA, clone ChEST614m19 / Gallus gallus finished cDNA, clone ChEST614m19 / Finished cDNA, clone ChEST871g12 / --- / --- / --- / Finished cDNA, clone ChEST871g12; / proline synthetase co-transcribed homolog (bacterial); / Gallus gallus finished cDNA, clone ChEST614m19 / Finished cDNA, clone ChEST871g12
RIGG19353	3.14	0.0374	CTCCCCAAAGAAAATACTCCCTCCC TGAATATTTGCCGTTCAACCCTTCCA CAGTAACTTTTAGCATTAA	sulfotransferase 1C [Gallus gallus]. [Source:RefSeq;Acc:NM_204601]	sulfotransferase 1C [Gallus gallus]. [Source:RefSeq / UP|Q90WR6_CHICK (Q90WR6) Sulfotransferase 1C, complete / Gallus gallus sulfotransferase 1C (SULT1C), mRNA / Sulfotransferase 1C (SULT1C gene) / SULT1C: Sulfotransferase 1C / Gallus gallus sulfotransferase 1C (SULT1C), mRNA. / sulfotransferase 1C; / Sulfotransferase 1C (SULT1C gene); / --- / Gallus gallus sulfotransferase 1C (SULT1C), mRNA / Sulfotransferase 1C (SULT1C gene)
RIGG18911	3.139	0.0116	AGGCCCTTTACAAGTTGTTCTTCACT GACACCATTTAAATGAAATGTTTGGT TACCTGTATCCCTTGCCT		ENSGALT00000025875.1 / Gallus gallus finished cDNA, clone ChEST350j21 / 603472485F1 CSEQRBN22 Gallus gallus cDNA clone ChEST350j21 5', mRNA sequence / Finished cDNA, clone ChEST350j21 / --- / --- / --- / --- / --- / 603472485F1 CSEQRBN22 Gallus gallus cDNA clone ChEST350j21 5', mRNA sequence / Finished cDNA, clone ChEST350j21
RIGG17162	3.138	0.0246	GGCAGTTCCAGCTTCATGCAGACAA GACCATGGCGTATCCCCAGAGACAC CACCTCCTTTGGGAC		ENSGALT00000021186.1 / homologue to GB|CAI53860.1|63094391|AJ879909 immunoglobulin-like receptor CHIR-AB3 {Gallus gallus} (exp=-1; wgp=0; cg=0), complete / Gallus gallus mRNA for immunoglobulin-like receptor CHIR-B6 (CHIR-B6 gene), strain H.B19 / Immunoglobulin-like receptor CHIR-B6 (CHIR-B6 gene), strain H.B19 / --- / PREDICTED: Gallus gallus similar to immunoglobulin-like receptor CHIR-A (LOC425815), mRNA.PREDICTED: Gallus gallus similar to immunoglobulin-like receptor CHIR-A (LOC430639), mRNA. / PREDICTED: similar to immunoglobulin-like receptor CHIR-A;PREDICTED: similar to immunoglobulin-like receptor CHIR-A; / --- / --- / --- / ---
RIGG00671	3.136	0.0273	GAATGTTCTCCAAACTCAGTAGAGC TCGGGTACCTAAAACAGAAGGCAAA ACAATGAGTTCACCAGGAATG	Genome Hit Contig2.921	Genome Hit Contig2.921 / --- / 603593827F1 CSEQCHN34 Gallus gallus cDNA clone ChEST561f7 5', mRNA sequence / Finished cDNA, clone ChEST1015o3 / --- / --- / --- / --- / --- / --- / ---

Gene Name	Fold Change	p-value	SEQUENCE	Array Description	Blast/Database Description
RIGG11104	3.135	0.0151	CTGGTCCATCGAGAGTAGTCTGTTG CACTGAAGTACGTAGTGAAGATTCC ATGGATTTTGTAGATGAACGTTT		ENSGALT00000003886.1 / homologue to GB|AAH00359.2|38197127|BC000359 SPC18 protein {Homo sapiens} (exp=-1; wgp=0; cg=0), partial (95%) / Gallus gallus finished cDNA, clone ChEST437a5 / Finished cDNA, clone ChEST280c17 / RCJMB04_1h3: Hypothetical protein / --- / --- / --- / --- / --- / --- / ---
RIGG04790	3.13	0.00972	GTCCTTGGAAGCCTCCGCGTGACTC AGTTACATTTTCATAAATGTGAACTT GTTCTTGCCCTGCTTTACT	Similar to ZN46_HUMAN (P24278) Zinc finger protein 46 (Zinc finger pro	Similar to ZN46_HUMAN (P24278) Zinc finger protein 46 (Zinc finger pro / Gallus gallus finished cDNA, clone ChEST668d16 / Gallus gallus finished cDNA, clone ChEST668d16 / Finished cDNA, clone ChEST108h10 / similar to Zinc finger and BTB domain containing 25 / --- / --- / --- / --- / Gallus gallus finished cDNA, clone ChEST668d16 / Finished cDNA, clone ChEST108h10
RIGG01488	3.125	0.0122	AATGACGCAGCTTCAAAGGCAGCCT AATAGCGGAAACTCTTCCACCACAT ATTCTAACCCAGACACCTCC	Weakly similar to AAH54551 (AAH54551) 5930421I10 protein	Weakly similar to AAH54551 (AAH54551) 5930421I10 protein / Gallus gallus finished cDNA, clone ChEST204f1 / Gallus gallus finished cDNA, clone ChEST204f1 / Finished cDNA, clone ChEST204f1 / similar to Zinc finger and BTB domain containing 25 / --- / --- / --- / --- / Gallus gallus finished cDNA, clone ChEST204f1 / Finished cDNA, clone ChEST204f1
RIGG10264	3.115	0.0267	CTTCCGTGTGACATCAGTGATGGAG AGAAGGAGCTCATCCAAGTTGAAAC CCTCTTTTCTCCTGGCTGTG		ENSGALT00000001432.1 / --- / --- / --- / --- / --- / --- / --- / --- / --- / ---
RIGG15146	3.114	0.00999	ATTAGAGAAAAGAACCGCAGAGGAA ATTAAACAAGGCAATGATAATGGTG TCCTCGAATGGCCGTTTTGG		ENSGALT00000015502.1 / --- / --- / --- / --- / --- / --- / --- / --- / --- / ---
RIGG07685	3.059	0.035	CCGGGAAGACTGAAAACACTAAGAA GGTTATTCAATACCTTGCTCACGTT GCTTCCTCCCACAAAGGAAG	nonmuscle myosin heavy chain [Gallus gallus]. [Source:RefSeq;Acc:NM_205474]	nonmuscle myosin heavy chain [Gallus gallus]. [Source:RefSeq / UP|Q789A4_CHICK (Q789A4) Nonmuscle myosin heavy chain, complete / Chicken nonmuscle myosin heavy chain (including B1 and B2 inserts) mRNA, complete cds / Nonmuscle myosin heavy chain (including B1 and B2 inserts) / Nonmuscle myosin heavy chain / Gallus gallus nonmuscle myosin heavy chain (LOC396465), mRNA. / nonmuscle myosin heavy chain; / Nonmuscle myosin heavy chain (including B1 and B2 inserts); / --- / Chicken nonmuscle myosin heavy chain (including B1 and B2 inserts) mRNA, complete cds / Nonmuscle myosin heavy chain (including B1 and B2 inserts)
RIGG12766	3.055	0.00678	ACGTGCTTTCTCCGACCAGCACTAT TTACGTTCCTACTATACGCTTATTGA GCCTTACCTGGGAGGTGCT		ENSGALT00000008577.1 / similar to UP|Q6IRG1_RAT (Q6IRG1) Mesothelin, partial (3%) / --- / --- / --- / PREDICTED: Gallus gallus similar to megakaryocyte potentiating factor precursor; megakaryocyte potentiating factor; mesothelin isoform 1 precursor (LOC416534), mRNA. / PREDICTED: similar to megakaryocyte potentiating factor precursor; megakaryocyte potentiating factor; mesothelin isoform 1 precursor; / --- / --- / --- / ---

Gene Name	Fold Change	p-value	SEQUENCE	Array Description	Blast/Database Description
RIGG02388	3.055	0.0215	CCAAATAGTTCGAACTCCCTTAAGCCCTCAACAATGAACTTTTCAGACACCCACCGCTACGAGAAAAGCT	Similar to AAQ24157 (AAQ24157) Protein kinase LYK5 splice variant 1	Similar to AAQ24157 (AAQ24157) Protein kinase LYK5 splice variant 1 / Gallus gallus finished cDNA, clone ChEST334g5 / Gallus gallus mRNA for hypothetical protein, clone 13d22 / Protein kinase LYK5 (LOC419957), mRNA / STRAD, RCJMB04_13d22: STE20-related adapter protein / --- / --- / Protein kinase LYK5 (LOC419957), mRNA; / --- / Gallus gallus mRNA for hypothetical protein, clone 13d22 / Protein kinase LYK5 (LOC419957), mRNA
RIGG06492	3.052	0.0274	GGTGACTGTCTCCCTCTTGCTTCCTATAAAGACTCCTTGCTTCTATTTCCTATCTTTTCTATCACTCTTCTTCCC	Genome Hit Contig11.41	Genome Hit Contig11.41 / Gallus gallus finished cDNA, clone ChEST904k22 / gPGC_EST00913 Embryonic gonadal PGC cDNA Library Gallus gallus cDNA 5', mRNA sequence / Finished cDNA, clone ChEST904k22 / --- / --- / --- / --- / --- / gPGC_EST00913 Embryonic gonadal PGC cDNA Library Gallus gallus cDNA 5', mRNA sequence / Finished cDNA, clone ChEST904k22
RIGG01436	3.047	0.0488	TTCTTAAGGGTATGCTGCTCTTAGTATATCCTTCCAGAAAATGCACATTCGTAAAACTTGTGGCCAACACAG	Weakly similar to LEU3_SOLTU (P29696) 3-isopropylmalate dehydrogenase, chloro	Weakly similar to LEU3_SOLTU (P29696) 3-isopropylmalate dehydrogenase, chloro / homologue to UP|IDH3A_MOUSE (Q9D6R2) Isocitrate dehydrogenase [NAD] subunit alpha, mitochondrial precursor (Isocitric dehydrogenase) (NAD(+)-specific ICDH) , complete / Gallus gallus mRNA for hypothetical protein, clone 31a13 / Hypothetical protein, clone 31a13 / RCJMB04_31a13: Hypothetical protein / --- / PREDICTED: similar to isocitrate dehydrogenase 3 (NAD+) alpha; / --- / --- / Gallus gallus mRNA for hypothetical protein, clone 31a13 / Hypothetical protein, clone 31a13
RIGG03005	3.042	0.033	GCTTTTCAGACTTGAGCTTGATAAAATCTTTTGGCACATCCTCTCTTTCATCCATGGCTCCCTAGAAGGG	Similar to MS2L_HUMAN (O96007) Molybdenum cofactor synthesis protein 2	Similar to MS2L_HUMAN (O96007) Molybdenum cofactor synthesis protein 2 / Gallus gallus finished cDNA, clone ChEST422h16 / Gallus gallus finished cDNA, clone ChEST960c8 / Finished cDNA, clone ChEST359e5 / / PREDICTED: Gallus gallus similar to Molybdenum cofactor synthesis protein 2 large subunit (Molybdopterin synthase large subunit) (MPT synthase large subunit) (MOCS2B) (MOCO1-B) (LOC427199), mRNA. / PREDICTED: similar to Molybdenum cofactor synthesis protein 2 large subunit (Molybdopterin synthase large subunit) (MPT synthase large subunit) (MOCS2B) (MOCO1-B); / --- / --- / Gallus gallus finished cDNA, clone ChEST960c8 / Finished cDNA, clone ChEST359e5
RIGG05119	3.041	0.043	TCGTATACACACTCTTCAATAAAACCTATCGCAGGGCTTTCTCCAGTTATATCCGCTGCCAATACAAGACCAGC	Weakly similar to EAA03663 (EAA03663) EbiP6270 (Fragment)	Weakly similar to EAA03663 (EAA03663) EbiP6270 (Fragment) / --- / Gallus gallus finished cDNA, clone ChEST714c18 / Finished cDNA, clone ChEST714c18 / similar to 5-hydroxytryptamine 2C receptor / --- / --- / --- / 5-hydroxytryptamine (serotonin) receptor 2C; / Gallus gallus finished cDNA, clone ChEST714c18 / Finished cDNA, clone ChEST714c18

Gene Name	Fold Change	p-value	SEQUENCE	Array Description	Blast/Database Description
RIGG14941	3.038	0.0467	TGAAACCTACTGGGAACTGCGAAAGGATCCTGGATTTAGTAAAGTAGAAATTCCTGTACTCTGGTAAACTGAGAA		ENSGALT00000014942.1 / Gallus gallus finished cDNA, clone ChEST175h17 / Gallus gallus finished cDNA, clone ChEST175h17 / Finished cDNA, clone ChEST175h17 / similar to OSBP-related protein 6; ORP6 isoform 1 / --- / --- / --- / --- / Gallus gallus finished cDNA, clone ChEST175h17 / Finished cDNA, clone ChEST175h17
RIGG13278	3.037	0.0316	TCTGCTCTCAGTGGCTTGCTTCTTGCATCCTGTCATCGTCTGGCATGTCACCATACCTGGGTCCA		ENSGALT00000010131.1 / --- / --- / --- / --- / --- / --- / --- / --- / --- / ---
RIGG02463	3.032	0.0482	ACTCTGACTAGTAAATTGGAGTTCCTGGGCATCAACAGACAATCTATCTCCAACTTCCACATGTTGCTGT	Similar to Q9NT81 (Q9NT81) Hypothetical protein (Fragment)	Similar to Q9NT81 (Q9NT81) Hypothetical protein (Fragment) / Gallus gallus finished cDNA, clone ChEST350c23 / --- / --- / --- / PREDICTED: Gallus gallus similar to formin binding protein 4; formin binding protein 30 (LOC425905), mRNA. / PREDICTED: similar to formin binding protein 4; formin binding protein 30; / --- / --- / --- / ---
RIGG17384	3.017	0.0262	CCTTTAATTGTCCCGAATGTCCAAGGAGCTTTAAACACAGTTCCGCACTTTACTCCCACCAGTTTATCCA		ENSGALT00000021844.1 / weakly similar to UP|Q9R161_MOUSE (Q9R161) Zinc finger protein ZFP235, partial (22%) / Gallus gallus mRNA for hypothetical protein, clone 3j12 / Zinc finger protein 23 (KOX 16) (ZNF23), mRNA / / --- / --- / --- / --- / 603122316F1 CSEQCHL23 Gallus gallus cDNA clone ChEST86o3 5', mRNA sequence / Transcribed locus, weakly similar to XP_001072183.1 PREDICTED: similar to zinc finger protein 2 isoform 2 [Rattus norvegicus]
RIGG03618	3.006	0.0389	TTCCCTCATCTCCTGCTCATCAGCCATTCCTCTTGATGTTTTACCCATAGAAACTGTATTTTCCATGTAT	Weakly similar to EAA14324 (EAA14324) ENSANGP00000006196 (Fragment)	Weakly similar to EAA14324 (EAA14324) ENSANGP00000006196 (Fragment) / Gallus gallus finished cDNA, clone ChEST528a21 / --- / --- / hypothetical protein / PREDICTED: Gallus gallus similar to Protein disulfide isomerase A5 precursor (Protein disulfide isomerase-related protein) (LOC424249), mRNA. / PREDICTED: similar to Protein disulfide isomerase A5 precursor (Protein disulfide isomerase-related protein); / --- / --- / --- / ---
RIGG08096	2.99	0.0197	GGGAATTAACTGGTACCATTGGAAAGGCCATGAGTTCTCCATCCCCTTTGTGGAAATGAAGATGCGACCT	restrictin [Gallus gallus]. [Source:RefSeq;Acc:NM_205276]	restrictin [Gallus gallus]. [Source:RefSeq / UP|TENR_CHICK (Q00546) Tenascin-R precursor (TN-R) (Restrictin), complete / Gallus gallus tenascin R (restrictin, janusin) (TNR), mRNA / Restrictin / / Gallus gallus restrictin (LOC396213), mRNA. / restrictin; / Restrictin; / --- / Gallus gallus tenascin R (restrictin, janusin) (TNR), mRNA / Restrictin
RIGG03801	2.984	0.0274	GGCAGCCGGTGTGTGTACTCTTGAACACAATAAATGAGCAGGACTCTCCAAACTCCCGCACCAAC	Weakly similar to Q8R0W8 (Q8R0W8) Similar to hypothetical protein FLJ20367	Weakly similar to Q8R0W8 (Q8R0W8) Similar to hypothetical protein FLJ20367 / Gallus gallus finished cDNA, clone ChEST546m12 / 603126911F1 CSEQCHL13 Gallus gallus cDNA clone ChEST97f4 5', mRNA sequence / Finished cDNA, clone ChEST577e11 / --- / PREDICTED: Gallus gallus similar to chromosome 10 open reading frame 26 (LOC423867), mRNA. / PREDICTED: similar to chromosome 10 open reading frame 26; / --- / --- / 603126911F1 CSEQCHL13 Gallus gallus cDNA clone ChEST97f4 5', mRNA sequence / Finished cDNA, clone ChEST577e11

Gene Name	Fold Change	p-value	SEQUENCE	Array Description	Blast/Database Description
RIGG02969	2.981	0.00868	TTCATCATTCCTTTGCTACTCAACCTCACATGCTCTTCCTTAGTTCTCAGGACTCTCCGGAAGCCTGCCA	Similar to P2Y5_CHICK (P32250) P2Y purinoceptor 5 (P2Y5) (Purinergic r	Similar to P2Y5_CHICK (P32250) P2Y purinoceptor 5 (P2Y5) (Purinergic r / Gallus gallus finished cDNA, clone ChEST416f7 / Gallus gallus finished cDNA, clone ChEST5c20 / Finished cDNA, clone ChEST5c20 / hypothetical protein / PREDICTED: Gallus gallus similar to G protein-coupled receptor 23 (LOC422149), mRNA. / PREDICTED: similar to G protein-coupled receptor 23; / Finished cDNA, clone ChEST5c20; / --- / Gallus gallus finished cDNA, clone ChEST5c20 / Finished cDNA, clone ChEST5c20
RIGG08384	2.971	0.0376	GAAGGTGGCACTAGAATCACCATCACTGGCTCAAACCTCGGGCAGAAACATCAAGACATTGCAGAAACTG		ENSGALG00000006526.1 / --- / --- / --- / / --- / --- / --- / --- / --- / ---
RIGG13853	2.962	0.00143	GGAACATCTGACAAAGAAAATGCTGTATGACATGGAGAATCCTCCCTCTGATGACTACTTTGGCCGCTGT		ENSGALT00000011871.1 / UP|LPP_CHICK (Q5F464) Lipoma-preferred partner homolog, complete / Gallus gallus mRNA for hypothetical protein, clone 2l20 / LIM domain containing preferred translocation partner in lipoma (LPP), mRNA / LPP, RCJMB04_2l20: Lipoma-preferred partner homolog / --- / --- / LIM domain containing preferred translocation partner in lipoma (LPP), mRNA; / --- / Gallus gallus mRNA for hypothetical protein, clone 2l20 / LIM domain containing preferred translocation partner in lipoma (LPP), mRNA
RIGG14100	2.948	0.0176	CAAGCTTCGTAAATTGCCTTGTTCTCACGAGTATCACGTCCACTGCATCGATCGCTGGTTATCAGAAAATTCC	LIM domain interacting RING finger protein [Gallus gallus]. [Source:RefSeq;Acc:NM_204828]	LIM domain interacting RING finger protein [Gallus gallus]. [Source:RefSeq / UP|Q9W677_CHICK (Q9W677) LIM domain interacting RING finger protein, complete / Gallus gallus ring finger protein 12 (RNF12), mRNA / LIM domain interacting RING finger protein (R-LIM) / R-LIM: LIM domain interacting RING finger protein / Gallus gallus LIM domain interacting RING finger protein (R-LIM), mRNA. / LIM domain interacting RING finger protein; / LIM domain interacting RING finger protein (R-LIM); / --- / Gallus gallus ring finger protein 12 (RNF12), mRNA / LIM domain interacting RING finger protein (R-LIM)
RIGG07039	2.945	0.0248	CACTTTCCATGTTACGTAACATCGTGCCATGGTCTTAGAAGACAGAAGAGTACAAAAGCAAGCTCCTGCA	Weakly similar to RMP1_MOUSE (Q9WTJ5) Receptor activity-modifying protein 1 p	Weakly similar to RMP1_MOUSE (Q9WTJ5) Receptor activity-modifying protein 1 p / Gallus gallus finished cDNA, clone ChEST979n17 / Gallus gallus finished cDNA, clone ChEST979n17 / Finished cDNA, clone ChEST979n17 / similar to receptor activity modifying protein 1 / PREDICTED: Gallus gallus similar to receptor-activity modifying protein 1 (LOC424016), mRNA. / PREDICTED: similar to receptor-activity modifying protein 1; / --- / --- / Gallus gallus finished cDNA, clone ChEST979n17 / Finished cDNA, clone ChEST979n17
RIGG07590	2.944	0.0407	TACAACAAGCAGGACAACAACTGCCACTTCGATGCCACACTTGTCTCCTTCATCAACTTCTTCACCTCCG		ENSGALG00000000394.1 / --- / --- / --- / similar to solute carrier family 6 (neurotransmitter transporter), member 17 / --- / --- / --- / solute carrier family 6, member 17; / --- / ---

Gene Name	Fold Change	p-value	SEQUENCE	Array Description	Blast/Database Description
RIGG09648	2.936	0.0303	GGAGGTCTGTTCTGGATGCAGCTCA GATTGTTGGATTAAACTGTCTTAGAT TAATGAATGACATGACAGCTGTG		ENSGALG00000017077.1 / similar to UP|Q8VCW6_MOUSE (Q8VCW6) Heat shock protein 105, partial (33%) / gPGC_EST01939 Embryonic gonadal PGC cDNA Library Gallus gallus cDNA 5', mRNA sequence / Transcribed locus, weakly similar to NP_986878.1 AGR212Wp [Eremothecium gossypii] / similar to Heat-shock protein 105 kDa / PREDICTED: Gallus gallus similar to Heat-shock protein 105 kDa (Heat shock 110 kDa protein) (Antigen NY-CO-25) (LOC418917), mRNA. / PREDICTED: similar to Heat-shock protein 105 kDa (Heat shock 110 kDa protein) (Antigen NY-CO-25); / --- / --- / --- / ---
RIGG08806	2.933	0.0239	CTTTGTCATCTGTTGGATGCCCTTCT ACATTGTGCAGCTGGTCAATGTCTT TGTAGAGCAGGATGACGCC		ENSGALG00000010139.1 / --- / Gallus gallus somatostatin receptor type 4 precursor (SS4R) mRNA, complete cds / Somatostatin receptor 4 (SSTR4), mRNA / similar to somatostatin receptor / --- / --- / --- / somatostatin receptor 1; / --- / ---
RIGG03000	2.925	0.0498	TGCTGAGAAATTGTTAGTGTTTGCA GGCTATATCTGTTACAAGAAGGAAT CTGTGGGAATTAGTCCTTGCAG	Genome Hit Contig61.187	Genome Hit Contig61.187 / weakly similar to UP|Q5ZBJ7_ORYSA (Q5ZBJ7) Phosphatase 2C-like protein, partial (3%) / Gallus gallus finished cDNA, clone ChEST421d8 / Finished cDNA, clone ChEST421d8 / --- / --- / --- / --- / --- / Gallus gallus finished cDNA, clone ChEST421d8 / Finished cDNA, clone ChEST421d8
RIGG07661	2.912	0.0102	CCCCGATCAGCAGTTGTATGACCCT GTCCTCGTTTGTAACTCCTGTTATGA CCACATCCAAGTGTCTCGT		ENSGALG00000001042.1 / homologue to UP|Q91XS1_MOUSE (Q91XS1) FYVE zinc finger phosphatase (Myotubularin related protein 4) (17 days pregnant adult female amnion cDNA, RIKEN full-length enriched library, clone:I920037F18 product:myotubularin related protein 4, full insert sequence), partial (12%) / --- / --- / / PREDICTED: Gallus gallus similar to FYVE domain-containing dual specificity protein phosphatase FYVE-DSP2 (LOC417472), mRNA. / PREDICTED: similar to FYVE domain-containing dual specificity protein phosphatase FYVE-DSP2; / --- / --- / --- / ---
RIGG02505	2.889	0.0359	TCCTTGTTCAGATATTATTCTCAATT TAATCTTCCCTCAGCTCCCAACAAG TACTTGAGGCCAAAGTCCC	Weakly similar to AAN06980 (AAN06980) Caveolin 3	Weakly similar to AAN06980 (AAN06980) Caveolin 3 / UP|Q6URC6_CHICK (Q6URC6) Caveolin-3, partial (80%) / 603473759F1 CSEQCHN70 Gallus gallus cDNA clone ChEST354e15 5', mRNA sequence / Caveolin 3 (CAV3), mRNA / Caveolin-3 / Gallus gallus caveolin 3 (CAV3), mRNA. / caveolin 3; / --- / --- / 603473759F1 CSEQCHN70 Gallus gallus cDNA clone ChEST354e15 5', mRNA sequence / Caveolin 3 (CAV3), mRNA
RIGG14047	2.889	0.00627	AACATCTTCACCCTTGCTCTTATGGT TGTGAACCTGTTCAACATGTTCATCA CCTACGGAGACACCTTCC		ENSGALT00000012402.1 / --- / Gallus gallus finished cDNA, clone ChEST687g12 / Finished cDNA, clone ChEST687g12 / --- / PREDICTED: Gallus gallus similar to Hypothetical protein MGC63733 (LOC423850), mRNA. / PREDICTED: similar to Hypothetical protein MGC63733; / Finished cDNA, clone ChEST687g12; / --- / Gallus gallus finished cDNA, clone ChEST687g12 / Finished cDNA, clone ChEST687g12

Gene Name	Fold Change	p-value	SEQUENCE	Array Description	Blast/Database Description
RIGG10716	2.879	0.00621	TTCCCCAGAAGCACATCCTCAACACAGATGGTTGTTCACGAAATCAGAAATTACCCTTATCCTCAGCTCC		ENSGALT00000002710.1 / similar to UP|Q99ML9_MOUSE (Q99ML9) Arkadia, partial (5%) / --- / --- / --- / PREDICTED: Gallus gallus similar to ring finger protein 111; Arkadia (LOC416382), mRNA. / PREDICTED: similar to ring finger protein 111; Arkadia; / --- / --- / --- / ---
RIGG03344	2.866	0.0381	CAGAGGCATCCTGTTGATAAGGCTGAAACTTAAGCTTCAGATGTTTGGGAAAGAGCCTGCCTATGCCAGG	Weakly similar to Q7ZXW9 (Q7ZXW9) Similar to zinc finger protein 36, C3H type	Weakly similar to Q7ZXW9 (Q7ZXW9) Similar to zinc finger protein 36, C3H type / Gallus gallus finished cDNA, clone ChEST485h7 / Gallus gallus finished cDNA, clone ChEST53k20 / Finished cDNA, clone ChEST53k20 / --- / --- / --- / --- / --- / Gallus gallus finished cDNA, clone ChEST53k20 / Finished cDNA, clone ChEST53k20
RIGG03980	2.865	0.0185	GTTCCTCTGGGATCGCCTGTGGTGTCCTTTATTTATTACAATGAGTAGAACAGGAAAATCTTGTAGGAGGTG	Weakly similar to Q8K560 (Q8K560) Otospiralin	Weakly similar to Q8K560 (Q8K560) Otospiralin / --- / --- / --- / --- / --- / --- / --- / myeloma overexpressed 2; / --- / ---
RIGG05264	2.859	0.0038	GAATACAGTTCTTGTTTCCCTTCACTTTGTGACCTTTCACGCCCGTGAAATAAATGCGATTTGGAGAATT	Genome Hit Contig50.157	Genome Hit Contig50.157 / --- / Gallus gallus finished cDNA, clone ChEST738b18 / Finished cDNA, clone ChEST738b18 / --- / --- / --- / --- / --- / Gallus gallus finished cDNA, clone ChEST738b18 / Finished cDNA, clone ChEST738b18
RIGG04390	2.856	0.0347	CCCAACAGCTTCGACCACATCTACAACAGCACCAAACTGATGAACGTCCTCCACACCGCCGAGAT	Weakly similar to EAA05179 (EAA05179) AgCP6996 (Fragment)	Weakly similar to EAA05179 (EAA05179) AgCP6996 (Fragment) / Gallus gallus finished cDNA, clone ChEST624b22 / Gallus gallus finished cDNA, clone ChEST624b22 / Finished cDNA, clone ChEST624b22 / similar to PAN2 / PREDICTED: Gallus gallus similar to double substrate-specificity short chain dehydrogenase/reductase 2 (LOC423274), mRNA. / PREDICTED: similar to double substrate-specificity short chain dehydrogenase/reductase 2; / --- / --- / Gallus gallus finished cDNA, clone ChEST624b22 / Finished cDNA, clone ChEST624b22
RIGG15246	2.855	0.0319	TTGCCCAGATCCTCAAGCTCTCCTGCTCAAAATCTGACTACATCAGGGAAGTTGGGATTATTTATTTTAG		ENSGALT00000015755.1 / Gallus gallus finished cDNA, clone ChEST533c24 / Gallus gallus finished cDNA, clone ChEST533c24 / Finished cDNA, clone ChEST533c24 / similar to olfactory receptor 5 / --- / --- / --- / --- / --- / ---
RIGG02035	2.853	0.0165	TGTCTCTAGTGCTTCTACACCTCGTGCAAACCATAATTCGTAGTGTAATTACATGTTAGGTGTGTTGCCCTT	Weakly similar to O89032 (O89032) Fish protein	Weakly similar to O89032 (O89032) Fish protein / Gallus gallus finished cDNA, clone ChEST275m7 / 603369931F1 CSEQRBN19 Gallus gallus cDNA clone ChEST275m7 5', mRNA sequence / Finished cDNA, clone ChEST275m7 / --- / --- / --- / --- / --- / 603369931F1 CSEQRBN19 Gallus gallus cDNA clone ChEST275m7 5', mRNA sequence / Finished cDNA, clone ChEST275m7
RIGG01940	2.852	0.00599	CGGACTCCCAGATTGCTGAATGAAGTGATGCACAATGCTTGTCTCTAGAAAATACGCTATTTAACTTGCGC	Genome Hit Contig11.494	Genome Hit Contig11.494 / Gallus gallus finished cDNA, clone ChEST264i20 / 603365677F1 CSEQRBN21 Gallus gallus cDNA clone ChEST264i20 5', mRNA sequence / Finished cDNA, clone ChEST264i20 / --- / --- / --- / --- / --- / 603365677F1 CSEQRBN21 Gallus gallus cDNA clone ChEST264i20 5', mRNA sequence / Finished cDNA, clone ChEST264i20

Gene Name	Fold Change	p-value	SEQUENCE	Array Description	Blast/Database Description
RIGG00719	2.844	0.00539	GGGCTCCAGCAAACTTTGTTAATCTAATTGCAACTGGTCCATTAAGCTAGTTGGTTTGTTTACTGGCAGCA	Weakly similar to Q86Z26 (Q86Z26) Polycystic kidney and hepatic disease 1	Weakly similar to Q86Z26 (Q86Z26) Polycystic kidney and hepatic disease 1 / Gallus gallus finished cDNA, clone ChEST792p11 / Gallus gallus finished cDNA, clone ChEST1020n2 / Finished cDNA, clone ChEST792p11 / --- / --- / --- / --- / --- / Gallus gallus finished cDNA, clone ChEST1020n2 / Finished cDNA, clone ChEST792p11
RIGG18182	2.84	0.0173	ACCTCTGCTAGTTCTGTCTTGTGTCTCTCATTTTGGTGTGGAAAGGATTCCTTGTGTTTATATGGCACAT		ENSGALT00000023969.1 / --- / --- / --- / --- / --- / --- / --- / F-box protein 4; / --- / ---
RIGG17969	2.835	0.0343	TACGTCACGATCACCATCTCTATAATCATTGTCCTGGTTGCCACTGGGATCATATTCAAGTTCTGCTGGG		ENSGALT00000023359.1 / --- / pgp1n.pk001.f24 Normalized Chicken Pituitary/Hypothalamus/Pineal Library Gallus gallus cDNA clone pgp1n.pk001.f24 5' similar to dbj|BAB01625.1 (AB046043) unnamed protein product [Macaca fascicularis], mRNA sequence / Transcribed locus, strongly similar to XP_416507.1 PREDICTED: similar to Zinc finger protein 384 (Nuclear matrix transcription factor 4) (CAG repeat protein 1) [Gallus gallus] / --- / PREDICTED: Gallus gallus similar to Zinc finger protein 384 (Nuclear matrix transcription factor 4) (CAG repeat protein 1) (LOC418282), mRNA. / PREDICTED: similar to Zinc finger protein 384 (Nuclear matrix transcription factor 4) (CAG repeat protein 1); / --- / --- / --- / ---
RIGG01950	2.828	0.00785	GGTTTGATGAGAATAAGCATGTCCTTGGCTCAACTGGGATGTGTATCTTTATAATGCAGATTCTTGGCATGG	Similar to Q8BJU2 (Q8BJU2) Tetraspan NET-5 homolog	Similar to Q8BJU2 (Q8BJU2) Tetraspan NET-5 homolog / Gallus gallus finished cDNA, clone ChEST265n20 / Gallus gallus finished cDNA, clone ChEST74e1 / Finished cDNA, clone ChEST265n20 / similar to MGC80926 protein / PREDICTED: Gallus gallus similar to RIKEN cDNA 9430079M16 (LOC418263), mRNA. / PREDICTED: similar to RIKEN cDNA 9430079M16; / --- / --- / Gallus gallus finished cDNA, clone ChEST74e1 / Finished cDNA, clone ChEST265n20
RIGG04770	2.826	0.00234	CACCCGCTGCACTCTGCTCTGTCCTTCTTCTGCCTTATTTCCTTCTTTTCTTCTGGAGAGATGCTTTAGC	Genome Hit Contig3622.2	Genome Hit Contig3622.2 / --- / Gallus gallus finished cDNA, clone ChEST666e20 / Finished cDNA, clone ChEST666e20 / --- / --- / --- / --- / --- / Gallus gallus finished cDNA, clone ChEST666e20 / Finished cDNA, clone ChEST666e20
RIGG19380	2.82	0.0402	CAACTGTGCCAAGCCAGATCAAGATGTTAAGTTTACCATCAAATTTCAAGAATTCAGCCCTAATCTCTGGG	ephrin-B2 [Gallus gallus]. [Source:RefSeq;Acc:NM_204824]	ephrin-B2 [Gallus gallus]. [Source:RefSeq / UP|Q9PUJ4_CHICK (Q9PUJ4) Ephrin-B2 precursor, complete / Gallus gallus ephrin-B2 (EFNB2), mRNA / Ephrin-B2 precursor / Ephrin-B2 precursor / Gallus gallus ephrin-B2 (LOC395625), mRNA. / ephrin-B2; / Ephrin-B2 precursor; / --- / Gallus gallus ephrin-B2 (EFNB2), mRNA / Ephrin-B2 precursor
RIGG14986	2.819	0.0252	ACCACTCTCATCTCTGAAACCAGTTTCTTCATCGCCCCACAATCGATACAGATGTTGCTGTAGTCAGGCA		ENSGALT00000015064.1 / Gallus gallus finished cDNA, clone ChEST962h1 / 603143043F1 CSEQCHL16 Gallus gallus cDNA clone ChEST137f11 5', mRNA sequence / Finished cDNA, clone ChEST962h1 / --- / --- / --- / --- / --- / --- / ---

Gene Name	Fold Change	p-value	SEQUENCE	Array Description	Blast/Database Description
RIGG20087	2.815	0.0386	GGAGTCTGCACTCATCTTGGTTGTGTACCAATTGCTAACTCTGGAGATTTTGGTGGTTATTACTGCCCGT	Gallus gallus similar to ubiquinol-cytochrome c reductase, Rieske iron-sulfur polypeptide 1 (LOC415752), mRNA	Gallus gallus similar to ubiquinol-cytochrome c reductase, Rieske iron-sulfur polypeptide 1 (LOC415752), mRNA / RF|NP_001005843.1|57524866|NM_001005843 ubiquinol-cytochrome c reductase, Rieske iron-sulfur polypeptide 1 {Gallus gallus} (exp=-1; wgp=0; cg=0), complete / gPGC_EST01590 Embryonic gonadal PGC cDNA Library Gallus gallus cDNA 5', mRNA sequence / Ubiquinol-cytochrome c reductase, Rieske iron-sulfur polypeptide 1 (UQCRFS1), mRNA / RCJMB04_5b19: Hypothetical protein / PREDICTED: Gallus gallus similar to ubiquinol-cytochrome c reductase, Rieske iron-sulfur polypeptide 1 (LOC415752), mRNA. / PREDICTED: similar to ubiquinol-cytochrome c reductase, Rieske iron-sulfur polypeptide 1; / Ubiquinol-cytochrome c reductase, Rieske iron-sulfur polypeptide 1 (UQCRFS1), mRNA; / --- / gPGC_EST01590 Embryonic gonadal PGC cDNA Library Gallus gallus cDNA 5', mRNA sequence / Ubiquinol-cytochrome c reductase, Rieske iron-sulfur polypeptide 1 (UQCRFS1), mRNA
RIGG10429	2.815	0.0282	CATGAAGTTCCACATGGACCAGAAAACGTACTCCTGCATTTTCTGTCCTGAGTCCTTCGACCGCTTGGAC		ENSGALT00000001952.1 / --- / --- / --- / / --- / --- / Finished cDNA, clone ChEST47n3; / PR domain containing 10; / 603506312F1 CSEQCHN52 Gallus gallus cDNA clone ChEST431h4 5', mRNA sequence / Finished cDNA, clone ChEST47n3
RIGG02022	2.814	0.0164	GCTGGTCAGCAAACCGACTGTCAGCAATTCAGATGTGTCAGATAGAGAATAAAACAGTACCAAACCGCTC	Weakly similar to Q8ILD7 (Q8ILD7) Hypothetical protein, conserved	Weakly similar to Q8ILD7 (Q8ILD7) Hypothetical protein, conserved / --- / Gallus gallus finished cDNA, clone ChEST93d11 / Finished cDNA, clone ChEST273j17 / --- / PREDICTED: Gallus gallus similar to C9orf114 protein (LOC417203), mRNA. / PREDICTED: similar to C9orf114 protein; / --- / --- / Gallus gallus finished cDNA, clone ChEST93d11 / Finished cDNA, clone ChEST273j17
RIGG16866	2.813	0.0175	TCTCCTTATCAGCTATACCACTAATGCCTTCCCAGGAGAATACATCCCCACTGTGTTTGATAACTATTCT		ENSGALT00000020360.1 / UP|Q6PRV0_COTJA (Q6PRV0) Rac2 protein, complete / Gallus gallus mRNA for hypothetical protein, clone 2b11 / Ras-related C3 botulinum toxin substrate 2 (rho family, small GTP binding protein Rac2) (RAC2), mRNA / RCJMB04_38b23: Hypothetical protein / PREDICTED: Gallus gallus similar to Rac2 protein (LOC418044), mRNA. / PREDICTED: similar to Rac2 protein; / --- / --- / --- / ---
RIGG18118	2.806	0.0247	CTCCAACTCCCTCCTTAACCCCATCATCTATGCCTACTTCAACAAAGACTTCCAAAGTGCTTTTAAGAAA		ENSGALT00000023773.1 / --- / Gallus gallus cholinergic receptor, muscarinic 3 (CHRM3), mRNA / M3 muscarinic acetylcholine receptor (mACHR) / similar to serotonin 5-HT1a receptor / PREDICTED: Gallus gallus similar to 5-hydroxytryptamine 1A receptor (5-HT-1A) (Serotonin receptor 1A) (5-HT1A) (G-21) (LOC431581), mRNA. / PREDICTED: similar to 5-hydroxytryptamine 1A receptor (5-HT-1A) (Serotonin receptor 1A) (5-HT1A) (G-21); / --- / --- / --- / ---

Gene Name	Fold Change	p-value	SEQUENCE	Array Description	Blast/Database Description
RIGG10751	2.794	0.00487	CCACCATAGAACTGCCATGATCAGAAGGCCACGTAATCAGATGGAAATAACCTCCTCTTCTGCTGTGCTG		ENSGALT00000002853.1 / RF|NP_001025797.1|71894815|NM_001030626 protein inhibitor of activated STAT X {Gallus gallus} (exp=-1; wgp=0; cg=0), complete / Gallus gallus mRNA for hypothetical protein, clone 24l12 / Protein inhibitor of activated STAT, 2 (PIAS2), mRNA / --- / --- / --- / --- / --- / Gallus gallus mRNA for hypothetical protein, clone 24l12 / Protein inhibitor of activated STAT, 2 (PIAS2), mRNA
RIGG04577	2.791	0.00762	AAGTGGGTAGAGCGTAGATTCATTGTTCTTTGTATCCACGTACAGTGAACACTTCTTGCCCATTCCTGCG	Similar to Q8BX87 (Q8BX87) Hypothetical serine-rich region/tyrosine sp	Similar to Q8BX87 (Q8BX87) Hypothetical serine-rich region/tyrosine sp / Gallus gallus finished cDNA, clone ChEST647b2 / Gallus gallus finished cDNA, clone ChEST647b2 / Finished cDNA, clone ChEST647b2 / hypothetical protein / --- / --- / --- / dual specificity phosphatase 27 (putative); / Gallus gallus finished cDNA, clone ChEST647b2 / Finished cDNA, clone ChEST647b2
RIGG09545	2.789	0.0159	CCAGGACACAAGCACAGGTTTGGAGGAAGTGATGGAGCAGCTTAACAACTCCTTCCCCAGTTCCA	Dystrophin. [Source:SWISSPROT;Acc:P11533]	Dystrophin. [Source:SWISSPROT / dystrophin [Gallus gallus] / Gallus gallus dystrophin (muscular dystrophy, Duchenne and Becker types) (DMD), mRNA / Dystrophin (Duchenne muscular dystrophy gene homolog) / --- / --- / --- / --- / --- / Gallus gallus dystrophin (muscular dystrophy, Duchenne and Becker types) (DMD), mRNA / Dystrophin (Duchenne muscular dystrophy gene homolog)
RIGG02104	2.788	0.0327	GGCAAATGCGAGTAATGAAAGTTGTTTTCAAGTGTAGTGTCTTGTGCAGTCCATAAGAGGCCACTACAAACTA	Genome Hit Contig192.6	Genome Hit Contig192.6 / Gallus gallus finished cDNA, clone ChEST286o14 / --- / --- / --- / --- / --- / --- / --- / --- / ---
RIGG02605	2.788	0.0163	TACTCGATCGAACAATAAGGATCTTGAGAAATCAGAAAATGCTATTAGAGGAGGCTCGGTTTGTCCTGTCAAG	Weakly similar to O76340 (O76340) Soluble guanylyl cyclase beta-3	Weakly similar to O76340 (O76340) Soluble guanylyl cyclase beta-3 / Gallus gallus finished cDNA, clone ChEST369m20 / Gallus gallus finished cDNA, clone ChEST369m20 / Finished cDNA, clone ChEST369m20 / hypothetical protein / PREDICTED: Gallus gallus similar to guanylate cyclase 1, soluble, beta 2 (LOC429128), mRNA. / PREDICTED: similar to guanylate cyclase 1, soluble, beta 2; / Finished cDNA, clone ChEST369m20; / --- / Gallus gallus finished cDNA, clone ChEST369m20 / Finished cDNA, clone ChEST369m20

Gene Name	Fold Change	p-value	SEQUENCE	Array Description	Blast/Database Description
RIGG10044	2.779	0.00277	CCTGGACCTGCTCAACCTCATCAACCAGTTCTTTCTGTTCTTCAAGTGCTCGGTGACGCCCGTCC		ENSGALT00000000849.1 / similar to GB|AAH50334.1|29791434|BC050334 G-protein coupled receptor 37 like 1 {Homo sapiens} (exp=-1; wgp=0; cg=0), partial (68%) / pgp2n.pk007.c21 Normalized Chicken Pituitary/Hypothalamus/Pineal Library (pgp2n) Gallus gallus cDNA clone pgp2n.pk007.c21 5' similar to gb|AAD54656.1|AF087947_1 (AF087947) G protein-coupled receptor GPCR/CNS2 [Rattus norvegicus], mRNA sequence / Transcribed locus, weakly similar to XP_001062802.1 PREDICTED: similar to Endothelin B receptor-like protein 2 precursor (ETBR-LP-2) (G-protein coupled receptor 37-like 1) (G-protein coupled receptor CNS2) [Rattus norvegicus] / similar to ETBR-LP-2 protein / PREDICTED: Gallus gallus similar to Endothelin B receptor-like protein-2 precursor (ETBR-LP-2) (G protein-coupled receptor 37 like 1) (LOC421176), mRNA. / PREDICTED: similar to Endothelin B receptor-like protein-2 precursor (ETBR-LP-2) (G protein-coupled receptor 37 like 1); / --- / --- / --- / ---
RIGG03783	2.779	0.0383	ACCAGTCCCCTTTTGGCTTTTGTGAAGAGACAAAGCTACACTCTGTTCAGTTCCTCCCTGTTTACCAGAC	Weakly similar to HPS3_HUMAN (Q969F9) Hermansky-Pudlak syndrome 3 protein	Weakly similar to HPS3_HUMAN (Q969F9) Hermansky-Pudlak syndrome 3 protein / similar to UP|HPS3_HUMAN (Q969F9) Hermansky-Pudlak syndrome 3 protein, partial (23%) / --- / --- / --- / --- / --- / --- / --- / --- / ---
RIGG19052	2.771	0.0192	TCTGTCCTGAGCTATACATTTGCTATATCCTCAGACTCACATGACTGATATTAATGCTGTATCCGCGTGAGT		ENSGALT00000026280.1 / --- / --- / --- / --- / --- / --- / --- / --- / --- / ---
RIGG04571	2.768	0.00636	GCACGTTTCATAGCTATTGCTCTGGCAAGTCAAAGTTGCAGCCTTTCTCATGACAGCTTTTCCTCTACTG	Weakly similar to Q9BSW4 (Q9BSW4) Hypothetical protein	Weakly similar to Q9BSW4 (Q9BSW4) Hypothetical protein / similar to UP|Q5E9J4_BOVIN (Q5E9J4) RAB, member RAS oncogene family-like 5, partial (75%) / Gallus gallus finished cDNA, clone ChEST646i7 / Finished cDNA, clone ChEST571e23 / --- / PREDICTED: Gallus gallus similar to RAB, member RAS oncogene family-like 5; RAB, member of RAS oncogene family-like 5 (LOC417503), mRNA. / PREDICTED: similar to RAB, member RAS oncogene family-like 5; RAB, member of RAS oncogene family-like 5; / Finished cDNA, clone ChEST571e23; / --- / Gallus gallus finished cDNA, clone ChEST646i7 / Finished cDNA, clone ChEST571e23
RIGG03072	2.742	0.0356	CCCGGGTAATTACAGGGTTAATAGTCCGGTTCTGTGTACTGACAACCTTCCAGATACCTGCTTCTCGAAA	Same gene AF218784; Gallus gallus MHC Rfp-Y class I alpha chain (YFVI) pseudogene, YFV-Y-FVw*7	Same gene AF218784; Gallus gallus MHC Rfp-Y class I alpha chain (YFVI) pseudogene, YFV-Y-FVw*7 / Gallus gallus finished cDNA, clone ChEST432k7 / gonad_EST06851 Embryonic gonad cDNA Library Gallus gallus cDNA 5', mRNA sequence / Finished cDNA, clone ChEST432k7 / --- / PREDICTED: Gallus gallus similar to class I alpha chain (LOC426689), partial mRNA. / PREDICTED: similar to MHC Rfp-Y class I alpha chain, partial;PREDICTED: similar to class I alpha chain, partial; / --- / --- / spleen_EST00172 Spleen cDNA Library Gallus gallus cDNA 3', mRNA sequence / Finished cDNA, clone ChEST170d6

Gene Name	Fold Change	p-value	SEQUENCE	Array Description	Blast/Database Description
RIGG00226	2.74	0.00852	CCCTCTTCACTTGTGTCCTTCCCAA CATCACTGCAGCATCTATCTATATAA TAACATCTGGGTGGGTTTATA	Gallus gallus mRNA for hypothetical protein, clone 16g11	Gallus gallus mRNA for hypothetical protein, clone 16g11 / similar to UP|Q9W482_DROME (Q9W482) CG15929-PA (Myb-MuvB complex subunit Lin-52), partial (12%) / Gallus gallus mRNA for hypothetical protein, clone 16g11 / Similar to hypothetical protein 5830457H20 (LOC423346), mRNA / --- / --- / --- / --- / --- / --- / Gallus gallus mRNA for hypothetical protein, clone 16g11 / Similar to hypothetical protein 5830457H20 (LOC423346), mRNA
RIGG17348	2.738	0.0219	CAGAAGATACACCCAGAAGGCAGTT CTCCCCTAACGGTGTGAACAAATCC CTCTGCAGCGACACG		ENSGALT00000021737.1 / --- / --- / --- / similar to MGC80729 protein / --- / --- / --- / --- / --- / ---
RIGG11503	2.736	0.00327	TGAAGCTGGCCGTGCTGAGCTCTCC CTCATTTATCCACGCTGAACAATGG CCTCTTGTTTTCAAG		ENSGALT00000004951.1 / --- / 603762166F1 CSEQRBN21 Gallus gallus cDNA clone ChEST682c18 5', mRNA sequence / Transcribed locus / --- / --- / --- / --- / --- / 603762166F1 CSEQRBN21 Gallus gallus cDNA clone ChEST682c18 5', mRNA sequence / Transcribed locus
RIGG03478	2.735	0.0173	AGGAGGTTGTGAGCAGAAAACGTAA TGCCTCAGTGCTTTAGTAGTGTTAG ATTGTCGGTGCTGAACGTAG	Contig Hit 344146.1	Contig Hit 344146.1 / Gallus gallus finished cDNA, clone ChEST500a2 / --- / --- / --- / --- / --- / --- / --- / --- / ---
RIGG15169	2.724	0.0417	AAACTCGAGCCCTGCATCTCAGTAT TTAAGCAAAAGCATGCACCAATACC TCATGAAAGGGGACGAAGGC		ENSGALT00000015559.1 / --- / --- / --- / --- / --- / --- / --- / --- / --- / ---
RIGG03375	2.721	0.0066	AGAAGTGGCACGTAATCACTCATTT CTCAGGTGCGTTCCAAAAGAAAGAT ACTGTATTTGTATCTGATGCAG	Contig Hit 003742.1	Contig Hit 003742.1 / Gallus gallus finished cDNA, clone ChEST48a6 / Gallus gallus finished cDNA, clone ChEST48a6 / Finished cDNA, clone ChEST48a6 / --- / --- / --- / --- / --- / Gallus gallus finished cDNA, clone ChEST48a6 / Finished cDNA, clone ChEST48a6
RIGG14187	2.712	0.0067	CACCATTGCAGGGGTTGTCACTACC TTCTTCCTGTTGGTTTATGCCACTGT CAACCTGGCCTGCC		ENSGALT00000012821.1 / --- / --- / --- / similar to solute carrier family 12 (potassium/chloride transporters), member 9; cation-chloride cotransporter-interacting protein 1 / --- / --- / --- / solute carrier family 12 (potassium/chloride transporters), member 9; / --- / ---
RIGG03825	2.709	0.0162	TTTAGCATCAACTGTACCCAGATCTT CCGTTTCCCTTACTCATAAGGATCC TGTCAGATGTGGCTGAAGG	Weakly similar to Q93ZU3 (Q93ZU3) Putative serine/threonine kinase	Weakly similar to Q93ZU3 (Q93ZU3) Putative serine/threonine kinase / Gallus gallus finished cDNA, clone ChEST549e20 / Gallus gallus finished cDNA, clone ChEST637h3 / Finished cDNA, clone ChEST637h3 / similar to Hypothetical protein CBG04537 / --- / --- / Finished cDNA, clone ChEST637h3; / --- / --- / ---
RIGG05827	2.708	0.0224	TGGTTGTGAGGTTTCCACATCTCTG TCTCGTATTCTGTGCAGGGACATTG TTGTGAAAGTGGTGTTTGTG	Contig Hit 038108.1	Contig Hit 038108.1 / Gallus gallus finished cDNA, clone ChEST807j6 / BX262403 AGENAE Gallus gallus multi-tissues normalized and once-subtracted cDNA library (gcal) Gallus gallus cDNA clone gcal0008.a.04 3prim, mRNA sequence / Finished cDNA, clone ChEST807j6 / --- / --- / --- / --- / --- / BX262403 AGENAE Gallus gallus multi-tissues normalized and once-subtracted cDNA library (gcal) Gallus gallus cDNA clone gcal0008.a.04 3prim, mRNA sequence / Finished cDNA, clone ChEST807j6

Gene Name	Fold Change	p-value	SEQUENCE	Array Description	Blast/Database Description
RIGG18464	2.708	0.0212	CCAGTTCTCACAGTCAATTCTCCTTTTCCAACAGTGAAAAGCTATGTTCTCTATATCACCACGGGAACAT		ENSGALT00000024725.1 / --- / --- / --- / --- / --- / --- / --- / --- / --- / ---
RIGG14458	2.693	0.0483	AACCATTGAAACAGATTCTGGGGAGCAAGATTATTCTCTAGAAACAGATGCAAGATTTCAGCTGGACTGC	caspase 8 [Gallus gallus]. [Source:RefSeq;Acc:NM_204592]	caspase 8 [Gallus gallus]. [Source:RefSeq / UP|Q90WU1_CHICK (Q90WU1) Caspase 8, complete / Gallus gallus caspase 8, apoptosis-related cysteine peptidase (CASP8), mRNA / Caspase 8 / Caspase 8 / Gallus gallus caspase 8 (LOC395284), mRNA. / caspase 8; / Caspase 8; / --- / Gallus gallus caspase 8, apoptosis-related cysteine peptidase (CASP8), mRNA / Caspase 8
RIGG18724	2.693	0.0188	TCCAAGGACAAGTTTCTGTTTCCAGTATGAGATAACAGCAGCCCACATTCTTCAGACAAGTCAATCTGAAT		ENSGALT00000025386.1 / similar to GB|AAH04896.2|38014060|BC004896 MRPL39 protein {Homo sapiens} (exp=-1; wgp=0; cg=0), partial (89%) / gonad_EST08537 Embryonic gonad cDNA Library Gallus gallus cDNA 5', mRNA sequence / Finished cDNA, clone ChEST173k14 / --- / PREDICTED: Gallus gallus similar to mitochondrial ribosomal protein L39 isoform a (LOC418475), mRNA. / PREDICTED: similar to mitochondrial ribosomal protein L39 isoform a; / --- / --- / gonad_EST08537 Embryonic gonad cDNA Library Gallus gallus cDNA 5', mRNA sequence / Finished cDNA, clone ChEST173k14
RIGG02467	2.69	0.0123	CCGCTGTCAACGCGCTCTGCATCATCGGCACAGAAGAGGCATATAACGTCATCGATAGGCAGAAG	Same gene L12469; Gallus gallus (max) gene, complete cds	Same gene L12469; Gallus gallus (max) gene, complete cds / Gallus gallus finished cDNA, clone ChEST350k16 / Gallus gallus finished cDNA, clone ChEST350k16 / Finished cDNA, clone ChEST350k16 / --- / --- / --- / --- / --- / Gallus gallus finished cDNA, clone ChEST350k16 / Finished cDNA, clone ChEST350k16
RIGG06513	2.687	0.00508	AGACTCATCTCCGTAGTGTCTGGATATAGTAAAGTATGTGTCCCTAATCAGTTTGTCCTCACAGAATCCTTGTGA	Similar to PWP2_MOUSE (Q8BU03) Periodic tryptophan protein 2 homolog	Similar to PWP2_MOUSE (Q8BU03) Periodic tryptophan protein 2 homolog / Gallus gallus finished cDNA, clone ChEST907e5 / Gallus gallus finished cDNA, clone ChEST907e5 / Finished cDNA, clone ChEST907e5 / --- / PREDICTED: Gallus gallus similar to WD-repeat protein p103 (LOC425645), partial mRNA. / PREDICTED: similar to WD-repeat protein p103, partial; / --- / --- / Gallus gallus finished cDNA, clone ChEST907e5 / Finished cDNA, clone ChEST907e5
RIGG18845	2.686	0.00405	GTTATGGAAAGGTCTCCTTCAACGTCAGTATCTTCAGTTGACATGAGAGTGATGATGTCTCCCTCTCCTGTG		ENSGALT00000025713.1 / Gallus gallus finished cDNA, clone ChEST261g12 / gPGC_EST10726 Embryonic gonadal PGC cDNA Library Gallus gallus cDNA 5', mRNA sequence / Finished cDNA, clone ChEST261g12 / --- / --- / --- / Finished cDNA, clone ChEST261g12; / --- / gPGC_EST10726 Embryonic gonadal PGC cDNA Library Gallus gallus cDNA 5', mRNA sequence / Finished cDNA, clone ChEST261g12

Gene Name	Fold Change	p-value	SEQUENCE	Array Description	Blast/Database Description
RIGG16021	2.678	0.0248	TGAGTCAGAGCTCCAGTGAAAACAG AACCCCACTAGAGGAGCAGCTACAC TGAATATTTAAGAAGTCCGAGA		ENSGALT00000017872.1 / --- / 603494148F1 CSEQCHN64 Gallus gallus cDNA clone ChEST401o11 5', mRNA sequence / Transcribed locus, strongly similar to XP_420547.1 PREDICTED: similar to dehydrogenase/reductase (SDR family) member 8; retinal short-chain dehydrogenase/reductase 2; 17-beta-hydroxysteroid dehydrogenase type XI; 17-BETA-HSD11; 17-BETA-HSDXI [Gallus gallus] / / --- / --- / --- / --- / 603494148F1 CSEQCHN64 Gallus gallus cDNA clone ChEST401o11 5', mRNA sequence / Transcribed locus, strongly similar to XP_420547.1 PREDICTED: similar to dehydrogenase/reductase (SDR family) member 8; retinal short-chain dehydrogenase/reductase 2; 17-beta-hydroxysteroid dehydrogenase type XI; 17-BETA-HSD11; 17-BETA-HSDXI [Gallus gall
RIGG03672	2.667	0.0169	TTTAAATGTGTACATTTCTTACCGAA CCGATTATGTTTTGTCGCTGTGAAC CACGAGTCGCCTCCATCTG	Genome Hit Contig96.45	Genome Hit Contig96.45 / Gallus gallus finished cDNA, clone ChEST533h5 / Gallus gallus finished cDNA, clone ChEST533h5 / Finished cDNA, clone ChEST533h5 / --- / --- / --- / --- / --- / Gallus gallus mRNA for hypothetical protein, clone 24h23 / 5'-3' exoribonuclease 2 (XRN2), mRNA
RIGG00629	2.664	0.0106	ACCTAGTGATTCTTCCGCAGTAAGA GTGGAAAGTACAGTTTCTAGGTTTT ATGGAGAAAATGCATGGTGG	Weakly similar to Q63094 (Q63094) Calcium/calmodulin-dependent protein kinase	Weakly similar to Q63094 (Q63094) Calcium/calmodulin-dependent protein kinase / Gallus gallus finished cDNA, clone ChEST1012h6 / Gallus gallus finished cDNA, clone ChEST1012h6 / Finished cDNA, clone ChEST1012h6 / RCJMB04_10k21: Hypothetical protein / --- / --- / Calcium/calmodulin-dependent protein kinase (CaM kinase) II delta (CAMK2D), mRNA; / --- / Gallus gallus finished cDNA, clone ChEST1012h6 / Finished cDNA, clone ChEST1012h6
RIGG01213	2.662	0.0312	GTGTTAAACACACCAATATGAACCG ACTCCGTAATGATGGGAAATAGCTT TTCTGGTCCATTTGCAAACT	Weakly similar to Q9UYA1 (Q9UYA1) Similar to SCHIZOSACCHAROMYCES POMBE 4-nitr	Weakly similar to Q9UYA1 (Q9UYA1) Similar to SCHIZOSACCHAROMYCES POMBE 4-nitr / Gallus gallus finished cDNA, clone ChEST168k20 / Gallus gallus finished cDNA, clone ChEST168k20 / Finished cDNA, clone ChEST168k20 / / --- / --- / Finished cDNA, clone ChEST168k20; / haloacid dehalogenase-like hydrolase domain containing 2; / Gallus gallus finished cDNA, clone ChEST168k20 / Finished cDNA, clone ChEST168k20
RIGG01063	2.649	0.0457	GAATGTTGCCTTCCCCACTGTCGTG TTAAGTAACCATTTCATCCTGGCCCT GTCCAGTAGTGTTCACCTC	Contig Hit 332653.4	Contig Hit 332653.4 / Gallus gallus finished cDNA, clone ChEST142l8 / Gallus gallus finished cDNA, clone ChEST142l8 / Finished cDNA, clone ChEST194c24 / --- / --- / --- / --- / --- / Gallus gallus finished cDNA, clone ChEST142l8 / Finished cDNA, clone ChEST194c24
RIGG00617	2.646	0.0326	AAGGGCATCACATTGACTGAACCCA TCTCCACTATGAACCTTGTTTATCCC TTCTGTTATTAGAATGTCT	Weakly similar to Q8K463 (Q8K463) Mesoderm-specific transcript isoform 2	Weakly similar to Q8K463 (Q8K463) Mesoderm-specific transcript isoform 2 / Gallus gallus finished cDNA, clone ChEST1010p22 / Gallus gallus finished cDNA, clone ChEST1010p22 / Finished cDNA, clone ChEST1010p22 / --- / --- / --- / --- / --- / Gallus gallus finished cDNA, clone ChEST1010p22 / Finished cDNA, clone ChEST1010p22

Gene Name	Fold Change	p-value	SEQUENCE	Array Description	Blast/Database Description
RIGG14781	2.644	0.0332	ACACTGGAAAGAAATCTGTGAGTAT CCTAGGAGACAAATCGCCAAATGGC ATATGTTGTGTGATGGTTAG		ENSGALT00000014456.1 / homologue to UP|Q2XQH1_POEGU (Q2XQH1) Synaptotagmin IV, partial (96%) / 603137419F1 CSEQCHL25 Gallus gallus cDNA clone ChEST123f5 5', mRNA sequence / Transcribed locus, weakly similar to NP_477464.1 Synaptotagmin IV CG10047-PA [Drosophila melanogaster] / / PREDICTED: Gallus gallus similar to Synaptotagmin IV (SytIV) (LOC422960), mRNA. / PREDICTED: similar to Synaptotagmin IV (SytIV); / --- / --- / --- / ---
RIGG02428	2.641	0.0104	TGGTCATTTGTCTTAGCTATTTCATC TCACTTGCAGATGCAGTCATAAAGA TTAGCGTAGATCAGCTTGGTGC	Weakly similar to Q08476 (Q08476) Connectin(titin) (Fragment)	Weakly similar to Q08476 (Q08476) Connectin(titin) (Fragment) / Gallus gallus finished cDNA, clone ChEST340p11 / Gallus gallus finished cDNA, clone ChEST340p11 / Finished cDNA, clone ChEST340p11 / / PREDICTED: Gallus gallus similar to Tyrosine-protein kinase transmembrane receptor ROR2 precursor (Neurotrophic tyrosine kinase, receptor-related 2) (LOC427268), partial mRNA. / PREDICTED: similar to Tyrosine-protein kinase transmembrane receptor ROR2 precursor (Neurotrophic tyrosine kinase, receptor-related 2), partial; / --- / --- / Gallus gallus finished cDNA, clone ChEST340p11 / Finished cDNA, clone ChEST340p11
RIGG20146	2.631	0.0211	AGCTCTCTCCTACGTTCTTGCAAGG AAAATGGCTGCTCTGCATATGTTGG ATATTAAAGAGCACAATGGC	Gallus gallus similar to molecule possessing ankyrin repeats induced by lipopolysaccharide; likely ortholog of mouse I kappa B-zeta (LOC418404), mRNA	Gallus gallus similar to molecule possessing ankyrin repeats induced by lipopolysaccharide; likely ortholog of mouse I kappa B-zeta (LOC418404), mRNA / RF|NP_001006254.1|57525458|NM_001006254 nuclear factor of kappa light polypeptide gene enhancer in B-cells inhibitor, zeta {Gallus gallus} (exp=-1; wgp=0; cg=0), complete / Gallus gallus mRNA for hypothetical protein, clone 35i9 / Nuclear factor of kappa light polypeptide gene enhancer in B-cells inhibitor, zeta (NFKBIZ), mRNA / --- / PREDICTED: Gallus gallus similar to molecule possessing ankyrin repeats induced by lipopolysaccharide; likely ortholog of mouse I kappa B-zeta (LOC418404), mRNA. / PREDICTED: similar to molecule possessing ankyrin repeats induced by lipopolysaccharide; likely ortholog of mouse I kappa B-zeta; / Nuclear factor of kappa light polypeptide gene enhancer in B-cells inhibitor, zeta (NFKBIZ), mRNA; / --- / Gallus gallus mRNA for hypothetical protein, clone 35i9 / Nuclear factor of kappa light polypeptide gene enhancer in B-cells inhibitor, zeta (NFKBIZ), mRNA
RIGG02087	2.63	0.0378	AGACGTCTGTACAGTGTATCTGCCA AGGGAAGACAACGGTGCATGAATAT TAATGTACTATACTCTCATACCAA	Weakly similar to Q96LW7 (Q96LW7) Hypothetical protein FLJ33154 (Hypothetical	Weakly similar to Q96LW7 (Q96LW7) Hypothetical protein FLJ33154 (Hypothetical / Gallus gallus finished cDNA, clone ChEST282k8 / Gallus gallus finished cDNA, clone ChEST282k8 / Finished cDNA, clone ChEST282k8 / similar to chromosome 9 open reading frame 89 / --- / --- / --- / --- / Gallus gallus finished cDNA, clone ChEST282k8 / Finished cDNA, clone ChEST282k8

Gene Name	Fold Change	p-value	SEQUENCE	Array Description	Blast/Database Description
RIGG13160	2.623	0.0069	CGAATTTCAGAGGAAGACTCAGTATCTCTTTGAGAAGACTTTTAACATGAACTTCATCTCGCCGTTCACGCAG	heparan sulfate 6-O-sulfotransferase-2 [Gallus gallus]. [Source:RefSeq;Acc:NM_204490]	heparan sulfate 6-O-sulfotransferase-2 [Gallus gallus]. [Source:RefSeq / UP|H6ST2_CHICK (Q76LW2) Heparan-sulfate 6-O-sulfotransferase 2 (HS6ST-2) (cHS6ST-2) , complete / Gallus gallus cHS6ST-1 mRNA for heparan sulfate 6-O-sulfotransferase, complete cds / Heparan sulfate 6-O-sulfotransferase 1 (HS6ST1), mRNA / HS6ST2, RCJMB04_19a20: Heparan-sulfate 6-O-sulfotransferase 2 / Gallus gallus heparan sulfate 6-O-sulfotransferase-2 (HS6ST-2), mRNA. / heparan sulfate 6-O-sulfotransferase-2; / --- / --- / Gallus gallus mRNA for hypothetical protein, clone 19a20 / Hypothetical protein, clone 19a20
RIGG06014	2.613	0.0216	TTTCACATCTCTAAGAATTGCCTGTCCCTGTTTCCCGGTGTCTTGTCACTGCTTTGTTCCCAGAGCTACC	Genome Hit Contig1.467	Genome Hit Contig1.467 / Gallus gallus finished cDNA, clone ChEST833a4 / Gallus gallus finished cDNA, clone ChEST833a4 / Finished cDNA, clone ChEST833a4 / --- / --- / --- / --- / --- / Gallus gallus finished cDNA, clone ChEST833a4 / Finished cDNA, clone ChEST833a4
RIGG09759	2.611	0.0395	GATTTTCTACAACCAAGACGCATGCTACAGACTTGATGTCCTGAATCTTTTGGGCATTGAATACTGCCGT		ENSGALT00000000163.1 / --- / --- / --- / --- / --- / --- / --- / gastrokine 1; / --- / ---
RIGG02834	2.608	0.0389	CTCTCACCTTTTCTTCTGACTTCAGTAAAGAAGCGTTGAAACTGGTTGGCTTGGAGACATCCCTGCTGTG	Genome Hit Contig7.400	Genome Hit Contig7.400 / --- / 603491878F1 CSEQCHN63 Gallus gallus cDNA clone ChEST395k20 5', mRNA sequence / Finished cDNA, clone ChEST395k20 / --- / --- / --- / --- / --- / 603491878F1 CSEQCHN63 Gallus gallus cDNA clone ChEST395k20 5', mRNA sequence / Finished cDNA, clone ChEST395k20
RIGG06263	2.605	0.00355	CGAACTGCTGTACAACTCTGGTGTTCCTAGGAACTGTGTTTTCTTTGAGAAATGGTGGTAATGAACAGAAAG	Similar to Q8C093 (Q8C093) Hypothetical riboflavin synthase domain-lik	Similar to Q8C093 (Q8C093) Hypothetical riboflavin synthase domain-lik / Gallus gallus finished cDNA, clone ChEST868e9 / Gallus gallus finished cDNA, clone ChEST868e9 / Finished cDNA, clone ChEST629b10 / --- / PREDICTED: Gallus gallus similar to MGC68903 protein (LOC420644), mRNA. / PREDICTED: similar to MGC68903 protein; / --- / --- / Gallus gallus finished cDNA, clone ChEST868e9 / Finished cDNA, clone ChEST629b10
RIGG09542	2.599	0.00377	TTGGAGAAGTGGAAATTCCATTTGATTCCTGGAACTTTGAAAATCAGGGCGATGAGTGGTTTGTGCTCCA		ENSGALG00000016258.1 / --- / --- / --- / / --- / --- / --- / synaptotagmin-like 5; / --- / ---
RIGG01245	2.575	0.0012	CCCCGACCCCAATCGGTATGGATTTGTCGCTAGTAAAAGAAATGTTAGAGCATGCAAATTTACAACAGCT	Weakly similar to Q98U09 (Q98U09) Env	Weakly similar to Q98U09 (Q98U09) Env / Gallus gallus finished cDNA, clone ChEST173l1 / Gallus gallus finished cDNA, clone ChEST173l1 / Finished cDNA, clone ChEST173l1 / --- / --- / --- / --- / --- / Gallus gallus finished cDNA, clone ChEST173l1 / Finished cDNA, clone ChEST173l1
RIGG05893	2.567	0.0399	GTAGTGTTTCATGGGTTGGTGGTTCCGTATGGAGGCTCGGAGTTACTGTGTTAAAGGATACAGGCTCTTC	Genome Hit Contig13.87	Genome Hit Contig13.87 / --- / Gallus gallus finished cDNA, clone ChEST817c22 / Finished cDNA, clone ChEST817c22 / --- / --- / --- / --- / --- / Gallus gallus finished cDNA, clone ChEST817c22 / Finished cDNA, clone ChEST817c22

Gene Name	Fold Change	p-value	SEQUENCE	Array Description	Blast/Database Description
RIGG11363	2.567	0.00127	CCGTCATTAAGAAGCAAAGCTCCTACAGGTTCTACTCCAGCTCCCTTCTCATCATTTACGACGGACTGGA		ENSGALT00000004543.1 / similar to UP|Q5TAQ4_HUMAN (Q5TAQ4) Inositol hexaphosphate kinase 3, partial (53%) / 603221964F1 CSEQRBN10 Gallus gallus cDNA clone ChEST217p16 5', mRNA sequence / Transcribed locus, weakly similar to NP_492519.2 F30A10.3 [Caenorhabditis elegans] / hypothetical protein / --- / --- / --- / chromosome 6 open reading frame 125; / 603221964F1 CSEQRBN10 Gallus gallus cDNA clone ChEST217p16 5', mRNA sequence / Transcribed locus, weakly similar to NP_492519.2 F30A10.3 [Caenorhabditis elegans]
RIGG04049	2.566	0.0271	ATGGCTTTATACCTCCGCACAAGTCTACCCTCTCAAGTCTTTTGCGATCCTACACCTGTACGCTGAGCAC	Genome Hit Contig25.170	Genome Hit Contig25.170 / --- / 603602362F1 CSEQCHN54 Gallus gallus cDNA clone ChEST579k15 5', mRNA sequence / Finished cDNA, clone ChEST579k15 / --- / --- / --- / --- / --- / 603602362F1 CSEQCHN54 Gallus gallus cDNA clone ChEST579k15 5', mRNA sequence / Finished cDNA, clone ChEST579k15
RIGG04765	2.563	0.0195	ACTCCTAGCTCAGTTTTGTCCATGCATGTTAGTAGTGAGGTATTTGAGAAGAAAATTACTGCAGTTCATGGACTG	Partial Contig Hit 035207.1	Partial Contig Hit 035207.1 / Gallus gallus finished cDNA, clone ChEST666b10 / Gallus gallus finished cDNA, clone ChEST666b10 / Finished cDNA, clone ChEST666b10 / --- / --- / --- / --- / --- / Gallus gallus finished cDNA, clone ChEST666b10 / Finished cDNA, clone ChEST666b10
RIGG01129	2.558	0.00781	GAATCAAGCAGAGCAAGACAATGTACTTAATCCAGACGGTCACAGCGAAACGCAATTCTTGTATTGTTCTT	Genome Hit Contig9.408	Genome Hit Contig9.408 / Gallus gallus finished cDNA, clone ChEST152o10 / --- / --- / --- / --- / --- / --- / --- / --- / ---
RIGG01728	2.558	0.0282	TAACTCAGGTCTTTTCTTTCGATCCCGCTTGTTCCGTAGGTCACTCGAAACCTGAAGTGAGCAAACATGA	Similar to Q8NE10 (Q8NE10) Hypothetical protein	Similar to Q8NE10 (Q8NE10) Hypothetical protein / Gallus gallus finished cDNA, clone ChEST234c12 / esa006_e11 Eimeiria tenella-infected caecal tonsil Gallus gallus cDNA, mRNA sequence / Finished cDNA, clone ChEST234c12 / --- / --- / --- / Finished cDNA, clone ChEST234c12; / --- / --- / ---
RIGG00344	2.549	0.0368	AGGACTCCTCCAAGCCTCAAAGCGACTTTTGGATGAAGGTGGATTCCTAAAAGACTGAAACAGAATAGAC	Gallus gallus mRNA for hypothetical protein, clone 33e6	Gallus gallus mRNA for hypothetical protein, clone 33e6 / UP|Q7SZH6_CHICK (Q7SZH6) Fanconi anemia group G protein, partial (98%) / Gallus gallus FANCG mRNA for Fanconi anemia group G protein, complete cds / Hypothetical protein, clone 33e6 / FANCG: Fanconi anemia group G protein / --- / --- / --- / --- / Gallus gallus FANCG mRNA for Fanconi anemia group G protein, complete cds / Hypothetical protein, clone 33e6
RIGG16955	2.546	0.0362	TCAAGCTAAGGGCATTTCTTCAGATTGGTGTTTCTGTGCTCCTTGCTAGCAGATAAGACCATGAATGTGGT		ENSGALT00000020582.1 / UP|HBLD2_CHICK (Q5ZJ74) HESB-like domain-containing protein 2, mitochondrial precursor (Iron sulfur assembly protein IscA), complete / Gallus gallus mRNA for hypothetical protein, clone 20e4 / Hypothetical protein, clone 20e4 / HBLD2, RCJMB04_20e4: HESB-like domain-containing protein 2, mitochondrial precursor / --- / --- / Hypothetical protein, clone 20e4; / --- / Gallus gallus mRNA for hypothetical protein, clone 20e4 / Hypothetical protein, clone 20e4

Gene Name	Fold Change	p-value	SEQUENCE	Array Description	Blast/Database Description
RIGG03074	2.533	0.0144	TAATGCTAGACTCTACTGATGTCAA CCTGTGGTATAAAATTGGTCGTCTG GCAATAAAACTCATCCGCCT	Weakly similar to CABI_RAT (O88480) Calcineurin-binding protein Cabin 1 (Calc	Weakly similar to CABI_RAT (O88480) Calcineurin-binding protein Cabin 1 (Calc / Gallus gallus finished cDNA, clone ChEST432o15 / Gallus gallus finished cDNA, clone ChEST432o15 / Finished cDNA, clone ChEST432o15 / similar to cain / --- / --- / --- / --- / Gallus gallus finished cDNA, clone ChEST432o15 / Finished cDNA, clone ChEST432o15
RIGG00730	2.533	0.0401	CGCATTGAGTTTGAGAATGTGCACT TCAGCTACATCGATGGGAAGGAAAT CCTGCAGGACATCTC	Similar to Q8G286 (Q8G286) ABC transporter, ATP-binding/permease prote	Similar to Q8G286 (Q8G286) ABC transporter, ATP-binding/permease prote / Gallus gallus finished cDNA, clone ChEST1021o14 / Gallus gallus finished cDNA, clone ChEST1021o14 / Finished cDNA, clone ChEST1021o14 / similar to ATP-binding cassette, sub-family B (MDR/TAP), member 7 / PREDICTED: Gallus gallus similar to ATP-binding cassette, sub-family B, member 7; ATP-binding cassette 7; Anemia, sideroblastic, with spinocerebellar ataxia (LOC422326), mRNA. / PREDICTED: similar to ATP-binding cassette, sub-family B, member 7; ATP-binding cassette 7; Anemia, sideroblastic, with spinocerebellar ataxia; / --- / --- / Gallus gallus finished cDNA, clone ChEST1021o14 / Finished cDNA, clone ChEST1021o14
RIGG04148	2.532	0.00481	CTGTTCTTCATCCCTCTGTGTGCCT GTATGTAACACCAGGGTCTCTCTGT TTGATTAAAGGCACGCCTCT	Similar to Q91YY4 (Q91YY4) Hypothetical 33.3 kDa protein	Similar to Q91YY4 (Q91YY4) Hypothetical 33.3 kDa protein / Gallus gallus finished cDNA, clone ChEST591a12 / 603607241F1 CSEQCHN55 Gallus gallus cDNA clone ChEST591a12 5', mRNA sequence / Finished cDNA, clone ChEST591a12 / --- / PREDICTED: Gallus gallus similar to ATP synthase mitochondrial F1 complex assembly factor 2 (LOC416511), mRNA. / PREDICTED: similar to ATP synthase mitochondrial F1 complex assembly factor 2; / --- / --- / 603607241F1 CSEQCHN55 Gallus gallus cDNA clone ChEST591a12 5', mRNA sequence / Finished cDNA, clone ChEST591a12
RIGG17291	2.531	0.0285	ACAGCGCTAATGATGCTATTGGCAA AGTGTACATTGATATTGATCCTTTGC TCTATAGTGAAGCAGCTACGGTTA		ENSGALT00000021579.1 / --- / --- / --- / --- / --- / --- / Finished cDNA, clone ChEST812a1;Transcribed locus, weakly similar to NP_651249.1 CG6454-PB, isoform B [Drosophila melanogaster]; / KIAA0528; / 603816801F1 CSEQCHN52 Gallus gallus cDNA clone ChEST812a1 5', mRNA sequence / Finished cDNA, clone ChEST812a1
RIGG14987	2.527	0.00851	AAAAGTCAAGCTTGGGTTTGGATTT TAGTTCCTTGGTTCCCTGTGCTTTGT ACGTTTTGGTCCTTGGCCC	lefty [Gallus gallus]. [Source:RefSeq;Acc:NM_204764]	lefty [Gallus gallus]. [Source:RefSeq / UP|Q9PVN4_CHICK (Q9PVN4) Lefty, complete / Gallus gallus left-right determination factor 2 (LEFTY2), mRNA / LEFTY-1 protein (LEFTY-1) / Lefty / Gallus gallus lefty (LOC395529), mRNA. / lefty; / LEFTY-1 protein (LEFTY-1); / --- / Gallus gallus left-right determination factor 2 (LEFTY2), mRNA / LEFTY-1 protein (LEFTY-1)

Gene Name	Fold Change	p-value	SEQUENCE	Array Description	Blast/Database Description
RIGG13784	2.524	0.00403	GTACAATTGATGGGACCAAGGACGAAAACAGCGACTACACCCTTTTCAACCTAATTCCTGTGGGTCTTCG	fibroblast growth factor 12 isoform A [Gallus gallus]. [Source:RefSeq;Acc:NM_204888]	fibroblast growth factor 12 isoform A [Gallus gallus]. [Source:RefSeq / UP|Q9W6A1_CHICK (Q9W6A1) Fibroblast growth factor 12 isoform A, complete / 603143456F1 CSEQCHL16 Gallus gallus cDNA clone ChEST138p13 5', mRNA sequence / Fibroblast growth factor 12 isoform A (FGF12) / FGF12: Fibroblast growth factor 12 isoform A / Gallus gallus fibroblast growth factor 12 isoform A (FGF12), mRNA. / fibroblast growth factor 12 isoform A; / --- / --- / 603143456F1 CSEQCHL16 Gallus gallus cDNA clone ChEST138p13 5', mRNA sequence / Fibroblast growth factor 12 isoform A (FGF12)
RIGG09541	2.524	0.00293	CCTGGCTACAGTCCATCTTCACAAACTTCCCTGCCTTGCTCAACTTTGTGAACAAAATGAAGTGTGTCAG		ENSGALG00000016257.1 / UP|Q4F8N2_CHICK (Q4F8N2) Otoconin, complete / Gallus gallus otoconin mRNA, complete cds / Otoconin 90 (OC90), mRNA / Otoconin / --- / --- / Otoconin 90 (OC90), mRNA; / --- / Gallus gallus otoconin mRNA, complete cds / Otoconin 90 (OC90), mRNA
RIGG01997	2.521	0.043	TGGCGGAGTAGCCTGTGGACTTCAGCACAACTATCAACATTGCTGTTCAAGATATTACAATTTATGTCCA	Same gene M18355; Chicken calmodulin-like protein (neoCaM) mRNA, partial cds	Same gene M18355; Chicken calmodulin-like protein (neoCaM) mRNA, partial cds / UP|Q3UKW2_MOUSE (Q3UKW2) 12 days pregnant adult female placenta cDNA, RIKEN full-length enriched library, clone:I530005B05 product:calmodulin 1, full insert sequence, partial (61%) / gPGC_EST00900 Embryonic gonadal PGC cDNA Library Gallus gallus cDNA 5', mRNA sequence / Calmodulin / Calmodulin / PREDICTED: Gallus gallus calmodulin-like protein (neoCaM) (LOC396523), mRNA. / PREDICTED: similar to calmodulin 1; Calmodulin 1 (phosphorylase kinase, delta); Calmodulin 1 (phosphorylase kinase delta); / --- / --- / --- / ---
RIGG02093	2.512	0.0411	ACAAGGTTAAGACTGTGTGCTGACATTCTTTTCAACCCTGAACTTGAATGTTAAGTTCTTCGTCCTGCCTG	Weakly similar to Q96EP3 (Q96EP3) Hypothetical protein	Weakly similar to Q96EP3 (Q96EP3) Hypothetical protein / Gallus gallus finished cDNA, clone ChEST618n3 / Gallus gallus finished cDNA, clone ChEST618n3 / Finished cDNA, clone ChEST283k10 / --- / PREDICTED: Gallus gallus similar to Hypothetical protein MGC75678 (LOC426392), mRNA.PREDICTED: Gallus gallus similar to Hypothetical protein MGC75678 (LOC426395), mRNA. / PREDICTED: similar to Hypothetical protein MGC75678;PREDICTED: similar to Hypothetical protein MGC75678; / --- / --- / Gallus gallus finished cDNA, clone ChEST618n3 / Finished cDNA, clone ChEST283k10
RIGG01980	2.506	0.0209	TATCTCCTTAAGGAGCACAGAGTGATTTCTTTGGCTGCAGGAATACATGGCTGGGAAGGTTACACCCTGC	Genome Hit Contig192.43	Genome Hit Contig192.43 / weakly similar to UP|Q370B2_RHOPA (Q370B2) IMP dehydrogenase/GMP reductase, partial (3%) / 603367115F1 CSEQRBN19 Gallus gallus cDNA clone ChEST268m3 5', mRNA sequence / Finished cDNA, clone ChEST268m3 / --- / --- / --- / --- / --- / 603367115F1 CSEQRBN19 Gallus gallus cDNA clone ChEST268m3 5', mRNA sequence / Finished cDNA, clone ChEST268m3

Gene Name	Fold Change	p-value	SEQUENCE	Array Description	Blast/Database Description
RIGG05640	2.496	0.0334	AAAGTCTTCTGCAAGATCTTTACCTCCTTCAGGAAGGGCTTAGCTATCTCCACGTTGAAAAGAAAAGGAC	Genome Hit Contig96.34	Genome Hit Contig96.34 / Gallus gallus finished cDNA, clone ChEST784k21 / Gallus gallus finished cDNA, clone ChEST784k21 / Finished cDNA, clone ChEST784k21 / --- / --- / --- / --- / --- / Gallus gallus finished cDNA, clone ChEST784k21 / Finished cDNA, clone ChEST784k21
RIGG03467	2.488	0.00234	TGTCCAGGTTTAATTGGCAGCGATTGTTTTGTCTTCTGTACAAAAGCAGCAAATCCCCAGAAGCTGGTCA	Genome Hit Contig50.142	Genome Hit Contig50.142 / Gallus gallus finished cDNA, clone ChEST49j22 / Gallus gallus finished cDNA, clone ChEST49j22 / Finished cDNA, clone ChEST49j22 / --- / --- / --- / --- / --- / Gallus gallus finished cDNA, clone ChEST49j22 / Finished cDNA, clone ChEST49j22
RIGG17932	2.483	0.0451	CATGTGCAGTATTATCTCCTGTGCTCTCTCAATACTCTTTGGTGACAAAAGTTCACTGTGAGGGACCTCT		ENSGALT00000023257.1 / Gallus gallus finished cDNA, clone ChEST195h23 / Gallus gallus finished cDNA, clone ChEST195h23 / Finished cDNA, clone ChEST195h23 / --- / --- / --- / --- / --- / Gallus gallus finished cDNA, clone ChEST195h23 / Finished cDNA, clone ChEST195h23
RIGG01498	2.476	0.0226	CGAGTCAGGGGTGGTATTTAATGTTTGATATCTCAGTTGTTTCTGAATCTGCTTTCTTCCCCAAATGCCAG	Genome Hit Contig103.107	Genome Hit Contig103.107 / Gallus gallus finished cDNA, clone ChEST205o17 / Gallus gallus finished cDNA, clone ChEST205o17 / Finished cDNA, clone ChEST205o17 / --- / --- / --- / --- / --- / Gallus gallus finished cDNA, clone ChEST205o17 / Finished cDNA, clone ChEST205o17
RIGG04374	2.475	0.0463	ACTCCATCCTGAACTCCCCTTATGCTAACCTCTACAATCCAGAAAACATCTATCTGTCTGAGCATGGAGG	Similar to TBG_CHLRE (Q39582) Tubulin gamma chain (Gamma tubulin)	Similar to TBG_CHLRE (Q39582) Tubulin gamma chain (Gamma tubulin) / UP|Q4QQZ8_XENLA (Q4QQZ8) Xgam protein, partial (98%) / Gallus gallus finished cDNA, clone ChEST653f16 / Finished cDNA, clone ChEST621o6 / similar to Xgam protein / PREDICTED: Gallus gallus similar to tubulin gamma chain - African clawed frog (LOC420025), mRNA. / PREDICTED: similar to tubulin gamma chain - African clawed frog; / --- / --- / Gallus gallus finished cDNA, clone ChEST653f16 / Finished cDNA, clone ChEST621o6
RIGG00835	2.474	0.048	CCTTACATGTTGGGAACTGGACTCCTGCTCTACTTACTCTCTAAGGAAATCTATGTCATTAACCATGAGACAGTT	Similar to Q9IAJ7 (Q9IAJ7) ATP synthase subunit B (Similar to ATP synt	Similar to Q9IAJ7 (Q9IAJ7) ATP synthase subunit B (Similar to ATP synt / Gallus gallus finished cDNA, clone ChEST1031e5 / 603539569F1 CSEQCHN60 Gallus gallus cDNA clone ChEST506j18 5', mRNA sequence / Transcribed locus, weakly similar to XP_322024.2 ENSANGP00000012069 [Anopheles gambiae str. PEST] / / PREDICTED: Gallus gallus similar to ATP synthase subunit B (LOC419866), mRNA. / PREDICTED: similar to ATP synthase subunit B; / Finished cDNA, clone ChEST130j14; / --- / 603150035F1 CSEQCHL19 Gallus gallus cDNA clone ChEST155e10 5', mRNA sequence / Finished cDNA, clone ChEST130j14
RIGG01435	2.473	0.00567	TTCGTGAAGCCAACTGAGCCGTGCCATCACCTGAGGATTTATAACCACAGAGAGAACATTTCCAGTAATG	Genome Hit Contig42.81	Genome Hit Contig42.81 / Gallus gallus finished cDNA, clone ChEST199p20 / 603214780F1 CSEQRBN14 Gallus gallus cDNA clone ChEST199p20 5', mRNA sequence / Finished cDNA, clone ChEST199p20 / --- / --- / --- / --- / --- / 603214780F1 CSEQRBN14 Gallus gallus cDNA clone ChEST199p20 5', mRNA sequence / Finished cDNA, clone ChEST199p20

Gene Name	Fold Change	p-value	SEQUENCE	Array Description	Blast/Database Description
RIGG16925	2.469	0.0376	TCCACGGTGGAGAGACCTTCTGTGTTCAACTACACCTGGATTGTCCCCATCACCTGGATGACGCC		ENSGALT00000020495.1 / UP|O57579_CHICK (O57579) Aminopeptidase Ey, complete / Gallus gallus alanyl (membrane) aminopeptidase (aminopeptidase N, aminopeptidase M, microsomal aminopeptidase, CD13, p150) (ANPEP), mRNA / Aminopeptidase Ey / / --- / --- / --- / --- / Gallus gallus alanyl (membrane) aminopeptidase (aminopeptidase N, aminopeptidase M, microsomal aminopeptidase, CD13, p150) (ANPEP), mRNA / Aminopeptidase Ey
RIGG03962	2.464	0.00185	CGGGCGAAACTGAGTTACTGCGCTGATAAGGGAGATAATGCAACTTTTATTATTTTGACCTCATCTCCTGTCT	Genome Hit Contig44.44	Genome Hit Contig44.44 / --- / 603597354F1 CSEQCHN73 Gallus gallus cDNA clone ChEST566l14 5', mRNA sequence / Finished cDNA, clone ChEST566l14 / --- / --- / --- / --- / --- / 603597354F1 CSEQCHN73 Gallus gallus cDNA clone ChEST566l14 5', mRNA sequence / Finished cDNA, clone ChEST566l14
RIGG08921	2.46	0.023	TTCCATGTGGTTGCACTAAAGAAGGGTGTAGCAATACAGCAGGTAGAATTGAATTTAATCCTATCCGTGT		ENSGALG00000010971.1 / --- / --- / --- / --- / --- / --- / --- / --- / --- / ---
RIGG14387	2.459	0.0345	GGATTTCAATGAAGACTATTCTGATCTCGATGGAATAGTACAGGAGCAGAGACAAGAGATGGAGGAGTCCA		ENSGALT00000013404.1 / Gallus gallus finished cDNA, clone ChEST153j8 / Gallus gallus finished cDNA, clone ChEST153j8 / Finished cDNA, clone ChEST153j8 / --- / PREDICTED: Gallus gallus similar to HCV NS3-transactivated protein 2 (LOC426813), mRNA. / PREDICTED: similar to HCV NS3-transactivated protein 2; / --- / --- / Gallus gallus finished cDNA, clone ChEST153j8 / Finished cDNA, clone ChEST153j8
RIGG00423	2.458	0.0221	GATGCAGAACCCTTGGATTTTAATGCAGACTCAATTAATGATGACCCTCTTGAATCGGACTCTGGAAGGTACCAG	Gallus gallus mRNA for hypothetical protein, clone 18o10	Gallus gallus mRNA for hypothetical protein, clone 18o10 / RF|NP_001026750.1|71896103|NM_001031579 LMBR1 domain containing 2 {Gallus gallus} (exp=-1; wgp=0; cg=0), complete / Gallus gallus mRNA for hypothetical protein, clone 18o10 / LMBR1 domain containing 2 (LMBRD2), mRNA / --- / PREDICTED: Gallus gallus similar to RIKEN cDNA 9930036E21 gene (LOC427436), mRNA.PREDICTED: Gallus gallus similar to RIKEN cDNA 9930036E21 gene (LOC429640), partial mRNA. / PREDICTED: similar to RIKEN cDNA 9930036E21 gene;PREDICTED: similar to RIKEN cDNA 9930036E21 gene, partial; / --- / --- / Gallus gallus mRNA for hypothetical protein, clone 18o10 / LMBR1 domain containing 2 (LMBRD2), mRNA
RIGG00601	2.444	0.031	TGAAGGGTTACACGTTTTGAGGAAGATAATAAATGTGCCCTAAGTGACAGAGCTGACATCTAGAAATCTGGA	Partial Contig Hit 506362.1	Partial Contig Hit 506362.1 / Gallus gallus finished cDNA, clone ChEST1010a21 / Gallus gallus finished cDNA, clone ChEST1010a21 / Finished cDNA, clone ChEST1010a21 / --- / --- / --- / --- / --- / Gallus gallus finished cDNA, clone ChEST1010a21 / Finished cDNA, clone ChEST1010a21

Gene Name	Fold Change	p-value	SEQUENCE	Array Description	Blast/Database Description
RIGG03704	2.443	0.00523	CAATGGTGGTAACTGGGTCAACTGCTTTCTCCTGCTGAATGTAGCTCCCAAGCATATACAGCTGTCTCTC	Weakly similar to EAA15114 (EAA15114) ENSANGP00000010651 (Fragment)	Weakly similar to EAA15114 (EAA15114) ENSANGP00000010651 (Fragment) / Gallus gallus finished cDNA, clone ChEST536o20 / Gallus gallus finished cDNA, clone ChEST536o20 / Finished cDNA, clone ChEST536o20 / similar to ring finger protein 123 / --- / --- / Transcribed locus, strongly similar to XP_414267.1 PREDICTED: similar to ring finger protein 123 [Gallus gallus];Transcribed locus, weakly similar to NP_650425.1 CG6752-PA [Drosophila melanogaster]; / ring finger protein 123; / Gallus gallus finished cDNA, clone ChEST536o20 / Finished cDNA, clone ChEST536o20
RIGG02914	2.441	0.0148	AAGGTTGCTTCGCTTACCCACTGCTGCTTTTGATTTTGGCCTGGATGGAATAGTAACTGATAGTCTGCGG	Genome Hit Contig56.100	Genome Hit Contig56.100 / Gallus gallus finished cDNA, clone ChEST405o1 / Gallus gallus finished cDNA, clone ChEST405o1 / Finished cDNA, clone ChEST405o1 / --- / --- / --- / --- / --- / Gallus gallus finished cDNA, clone ChEST405o1 / Finished cDNA, clone ChEST405o1
RIGG02552	2.431	0.000166	CCAACGTGCTCAGTACCCACAAATAAAGGTGTTTGTTTTATCACTCCCATTTTAATGGTGAGGAAACTGC	Weakly similar to Q9DVW0 (Q9DVW0) PxORF73 peptide	Weakly similar to Q9DVW0 (Q9DVW0) PxORF73 peptide / Gallus gallus finished cDNA, clone ChEST361d18 / Gallus gallus Frizzled-2 (cFz-2) mRNA, partial cds / FZ-2 mRNA for frizzled-2 / --- / --- / --- / --- / proline rich 11; / --- / ---
RIGG11862	2.419	0.0171	CGTATGTGTTGGTGTTTTGTACAACAGACAATTACCAAAGTCTCCGCAAGAGGAAGATGTTTGGGAGTTG		ENSGALT00000005993.1 / similar to UP|Q4VB91_HUMAN (Q4VB91) NELL1 protein, partial (20%) / 603785128F1 CSEQCHN72 Gallus gallus cDNA clone ChEST739h7 5', mRNA sequence / Transcribed locus, moderately similar to NP_112331.1 NEL-like 1 [Rattus norvegicus] / --- / --- / --- / --- / NEL-like 1 (chicken); / 603785128F1 CSEQCHN72 Gallus gallus cDNA clone ChEST739h7 5', mRNA sequence / Transcribed locus, moderately similar to NP_112331.1 NEL-like 1 [Rattus norvegicus]
RIGG16832	2.417	0.0233	GGTTTTCACTTCGACGCGGACGAACTGTTCCACGTGGAGTTGGACGCCGCTCAGACCGTATGGAG		ENSGALT00000020245.1 / UP|Q4U5Z6_CHICK (Q4U5Z6) MHC class II alpha chain, complete / Gallus gallus MHC class II alpha chain (B-LA) mRNA, B-LA-B21 allele, complete cds / MHC class II antigen / hypothetical protein LOC430189, partial / --- / --- / --- / --- / Gallus gallus clone 9 MHC class II antigen mRNA, complete cds / MHC class II antigen alpha (B-LA), mRNA
RIGG14888	2.415	0.0246	GGGTGTGTGGAGAGATGCTATATGACACCTTTTCTGGCTTAAACAAAACCTTTGAGAAAGCATCTCTCAT		ENSGALT00000014774.1 / Gallus gallus finished cDNA, clone ChEST710o24 / --- / --- / similar to Mannosidase, alpha, class 1B, member 1 / PREDICTED: Gallus gallus similar to Endoplasmic reticulum mannosyl-oligosaccharide 1,2-alpha-mannosidase (ER alpha-1,2-mannosidase) (Mannosidase alpha class 1B member 1) (Man9GlcNAc2-specific processing alpha-mannosidase) (UNQ747/PRO1477) (LOC417296), mRN / PREDICTED: similar to Endoplasmic reticulum mannosyl-oligosaccharide 1,2-alpha-mannosidase (ER alpha-1,2-mannosidase) (Mannosidase alpha class 1B member 1) (Man9GlcNAc2-specific processing alpha-mannosidase) (UNQ747/PRO1477); / --- / --- / --- / ---

Gene Name	Fold Change	p-value	SEQUENCE	Array Description	Blast/Database Description
RIGG00202	2.41	0.0473	GTGTGACCAAGTTTCCAACAGGGATGATGTCTTGCATTTGCCATGGAACAAACTTTCCCAAATGCCCACC	Gallus gallus mRNA for hypothetical protein, clone 13g19	Gallus gallus mRNA for hypothetical protein, clone 13g19 / similar to UP|SFRP1_CHICK (Q9DEQ4) Secreted frizzled-related protein 1 precursor (sFRP-1) (CsFRP1), partial (47%) / --- / --- / --- / --- / --- / --- / --- / --- / ---
RIGG03237	2.41	0.0325	GCTGCAGAGTGCAATTACATTACAGGCTTTTAAGCTGTTTTCATTTGGGCTTATACATCCCCATGCTATT	Weakly similar to RW1_MOUSE (O70472) RW1 protein	Weakly similar to RW1_MOUSE (O70472) RW1 protein / Gallus gallus finished cDNA, clone ChEST464h16 / Gallus gallus mRNA for hypothetical protein, clone 17n23 / Hypothetical protein, clone 17n23 / --- / PREDICTED: Gallus gallus similar to RW1 protein (LOC418689), mRNA. / PREDICTED: similar to RW1 protein; / --- / --- / --- / ---
RIGG00665	2.403	0.024	GTCGTCTCGTACAAAGATTCACAACAGTGTCCCAGACAAAACCTCACCAAAGCACTTTGACAAGAACGCTTA	Similar to CLDA_MOUSE (Q9Z0S6) Claudin-10	Similar to CLDA_MOUSE (Q9Z0S6) Claudin-10 / Gallus gallus finished cDNA, clone ChEST1015c6 / Gallus gallus finished cDNA, clone ChEST631g17 / Finished cDNA, clone ChEST631g17 / similar to claudin 10 isoform 2 / --- / --- / --- / claudin 10; / Gallus gallus finished cDNA, clone ChEST631g17 / Finished cDNA, clone ChEST631g17
RIGG17537	2.401	0.0235	CATGCACCAAAGAACTCTGAAATCTCTCATTGAATTATACCATCTGCTTCGGGTCAGCCAACAAGAACTG		ENSGALT00000022283.1 / similar to UP|Q4S229_TETNG (Q4S229) Chromosome undetermined SCAF14764, whole genome shotgun sequence, partial (30%) / 603005172F1 CSEQCHL01 Gallus gallus cDNA clone ChEST21p18 5', mRNA sequence / Transcribed locus, weakly similar to XP_001061354.1 PREDICTED: similar to CG12038-PA, isoform A [Rattus norvegicus] / --- / PREDICTED: Gallus gallus similar to hypothetical protein B230399E16 (LOC421014), mRNA. / PREDICTED: similar to hypothetical protein B230399E16; / Transcribed locus, weakly similar to XP_001061354.1 PREDICTED: similar to CG12038-PA, isoform A [Rattus norvegicus]; / --- / 603005172F1 CSEQCHL01 Gallus gallus cDNA clone ChEST21p18 5', mRNA sequence / Transcribed locus, weakly similar to XP_001061354.1 PREDICTED: similar to CG12038-PA, isoform A [Rattus norvegicus]
RIGG01189	2.4	0.0103	AAGTCTGAGGATGCCTTGGGGATCAGCTACCCTATTTCTGCTGCCATGTATCAAGTTTGCCTGAAGTACT	Weakly similar to Q9Y6X4 (Q9Y6X4) Hypothetical protein KIAA0888 (Fragment)	Weakly similar to Q9Y6X4 (Q9Y6X4) Hypothetical protein KIAA0888 (Fragment) / Gallus gallus finished cDNA, clone ChEST163l2 / gonad_EST07908 Embryonic gonad cDNA Library Gallus gallus cDNA 5', mRNA sequence / Finished cDNA, clone ChEST604j16 / --- / PREDICTED: Gallus gallus similar to hypothetical protein FLJ39743 (LOC415511), mRNA. / PREDICTED: similar to hypothetical protein FLJ39743; / --- / --- / gonad_EST07908 Embryonic gonad cDNA Library Gallus gallus cDNA 5', mRNA sequence / Finished cDNA, clone ChEST604j16
RIGG02928	2.394	0.0487	ACAGAGTACAAAGTAAATGTGAGTGTGTTGGTTACAGGTGCCATAAACCTCCTACGAAAGGTCACCACGG	Genome Hit Contig52.54	Genome Hit Contig52.54 / Gallus gallus finished cDNA, clone ChEST40a17 / --- / --- / --- / --- / --- / --- / --- / Gallus gallus finished cDNA, clone ChEST541j20 / Finished cDNA, clone ChEST40a17

Gene Name	Fold Change	p-value	SEQUENCE	Array Description	Blast/Database Description
RIGG06664	2.394	0.00984	GAGTGCTCCCTAGTTTCTTTACCAG CTGCTACAGTATGTTATTACTTACTT CTCTATGGCAGTGACCTGTG	Genome Hit Contig8.321	Genome Hit Contig8.321 / homologue to GB|AAH32862.1|23273934|BC032862 SOCS5 protein {Homo sapiens} (exp=-1; wgp=0; cg=0), partial (9%) / Gallus gallus finished cDNA, clone ChEST924c14 / Finished cDNA, clone ChEST924c14 / --- / --- / --- / --- / --- / Gallus gallus finished cDNA, clone ChEST924c14 / Finished cDNA, clone ChEST924c14
RIGG09514	2.381	0.0151	AGTGAAGGAGTATGGTCTTCCAGAC GTGGACATCTTGTTGTTCCAGAACA TTGATGGGAAAGAGTTGTGTA	Transcriptional regulator Erg. [Source:SWISSPROT;Acc:Q90837]	Transcriptional regulator Erg. [Source:SWISSPROT / UP|Q8UUU0_CHICK (Q8UUU0) Erg isoform C-1-1, complete / Gallus gallus v-ets erythroblastosis virus E26 oncogene like (avian) (ERG), mRNA / ERG mRNA / Erg isoform C-1-1 / --- / --- / --- / --- / Gallus gallus v-ets erythroblastosis virus E26 oncogene like (avian) (ERG), mRNA / ERG mRNA
RIGG08150	2.375	0.0259	CAAAAGGAGACATTGGCGTCAGCAC CATCCTTGGATCAGCCATCTACAAT CTTCTTGGGATTTGTGCAGC		ENSGALG00000004885.1 / solute carrier family 24, member 5 [Gallus gallus] / 603134006F1 CSEQCHL24 Gallus gallus cDNA clone ChEST115k2 5', mRNA sequence / Finished cDNA, clone ChEST36i19 / / --- / --- / --- / --- / --- / ---
RIGG19381	2.375	0.0472	CCTTCAGCCCTGAACAACTTGAACT GATGTTTACTTTCCCACTCATCTACA GCATTTTCCAACTGTTGTT		ENSGALT00000027235.1 / --- / BX268911 AGENAE Gallus gallus multi-tissues normalized and once-subtracted cDNA library (gcal) Gallus gallus cDNA clone gcal0012.o.04 3prim, mRNA sequence / Transcribed locus, strongly similar to XP_426338.1 PREDICTED: similar to solute carrier family 10 (sodium/bile acid cotransporter family), member 4 [Gallus gallus] / hypothetical protein / PREDICTED: Gallus gallus similar to Ileal sodium/bile acid cotransporter (Ileal Na(+)/bile acid cotransporter) (Na(+) dependent ileal bile acid transporter) (Ileal sodium-dependent bile acid transporter) (ISBT) (Sodium/taurocholate cotransporting polypept / PREDICTED: similar to Ileal sodium/bile acid cotransporter (Ileal Na(+)/bile acid cotransporter) (Na(+) dependent ileal bile acid transporter) (Ileal sodium-dependent bile acid transporter) (ISBT) (Sodium/taurocholate cotransporting polypeptide, ileal)...; / --- / --- / --- / ---
RIGG13911	2.37	0.0495	GCAACAGGTTATGGAAATGCATTAG TTTGCCTCAAACCACAGTTTCTCTTG AATTTGAGGGATGATCTGC		ENSGALT00000012019.1 / --- / pgn1c.pk011.k12 Chicken lymphoid cDNA library (pgn1c) Gallus gallus cDNA clone pgn1c.pk011.k12 5' similar to no significant hits (pLog(P) 4), mRNA sequence / Transcribed locus / --- / --- / --- / --- / --- / pgn1c.pk011.k12 Chicken lymphoid cDNA library (pgn1c) Gallus gallus cDNA clone pgn1c.pk011.k12 5' similar to no significant hits (pLog(P) 4), mRNA sequence / Transcribed locus

Gene Name	Fold Change	p-value	SEQUENCE	Array Description	Blast/Database Description
RIGG03637	2.368	0.05	GTCCTTCATCGAATACATCAAGAGCCAACCAATTGTATTTGAGGTCTTCGGGCACTACCAACAGCACCCC	Similar to Q8R524 (Q8R524) Kinesin-family protein 1Bp204	Similar to Q8R524 (Q8R524) Kinesin-family protein 1Bp204 / Gallus gallus finished cDNA, clone ChEST52m22 / GGEZHT1007A03.g HT1 Gallus gallus cDNA clone GGEZHT1007A03, mRNA sequence / Finished cDNA, clone ChEST52m22 / --- / PREDICTED: Gallus gallus similar to kinesin-related microtubule-based motor protein (LOC424849), mRNA. / PREDICTED: similar to kinesin-related microtubule-based motor protein; / Finished cDNA, clone ChEST52m22;Transcribed locus, weakly similar to XP_514364.1 PREDICTED: similar to KIAA1448 protein [Pan troglodytes]; / --- / --- / ---
RIGG03167	2.355	0.00905	GACAGTGGACCTGCAAATGCAACAAGAAACAGGAAGCTCTTCTTTAACGTCCGTTGTTGCCTTCGTCCTC	Contig Hit 045891.3	Contig Hit 045891.3 / Gallus gallus finished cDNA, clone ChEST445i1 / 603511705F1 CSEQCHN57 Gallus gallus cDNA clone ChEST445i1 5', mRNA sequence / Finished cDNA, clone ChEST445i1 / --- / --- / --- / --- / --- / 603511705F1 CSEQCHN57 Gallus gallus cDNA clone ChEST445i1 5', mRNA sequence / Finished cDNA, clone ChEST445i1
RIGG18418	2.351	0.0139	GGAACATGTTTTCTCCTTCCTTCAACTCAGGTTAAGAATTTGCTGGACATGCATGTCTTGGCCTTCATTC		ENSGALT00000024584.1 / --- / Gallus gallus mRNA for hypothetical protein, clone 7i18 / Chloride channel 7 (CLCN7), mRNA / / --- / --- / Chloride channel 7 (CLCN7), mRNA; / --- / --- / ---
RIGG01565	2.341	0.00409	ACCAGCACAAAACACGCATCTCTTACCCACCCAATTTCATCATGATAATGCTATTTGTAGGACTGATGAG	Weakly similar to Q9Y3J8 (Q9Y3J8) Hypothetical protein	Weakly similar to Q9Y3J8 (Q9Y3J8) Hypothetical protein / GB|CR386029.1|CR386029.1 Gallus gallus finished cDNA, clone ChEST211j6 / Gallus gallus finished cDNA, clone ChEST234b4 / Finished cDNA, clone ChEST234b4 / --- / PREDICTED: Gallus gallus similar to RIKEN cDNA A530082C11 gene (LOC419405), mRNA. / PREDICTED: similar to RIKEN cDNA A530082C11 gene; / Finished cDNA, clone ChEST234b4; / --- / Gallus gallus finished cDNA, clone ChEST234b4 / Finished cDNA, clone ChEST234b4
RIGG00331	2.337	0.0125	AGGCATGCAGGTCAAAATGTTTCGCATACGTTTTCAACCAACTAAAGGATTTGGGCAGCATCTGAGTTTA	Gallus gallus mRNA for hypothetical protein, clone 32a22	Gallus gallus mRNA for hypothetical protein, clone 32a22 / RF|NP_001026143.1|71896691|NM_001030972 Rap2-binding protein 9 {Gallus gallus} (exp=-1; wgp=0; cg=0), complete / Gallus gallus mRNA for hypothetical protein, clone 32a22 / Rap2-binding protein 9 (LOC420535), mRNA / --- / --- / --- / Rap2-binding protein 9 (LOC420535), mRNA; / --- / Gallus gallus mRNA for hypothetical protein, clone 32a22 / Rap2-binding protein 9 (LOC420535), mRNA
RIGG00295	2.323	0.000383	GTGTCAGCACTGAGTTTGTGCCTTGTAAGTTTGCACTGGCTGTAGATGCGAAACATCATGAGGATACCCAG	Gallus gallus mRNA for hypothetical protein, clone 24f7	Gallus gallus mRNA for hypothetical protein, clone 24f7 / RF|NP_001006378.1|57530453|NM_001006378 vacuolar protein sorting 4B {Gallus gallus} (exp=-1; wgp=0; cg=0), complete / Gallus gallus mRNA for hypothetical protein, clone 24f7 / Vacuolar protein sorting 4 homolog B (S. cerevisiae) (VPS4B), mRNA / RCJMB04_1o9: Hypothetical protein / PREDICTED: Gallus gallus similar to SKD1 (LOC420901), mRNA. / PREDICTED: similar to SKD1; / --- / --- / Gallus gallus mRNA for hypothetical protein, clone 24f7 / Vacuolar protein sorting 4 homolog B (S. cerevisiae) (VPS4B), mRNA

Gene Name	Fold Change	p-value	SEQUENCE	Array Description	Blast/Database Description
RIGG08126	2.317	0.00663	AACTGGAAGCACAAAGATGCAGCTATATACCTTGTTACATCGTTGGCATCCAAGGCTCAGACACAGAAGC		ENSGALG00000004707.1 / --- / --- / --- / similar to cellular apoptosis susceptibility protein / --- / --- / --- / CSE1 chromosome segregation 1-like (yeast); / --- / ---
RIGG03156	2.314	0.0296	TGGTTTGTTGAGGCATTCAGGGTAGGTCTTACATAGTTTTCTTTGTCTTGCACCTTAGAGTCTGAAGAGAAATG	RIKENRP Weakly similar to PC8915	RIKENRP Weakly similar to PC8915 / Gallus gallus finished cDNA, clone ChEST442p13 / 603510856F1 CSEQCHN57 Gallus gallus cDNA clone ChEST442p13 5', mRNA sequence / Finished cDNA, clone ChEST442p13 / --- / --- / --- / --- / --- / 603510856F1 CSEQCHN57 Gallus gallus cDNA clone ChEST442p13 5', mRNA sequence / Finished cDNA, clone ChEST442p13
RIGG05913	2.312	0.00544	CAGCTCCGTTATCTGCACCTTCTGAGTCTGTAATGAATGTTTCTATCCCTTGAAGCCCGTGATGTGTCTG	Genome Hit Contig240.21	Genome Hit Contig240.21 / --- / Gallus gallus finished cDNA, clone ChEST818p16 / Finished cDNA, clone ChEST818p16 / --- / --- / --- / --- / --- / Gallus gallus finished cDNA, clone ChEST818p16 / Finished cDNA, clone ChEST818p16
RIGG19316	2.31	0.0443	CATCTTCAGAACTGCAGAGAGTCATTTCTATGGAGGGAAGACATCTATCTGGATCACAAACGAGCCCATT	Cyclic nucleotide gated channel cone photoreceptor alpha subunit (CNG channel 1) (CNG-1). [Source:SWISSPROT;Acc:Q90805]	Cyclic nucleotide gated channel cone photoreceptor alpha subunit (CNG channel 1) (CNG-1). [Source:SWISSPROT / UP|CNG1_CHICK (Q90805) Cyclic nucleotide-gated channel, cone photoreceptor, subunit alpha (CNG channel 1) (CNG-1), complete / Gallus gallus cyclic nucleotide gated channel alpha 3 (CNGA3), mRNA / Alpha subunit of cone photoreceptor CNG-channel / Cyclic nucleotide-gated channel cone photoreceptor subunit alpha / Gallus gallus alpha subunit of cone photoreceptor CNG-channel (LOC396144), mRNA. / alpha subunit of cone photoreceptor CNG-channel; / Alpha subunit of cone photoreceptor CNG-channel; / --- / Gallus gallus cyclic nucleotide gated channel alpha 3 (CNGA3), mRNA / Alpha subunit of cone photoreceptor CNG-channel
RIGG17863	2.307	0.0196	GACTTTCGTTCTGGATTAGAAAACGGCATTCTTCTGTGTGAGTTGCTGAATGCAATAAAGCCAGGATTGGTC		ENSGALT00000023063.1 / Gallus gallus finished cDNA, clone ChEST639c24 / --- / --- / similar to KIAA1102 protein / --- / hypothetical protein LOC22998; / --- / --- / --- / ---
RIGG13318	2.304	0.0119	GAAAGGTTCCTCGGAATGAATATGGAAACGTCTATCTCTTCCTGCCCTCTATGTTACCCATTGGTTGTGT		ENSGALT00000010275.1 / --- / --- / --- / similar to XP-C repair complementing protein / --- / --- / Transcribed locus, weakly similar to NP_587828.1 hypothetical protein SPCC4G3.10c [Schizosaccharomyces pombe 972h-]; / xeroderma pigmentosum, complementation group C; / --- / ---
RIGG03966	2.302	0.0429	GTTCTTTTAGGGAAGACTTCATACCACAAGGGAAACTTTTAACCCAGTCCTAAGGCACAACGTTTTATGC	Genome Hit Contig230.62	Genome Hit Contig230.62 / --- / Gallus gallus finished cDNA, clone ChEST567b14 / Finished cDNA, clone ChEST567b14 / --- / --- / --- / --- / --- / Gallus gallus finished cDNA, clone ChEST567b14 / Finished cDNA, clone ChEST567b14
RIGG05323	2.302	0.00207	CACTGCCAGCATAGTCTTGAGTGCATCACTTGGATGTTGTATCAGTGGACAGAATTTCAAGCCTCCACGTA	Genome Hit Contig19.397	Genome Hit Contig19.397 / --- / Gallus gallus finished cDNA, clone ChEST743l10 / Finished cDNA, clone ChEST743l10 / --- / --- / --- / --- / --- / Gallus gallus finished cDNA, clone ChEST743l10 / Finished cDNA, clone ChEST743l10

Gene Name	Fold Change	p-value	SEQUENCE	Array Description	Blast/Database Description
RIGG02976	2.297	0.0441	GCAAACAGCGTTCCTTTCTTTAATAA AATGTTACAAAACCCCAGTTCCATG CAGTTGCTTTACACTCAGG	Weakly similar to AAM21096 (AAM21096) Small GTP binding protein RAB13	Weakly similar to AAM21096 (AAM21096) Small GTP binding protein RAB13 / RF|NP_001026675.1|71895669|NM_001031504 mel transforming oncogene {Gallus gallus} (exp=-1; wgp=0; cg=0), partial (66%) / Gallus gallus mRNA for hypothetical protein, clone 2k8 / RAB8A, member RAS oncogene family (RAB8A), mRNA / RAB8A, RCJMB04_2k8: Ras-related protein Rab-8A / --- / --- / RAB8A, member RAS oncogene family (RAB8A), mRNA; / --- / --- / ---
RIGG01759	2.292	0.0228	CACTGAAGAGTAGTTCTGAATTGGA TTATTGCTGCCAAGTGAGTGGCTTT TGGTCCCCTCTAACCTTAGA	Weakly similar to MTS1_HUMAN (O43312) Metastasis suppressor protein 1 (Missin	Weakly similar to MTS1_HUMAN (O43312) Metastasis suppressor protein 1 (Missin / Gallus gallus finished cDNA, clone ChEST236p13 / 603235156F1 CSEQRBN09 Gallus gallus cDNA clone ChEST236p13 5', mRNA sequence / Finished cDNA, clone ChEST236p13 / RCJMB04_1a13: Hypothetical protein / --- / --- / Metastasis suppressor 1 (MTSS1), mRNA; / --- / 603235156F1 CSEQRBN09 Gallus gallus cDNA clone ChEST236p13 5', mRNA sequence / Finished cDNA, clone ChEST236p13
RIGG03821	2.284	0.00659	GTCCTGCTCTTAGGACAGTGCTGCT CTGTAATTATGTTCTGCTTACTAATT AAGCGTCATGTTCCTGTTATCCC	Genome Hit Contig43.35	Genome Hit Contig43.35 / Gallus gallus finished cDNA, clone ChEST1022j9 / Gallus gallus finished cDNA, clone ChEST548p12 / Finished cDNA, clone ChEST548p12 / --- / --- / --- / --- / --- / Gallus gallus finished cDNA, clone ChEST548p12 / Finished cDNA, clone ChEST548p12
RIGG11292	2.283	0.0307	CTGGCTTTGTTGTCAATATCTGGCTT TTGCGACTGTAGACTCGCTTCATCG CATCGTGAGAATCATGCAG		ENSGALT00000004357.1 / --- / --- / --- / hypothetical protein / --- / --- / --- / --- / --- / ---
RIGG06885	2.283	0.00567	TTGGAAATGTGTTTGAAAGTTGTGC TGTGTCTGAATTCAGCTGAAAGTGG TGAAAATACATTGCCTCACTGCC	Weakly similar to Q96FY9 (Q96FY9) Hypothetical protein	Weakly similar to Q96FY9 (Q96FY9) Hypothetical protein / Gallus gallus finished cDNA, clone ChEST959m4 / Gallus gallus finished cDNA, clone ChEST959m4 / Finished cDNA, clone ChEST959m4 / --- / --- / --- / --- / --- / Gallus gallus finished cDNA, clone ChEST959m4 / Finished cDNA, clone ChEST959m4
RIGG14570	2.275	0.0427	GTGTCACATCCCCATTGTTGTTCAC TGCTTCTGGATACTTATCCTTCAGTA TCATGCAAGTTGTTGACATCTT		ENSGALT00000013944.1 / similar to UP|Q80U92_MOUSE (Q80U92) MKIAA0027 protein (Fragment), partial (74%) / --- / --- / --- / PREDICTED: Gallus gallus similar to Megalencephalic leukoencephalopathy with subcortical cysts 1 gene product (LOC417737), mRNA. / PREDICTED: similar to Megalencephalic leukoencephalopathy with subcortical cysts 1 gene product; / --- / --- / --- / ---

Gene Name	Fold Change	p-value	SEQUENCE	Array Description	Blast/Database Description
RIGG07343	2.275	0.0216	TGTGGTTATACAGTCAATAGTATTGTTCTTTAGAGCAGCCTGTGTTAGAAGGATCCCAGGACACCTGTGTG		Contig_141_reverse / similar to GB|AAR21087.1|38479412|AY450394 DNA polymerase-transactivated protein 6 {Homo sapiens} (exp=-1; wgp=0; cg=0), partial (70%) / Gallus gallus mRNA for hypothetical protein, clone 33c18 / Similar to DNA polymerase-transactivated protein 6 (LOC424067), mRNA / --- / PREDICTED: Gallus gallus similar to DNA polymerase-transactivated protein 6 (LOC424067), mRNA.PREDICTED: Gallus gallus similar to DNA polymerase-transactivated protein 6 (LOC424068), mRNA. / PREDICTED: similar to DNA polymerase-transactivated protein 6; / Similar to DNA polymerase-transactivated protein 6 (LOC424067), mRNA; / --- / Gallus gallus mRNA for hypothetical protein, clone 33c18 / Similar to DNA polymerase-transactivated protein 6 (LOC424067), mRNA
RIGG02998	2.272	0.0458	CCAGCTTGAGGAAGACATCATGACTGTTACTCAGAAAGCCATTGCAAAAGAAACAGAGTTAGACAGTATGA	Same gene D89964; Gallus gallus MDP62 mRNA for muscle-derived protein 62, complete cds	Same gene D89964; Gallus gallus MDP62 mRNA for muscle-derived protein 62, complete cds / Gallus gallus finished cDNA, clone ChEST420p7 / Gallus gallus MDP62 mRNA for muscle-derived protein 62, complete cds / MDP62 mRNA for muscle-derived protein 62 / --- / PREDICTED: Gallus gallus muscle-derived protein 62 (MDP62), mRNA. / --- / MDP62 mRNA for muscle-derived protein 62; / --- / Gallus gallus MDP62 mRNA for muscle-derived protein 62, complete cds / MDP62 mRNA for muscle-derived protein 62
RIGG03678	2.268	0.0482	GATGATGGGCTTTGGTTCCCACATGCAACATCTTTGCCTGCCTGTATTCTCAGACTCCTAGCTATATCAGCCTT	Weakly similar to NDP_HUMAN (Q00604) Norrin (Norrie disease protein)	Weakly similar to NDP_HUMAN (Q00604) Norrin (Norrie disease protein) / Gallus gallus finished cDNA, clone ChEST533p17 / Gallus gallus finished cDNA, clone ChEST273h1 / Finished cDNA, clone ChEST740l1 / --- / PREDICTED: Gallus gallus similar to Norrie disease protein; norrin (LOC418560), mRNA. / PREDICTED: similar to Norrie disease protein; norrin; / Finished cDNA, clone ChEST740l1; / --- / Gallus gallus finished cDNA, clone ChEST273h1 / Finished cDNA, clone ChEST740l1
RIGG03425	2.267	0.0366	GTGCAGTCTCTGCGATATGATGTGGTCATGAAAGCTGGCTTAGACATTGCAAGAAATAAAGTAGAAGATGC	Weakly similar to EAA04857 (EAA04857) AgCP4268 (Fragment)	Weakly similar to EAA04857 (EAA04857) AgCP4268 (Fragment) / Gallus gallus finished cDNA, clone ChEST1017p10 / Gallus gallus finished cDNA, clone ChEST730h15 / Finished cDNA, clone ChEST742n24 / --- / PREDICTED: Gallus gallus similar to HSPC230 protein; HSPC230 gene (LOC421779), mRNA. / PREDICTED: similar to HSPC230 protein; HSPC230 gene; / --- / --- / --- / ---

Gene Name	Fold Change	p-value	SEQUENCE	Array Description	Blast/Database Description
RIGG04068	2.26	0.0184	TTCCACGCTCTTCTCTCCGGCGCGG TAAATCACTCTTCTTTAGTTACAGTC TTCTCCCGTCAGCA	Weakly similar to PBX1_MOUSE (P41778) Pre-B-cell leukemia transcription facto	Weakly similar to PBX1_MOUSE (P41778) Pre-B-cell leukemia transcription facto / RF|NP_001025849.1|71897289|NM_001030678 pre-B-cell leukemia transcription factor 3 {Gallus gallus} (exp=-1; wgp=0; cg=0), complete / Gallus gallus mRNA for hypothetical protein, clone 6b10 / Pre-B-cell leukemia transcription factor 3 (PBX3), mRNA / RCJMB04_6b10: Hypothetical protein / PREDICTED: Gallus gallus similar to pre-B-cell leukemia transcription factor 3 (LOC417093), mRNA. / PREDICTED: similar to pre-B-cell leukemia transcription factor 3; / Pre-B-cell leukemia transcription factor 3 (PBX3), mRNA; / --- / --- / ---
RIGG03797	2.258	0.0425	CATACAAGGTATGGACAAGGCATAC AAGTTACTACATGCTAGGCACTCCC TTTTCATCCCTTGAATCGTTTGT	Weakly similar to O75271 (O75271) R31237_1, partial CDS (Fragment)	Weakly similar to O75271 (O75271) R31237_1, partial CDS (Fragment) / Gallus gallus finished cDNA, clone ChEST546i7 / Gallus gallus finished cDNA, clone ChEST546i7 / Finished cDNA, clone ChEST546i7 / --- / --- / --- / --- / --- / Gallus gallus finished cDNA, clone ChEST546i7 / Finished cDNA, clone ChEST546i7
RIGG18665	2.254	0.0275	CTCCAGTTGGTAAAGCCATGGTATG ATGAAGTGAAGGATTATGCTTTTCCT TATCCTCAGGACTGCAACC	protease inhibitor 15 [Gallus gallus]. [Source:RefSeq;Acc:NM_204334]	protease inhibitor 15 [Gallus gallus]. [Source:RefSeq / UP|Q98ST6_CHICK (Q98ST6) SugarCrisp, partial (82%) / Gallus gallus peptidase inhibitor 15 (PI15), mRNA / SugarCrisp / SugarCrisp / --- / protease inhibitor 15; / --- / --- / Gallus gallus peptidase inhibitor 15 (PI15), mRNA / SugarCrisp
RIGG13407	2.253	0.0226	TTTACGATGTCATGATAAGATGCTG GAAGACCAAACCAGAGGACCGTCC CACCTTTGAGTACATGCAGAG	Proto-oncogene tyrosine-protein kinase LCK (EC 2.7.1.112) (Protein- tyrosine kinase C-TKL). [Source:SWISSPROT;Acc:P42683]	Proto-oncogene tyrosine-protein kinase LCK (EC 2.7.1.112) (Protein- tyrosine kinase C-TKL). [Source:SWISSPROT / similar to UP|HCK_MOUSE (P08103) Tyrosine-protein kinase HCK (p56-HCK/p59-HCK) (Hemopoietic cell kinase) (B-cell/myeloid kinase) (BMK) , partial (31%) / Gallus gallus mRNA for hypothetical protein, clone 2j8 / V-yes-1 Yamaguchi sarcoma viral related oncogene homolog (LYN), mRNA / / --- / --- / --- / hemopoietic cell kinase; / spleen_EST04459 Spleen cDNA Library Gallus gallus cDNA 3', mRNA sequence / Transcribed locus, moderately similar to XP_417455.1 PREDICTED: similar to Transmembrane 9 superfamily protein member 4 [Gallus gallus]
RIGG00137	2.249	0.044	GATGTGCAAGACCTGCTGAACAGAA CACTGACAATGGTGTCCAACGGCTT CCTCGACAAGCAGGC	Gallus gallus mRNA for hypothetical protein, clone 7i4	Gallus gallus mRNA for hypothetical protein, clone 7i4 / similar to GB|AAA66354.1|183186|HUMGIF intrinsic factor {Homo sapiens} (exp=-1; wgp=0; cg=0), partial (8%) / Gallus gallus mRNA for hypothetical protein, clone 7i4 / Hypothetical protein, clone 7i4 / RCJMB04_7i4: Hypothetical protein / --- / --- / --- / --- / Gallus gallus finished cDNA, clone ChEST535g22 / Finished cDNA, clone ChEST535g22
RIGG00343	2.247	0.048	TACTTCAATCTCATCTCCTCGGTGG ACCCCAAATTCCTGACGCTCACCAA AGTGGACGAGCAGATCTATG	Gallus gallus mRNA for hypothetical protein, clone 33c16	Gallus gallus mRNA for hypothetical protein, clone 33c16 / similar to GB|AAA18780.1|488466|MMU08339 GDF7 {Mus musculus} (exp=-1; wgp=0; cg=0), partial (10%) / AJ453064 riken1 Gallus gallus cDNA clone 33c16r1, mRNA sequence / Hypothetical protein, clone 33c16 / --- / --- / --- / --- / --- / AJ453064 riken1 Gallus gallus cDNA clone 33c16r1, mRNA sequence / Hypothetical protein, clone 33c16

Gene Name	Fold Change	p-value	SEQUENCE	Array Description	Blast/Database Description
RIGG17764	2.245	0.00266	TGGCAAATTCAGGGACGTAAAGTCTCCTCAGTTACTCATCTTTTCCGCATAGACCGCCTTGAAACGTTGTGTTT		ENSGALT00000022823.1 / Gallus gallus finished cDNA, clone ChEST923o19 / Gallus gallus finished cDNA, clone ChEST216n18 / Finished cDNA, clone ChEST923o19 / --- / --- / --- / Finished cDNA, clone ChEST923o19; / --- / Gallus gallus finished cDNA, clone ChEST216n18 / Finished cDNA, clone ChEST923o19
RIGG08920	2.243	0.0274	CCATCCACATCTTCGTCATCGTCTGGACGCTGGTGGACATCGACAAGAAGAACCCCTACGTGGTG		ENSGALG00000010966.1 / --- / 603749479F1 CSEQCHN04 Gallus gallus cDNA clone ChEST659n20 5', mRNA sequence / Transcribed locus, weakly similar to XP_320274.2 ENSANGP00000009053 [Anopheles gambiae str. PEST] / similar to delta opioid receptor / PREDICTED: Gallus gallus similar to delta opioid-like receptor (LOC429951), mRNA. / PREDICTED: similar to delta opioid-like receptor; / --- / --- / --- / ---
RIGG10053	2.241	0.0357	ACGGGAATGGCAACACAGCCCTGCACTACAGCGTCTCCCACTCCAACTTCCACATTGTGAAGCTG		ENSGALT00000000880.1 / --- / --- / --- / --- / --- / --- / --- / --- / --- / ---
RIGG02510	2.241	0.0229	TTTGTGCAGTGCTTTCATGGAAGGTATCGACTTCTGGAAGTTCTGATGGATGCCTGGATGTGCATTTAAA	Weakly similar to Q99M07 (Q99M07) Similar to CG13018 gene product	Weakly similar to Q99M07 (Q99M07) Similar to CG13018 gene product / Gallus gallus finished cDNA, clone ChEST357b16 / 603110083F1 CSEQCHL12 Gallus gallus cDNA clone ChEST55e22 5', mRNA sequence / Finished cDNA, clone ChEST871f14 / --- / --- / --- / --- / --- / 603110083F1 CSEQCHL12 Gallus gallus cDNA clone ChEST55e22 5', mRNA sequence / Finished cDNA, clone ChEST871f14
RIGG02651	2.239	0.0339	TGGTGTTTAGGCATTGGAATCATCTGCTGGCCATTGTAAGTGTGTTCAATAAATGCACTGGAGTTCTCTC	Weakly similar to RK27_TOBAC (P30155) 50S ribosomal protein L27, chloroplast	Weakly similar to RK27_TOBAC (P30155) 50S ribosomal protein L27, chloroplast / Gallus gallus finished cDNA, clone ChEST1020h15 / gPGC_EST07052 Embryonic gonadal PGC cDNA Library Gallus gallus cDNA 5', mRNA sequence / Finished cDNA, clone ChEST1020h15 / similar to mitochondrial ribosomal protein L27 isoform 1 / PREDICTED: Gallus gallus similar to mitochondrial ribosomal protein L27 (L27mt) (LOC422105), mRNA. / PREDICTED: similar to mitochondrial ribosomal protein L27 (L27mt); / --- / --- / gPGC_EST07052 Embryonic gonadal PGC cDNA Library Gallus gallus cDNA 5', mRNA sequence / Finished cDNA, clone ChEST1020h15
RIGG02865	2.238	0.0222	CTCAGAGACCTCCTCCGTTCTTTGTGGGTCACGCCGATATTTATTGGTCGTATTTGTATTTATTTAAGGGG	Similar to EAA35389 (EAA35389) Hypothetical protein	Similar to EAA35389 (EAA35389) Hypothetical protein / Gallus gallus finished cDNA, clone ChEST398p5 / Gallus gallus finished cDNA, clone ChEST398p5 / Finished cDNA, clone ChEST398p5 / --- / --- / --- / --- / --- / Gallus gallus finished cDNA, clone ChEST398p5 / Finished cDNA, clone ChEST398p5
RIGG05075	2.237	0.0255	GCAAAAGGAGTTGATACACTTGCTCATTCACCTTTCTGAAGCTCTGCATGAAGCACAACTAAAACTCATAC	Similar to Q96SK0 (Q96SK0) Hypothetical protein FLJ14805	Similar to Q96SK0 (Q96SK0) Hypothetical protein FLJ14805 / Gallus gallus finished cDNA, clone ChEST709a22 / Gallus gallus finished cDNA, clone ChEST709a22 / Finished cDNA, clone ChEST709a22 / / PREDICTED: Gallus gallus similar to 3110023E09Rik protein (LOC423103), mRNA. / PREDICTED: similar to 3110023E09Rik protein; / Finished cDNA, clone ChEST709a22; / --- / Gallus gallus finished cDNA, clone ChEST709a22 / Finished cDNA, clone ChEST709a22

Gene Name	Fold Change	p-value	SEQUENCE	Array Description	Blast/Database Description
RIGG07900	2.236	0.0289	ATAAGTTCTACAGAGAATGGGAAGATTGAGGCCTCTGTCAGTAAAGGCTTGTCCAAATTTTACGCACTTC		ENSGALG00000002710.1 / similar to UP|IPMK_HUMAN (Q8NFU5) Inositol polyphosphate multikinase (Inositol 1,3,4,6-tetrakisphosphate 5-kinase) , partial (90%) / 603140008F1 CSEQCHL15 Gallus gallus cDNA clone ChEST130o2 5', mRNA sequence / Transcribed locus, weakly similar to XP_507801.1 PREDICTED: similar to inositol polyphosphate multikinase [Pan troglodytes] / similar to inositol polyphosphate multikinase / --- / --- / --- / --- / 603140008F1 CSEQCHL15 Gallus gallus cDNA clone ChEST130o2 5', mRNA sequence / Transcribed locus, weakly similar to XP_507801.1 PREDICTED: similar to inositol polyphosphate multikinase [Pan troglodytes]
RIGG09680	2.234	0.0259	CGTTGGAGATTTCAACTACATCCGAAGAATCTTTCCCTCTGGCAATGTCACCAACATACTGGAACTAAGC	Teneurin-4 (Fragment). [Source:SPTREMBL;Acc:Q9DEQ8]	Teneurin-4 (Fragment). [Source:SPTREMBL / --- / Gallus gallus teneurin 2 (TEN2), transcript variant 1, mRNA / Teneurin-2, short splice variant (ten2 gene) / --- / PREDICTED: Gallus gallus similar to KIAA1302 protein (LOC429230), partial mRNA. / PREDICTED: similar to KIAA1302 protein, partial; / --- / --- / --- / ---
RIGG02886	2.227	0.0121	CGTCTGGGTGGGGTGTCCGTGTACTGTATGCACATTGCTATATTTAACAAACTATATGTTAGAACCACATT	Similar to Q8R1A9 (Q8R1A9) Hypothetical 49.1 kDa protein (Fragment)	Similar to Q8R1A9 (Q8R1A9) Hypothetical 49.1 kDa protein (Fragment) / Gallus gallus finished cDNA, clone ChEST3i10 / Gallus gallus mRNA for hypothetical protein, clone 35d18 / WD repeat domain, phosphoinositide interacting 2 (WIPI2), mRNA / --- / --- / --- / --- / WD repeat domain, phosphoinositide interacting 1; / 603490078F1 CSEQCHN63 Gallus gallus cDNA clone ChEST390l19 5', mRNA sequence / Finished cDNA, clone ChEST226o18
RIGG07999	2.225	0.0421	CGGTTTGCTTATTCACGCTGTCATAGTCCTACCTCTGCTGTACTTCCTAATCACTCGTAAGAACCCTTGG	Na+-dependent glutamate/aspartate transporter (Fragment). [Source:SPTREMBL;Acc:Q9IAS3]	Na+-dependent glutamate/aspartate transporter (Fragment). [Source:SPTREMBL / homologue to GB|AAB26422.1|299942|S59158 glutamate transporter {Rattus sp.} (exp=-1; wgp=0; cg=0), partial (55%) / 603135172F1 CSEQCHL25 Gallus gallus cDNA clone ChEST118o17 5', mRNA sequence / Na+-dependent glutamate/aspartate transporter / Na+-dependent glutamate/aspartate transporter (Fragment) / --- / --- / --- / --- / 603135172F1 CSEQCHL25 Gallus gallus cDNA clone ChEST118o17 5', mRNA sequence / Na+-dependent glutamate/aspartate transporter
RIGG18106	2.224	0.0214	GAAGGATGGTGACTCCCTTGACTTGCATAACAGCAACAATTCTCATCATGAGCGAGATATAAACCGAAACTTTGA		ENSGALT00000023747.1 / similar to UP|Q6NSH6_HUMAN (Q6NSH6) GPBP1 protein (Fragment), partial (84%) / Gallus gallus finished cDNA, clone ChEST154m14 / Finished cDNA, clone ChEST154m14 / --- / --- / --- / --- / GC-rich promoter binding protein 1; / Gallus gallus finished cDNA, clone ChEST154m14 / Finished cDNA, clone ChEST154m14
RIGG08641	2.224	0.0382	CAACAACGAGGGCTGGAACCTGTGTGACATCTGACTCAGAAGTTGTTCGTGACATATTTTATGATGGAAA		ENSGALG00000008694.1 / --- / --- / --- / --- / PREDICTED: Gallus gallus similar to selenoprotein O (LOC417745), mRNA. / PREDICTED: similar to selenoprotein O; / --- / --- / --- / ---

Gene Name	Fold Change	p-value	SEQUENCE	Array Description	Blast/Database Description
RIGG06210	2.222	0.0262	TTGTGCTTTCTGCTAAAGACGACGT GTTATTACCTGTCAGCGGGTCTGAT AATACATAGAACCTGGTGCA	Genome Hit Contig68.60	Genome Hit Contig68.60 / Gallus gallus finished cDNA, clone ChEST433c17 / --- / --- / --- / --- / --- / --- / --- / --- / ---
RIGG00210	2.221	0.0357	TTTAAAGCTGAGAATGCCTCTGCCT TGTCTGTGGAAACTGTCTTGATCCC TCTGTTTGGATGTCCCCAGG	Gallus gallus mRNA for hypothetical protein, clone 14g3	Gallus gallus mRNA for hypothetical protein, clone 14g3 / similar to UP|Q802D6_MELUD (Q802D6) Thioredoxin 2, partial (82%) / gPGC_EST02752 Embryonic gonadal PGC cDNA Library Gallus gallus cDNA 5', mRNA sequence / Similar to thioredoxin 2 (LOC426978), mRNA / RCJMB04_14g3: Hypothetical protein / --- / --- / --- / --- / gPGC_EST02752 Embryonic gonadal PGC cDNA Library Gallus gallus cDNA 5', mRNA sequence / Similar to thioredoxin 2 (LOC426978), mRNA
RIGG14847	2.221	0.0396	TGGCAAGTCTCACCTGAAAAGAGTG AAGCAGCTAAATAATGGAAAGCTCC CTACAAGTACAGCTCCTGGC		ENSGALT00000014659.1 / Gallus gallus finished cDNA, clone ChEST470h3 / WLA108D03.ab1 WLbrain Gallus gallus cDNA 5', mRNA sequence / Finished cDNA, clone ChEST470h3 / similar to Zinc finger protein 533 / --- / --- / --- / --- / WLA108D03.ab1 WLbrain Gallus gallus cDNA 5', mRNA sequence / Finished cDNA, clone ChEST470h3
RIGG03050	2.22	0.0189	CTTTTCTTGCTTCCCTTTGGTTTTGT CACTGTTGGTGCTAGAAATAGATGT CCACATAGTGTTTCGCTGT	Genome Hit Contig18.193	Genome Hit Contig18.193 / Gallus gallus finished cDNA, clone ChEST42m2 / 603104807F1 CSEQCHN04 Gallus gallus cDNA clone ChEST42m2 5', mRNA sequence / Finished cDNA, clone ChEST42m2 / --- / PREDICTED: Gallus gallus hypothetical gene supported by CR387621 (LOC419237), mRNA. / PREDICTED: hypothetical protein XP_429640; / Finished cDNA, clone ChEST42m2; / --- / 603104807F1 CSEQCHN04 Gallus gallus cDNA clone ChEST42m2 5', mRNA sequence / Finished cDNA, clone ChEST42m2
RIGG18727	2.22	0.04	GACGGAGGTTGTCCAGTCTTGCCGC AATTTCATTAAAGATGCCTTAAACAT AAGTATAAAATCAGAAGCTCCG		ENSGALT00000025397.1 / Gallus gallus finished cDNA, clone ChEST610j14 / gPGC_EST02672 Embryonic gonadal PGC cDNA Library Gallus gallus cDNA 5', mRNA sequence / Finished cDNA, clone ChEST610j14 / similar to zinc finger and BTB domain containing 10; zinc finger protein RINZF / --- / --- / --- / zinc finger and BTB domain containing 10; / --- / ---
RIGG06392	2.218	0.042	AGGCAGCTAATCTGTGTTTCCCCTT CAAACTATGTCATAAATCAACAGCA GATCCTCCTCTTCTTGGGAAC	Genome Hit Contig42.320	Genome Hit Contig42.320 / Gallus gallus finished cDNA, clone ChEST745f19 / Gallus gallus finished cDNA, clone ChEST745f19 / Finished cDNA, clone ChEST887d2 / --- / --- / --- / --- / --- / Gallus gallus finished cDNA, clone ChEST745f19 / Finished cDNA, clone ChEST887d2
RIGG03469	2.211	0.0422	AAATCAGGTTGAGAAGGGCTGTAAT GGCTTAGAACTGACTGTAAAACATC AACCGTTCTGATTTTCTGCT	Contig Hit 339567.3	Contig Hit 339567.3 / Gallus gallus finished cDNA, clone ChEST49n15 / --- / --- / --- / --- / --- / --- / --- / --- / ---

Gene Name	Fold Change	p-value	SEQUENCE	Array Description	Blast/Database Description
RIGG06191	2.21	0.0178	GGTGCTGCTCTTACCTCATCCTTTC TTCATTTTGCCATGAAATTTACTTTT GTCTTTGGCTTGAAGTGGA	Similar to Q8VHI8 (Q8VHI8) BNIP1 protein	Similar to Q8VHI8 (Q8VHI8) BNIP1 protein / Gallus gallus finished cDNA, clone ChEST85o20 / Gallus gallus finished cDNA, clone ChEST85o20 / Finished cDNA, clone ChEST85o20 / --- / PREDICTED: Gallus gallus similar to BCL2/adenovirus E1B 19kD interacting protein 1, isoform BNIP1 (LOC416207), mRNA. / PREDICTED: similar to BCL2/adenovirus E1B 19kD interacting protein 1, isoform BNIP1; / Finished cDNA, clone ChEST85o20; / --- / Gallus gallus finished cDNA, clone ChEST85o20 / Finished cDNA, clone ChEST85o20
RIGG19145	2.206	0.0396	AGGTCTTTCCAGAAGATTCCTCTGT AGTTGAAGTTGATTGTTCCCAAAGTT CAGAACAGCTGTTTCAGGG		ENSGALT00000026572.1 / Gallus gallus finished cDNA, clone ChEST280j17 / --- / --- / hypothetical protein / --- / --- / --- / --- / --- / ---
RIGG01100	2.205	0.0241	CCCTAACCTACCATTTCCAGGACCT ACATTCTCACAGTCTAACCTAAGCTA TTAACACGTATGGCAGATC	Weakly similar to Q802H1 (Q802H1) Chemokine receptor CXCR4	Weakly similar to Q802H1 (Q802H1) Chemokine receptor CXCR4 / Gallus gallus finished cDNA, clone ChEST149a23 / Gallus gallus finished cDNA, clone ChEST149a23 / Finished cDNA, clone ChEST149a23 / / --- / --- / Finished cDNA, clone ChEST149a23; / G protein-coupled receptor 146; / Gallus gallus finished cDNA, clone ChEST149a23 / Finished cDNA, clone ChEST149a23
RIGG11649	2.205	0.012	GCGTGCAGCTCGTGGTCAACTATGA CTTCCCAAACACCCTGCAGGATTAC CTACACCGTGTGGGG		ENSGALT00000005401.1 / similar to UP|DDX28_HUMAN (Q9NUL7) Probable ATP-dependent RNA helicase DDX28 (Mitochondrial DEAD box protein 28) , partial (47%) / --- / --- / --- / PREDICTED: Gallus gallus similar to DEAD (Asp-Glu-Ala-Asp) box polypeptide 28; DEAD/H (Asp-Glu-Ala-Asp/His) box polypeptide 28 (LOC427544), mRNA. / PREDICTED: similar to DEAD (Asp-Glu-Ala-Asp) box polypeptide 28; DEAD/H (Asp-Glu-Ala-Asp/His) box polypeptide 28; / --- / --- / BX278843 AGENAE Gallus gallus multi-tissues normalized library (gcag) Gallus gallus cDNA clone gcag0011.j.12 5prim, mRNA sequence / Transcribed locus, strongly similar to XP_425116.1 PREDICTED: similar to DEAD (Asp-Glu-Ala-Asp) box polypeptide 28; DEAD/H (Asp-Glu-Ala-Asp/His) box polypeptide 28 [Gallus gallus]
RIGG01064	2.204	0.0306	GACTTCATGCAGCCCCTTCAGAGTA TACTCCATCTGGCCATAGGAGACAA ACTTTGTGGGTTTTAACAAA	Weakly similar to Q8I3B7 (Q8I3B7) Hypothetical protein	Weakly similar to Q8I3B7 (Q8I3B7) Hypothetical protein / homologue to UP|RER1_MOUSE (Q9CQU3) RER1 protein, complete / Gallus gallus mRNA for hypothetical protein, clone 35f13 / Similar to RER1 homolog (LOC419397), mRNA / RER1, RCJMB04_35f13: Protein RER1 / --- / PREDICTED: similar to RER1 homolog; / --- / --- / Gallus gallus mRNA for hypothetical protein, clone 35f13 / Similar to RER1 homolog (LOC419397), mRNA

Gene Name	Fold Change	p-value	SEQUENCE	Array Description	Blast/Database Description
RIGG19526	2.196	0.026	GTAAGAGTGCCGTCTTTATTCAGCATTATGCCTATTCCTGTTCTCCGATCCATGCTGTCAAAAGTGGTCC		ENSGALT00000027606.1 / weakly similar to UP|Q96QA1_HUMAN (Q96QA1) FKSG16, partial (52%) / Gallus gallus mRNA for hypothetical protein, clone 1c21 / Similar to hypothetical protein LOC283537 (LOC418924), mRNA / --- / PREDICTED: Gallus gallus similar to hypothetical protein LOC283537 (LOC418924), mRNA. / PREDICTED: hypothetical protein XP_417118; / Similar to hypothetical protein LOC283537 (LOC418924), mRNA; / --- / Gallus gallus mRNA for hypothetical protein, clone 1c21 / Similar to hypothetical protein LOC283537 (LOC418924), mRNA
RIGG16825	2.193	0.0316	AGTATTTCCCTACGGAACCCCTCAACATCATCACAGTCAATACTGCTTTCGCATGGCTCAGTTGTGCTGG		ENSGALT00000020218.1 / --- / --- / --- / / --- / --- / --- / --- / --- / ---
RIGG04784	2.188	0.0312	GCTGTTTACATGCTCCTGAAATCTGTTTGGAGTTCTTGGTATGACAATAACTGGGTCAGATGATTGTGCT	Genome Hit Contig3633.1	Genome Hit Contig3633.1 / --- / Gallus gallus finished cDNA, clone ChEST667h5 / Finished cDNA, clone ChEST667h5 / --- / --- / --- / --- / --- / Gallus gallus finished cDNA, clone ChEST667h5 / Finished cDNA, clone ChEST667h5
RIGG01979	2.185	0.000836	CCCACTCCTATTCTTCCACTTCCTACTCTCATGGACAATCTGGTGGAGACAAGTCAGGGCAGACAAGAGT	Weakly similar to K1CO_MOUSE (Q61414) Keratin, type I cytoskeletal 15 (Cytoke	Weakly similar to K1CO_MOUSE (Q61414) Keratin, type I cytoskeletal 15 (Cytoke / Gallus gallus finished cDNA, clone ChEST268i20 / Gallus gallus finished cDNA, clone ChEST268i20 / Finished cDNA, clone ChEST268i20 / similar to keratin 10; cytokeratin 10 / --- / --- / --- / --- / Gallus gallus finished cDNA, clone ChEST268i20 / Finished cDNA, clone ChEST268i20
RIGG08497	2.183	0.0315	ATGCCCTGGGTCCCTTTGTGGAGGAGCTGTTTGAAGTGACATCCTGCTACTTCCCTATTGATTTTACTCC		ENSGALG00000007572.1 / similar to UP|MMS19_MOUSE (Q9D071) MMS19-like protein (MET18 homolog), partial (29%) / --- / --- / --- / --- / --- / --- / MMS19-like (MET18 homolog, S. cerevisiae); / --- / ---
RIGG02397	2.181	0.0295	GTTCTTCCTCTCAGCCAGAATCAGGAATTCATGCCTTTTGTCAAAGAATTATCTGAAGCACTCACCAGGA	Same gene AC092403; Gallus gallus clone WAG-100N11, complete sequence	Same gene AC092403; Gallus gallus clone WAG-100N11, complete sequence / RF|NP_001026681.1|71895709|NM_001031510 glycyl-tRNA synthetase {Gallus gallus} (exp=-1; wgp=0; cg=0), complete / Gallus gallus mRNA for hypothetical protein, clone 34b10 / Glycyl-tRNA synthetase (GARS), mRNA / RCJMB04_34b10: Hypothetical protein / --- / --- / Glycyl-tRNA synthetase (GARS), mRNA; / --- / Gallus gallus mRNA for hypothetical protein, clone 34b10 / Glycyl-tRNA synthetase (GARS), mRNA

Gene Name	Fold Change	p-value	SEQUENCE	Array Description	Blast/Database Description
RIGG15034	2.177	0.036	TCTCAGATCCTGAGTTGTAATAAGTGCAACACGCCTTTTAAAGATAATGACACTAAACATCACTGCCGGG		ENSGALT00000015206.1 / homologue to UP|ZFYV1_HUMAN (Q9HBF4) Zinc finger FYVE domain-containing protein 1 (Double FYVE-containing protein 1) (Tandem FYVE fingers-1) (SR3), partial (60%) / --- / --- / / PREDICTED: Gallus gallus similar to zinc finger, FYVE domain containing 1 isoform 1; tandem FYVE fingers-1 protein; zinc finger protein, subfamily 2A, member 1; double FYVE-containing protein 1; phosphoinositide-binding protein SR3; zinc finger protein, s / PREDICTED: similar to zinc finger, FYVE domain containing 1 isoform 1; tandem FYVE fingers-1 protein; zinc finger protein, subfamily 2A, member 1; double FYVE-containing protein 1; phosphoinositide-binding protein SR3; zinc finger protein, subfamily 2A (F; / --- / --- / --- / ---
RIGG16920	2.177	0.00944	CTGGGAATTCAGAAGAGAAACTGGTGTCCAAAGCCACCAAGATGATGTCGGATCCCATGAGCCAG	G protein-activated inward rectifier potassium channel 1 (GIRK1) (Potassium channel	G protein-activated inward rectifier potassium channel 1 (GIRK1) (Potassium channel / UP|IRK3_CHICK (Q90854) G protein-activated inward rectifier potassium channel 1 (GIRK1) (Potassium channel, inwardly rectifying subfamily J member 3) (Inward rectifier K(+) channel Kir3.1), complete / Gallus gallus potassium inwardly-rectifying channel, subfamily J, member 3 (KCNJ3), mRNA / GIRK1 / KCNJ3, GIRK1: G protein-activated inward rectifier potassium channel 1 / Gallus gallus GIRK1 protein (GIRK1), mRNA. / GIRK1 protein; / GIRK1; / --- / Gallus gallus potassium inwardly-rectifying channel, subfamily J, member 3 (KCNJ3), mRNA / GIRK1
RIGG05636	2.176	0.0181	ATTCAATGCATCGACTCCTACAGACCATACAGTTCCCCAAAGATGCTCTGAAATACCTGATTCAAGATGT	Similar to AAH54494 (AAH54494) Nischarin	Similar to AAH54494 (AAH54494) Nischarin / Gallus gallus finished cDNA, clone ChEST783j18 / 604130394F1 CSEQRBN37 Gallus gallus cDNA clone ChEST956k23 5', mRNA sequence / Finished cDNA, clone ChEST783j18 / similar to KIAA0975 protein / --- / --- / --- / nischarin; / 604130394F1 CSEQRBN37 Gallus gallus cDNA clone ChEST956k23 5', mRNA sequence / Finished cDNA, clone ChEST783j18
RIGG00383	2.175	0.00817	ACCAGCCCATCTCTTTGGTCTTTTATTAGATATCAGAGAAGACACATAGAAGAGACCAGCCTTTGCAATTG	Gallus gallus mRNA for hypothetical protein, clone 3j12	Gallus gallus mRNA for hypothetical protein, clone 3j12 / RF|NP_001025866.1|71897351|NM_001030695 zinc finger protein 184 (Kruppel-like) {Gallus gallus} (exp=-1; wgp=0; cg=0), complete / Gallus gallus mRNA for hypothetical protein, clone 3j12 / Zinc finger protein 23 (KOX 16) (ZNF23), mRNA / RCJMB04_3j12: Hypothetical protein / --- / --- / --- / --- / Gallus gallus mRNA for hypothetical protein, clone 3j12 / Zinc finger protein 23 (KOX 16) (ZNF23), mRNA
RIGG14293	2.174	0.00886	CCCAGCTCCTTTGTGCTCAACGGCTTCATGTACTCACTCATTGGGCTCTACGACCTGAAGGAGAC		ENSGALT00000013097.1 / --- / --- / --- / similar to D-glucuronyl C5 epimerase / --- / --- / --- / UDP-glucuronic acid epimerase; / --- / ---
RIGG04763	2.171	0.0111	GCTGTCTCGAATCACCTCATTGTCTCGTATGTGCCTTAACATAGCTTCTAGGAGGATCTGCCCCATGATT	Similar to EAA45768 (EAA45768) ENSANGP00000024954 (Fragment)	Similar to EAA45768 (EAA45768) ENSANGP00000024954 (Fragment) / Gallus gallus finished cDNA, clone ChEST665n17 / Gallus gallus mRNA for hypothetical protein, clone 6c16 / Nedd4 family interacting protein 2 (NDFIP2), mRNA / Hypothetical protein / --- / --- / --- / --- / --- / ---

Gene Name	Fold Change	p-value	SEQUENCE	Array Description	Blast/Database Description
RIGG01922	2.166	0.0141	GGGTGGTTTTGCTCCAGTAGGAATTACTCTGACAATTGTTTGGATAAACAGAGTAAAGTTACATTGCAGCCT	Genome Hit Contig190.60	Genome Hit Contig190.60 / Gallus gallus finished cDNA, clone ChEST260o23 / 603364982F1 CSEQRBN21 Gallus gallus cDNA clone ChEST262d22 5', mRNA sequence / Finished cDNA, clone ChEST260o23 / --- / --- / --- / --- / --- / 603364982F1 CSEQRBN21 Gallus gallus cDNA clone ChEST262d22 5', mRNA sequence / Finished cDNA, clone ChEST260o23
RIGG16888	2.163	0.0302	CTGGAAGAAGCAGAGTTCTATAACATCGGCCCTTTAATCCGGATAATTAAGGATCGTCTAGAGGAGAAGGAC		ENSGALT00000020399.1 / homologue to UP|Q8N5Z5_HUMAN (Q8N5Z5) KCTD17 protein, partial (60%) / Gallus gallus mRNA for hypothetical protein, clone 20i10 / Potassium channel tetramerisation domain containing 2 (KCTD2), mRNA / similar to BTB/POZ domain-containing protein KCTD17 / PREDICTED: Gallus gallus similar to hypothetical protein FLJ12242 (LOC418047), mRNA. / PREDICTED: similar to hypothetical protein FLJ12242; / --- / --- / --- / ---
RIGG02057	2.162	0.0057	CAGAACAAGATGAAGGAGAATATCTGTGTAGAGCCAACAATTTCGTAGGCATAGCCGAAAAGCCATTTTG	Weakly similar to Q9WUR7 (Q9WUR7) Fibroblast growth factor receptor-3 (Fragme	Weakly similar to Q9WUR7 (Q9WUR7) Fibroblast growth factor receptor-3 (Fragme / Gallus gallus finished cDNA, clone ChEST278c3 / --- / --- / --- / --- / --- / --- / --- / --- / ---
RIGG19530	2.161	0.00846	CAGTCTGTTGTCATGGTGCTTTCAAAACAAACCTTGAAGACAAAATGATCCTGTCTCTGTCCACTGCTGTG		ENSGALT00000027615.1 / Gallus gallus finished cDNA, clone ChEST28i14 / Gallus gallus finished cDNA, clone ChEST28i14 / Finished cDNA, clone ChEST28i14 / --- / PREDICTED: Gallus gallus similar to hypothetical protein MGC9850 (LOC418927), mRNA. / PREDICTED: similar to hypothetical protein MGC9850; / Finished cDNA, clone ChEST28i14; / --- / Gallus gallus finished cDNA, clone ChEST28i14 / Finished cDNA, clone ChEST28i14
RIGG10621	2.154	0.035	GATAGCTGTGTCCCTCCTCTACTGCTTCCCACTTGTCATGTACTTTGATGTCACCTACGAGATGCCAGCG		ENSGALT00000002417.1 / Gallus gallus finished cDNA, clone ChEST604a20 / Gallus gallus finished cDNA, clone ChEST604a20 / Finished cDNA, clone ChEST604a20 / similar to alkaline ceramidase / --- / --- / --- / N-acylsphingosine amidohydrolase (alkaline ceramidase) 3; / Gallus gallus finished cDNA, clone ChEST604a20 / Finished cDNA, clone ChEST604a20
RIGG08097	2.152	0.0218	CCCTGATGTTATTGCCTTCAGAAAAGCTAATAAGGTAGGAGTTTTCATCAAGGTTACCCCACAGAAAGAGGAG		ENSGALG00000004534.1 / homologue to UP|DCTN4_HUMAN (Q9UJW0) Dynactin subunit 4 (Dynactin subunit p62), partial (86%) / 603866264F1 CSEQCHN54 Gallus gallus cDNA clone ChEST885p16 5', mRNA sequence / Transcribed locus, weakly similar to NP_610311.1 CG12042-PA [Drosophila melanogaster] / --- / PREDICTED: Gallus gallus similar to dynactin 4 (p62); dynactin p62 subunit (LOC416269), mRNA. / PREDICTED: similar to dynactin 4 (p62); dynactin p62 subunit; / --- / --- / --- / ---

Gene Name	Fold Change	p-value	SEQUENCE	Array Description	Blast/Database Description
RIGG13864	2.151	0.0404	GCATTGCTATAAGATGTTCAGAACT CCTGTATATCTACGGCTGTCTTGAT CCCCGTGTGAGAGCTCTGGT		ENSGALT00000011888.1 / similar to UP|Q6P7E5_HUMAN (Q6P7E5) PAP associated domain containing 1, partial (34%) / Gallus gallus finished cDNA, clone ChEST720b7 / Finished cDNA, clone ChEST720b7 / --- / PREDICTED: Gallus gallus similar to PAP associated domain containing 1 (LOC420478), mRNA. / PREDICTED: similar to PAP associated domain containing 1; / Finished cDNA, clone ChEST720b7; / --- / Gallus gallus finished cDNA, clone ChEST720b7 / Finished cDNA, clone ChEST720b7
RIGG11959	2.15	0.00208	CAGCTCACTAATGCTTCTGGAAGTT TTGATCTCCTTGACAACACTGACAT CCATCTGCACTTCCCAGCTG		ENSGALT00000006284.1 / Gallus gallus finished cDNA, clone ChEST35d5 / --- / --- / / --- / --- / --- / lon peptidase 2, peroxisomal; / gPGC_EST00743 Embryonic gonadal PGC cDNA Library Gallus gallus cDNA 5', mRNA sequence / Finished cDNA, clone ChEST35d5
RIGG00520	2.149	0.0361	GTTGCAGTTGCTCTACTCAGATGAC AGTGAAGTCTGAATAACAAACATTTC TTAACCTGCTATCAGCTGGC	Partial Contig Hit 509897.1	Partial Contig Hit 509897.1 / Gallus gallus finished cDNA, clone ChEST1004e4 / Gallus gallus finished cDNA, clone ChEST1004e4 / Finished cDNA, clone ChEST1004e4 / --- / --- / --- / --- / --- / Gallus gallus finished cDNA, clone ChEST1004e4 / Finished cDNA, clone ChEST1004e4
RIGG06897	2.148	0.0219	TATGCACTTACCTTCTCAAAGGCAG TACCAACAAAGTCAGTGAATTGGAT ATGTAGACTGCTCCCATACAAG	Genome Hit Contig83.120	Genome Hit Contig83.120 / RF|NP_001007976.1|56118964|NM_001007975 N-acetylneuraminic acid synthase (sialic acid synthase) {Gallus gallus} (exp=-1; wgp=0; cg=0), complete / Gallus gallus mRNA for hypothetical protein, clone 2a5 / N-acetylneuraminic acid synthase (sialic acid synthase) (NANS), mRNA / --- / --- / --- / N-acetylneuraminic acid synthase (sialic acid synthase) (NANS), mRNA; / --- / Gallus gallus mRNA for hypothetical protein, clone 2a5 / N-acetylneuraminic acid synthase (sialic acid synthase) (NANS), mRNA
RIGG00049	2.148	0.0198	AGTCCAAGGCTCTAGGAGAAAGCTG ATGGTTATTGAGAGTGATGCTTCTT GTATATTGTCAATGTGATTCCT	Gallus gallus mRNA for hypothetical protein, clone 2d1	Gallus gallus mRNA for hypothetical protein, clone 2d1 / RF|NP_001012806.1|61098278|NM_001012788 epsin 2 {Gallus gallus} (exp=-1; wgp=0; cg=0), complete / Gallus gallus mRNA for hypothetical protein, clone 2d1 / Epsin 2 (EPN2), mRNA / --- / --- / PREDICTED: similar to epsin 2 isoform a; Eps15 binding protein; / Epsin 2 (EPN2), mRNA; / --- / Gallus gallus mRNA for hypothetical protein, clone 2d1 / Epsin 2 (EPN2), mRNA
RIGG02833	2.147	0.0444	GCCCCAGCTAGGATGCAGTGTAGAA AGCCTCACTTCTCAAGCTGGACAAT TGTAGCTAAGGGCTAATTGT	Weakly similar to Q8C2Q7 (Q8C2Q7) Heterogeneous nuclear ribonucleoprotein H1	Weakly similar to Q8C2Q7 (Q8C2Q7) Heterogeneous nuclear ribonucleoprotein H1 / Gallus gallus finished cDNA, clone ChEST395k18 / 603491870F1 CSEQCHN63 Gallus gallus cDNA clone ChEST395k18 5', mRNA sequence / Finished cDNA, clone ChEST395k18 / --- / --- / --- / --- / --- / 603491870F1 CSEQCHN63 Gallus gallus cDNA clone ChEST395k18 5', mRNA sequence / Finished cDNA, clone ChEST395k18

Gene Name	Fold Change	p-value	SEQUENCE	Array Description	Blast/Database Description
RIGG03538	2.141	0.0286	CCTGCTCTCTCCAAGCTCACACTGATGTATGCGTTTATTCTGGAGTTCTCTCAGGAAAGACCATAATTACT	Genome Hit Contig61.77	Genome Hit Contig61.77 / --- / Gallus gallus finished cDNA, clone ChEST50p8 / Finished cDNA, clone ChEST50p8 / --- / --- / --- / --- / --- / Gallus gallus finished cDNA, clone ChEST50p8 / Finished cDNA, clone ChEST50p8
RIGG03627	2.14	0.0488	CGGTCAAGTCCCAGTATTACTTTCCTTTCAACCAAGTGCCATTCGGACATGCTTCACCTTTACCCGTGAA	Similar to Q8C1A9 (Q8C1A9) Hypothetical alpha/beta-Hydrolases/putative	Similar to Q8C1A9 (Q8C1A9) Hypothetical alpha/beta-Hydrolases/putative / homologue to UP|Q5CZU3_BRARE (Q5CZU3) Zgc:110741, partial (28%) / Gallus gallus mRNA for hypothetical protein, clone 2k21 / Similar to CG32112-PB (LOC422499), mRNA / --- / --- / --- / Similar to CG32112-PB (LOC422499), mRNA; / --- / Gallus gallus mRNA for hypothetical protein, clone 2k21 / Similar to CG32112-PB (LOC422499), mRNA
RIGG03113	2.14	0.0216	TCTATTCCATCTGTTCCAACTACGCTGAACAACAAAGTAACCAAAAGCACAGGTTTTACTCCCAGACCTGG	Contig Hit 037550.1	Contig Hit 037550.1 / Gallus gallus finished cDNA, clone ChEST437h7 / 603508828F1 CSEQCHN52 Gallus gallus cDNA clone ChEST437h7 5', mRNA sequence / Finished cDNA, clone ChEST437h7 / --- / --- / --- / --- / --- / 603508828F1 CSEQCHN52 Gallus gallus cDNA clone ChEST437h7 5', mRNA sequence / Finished cDNA, clone ChEST437h7
RIGG02565	2.139	0.0416	TTCTCAAACAAGTTGTGTGAAGGCAACACTTATCTCCTCTTACTGAAACTAAAAGACATGCATTGGCAGC	Weakly similar to CAE33220 (CAE33220) Putative exported protein	Weakly similar to CAE33220 (CAE33220) Putative exported protein / Gallus gallus finished cDNA, clone ChEST362d23 / --- / --- / --- / --- / --- / --- / sulfatase modifying factor 1; / --- / ---
RIGG04395	2.139	0.0106	TAAAGAATGCCTGCATCTCTTGTCCTGCGTGCAATCATGCTATAAACAAAATTACCATGTCTGGCTCCAG	Genome Hit Contig149.48	Genome Hit Contig149.48 / Gallus gallus finished cDNA, clone ChEST624f16 / Gallus gallus finished cDNA, clone ChEST624f16 / Finished cDNA, clone ChEST624f16 / --- / --- / --- / --- / --- / Gallus gallus finished cDNA, clone ChEST624f16 / Finished cDNA, clone ChEST624f16
RIGG01445	2.137	0.00842	CCTAATTTTAACTCGGGCGTATTTCTAACAATCGGTGGAAACTATGCACCTCTAACGGCCTTCTGAAAGC	Weakly similar to M4K6_MOUSE (Q9JM52) Mitogen-activated protein kinase kinase	Weakly similar to M4K6_MOUSE (Q9JM52) Mitogen-activated protein kinase kinase / Gallus gallus finished cDNA, clone ChEST200b23 / 603215289F1 CSEQRBN14 Gallus gallus cDNA clone ChEST200b23 5', mRNA sequence / Finished cDNA, clone ChEST200b23 / --- / --- / --- / --- / --- / 603215289F1 CSEQRBN14 Gallus gallus cDNA clone ChEST200b23 5', mRNA sequence / Finished cDNA, clone ChEST200b23
RIGG00659	2.136	0.0381	AACGGCTACATCCAGAAGATCAAATCAGGAGAAGAGGACTTTGAATCTCTCGCTTCGCAGTTCAGTGACT	Similar to Q9I9K6 (Q9I9K6) Prolyl isomerase Pin1	Similar to Q9I9K6 (Q9I9K6) Prolyl isomerase Pin1 / homologue to UP|Q5BIN5_BOVIN (Q5BIN5) Protein (Peptidyl-prolyl cis/trans isomerase) NIMA-interacting 1, partial (98%) / gPGC_EST08007 Embryonic gonadal PGC cDNA Library Gallus gallus cDNA 5', mRNA sequence / Finished cDNA, clone ChEST902k14 / --- / --- / --- / --- / --- / gPGC_EST08007 Embryonic gonadal PGC cDNA Library Gallus gallus cDNA 5', mRNA sequence / Finished cDNA, clone ChEST902k14

Gene Name	Fold Change	p-value	SEQUENCE	Array Description	Blast/Database Description
RIGG04511	2.135	0.028	AGTGGCAGTGATTCACATTCATTCC TCATAAGCCATTGAAATCAGTTCTTC ATCCGACCCATGATTGTAATCC	Weakly similar to RIK2_MOUSE (P58801) Receptor-interacting serine/threonine p	Weakly similar to RIK2_MOUSE (P58801) Receptor-interacting serine/threonine p / --- / Gallus gallus finished cDNA, clone ChEST637h3 / Finished cDNA, clone ChEST637h3 / similar to receptor-interacting protein 2 / --- / --- / Finished cDNA, clone ChEST637h3; / --- / Gallus gallus finished cDNA, clone ChEST637h3 / Finished cDNA, clone ChEST637h3
RIGG02339	2.135	0.0109	TACTTGGGTTGCTCTAGAATCTGTC ACTTAAGCATGTGCACTCTAACCATT GCCATGACTAGTTGGTGGGA	Contig Hit 042388.1	Contig Hit 042388.1 / Gallus gallus finished cDNA, clone ChEST329k3 / 603411314F1 CSEQCHN24 Gallus gallus cDNA clone ChEST329k3 5', mRNA sequence / Finished cDNA, clone ChEST329k3 / --- / --- / --- / --- / --- / 603411314F1 CSEQCHN24 Gallus gallus cDNA clone ChEST329k3 5', mRNA sequence / Finished cDNA, clone ChEST329k3
RIGG17643	2.131	0.0247	AGGCAAGACGGAAGAGGAAATCGA GATGATGAAGATGATGGGCTTTGCC TCTTTTGACACAACTAAAGGC		ENSGALT00000022512.1 / Gallus gallus finished cDNA, clone ChEST496a19 / gPGC_EST03546 Embryonic gonadal PGC cDNA Library Gallus gallus cDNA 5', mRNA sequence / Finished cDNA, clone ChEST361a23 / --- / --- / --- / --- / --- / gPGC_EST03546 Embryonic gonadal PGC cDNA Library Gallus gallus cDNA 5', mRNA sequence / Finished cDNA, clone ChEST361a23
RIGG19327	2.13	0.0463	AATACGTGACCTTGCAATCCGTTAG GAATGAAGTCAGTTACCTAAAACAT GGGATTCCACTTCTCCCACT	HNK-1 sulfotransferase (Fragment). [Source:SPTREMBL;Acc:Q8AYG8]	HNK-1 sulfotransferase (Fragment). [Source:SPTREMBL / UP|CHSTA_CHICK (Q5ZIE4) Carbohydrate sulfotransferase 10 (HNK-1 sulfotransferase) (HNK1ST) (HNK-1ST) , complete / Gallus gallus mRNA for hypothetical protein, clone 27h10 / HNK-1 sulfotransferase / CHST10, RCJMB04_27h10: Carbohydrate sulfotransferase 10 / PREDICTED: Gallus gallus HNK-1 sulfotransferase (LOC395206), mRNA. / PREDICTED: similar to HNK-1 sulfotransferase; / HNK-1 sulfotransferase; / --- / Gallus gallus mRNA for hypothetical protein, clone 27h10 / HNK-1 sulfotransferase
RIGG14459	2.13	0.00992	TTTCTCACCTTCCAGCTCGTCCTCT GTATCTTTGCTTCCACTGATGAACG CAGGGAGGACAACGT		ENSGALT00000013607.1 / GB|AJ250343.1|CAC13960.1 putative aquaporin [Gallus gallus] / Gallus gallus partial aqp2 gene for putative aquaporin / Partial aqp2 gene for putative aquaporin / AQP5: Aquaporin 5 homologue (Fragment) / --- / --- / --- / --- / Gallus gallus partial aqp2 gene for putative aquaporin / Partial aqp2 gene for putative aquaporin
RIGG03258	2.118	0.00823	CATTGCTTGCTAAAGCTCCATCTGC CGTGTGTTTTACACCTGAAGGCTCT TTGTTCTTCACCTGTCTGCC	Partial Contig Hit 003612.1	Partial Contig Hit 003612.1 / GB|CR387565.1|CR387565.1 Gallus gallus finished cDNA, clone ChEST46a22 / Gallus gallus finished cDNA, clone ChEST46a22 / Finished cDNA, clone ChEST46a22 / --- / --- / --- / --- / --- / Gallus gallus finished cDNA, clone ChEST46a22 / Finished cDNA, clone ChEST46a22

Gene Name	Fold Change	p-value	SEQUENCE	Array Description	Blast/Database Description
RIGG08866	2.116	0.0349	TACAAGCGCCAGTGGAAGATGGCCTTCTTTTATGCAGATCTACTAAGCAAAGAAAACACCTGGTCGAAGG		ENSGALG00000010540.1 / --- / gPGC_EST08907 Embryonic gonadal PGC cDNA Library Gallus gallus cDNA 5', mRNA sequence / Transcribed locus, weakly similar to XP_513400.1 PREDICTED: similar to RIKEN cDNA 4922503N01 [Pan troglodytes] / similar to RIKEN cDNA 4922503N01 gene / PREDICTED: Gallus gallus similar to RIKEN cDNA 4922503N01 (LOC424632), mRNA. / PREDICTED: similar to RIKEN cDNA 4922503N01; / --- / --- / gPGC_EST06606 Embryonic gonadal PGC cDNA Library Gallus gallus cDNA 5', mRNA sequence / Finished cDNA, clone ChEST530m4
RIGG02391	2.112	0.0377	GGGTTTTCACCTTTCACTGCACTTTAATGGGATTTCTCATCAATGTCTGCATGTTCTTGGCCACCTGTTT	Weakly similar to BAC78184 (BAC78184) ZNFB7 protein	Weakly similar to BAC78184 (BAC78184) ZNFB7 protein / Gallus gallus finished cDNA, clone ChEST334j22 / Gallus gallus finished cDNA, clone ChEST334j22 / Finished cDNA, clone ChEST19n21 / --- / --- / --- / --- / --- / Gallus gallus finished cDNA, clone ChEST334j22 / Finished cDNA, clone ChEST19n21
RIGG02745	2.111	0.0255	CTGCAGTTCTTGGTTTATGTATTGCGATGATTTGTTACAAAGCCCTCTGGATGTATTTGTGCTGGAAACC	Genome Hit Contig10.40	Genome Hit Contig10.40 / Gallus gallus finished cDNA, clone ChEST383l15 / Gallus gallus finished cDNA, clone ChEST961i9 / Finished cDNA, clone ChEST847k9 / --- / --- / --- / --- / --- / Gallus gallus finished cDNA, clone ChEST961i9 / Finished cDNA, clone ChEST847k9
RIGG02058	2.108	0.0246	CTGTCTCCAGCTAAATACTCCTCTTGTCCTTACACTGCCAAGACATGGGCTACTGTATACCTCACCTGAG	Weakly similar to AAQ23621 (AAQ23621) LD04013p	Weakly similar to AAQ23621 (AAQ23621) LD04013p / homologue to UP|BTG2_RAT (P27049) BTG2 protein (NGF-inducible anti-proliferative protein PC3), partial (65%) / --- / --- / --- / PREDICTED: Gallus gallus similar to BTG2 protein (NGF-inducible anti-proliferative protein PC3) (LOC419932), mRNA. / PREDICTED: similar to BTG2 protein (NGF-inducible anti-proliferative protein PC3); / --- / --- / --- / ---
RIGG09559	2.102	0.0215	GAATGTTGTAGTAACAATAGCCTCACAGACATTTGCTTTTCGTACAACACTAACTTGGACAGCCATCGGT		ENSGALG00000016382.1 / --- / --- / --- / / --- / --- / --- / membrane-bound transcription factor peptidase, site 2; / --- / ---
RIGG06057	2.1	0.004	TGTATGACCTTGGCTTGATTCCTCTGAAGTCAGTAGAGAAAGCATGGGTGGAGCGTGGAGAAAATTGGCTT	Contig Hit 031250.1	Contig Hit 031250.1 / Gallus gallus finished cDNA, clone ChEST839f6 / Gallus gallus finished cDNA, clone ChEST839f6 / Finished cDNA, clone ChEST839f6 / --- / --- / --- / --- / --- / Gallus gallus finished cDNA, clone ChEST839f6 / Finished cDNA, clone ChEST839f6
RIGG07958	2.1	0.0118	TTTGTGGAGAAGCCCTTCCTGGAGAAAGTTAAAACCATTCTCAAGCCAGAAGGGGTCTTTGTGCTCAACC		ENSGALG00000003206.1 / --- / --- / --- / --- / PREDICTED: Gallus gallus similar to CGI-01 protein isoform 1 (LOC431420), partial mRNA. / PREDICTED: similar to CGI-01 protein isoform 1, partial; / --- / --- / --- / ---
RIGG19429	2.098	0.0321	AAACTCTCGCATGTGCATGTCACCTCTTGCAGAAAGTAGTGCAATTCCGTGCGACAACAGCAGTATTCAG		ENSGALT00000027371.1 / similar to UP|Q5W0P4_HUMAN (Q5W0P4) OTTHUMP00000018495, partial (54%) / Gallus gallus mRNA for hypothetical protein, clone 8c5 / Hypothetical protein, clone 8c5 / --- / --- / --- / Hypothetical protein, clone 8c5; / --- / Gallus gallus mRNA for hypothetical protein, clone 8c5 / Hypothetical protein, clone 8c5

Gene Name	Fold Change	p-value	SEQUENCE	Array Description	Blast/Database Description
RIGG00842	2.096	0.038	TGTCCAGATGGACCTACAAGGATTGCATTGTAGCAAAGGTGTATGAAGATCATGTAGCAGACTAAGGACA	Partial Contig Hit 505794.1	Partial Contig Hit 505794.1 / Gallus gallus finished cDNA, clone ChEST1033b2 / Gallus gallus finished cDNA, clone ChEST136p14 / Finished cDNA, clone ChEST136p14 / --- / --- / --- / --- / --- / --- / ---
RIGG05542	2.091	0.0205	CAAATAAGATGACTTAGTTGCCAACTGTGGAATTCAGCAATTTGAGGCTTCCATTTTGTTGCCCATCTTG	Weakly similar to Q8MV48 (Q8MV48) UDP-N-acetylgalactosamine	Weakly similar to Q8MV48 (Q8MV48) UDP-N-acetylgalactosamine / homologue to GB|BAB13699.2|10336504|AB040672 UDP-GalNAc: polypeptide N-acetylgalactosaminyltransferase {Homo sapiens} (exp=-1; wgp=0; cg=0), partial (29%) / Gallus gallus finished cDNA, clone ChEST767e5 / Finished cDNA, clone ChEST767e5 / --- / --- / --- / --- / --- / Gallus gallus finished cDNA, clone ChEST767e5 / Finished cDNA, clone ChEST767e5
RIGG14982	2.086	0.0253	TGCCTGGTGTGAGTAATGTGACAAAGGAGGATGTTATCATGAGAGAAATCATCCATCTACTTTGTATTGAGCC		ENSGALT00000015048.1 / Gallus gallus finished cDNA, clone ChEST439h13 / Gallus gallus finished cDNA, clone ChEST439h13 / Finished cDNA, clone ChEST439h13 / --- / PREDICTED: Gallus gallus similar to ubiquitin protein ligase E3 component n-recognin 1; ubiquitin ligase E3 alpha-I (LOC423242), mRNA. / PREDICTED: similar to ubiquitin protein ligase E3 component n-recognin 1; ubiquitin ligase E3 alpha-I; / Finished cDNA, clone ChEST439h13; / --- / Gallus gallus finished cDNA, clone ChEST439h13 / Finished cDNA, clone ChEST439h13
RIGG09744	2.08	0.0384	GAATTCTCCTGGCTTTGATTGAAGGAGCTGGTATTCTCTTAACAAGATTTGCCTCCGCACAGTTTCAGAA		ENSGALT00000000123.1 / RF|NP_001026197.1|71895925|NM_001031026 translocase of inner mitochondrial membrane 17 homolog A {Gallus gallus} (exp=-1; wgp=0; cg=0), complete / Gallus gallus mRNA for hypothetical protein, clone 18k10 / Translocase of inner mitochondrial membrane 17 homolog A (yeast) (TIMM17A), mRNA / RCJMB04_18k10: Hypothetical protein / PREDICTED: Gallus gallus similar to Mitochondrial import inner membrane translocase subunit TIM17 A (Inner membrane preprotein translocase Tim17a) (LOC421147), mRNA. / PREDICTED: similar to Mitochondrial import inner membrane translocase subunit TIM17 A (Inner membrane preprotein translocase Tim17a); / Translocase of inner mitochondrial membrane 17 homolog A (yeast) (TIMM17A), mRNA; / --- / Gallus gallus mRNA for hypothetical protein, clone 18k10 / Translocase of inner mitochondrial membrane 17 homolog A (yeast) (TIMM17A), mRNA
RIGG07514	2.078	0.0468	CTGTCCCTGCTGGTTCCAAGTAACTGCTGCTAACATGACACCCAGATTCCTTATTTCAATAGTCTTGTCATG		Contig_66_reverse / UP|HSP47_CHICK (P13731) 47 kDa heat shock protein precursor (Collagen-binding protein 1), complete / Gallus gallus serpin peptidase inhibitor, clade H (heat shock protein 47), member 1, (collagen binding protein 1) (SERPINH1), mRNA / Hsp47, heat shock protein 47 / --- / Gallus gallus heat shock protein 47 (HSP47), mRNA. / heat shock protein 47; / Hsp47, heat shock protein 47; / --- / Gallus gallus serpin peptidase inhibitor, clade H (heat shock protein 47), member 1, (collagen binding protein 1) (SERPINH1), mRNA / Hsp47, heat shock protein 47

Gene Name	Fold Change	p-value	SEQUENCE	Array Description	Blast/Database Description
RIGG03350	2.078	0.0378	GCAGCCTCTTCTAGCTGGCGAAGAAGTTCTTTGAAGTCATCTGCCATAGACTCGTATTCGTTATAGTCATTTA	Weakly similar to Q9DEP8 (Q9DEP8) Relaxin-like protein	Weakly similar to Q9DEP8 (Q9DEP8) Relaxin-like protein / Gallus gallus finished cDNA, clone ChEST486l24 / 603219583F1 CSEQRBN10 Gallus gallus cDNA clone ChEST211f22 5', mRNA sequence / Finished cDNA, clone ChEST486l24 / / PREDICTED: Gallus gallus similar to relaxin 3 preproprotein; insulin-like 7 (LOC427223), mRNA. / PREDICTED: similar to relaxin 3 preproprotein; insulin-like 7; / --- / --- / 603219583F1 CSEQRBN10 Gallus gallus cDNA clone ChEST211f22 5', mRNA sequence / Finished cDNA, clone ChEST486l24
RIGG05779	2.076	0.0259	TGTCCTGTGCACTAAATGTTGTGCCGTACTGTTGAAAGCGCTTCTCAAGATCAGCATTAAAGGTATAATCT	Genome Hit Contig63.134	Genome Hit Contig63.134 / --- / Gallus gallus finished cDNA, clone ChEST800g21 / Finished cDNA, clone ChEST800g21 / --- / --- / --- / --- / --- / Gallus gallus finished cDNA, clone ChEST800g21 / Finished cDNA, clone ChEST800g21
RIGG00581	2.072	0.0218	GCAAGGAATCTCACCAATCCAAATACTGTGATAATCCTCATAGGAAATAAAGCAGATCTGGAAGCACAGAGGG	Similar to Q7ZXI8 (Q7ZXI8) Hypothetical protein	Similar to Q7ZXI8 (Q7ZXI8) Hypothetical protein / UP|Q52NJ6_PIG (Q52NJ6) Ras-related protein Rab-14, complete / Gallus gallus mRNA for hypothetical protein, clone 9b24 / RAB14, member RAS oncogene family (RAB14), mRNA / RAB14, RCJMB04_9b24: Ras-related protein Rab-14 / PREDICTED: Gallus gallus similar to GTPase Rab14 (LOC417119), mRNA. / PREDICTED: similar to GTPase Rab14; / RAB14, member RAS oncogene family (RAB14), mRNA; / --- / Gallus gallus mRNA for hypothetical protein, clone 9b24 / RAB14, member RAS oncogene family (RAB14), mRNA
RIGG04251	2.066	0.0343	CTGACCACGACTTCCCACTCCAATTAACAAATGCTTCTTCTCTGACTGATTACTTTGCTACCGTCTCAAT	Similar to Q8R238 (Q8R238) RIKEN cDNA 4432411H13 gene	Similar to Q8R238 (Q8R238) RIKEN cDNA 4432411H13 gene / Gallus gallus finished cDNA, clone ChEST601o1 / Gallus gallus finished cDNA, clone ChEST804f18 / Finished cDNA, clone ChEST599n15 / / PREDICTED: Gallus gallus similar to Serine dehydratase related sequence 1 (LOC417030), mRNA. / PREDICTED: similar to Serine dehydratase related sequence 1; / Finished cDNA, clone ChEST599n15; / --- / Gallus gallus finished cDNA, clone ChEST804f18 / Finished cDNA, clone ChEST599n15
RIGG00511	2.062	0.0415	GGTGTACTTGCAGGACCCTCTCACTTAAGCACTTTTAAGAATCCCTTTAAGGTCAGCTTGAAAAGTTAGGC	Weakly similar to Q9H204 (Q9H204) Hypothetical protein (Tumor ANGIOGENESIS ma	Weakly similar to Q9H204 (Q9H204) Hypothetical protein (Tumor ANGIOGENESIS ma / Gallus gallus finished cDNA, clone ChEST1003h20 / Gallus gallus finished cDNA, clone ChEST1003h20 / Finished cDNA, clone ChEST572b4 / --- / PREDICTED: Gallus gallus similar to endothelial-derived gene 1; endothelial-derived gene (LOC425350), mRNA. / PREDICTED: similar to endothelial-derived gene 1; endothelial-derived gene; / --- / --- / Gallus gallus finished cDNA, clone ChEST1003h20 / Finished cDNA, clone ChEST572b4
RIGG04977	2.06	0.00679	GCTGTGTCTAGGTCTCTTACGGAGGTTACAGGGTACAAATGCATAGCAATACAGTATCTGGGAAGCAGAG	Genome Hit Contig164.44	Genome Hit Contig164.44 / Gallus gallus finished cDNA, clone ChEST696p5 / Gallus gallus finished cDNA, clone ChEST696p5 / Finished cDNA, clone ChEST696p5 / --- / --- / --- / --- / --- / Gallus gallus finished cDNA, clone ChEST696p5 / Finished cDNA, clone ChEST696p5

Gene Name	Fold Change	p-value	SEQUENCE	Array Description	Blast/Database Description
RIGG05912	2.057	0.0247	CCAGGTTTTCAGGTGATGCGCGTTG GTACAGAGCTGTTGTTCTAAAAGTTT CTCAGTCCACAGTTGAAGTACTGT	Weakly similar to Q9FLT0 (Q9FLT0) Transcription factor-like protein (100 kDa	Weakly similar to Q9FLT0 (Q9FLT0) Transcription factor-like protein (100 kDa / Gallus gallus finished cDNA, clone ChEST818o3 / 603215073F1 CSEQRBN14 Gallus gallus cDNA clone ChEST200a20 5', mRNA sequence / Finished cDNA, clone ChEST818o3 / / --- / --- / --- / tudor domain containing 1; / 603215073F1 CSEQRBN14 Gallus gallus cDNA clone ChEST200a20 5', mRNA sequence / Finished cDNA, clone ChEST818o3
RIGG19047	2.05	0.04	CCTCAGAGCTCATCATGACTAATGT AGACCAAATCAGCACGCAGATATCA CGAGAGAGGAAAATGGAGAC		ENSGALT00000026259.1 / --- / gPGC_EST07896 Embryonic gonadal PGC cDNA Library Gallus gallus cDNA 5', mRNA sequence / Transcribed locus, weakly similar to XP_001061435.1 PREDICTED: hypothetical protein [Rattus norvegicus] / --- / PREDICTED: Gallus gallus similar to chromosome X open reading frame 21; 5430427O19Rik (LOC418590), mRNA. / PREDICTED: similar to chromosome X open reading frame 21; 5430427O19Rik; / --- / --- / --- / ---
RIGG00837	2.047	0.0147	AGTAGAGGACCCACCCACTCTGATC CTTCTTCTACTAACTTATTTACCATC CTCATGTTATTGAGGCAAG	Contig Hit 507533.1	Contig Hit 507533.1 / Gallus gallus finished cDNA, clone ChEST1032g14 / Gallus gallus finished cDNA, clone ChEST1032g14 / Finished cDNA, clone ChEST1032g14 / --- / --- / --- / --- / --- / Gallus gallus finished cDNA, clone ChEST1032g14 / Finished cDNA, clone ChEST1032g14
RIGG02968	2.043	0.0392	CTGTTCATTTTCTGAAGCCTGTGGG AGCGCCAAGCAATACTCCTGAACTT TGTTTAATAAATGTCTTTGA	Genome Hit Contig13.254	Genome Hit Contig13.254 / Gallus gallus finished cDNA, clone ChEST416f22 / Gallus gallus finished cDNA, clone ChEST416f22 / Finished cDNA, clone ChEST416f22 / --- / --- / --- / --- / --- / Gallus gallus finished cDNA, clone ChEST416f22 / Finished cDNA, clone ChEST416f22
RIGG15831	2.042	0.00714	TGCTCAACTGAACTCCATCCATCTT CGGTCCATTACGCAGTAACAGAACA GCAAGATCAGTGTCAACACT		ENSGALT00000017347.1 / --- / --- / --- / / --- / --- / --- / --- / --- / ---
RIGG02088	2.04	0.0465	AGAGTCAGGATTATAATTAGATGCA ATCCCTGTGTGTGAATCTCAGTTCT GCTATTTGTCATGGACCTGATCC	Genome Hit Contig42.11	Genome Hit Contig42.11 / Gallus gallus finished cDNA, clone ChEST282m15 / 603372763F1 CSEQRBN20 Gallus gallus cDNA clone ChEST282m15 5', mRNA sequence / Finished cDNA, clone ChEST282m15 / --- / --- / --- / --- / transmembrane protein 166; / 603372763F1 CSEQRBN20 Gallus gallus cDNA clone ChEST282m15 5', mRNA sequence / Finished cDNA, clone ChEST282m15
RIGG19546	2.038	0.0324	AAACCGTATTTCAGGAAGCAGAATT GGAAAGCTGTGATGTTAATACCATG CTGCTGGTACATTTCTTTGG		ENSGALT00000027664.1 / --- / --- / --- / hypothetical protein / PREDICTED: Gallus gallus similar to Smhs2 homolog (LOC418946), mRNA. / PREDICTED: similar to Smhs2 homolog; / Finished cDNA, clone ChEST790k4; / --- / 603808114F1 CSEQCHN74 Gallus gallus cDNA clone ChEST790k4 5', mRNA sequence / Finished cDNA, clone ChEST790k4

Gene Name	Fold Change	p-value	SEQUENCE	Array Description	Blast/Database Description
RIGG09734	2.037	0.0318	GAAAATTAAGGAATGCTTTGTTTGA GTGAATCCCTGTGTTTCAGTCTCTC CACACAAGCCCCTGTGCACA		ENSGALT00000000101.1 / Gallus gallus finished cDNA, clone ChEST912m14 / Gallus gallus finished cDNA, clone ChEST912m14 / Finished cDNA, clone ChEST912m14 / similar to forkhead transcription factor i3b, partial / --- / --- / --- / --- / Gallus gallus finished cDNA, clone ChEST912m14 / Finished cDNA, clone ChEST912m14
RIGG01653	2.034	0.0237	GAATGTGAGGCAAGAATACAAATTG GACTTCACTACAACATCCCCTACCC AAGACCTATCCATCTGCCTC	Weakly similar to CAA78050 (CAA78050) Hypothetical protein R08D7.4	Weakly similar to CAA78050 (CAA78050) Hypothetical protein R08D7.4 / Gallus gallus finished cDNA, clone ChEST222k20 / Gallus gallus finished cDNA, clone ChEST770e7 / Finished cDNA, clone ChEST770e7 / --- / PREDICTED: Gallus gallus similar to RIKEN cDNA 4930404J24 (LOC427003), mRNA. / PREDICTED: similar to RIKEN cDNA 4930404J24; / --- / --- / Gallus gallus finished cDNA, clone ChEST770e7 / Finished cDNA, clone ChEST770e7
RIGG03781	2.034	0.00137	GGCAGTGCAATGAAAGCACAGGTAT ACATGCTGCTCCAAAGCTCTCTCTC CTAAGCAGTGATTATCATTA	Genome Hit Contig8.18	Genome Hit Contig8.18 / Gallus gallus finished cDNA, clone ChEST544n9 / --- / --- / --- / --- / --- / --- / --- / --- / ---
RIGG20339	2.033	0.0407	GAGGCATTCAAGAACATCCTTACAG AAATCTACCGCATTGTTTCTCAGAA GCAGATTGCAGACCGGTCTG	Gallus gallus similar to GTP-binding protein ora3 - electric ray (Discopyge ommata) (LOC420063), mRNA	Gallus gallus similar to GTP-binding protein ora3 - electric ray (Discopyge ommata) (LOC420063), mRNA / RF|NP_001012569.1|60302752|NM_001012551 RAB11B, member RAS oncogene family {Gallus gallus} (exp=-1; wgp=0; cg=0), complete / Gallus gallus mRNA for hypothetical protein, clone 8i9 / RAB11B, member RAS oncogene family (RAB11B), mRNA / RCJMB04_8i9: Hypothetical protein / PREDICTED: Gallus gallus similar to GTP-binding protein ora3 - electric ray (Discopyge ommata) (LOC420063), mRNA. / PREDICTED: similar to GTP-binding protein ora3 - electric ray (Discopyge ommata); / RAB11B, member RAS oncogene family (RAB11B), mRNA; / --- / Gallus gallus mRNA for hypothetical protein, clone 8i9 / RAB11B, member RAS oncogene family (RAB11B), mRNA
RIGG18463	2.033	0.0272	CACCGCTCATGGACATGGAAATGCC CTTCACTCCACCACGAGTCTCCTTT TCATTCTCTCAGTTTCTCTTC		ENSGALT00000024724.1 / Gallus gallus finished cDNA, clone ChEST387i20 / Gallus gallus finished cDNA, clone ChEST76p7 / Finished cDNA, clone ChEST387i20 / --- / --- / --- / --- / --- / --- / ---
RIGG03280	2.031	0.0304	TTCTCAGCCTTCTTAGTGCTTCCACA GGCAAGACCATCTGGACACTGAACT CCATTCACCTTTCAGATGG	Similar to Q8BYI8 (Q8BYI8) Hypothetical quinoprotein alcohol dehydroge	Similar to Q8BYI8 (Q8BYI8) Hypothetical quinoprotein alcohol dehydroge / Gallus gallus finished cDNA, clone ChEST446a20 / Gallus gallus mRNA for hypothetical protein, clone 14d19 / Similar to RIKEN cDNA 8430419L09 (LOC417963), mRNA / --- / PREDICTED: Gallus gallus similar to RIKEN cDNA 8430419L09 (LOC417963), mRNA. / PREDICTED: similar to RIKEN cDNA 8430419L09; / Similar to RIKEN cDNA 8430419L09 (LOC417963), mRNA; / --- / Gallus gallus mRNA for hypothetical protein, clone 14d19 / Similar to RIKEN cDNA 8430419L09 (LOC417963), mRNA

Gene Name	Fold Change	p-value	SEQUENCE	Array Description	Blast/Database Description
RIGG04900	2.029	0.0459	AAAGCCATCCTTCCCTCATGTTAAGTGGTGATCAAATTCATTCTCTTTGCTTCACTCTTTAACATGGTCGG	Weakly similar to Q7ZX53 (Q7ZX53) Similar to fly-like putative organic ion tr	Weakly similar to Q7ZX53 (Q7ZX53) Similar to fly-like putative organic ion tr / Gallus gallus finished cDNA, clone ChEST688f11 / Gallus gallus finished cDNA, clone ChEST688f11 / Finished cDNA, clone ChEST688f11 / --- / --- / --- / --- / --- / Gallus gallus finished cDNA, clone ChEST688f11 / Finished cDNA, clone ChEST688f11
RIGG13700	2.025	0.00715	CCTCTGTTGTTTCACCAAAGGGAAATGAGGCATTATAAAACAACACGGACATTGCAGCCCAATCCCTGAC		ENSGALT00000011437.1 / --- / --- / --- / --- / --- / --- / --- / --- / --- / ---
RIGG00301	2.021	0.0169	AGGGGAAGGCAGATGGTTAGTCTTTGGCAGGACATAATGCTTTAATAAAGAAAGGAGCAAATGATCATCA	Gallus gallus mRNA for hypothetical protein, clone 24n6	Gallus gallus mRNA for hypothetical protein, clone 24n6 / similar to UP|STXB3_MOUSE (Q60770) Syntaxin-binding protein 3 (UNC-18 homolog 3) (UNC-18C) (MUNC-18-3), partial (94%) / Gallus gallus mRNA for hypothetical protein, clone 24n6 / Hypothetical protein, clone 24n6 / RCJMB04_24n6: Hypothetical protein / --- / --- / Hypothetical protein, clone 24n6; / --- / Gallus gallus mRNA for hypothetical protein, clone 24n6 / Hypothetical protein, clone 24n6
RIGG08957	2.011	0.0343	CTCCTTGTCTCTTGTGGTCACCTACCCTCTGATGAAGAGAATAACATATTGGCCACAGTTAGTTTTGGGA		ENSGALG00000011196.1 / similar to GB|CAF18241.1|51870555|AJ621061 para-hydroxybenzoate--polyprenyltransferase, mitochondrial precursor {Homo sapiens} (exp=-1; wgp=0; cg=0), partial (46%) / 603598727F1 CSEQCHN54 Gallus gallus cDNA clone ChEST570e7 5', mRNA sequence / Transcribed locus, moderately similar to XP_526567.1 PREDICTED: similar to CL640 protein [Pan troglodytes] / --- / PREDICTED: Gallus gallus similar to hypothetical protein CL640 (LOC422605), mRNA. / PREDICTED: similar to hypothetical protein CL640; / --- / --- / testis_EST00821 Testis cDNA Library Gallus gallus cDNA 3', mRNA sequence / Transcribed locus, moderately similar to XP_573556.2 PREDICTED: similar to para-hydroxybenzoate-polyprenyltransferase, mitochondrial [Rattus norvegicus]
RIGG01848	2.007	0.023	GGGTTGTGGAGACCTCACGGCTTCTTGGGACTCATCACCAGTGCCTGAGAGAGGAATACAGCTCT	Weakly similar to Q9LIK1 (Q9LIK1) Similarity to ADP-ribosylation factor (Puta	Weakly similar to Q9LIK1 (Q9LIK1) Similarity to ADP-ribosylation factor (Puta / Gallus gallus finished cDNA, clone ChEST250h10 / Gallus gallus finished cDNA, clone ChEST585a23 / Finished cDNA, clone ChEST250h10 / hypothetical protein, partial / --- / --- / Finished cDNA, clone ChEST250h10; / --- / --- / ---
RIGG19039	1.999	0.0469	CTGGGTGAGACAAAGTGGACAGAGCTCTAATCTGGGGATCAGTATGAGTGACTTTCATTTACAATGACTTA		ENSGALT00000026241.1 / --- / --- / --- / --- / --- / --- / --- / --- / --- / ---

Gene Name	Fold Change	p-value	SEQUENCE	Array Description	Blast/Database Description
RIGG15787	1.999	0.029	CAGGATTGCCATAAACCTCAGGTGACAGACAAGGAAGTGAATGATCCTCGGCTTGTATGGTACTGTGCCC		ENSGALT00000017210.1 / similar to UP|PHF22_MOUSE (Q9D168) Integrator complex subunit 12 (PHD finger protein 22), partial (44%) / gPGC_EST01098 Embryonic gonadal PGC cDNA Library Gallus gallus cDNA 5', mRNA sequence / Finished cDNA, clone ChEST33i24 / hypothetical protein / PREDICTED: Gallus gallus similar to RIKEN cDNA 1110020M19 (LOC422537), mRNA. / PREDICTED: similar to RIKEN cDNA 1110020M19; / --- / --- / gPGC_EST01098 Embryonic gonadal PGC cDNA Library Gallus gallus cDNA 5', mRNA sequence / Finished cDNA, clone ChEST33i24
RIGG00407	1.999	0.0483	CACCAACGGGAACTTTAATATTGGGACACTTCAGATCTGACCTGAGTGGAGTTTACACTTGTTCTCTTGTTTAT	Gallus gallus mRNA for hypothetical protein, clone 11c4	Gallus gallus mRNA for hypothetical protein, clone 11c4 / --- / Gallus gallus mRNA for hypothetical protein, clone 11c4 / Hypothetical protein, clone 11c4 / RCJMB04_11c4: Hypothetical protein / PREDICTED: Gallus gallus zona pellucida binding protein 1 (ZPBP1), mRNA. / PREDICTED: similar to zona pellucida binding protein 1; / --- / --- / Gallus gallus mRNA for hypothetical protein, clone 11c4 / Hypothetical protein, clone 11c4
RIGG19545	1.993	0.0262	ATAAACACGTGGACACTGGAAGACGATACTACGTGGCGTTAAATAAAGATGGAACTCCAAGAGAAGGGAC		ENSGALT00000027663.1 / UP|Q7ZZN4_CHICK (Q7ZZN4) Fibroblast groth factor 9, complete / Gallus gallus Fgf9 mRNA for fibroblast groth factor 9, complete cds / Fibroblast growth factor 9 (glia-activating factor) (FGF9), mRNA / Fgf9: Fibroblast groth factor 9 / --- / --- / --- / --- / Gallus gallus Fgf9 mRNA for fibroblast groth factor 9, complete cds / Fibroblast growth factor 9 (glia-activating factor) (FGF9), mRNA
RIGG09678	1.991	0.046	TAACAAAGGGCAGGTTCGCTTTGATGAACAGGAAAGTTTTAGATGTATTCGGTACTGAAGCATCCAGGAAC		ENSGALG00000017241.1 / --- / --- / --- / --- / PREDICTED: Gallus gallus similar to hypothetical protein FLJ22104 (LOC419015), mRNA. / PREDICTED: similar to hypothetical protein FLJ22104; / --- / --- / --- / ---
RIGG00048	1.991	0.0296	TCACTCCGAACTGTCTTGGATTTTAGGACCGGTATGCTCCATCACTTTATAACTGGAATGTACATGGTATT	Gallus gallus mRNA for hypothetical protein, clone 2c16	Gallus gallus mRNA for hypothetical protein, clone 2c16 / RF|NP_001025838.1|71897241|NM_001030667 D-dopachrome tautomerase {Gallus gallus} (exp=0; wgp=1; cg=0), complete / Gallus gallus mRNA for hypothetical protein, clone 2c16 / D-dopachrome tautomerase (DDT), mRNA / --- / PREDICTED: Gallus gallus similar to D-dopachrome tautomerase (Phenylpyruvate tautomerase II) (LOC416937), mRNA. / PREDICTED: similar to D-dopachrome tautomerase (Phenylpyruvate tautomerase II); / D-dopachrome tautomerase (DDT), mRNA; / --- / Gallus gallus mRNA for hypothetical protein, clone 2c16 / D-dopachrome tautomerase (DDT), mRNA
RIGG09649	1.986	0.0344	AATGGCAACAGGTTCGGCAAGAATTAGTCGAAGAATATGAGCAAGTTAAGAGCATTGTCGACACTTTAGAGAG		ENSGALG00000017083.1 / similar to UP|KATL1_HUMAN (Q9BW62) Katanin p60 ATPase-containing subunit A-like 1 (Katanin p60 subunit A-like 1) (p60 katanin-like 1) , partial (68%) / Gallus gallus p60 katanin mRNA, complete cds / P60 katanin / / PREDICTED: Gallus gallus similar to katanin p60 subunit A-like 1 (LOC418920), mRNA. / PREDICTED: similar to katanin p60 subunit A-like 1; / --- / --- / --- / ---

Gene Name	Fold Change	p-value	SEQUENCE	Array Description	Blast/Database Description
RIGG05422	1.985	0.0445	TGCACTACAGAAGCTCACAAACAGAGAATGGCACTGATTCGCAAAACCACCAAGAAATAACCCCTGGGAG	Weakly similar to O13722 (O13722) Hypothetical protein	Weakly similar to O13722 (O13722) Hypothetical protein / Gallus gallus finished cDNA, clone ChEST753d3 / Gallus gallus finished cDNA, clone ChEST636h22 / Finished cDNA, clone ChEST636h22 / RCJMB04_8m22: Hypothetical protein / --- / --- / --- / --- / gPGC_EST06596 Embryonic gonadal PGC cDNA Library Gallus gallus cDNA 5', mRNA sequence / TAF12 RNA polymerase II, TATA box binding protein (TBP)-associated factor, 20kDa (TAF12), mRNA
RIGG06274	1.983	0.0464	GGCAGACATTGCTGTTGCTTTGATCTCTCCAGATAACTAGTTTCTACTCTACTGTGCCTTGTGGAATTAA	Similar to Q8CAN0 (Q8CAN0) Protein tyrosine phosphatase	Similar to Q8CAN0 (Q8CAN0) Protein tyrosine phosphatase / Gallus gallus finished cDNA, clone ChEST869j10 / Gallus gallus finished cDNA, clone ChEST869j10 / Finished cDNA, clone ChEST869j10 / hypothetical protein / PREDICTED: Gallus gallus similar to PTPN5 protein (LOC423085), mRNA. / PREDICTED: similar to PTPN5 protein; / Transcribed locus, weakly similar to XP_509221.1 PREDICTED: protein tyrosine phosphatase, receptor type, R [Pan troglodytes]; / --- / Gallus gallus finished cDNA, clone ChEST869j10 / Finished cDNA, clone ChEST869j10
RIGG08389	1.976	0.0409	GTTTGCCATCGACGAAATCAACAGAGACAACTACTTGCTGCCAGGAATTAAACTTGGAGTTCACATCTTG		ENSGALG00000006576.1 / homologue to UP|Q1ZZH1_MACFA (Q1ZZH1) Metabotropic glutamate receptor 3, partial (41%) / --- / --- / / PREDICTED: Gallus gallus similar to Metabotropic glutamate receptor 3 precursor (mGluR3) (LOC418644), mRNA. / PREDICTED: similar to Metabotropic glutamate receptor 3 precursor (mGluR3); / --- / --- / 603852937F1 CSEQCHN62 Gallus gallus cDNA clone ChEST852i10 5', mRNA sequence / Finished cDNA, clone ChEST665j6
RIGG03239	1.976	0.0416	TATATTGTCATTTTACCGTTCCTGGCGCATGTAGGTGTGGGTTGGTTCCTCTGTGTGTGACTCTGACTGC	Partial Contig Hit 010920.1	Partial Contig Hit 010920.1 / Gallus gallus finished cDNA, clone ChEST464n23 / 603522895F1 CSEQCHN68 Gallus gallus cDNA clone ChEST464n23 5', mRNA sequence / Finished cDNA, clone ChEST464n23 / --- / --- / --- / --- / --- / 603522895F1 CSEQCHN68 Gallus gallus cDNA clone ChEST464n23 5', mRNA sequence / Finished cDNA, clone ChEST464n23

Gene Name	Fold Change	p-value	SEQUENCE	Array Description	Blast/Database Description
RIGG13664	1.975	0.0341	CTGGGACAGGTCTATGAAGCTCAGG ATAACATAGAAAAGGCTTTTGGGTG TTACAAGCGATCCGTGGAGC		ENSGALT00000011324.1 / Gallus gallus finished cDNA, clone ChEST1025k21 / --- / --- / similar to Ran-binding protein 2, partial / PREDICTED: Gallus gallus similar to Ran-binding protein 2 (LOC425387), partial mRNA.PREDICTED: Gallus gallus similar to Ran-binding protein 2 (LOC426816), partial mRNA.PREDICTED: Gallus gallus similar to sperm membrane protein BS-63 (LOC429314), partial mRNA.PREDICTED: Gallus gallus similar to Ran-binding protein 2 (LOC429600), partial mRNA.PREDICTED: Gallus gallus similar to Ran-binding protein 2 (LOC429662), partial mRNA.PREDICTED: Gallus gallus similar to RAN binding protein 2 (LOC429668), partial mRNA.PREDICTED: Gallus gallus similar to Ran-binding protein 2 (RanBP2) (Nuclear pore complex protein Nup358) (Nucleoporin Nup358) (358 kDa nucleoporin) (P270) (LOC429938), partial mRNA.PREDICTED: Gallus gallus similar to Ran-binding protein 2 (LOC429950), partial mRNA.PREDICTED: Gallus gallus similar to Ran-binding protein 2 (LOC430199), partial mRNA.PREDICTED: Gallus gallus similar to Ran-binding protein 2 (LOC430215), partial mRNA.PREDICTED: Gallus gallus similar to Ran-binding protein 2 (LOC430296), partial mRNA.PREDICTED: Gallus gallus similar to RAN binding protein 2; nucleoporin 358; nuclear pore complex protein Nup358; P270 (LOC430315), partial mRNA.PREDICTED: Gallus gallus similar to Ran-binding protein 2 (LOC430320), partial mRNA.PREDICTED: Gallus gallus similar to RAN binding
RIGG02004	1.974	0.0188	GCGTTCATAAACCCATTTCTAAACC CTCCTCATTTCTAAAGCAGAGATGA AAGGAGAATGTTTGCAGGTGA	Weakly similar to Q9BLX4 (Q9BLX4) ASH1 protein	Weakly similar to Q9BLX4 (Q9BLX4) ASH1 protein / Gallus gallus finished cDNA, clone ChEST270l24 / Gallus gallus finished cDNA, clone ChEST270l24 / Finished cDNA, clone ChEST69j4 / / --- / --- / --- / --- / Gallus gallus finished cDNA, clone ChEST270l24 / Finished cDNA, clone ChEST69j4
RIGG05256	1.973	0.0176	CTGACCACCTGAAATGGTTACTGAG TGTAACGGTTTAATGCCTTGTTTCCA TTTACGCATCAGAAAGTAA	Similar to O97181 (O97181) Nucleolar protein, putative	Similar to O97181 (O97181) Nucleolar protein, putative / Gallus gallus finished cDNA, clone ChEST58f24 / Gallus gallus finished cDNA, clone ChEST735k13 / Finished cDNA, clone ChEST735k13 / / PREDICTED: Gallus gallus similar to zinc finger protein 330; nucleolar cysteine-rich protein; zinc finger autoantigen 330 (LOC422453), mRNA. / PREDICTED: similar to zinc finger protein 330; nucleolar cysteine-rich protein; zinc finger autoantigen 330; / Finished cDNA, clone ChEST735k13; / --- / Gallus gallus finished cDNA, clone ChEST735k13 / Finished cDNA, clone ChEST735k13
RIGG11739	1.973	0.0116	GCGTACAGCACCAGCGTGATTTCCG GACCCAGAACCGATGAACTTGGGAG ATCTTCTTCGGGCTC		ENSGALT00000005677.1 / --- / Gallus gallus iroquois homeobox protein 2 (IRX2), mRNA / Iroquois homologue-2 / / --- / --- / --- / iroquois homeobox protein 5; / --- / ---
RIGG11316	1.972	0.0105	CAACTTTGTCCACAAGAACGGCATG CTGGATCTCTCGGTAGTCCTGAAGG CTGTTTACTTGGTCCTGAAC		ENSGALT00000004429.1 / --- / --- / --- / --- / PREDICTED: Gallus gallus similar to RIKEN cDNA C030018G13 gene (LOC424002), mRNA. / --- / --- / --- / --- / ---
RIGG05809	1.972	0.0374	TCAGGAGAGAATCTGAATGTCTTAG TAGTATGAGTGACTCCTCACCTTCC CCTAGTCTTCTGCTGCAGAC	Weakly similar to Q9NT21 (Q9NT21) Hypothetical protein (Fragment)	Weakly similar to Q9NT21 (Q9NT21) Hypothetical protein (Fragment) / Gallus gallus finished cDNA, clone ChEST804d6 / Gallus gallus finished cDNA, clone ChEST655p20 / Finished cDNA, clone ChEST804d6 / --- / --- / --- / --- / sperm associated antigen 17; / --- / ---

Gene Name	Fold Change	p-value	SEQUENCE	Array Description	Blast/Database Description
RIGG10360	1.968	0.00893	TCGCCACTTGCAGCCAATTTACTTG AGCAAGGTTTTGAAATAACTTTGTGA CAACCTCCTAATGAAAGCGA		ENSGALT00000001730.1 / --- / --- / --- / --- / --- / --- / --- / --- / --- / ---
RIGG08287	1.959	0.046	GATTTCAGCAATCTCTACAAAATCCT TAAAGAAAGTTCCTGCAGGTGCAGT GTCTCTGTTCCCAGGAGGG		ENSGALG00000005914.1 / similar to UP|FA21C_PONPY (Q5RDC1) Protein FAM21C, partial (48%) / Gallus gallus mRNA for hypothetical protein, clone 36d12 / Hypothetical protein, clone 36d12 / --- / --- / --- / --- / --- / Gallus gallus mRNA for hypothetical protein, clone 36d12 / Hypothetical protein, clone 36d12
RIGG07982	1.959	0.0209	TGACTGCAAAAGAGAAACTGGTGGA TCAGAGTACATGGACCCATCCTCAT TTATGCCCTCCTTTAAGTAA	Prolactin receptor precursor (PRL-R) (CPRLP). [Source:SWISSPROT;Acc:Q04594]	Prolactin receptor precursor (PRL-R) (CPRLP). [Source:SWISSPROT / GB|AF051808.1|AAC08324.1 truncated testis-specific box 1-less prolactin receptor [Gallus gallus] / Gallus gallus prolactin receptor (PRLR), mRNA / Prolactin receptor / --- / --- / --- / --- / --- / Gallus gallus prolactin receptor (PRLR), mRNA / Prolactin receptor
RIGG03351	1.958	0.0353	AGCAGTGGAACGTTAGAGGAGGAC CTAAATTGTCTTGCAAAATACAACAT TCCTAACTTCGTGAACACGG	Similar to Q96G30 (Q96G30) Hypothetical protein (Fragment)	Similar to Q96G30 (Q96G30) Hypothetical protein (Fragment) / Gallus gallus finished cDNA, clone ChEST486m14 / Gallus gallus finished cDNA, clone ChEST486m14 / Finished cDNA, clone ChEST881g15 / --- / PREDICTED: Gallus gallus similar to Chromosome 6 open reading frame 117 (LOC421836), mRNA. / PREDICTED: similar to Chromosome 6 open reading frame 117; / --- / --- / Gallus gallus finished cDNA, clone ChEST486m14 / Finished cDNA, clone ChEST881g15
RIGG02436	1.956	0.0451	AGGCATTATTGTGAAGCACAGACTG CGTAATTCTCAGATTCTCACCATTGT AATTTGAACAGTGCATTGCTG	Weakly similar to Q8IY39 (Q8IY39) Similar to RIKEN cDNA 4933426M09 gene	Weakly similar to Q8IY39 (Q8IY39) Similar to RIKEN cDNA 4933426M09 gene / Gallus gallus finished cDNA, clone ChEST342f4 / Gallus gallus finished cDNA, clone ChEST342f4 / Finished cDNA, clone ChEST342f4 / --- / --- / --- / --- / --- / Gallus gallus finished cDNA, clone ChEST342f4 / Finished cDNA, clone ChEST342f4
RIGG03447	1.949	0.0217	TTGTGAGCCTGACCATCCAGTCCTA GTGCTTGGGAGTTATTTTCTGGCTT TGTCCTGGAGACCCA	Genome Hit Contig50.294	Genome Hit Contig50.294 / Gallus gallus finished cDNA, clone ChEST497p5 / --- / --- / --- / --- / --- / --- / --- / --- / ---
RIGG15719	1.945	0.0393	GAAATAGATGCTGTCCTTTTATTGCA GAGCACACACACTCAGGATTAGAGT CACACTGCAGTTCCACCTG		ENSGALT00000017026.1 / --- / 603212037F1 CSEQRBN13 Gallus gallus cDNA clone ChEST192j9 5', mRNA sequence / Transcribed locus, strongly similar to XP_421290.1 PREDICTED: similar to CG15625-PA [Gallus gallus] / --- / PREDICTED: Gallus gallus similar to CG15625-PA (LOC423376), mRNA. / PREDICTED: similar to CG15625-PA; / --- / --- / 603212037F1 CSEQRBN13 Gallus gallus cDNA clone ChEST192j9 5', mRNA sequence / Transcribed locus, strongly similar to XP_421290.1 PREDICTED: similar to CG15625-PA [Gallus gallus]

Gene Name	Fold Change	p-value	SEQUENCE	Array Description	Blast/Database Description
RIGG00666	1.944	0.0471	TGGGCTGCCAGATAACATTGTGAATTGCTATGAGTTCTTGGCTGTATTACAGGAGAAGCTTGACAATTTGCAG	Similar to Q8BVL5 (Q8BVL5) Zinc finger protein 277 homolog	Similar to Q8BVL5 (Q8BVL5) Zinc finger protein 277 homolog / Gallus gallus finished cDNA, clone ChEST1015f8 / Gallus gallus finished cDNA, clone ChEST485f21 / Finished cDNA, clone ChEST1015f8 / similar to nuclear receptor-interacting factor / PREDICTED: Gallus gallus similar to zinc finger protein (C2H2 type) 277; zinc finger protein 277; nuclear receptor-interacting factor 4 (LOC427859), mRNA. / PREDICTED: similar to zinc finger protein (C2H2 type) 277; zinc finger protein 277; nuclear receptor-interacting factor 4; / Finished cDNA, clone ChEST1015f8; / --- / Gallus gallus finished cDNA, clone ChEST485f21 / Finished cDNA, clone ChEST1015f8
RIGG01829	1.944	0.0414	TTGTGACTGGGAACTTGGCTCCATACTTCCTGACTGTGTGTAAACCCAACTACACTGGAAACGACTGTCG	Similar to Q8TBJ4 (Q8TBJ4) Hypothetical protein	Similar to Q8TBJ4 (Q8TBJ4) Hypothetical protein / homologue to UP|Q8TBJ4_HUMAN (Q8TBJ4) Plasticity related gene 3 (Lipid phosphate phosphatase-related protein type 1) (OTTHUMP00000021801), complete / Gallus gallus finished cDNA, clone ChEST246m3 / Finished cDNA, clone ChEST246m3 / --- / PREDICTED: Gallus gallus similar to plasticity related gene 3; lipid phosphate phosphatase-related protein type 1 (LOC427306), mRNA. / PREDICTED: similar to plasticity related gene 3; lipid phosphate phosphatase-related protein type 1; / Finished cDNA, clone ChEST246m3; / --- / Gallus gallus finished cDNA, clone ChEST246m3 / Finished cDNA, clone ChEST246m3
RIGG19179	1.943	0.00768	AAGGGTCACTATTCCTCTGTAATCTTCCCTTTTCATTTGTATAGAGAGATACTGCAGGAAATCCAGCGGC		ENSGALT00000026670.1 / --- / Gallus gallus mRNA for hypothetical protein, clone 11i20 / Similar to dystrobrevin B DTN-B2 (LOC421992), mRNA / / --- / --- / --- / --- / --- / --- / ---
RIGG04418	1.943	0.00615	GTCGGGATCTCCCCATACGATGTGCACTCCATTAACTAAGTCATTAATGAACCAAGGCATGTTCAATCAT	Similar to CCG1_MOUSE (O70578) Voltage-dependent calcium channel gamma	Similar to CCG1_MOUSE (O70578) Voltage-dependent calcium channel gamma / Gallus gallus finished cDNA, clone ChEST626k17 / Gallus gallus finished cDNA, clone ChEST844b3 / Finished cDNA, clone ChEST626k17 / / PREDICTED: Gallus gallus similar to Voltage-dependent calcium channel gamma-1 subunit (Dihydropyridine-sensitive L-type, skeletal muscle calcium channel gamma subunit) (LOC417428), mRNA. / PREDICTED: similar to Voltage-dependent calcium channel gamma-1 subunit (Dihydropyridine-sensitive L-type, skeletal muscle calcium channel gamma subunit); / Finished cDNA, clone ChEST626k17; / --- / Gallus gallus finished cDNA, clone ChEST844b3 / Finished cDNA, clone ChEST626k17
RIGG01806	1.941	0.0362	TGGCTTCAGAGGCAATTCGAGGGCTTAAGGAATCCAACCCATCTTCATGTTCCTTTTGGTTTGAATATTT	Genome Hit Contig190.52	Genome Hit Contig190.52 / similar to UP|Q9AT40_BRAJU (Q9AT40) Glutathione reductase, partial (3%) / --- / --- / --- / --- / --- / --- / --- / --- / ---
RIGG06758	1.941	0.00756	TTCCTTACCGTTCAGCTCCAGCATCAGAGTTGAAACATGTTCTTGCTAATGCTGCCTGCTTGACAAACTGT	Genome Hit Contig52.144	Genome Hit Contig52.144 / --- / Gallus gallus finished cDNA, clone ChEST936h5 / Finished cDNA, clone ChEST936h5 / --- / --- / --- / --- / --- / Gallus gallus finished cDNA, clone ChEST936h5 / Finished cDNA, clone ChEST936h5

Gene Name	Fold Change	p-value	SEQUENCE	Array Description	Blast/Database Description
RIGG03234	1.938	0.0233	GTAGCACAACGTCCTCCAGACTTTTCCTATAGCTATAGCACAGTTTGCCCTCAATGTCTCCCACAAATGC	Genome Hit Contig70.83	Genome Hit Contig70.83 / Gallus gallus finished cDNA, clone ChEST463p18 / 603589843F1 CSEQCHN74 Gallus gallus cDNA clone ChEST551f11 5', mRNA sequence / Finished cDNA, clone ChEST463p18 / --- / --- / --- / --- / --- / 603589843F1 CSEQCHN74 Gallus gallus cDNA clone ChEST551f11 5', mRNA sequence / Finished cDNA, clone ChEST463p18
RIGG03566	1.934	0.0162	TCTAGGCACACTATGAAAATCCTTCTTGAATCAAATGTCCCCAAGGGAGTCAGAAATTAAAGATGCTCTTCC	Genome Hit Contig217.46	Genome Hit Contig217.46 / --- / 603544410F1 CSEQCHN61 Gallus gallus cDNA clone ChEST518d3 5', mRNA sequence / Finished cDNA, clone ChEST518d3 / --- / --- / --- / --- / --- / 603544410F1 CSEQCHN61 Gallus gallus cDNA clone ChEST518d3 5', mRNA sequence / Finished cDNA, clone ChEST518d3
RIGG08611	1.931	0.0354	TTGCTCAGGGCCTTCTGCAGTCAGATTTTGAATGTTCCCAAGGACACAGTGAATTTTACCTCACTAACAC		ENSGALG00000008445.1-2 / --- / --- / --- / --- / --- / --- / --- / --- / --- / ---
RIGG18484	1.929	0.0424	GGCCTGGATGTTACTGTTGTTGGTGACTAACCTTCCTAAATGGATAGATAGCAAAATGGGAGTCACTCAGC		ENSGALT00000024790.1 / similar to UP|Q96PI2_HUMAN (Q96PI2) P90 autoantigen, partial (90%) / Gallus gallus mRNA for hypothetical protein, clone 1n3 / Similar to KIAA1524 protein (LOC418410), mRNA / RCJMB04_1n3: Hypothetical protein / --- / --- / --- / --- / Gallus gallus mRNA for hypothetical protein, clone 1n3 / Similar to KIAA1524 protein (LOC418410), mRNA
RIGG03233	1.927	0.0371	TCTGGTGTTTAACTCCTATAGTCTCATCGCTTGCAAGAGTAACTAGGCTCTGGTTCTAATGCAACTTTTGTAACT	Weakly similar to BAC37071 (BAC37071) 13 days embryo male testis cDNA, RIKEN	Weakly similar to BAC37071 (BAC37071) 13 days embryo male testis cDNA, RIKEN / Gallus gallus finished cDNA, clone ChEST463o9 / Gallus gallus finished cDNA, clone ChEST463o9 / Finished cDNA, clone ChEST463o9 / --- / --- / --- / --- / --- / Gallus gallus finished cDNA, clone ChEST463o9 / Finished cDNA, clone ChEST463o9
RIGG02723	1.925	0.0495	CGTAAGTAACTGAATGGGACACGAAGAATTGACGTTTAAATGGCCAGCATGTTGCAAAAGTCTGACAGCA	Similar to ANK2_HUMAN (Q01484) Ankyrin 2 (Brain ankyrin) (Ankyrin B) (	Similar to ANK2_HUMAN (Q01484) Ankyrin 2 (Brain ankyrin) (Ankyrin B) (/ Gallus gallus finished cDNA, clone ChEST314a11 / Gallus gallus finished cDNA, clone ChEST380o23 / Finished cDNA, clone ChEST314a11 / similar to ankyrin B / PREDICTED: Gallus gallus similar to ankyrin 2 isoform 1; ankyrin-2, nonerythrocytic; ankyrin-B; ankyrin, brain; ankyrin, neuronal; ankyrin, nonerythroid; Long QT syndrome-4; long (electrocardiographic) QT syndrome 4 (LOC422689), mRNA. / PREDICTED: similar to ankyrin 2 isoform 1; ankyrin-2, nonerythrocytic; ankyrin-B; ankyrin, brain; ankyrin, neuronal; ankyrin, nonerythroid; Long QT syndrome-4; long (electrocardiographic) QT syndrome 4; / Transcribed locus, moderately similar to NP_001029340.1 ankyrin 2, brain isoform 3 [Mus musculus];Ankyrin 2 (chAnk2);Finished cDNA, clone ChEST314a11; / --- / Gallus gallus finished cDNA, clone ChEST380o23 / Finished cDNA, clone ChEST314a11

Gene Name	Fold Change	p-value	SEQUENCE	Array Description	Blast/Database Description
RIGG05765	1.922	0.038	TTTAGGCCGTAATTAGTTCTGTAATTGTGCATCAGAGCCAAACTCCCTCCATTGCCTTCCAGTGCATCCC	Weakly similar to CAH7_MOUSE (Q9ERQ8) Carbonic anhydrase VII (EC 4.2.1.1) (Ca	Weakly similar to CAH7_MOUSE (Q9ERQ8) Carbonic anhydrase VII (EC 4.2.1.1) (Ca / Gallus gallus finished cDNA, clone ChEST799d8 / 603811826F1 CSEQCHN74 Gallus gallus cDNA clone ChEST799d8 5', mRNA sequence / Finished cDNA, clone ChEST799d8 / similar to Carbonic anhydrase 5b, mitochondrial / PREDICTED: Gallus gallus similar to carbonic anhydrase 5b, mitochondrial; carbonic anhydrase VB (LOC415833), mRNA. / PREDICTED: similar to carbonic anhydrase 5b, mitochondrial; carbonic anhydrase VB; / --- / --- / 603811826F1 CSEQCHN74 Gallus gallus cDNA clone ChEST799d8 5', mRNA sequence / Finished cDNA, clone ChEST799d8
RIGG08852	1.921	0.0381	GTTCCGAACCATGGAGTCTAATCAAAAGACATCCTTCCAAGAAATCCCCAAACTGAATGAAGATTTGGTGC		ENSGALG00000010446.1 / --- / --- / --- / similar to Protein KIAA0494 / --- / --- / --- / KIAA0494; / --- / ---
RIGG01311	1.92	0.0172	CTGGAGGACGTCATCGAGGAGATCATCAAGTCGGAGATCCTGGACGAGTCGGATGCTTTTGCAGC	Homologue of Q9JIM7 (Q9JIM7) Ancient conserved domain protein 4	Homologue of Q9JIM7 (Q9JIM7) Ancient conserved domain protein 4 / Gallus gallus finished cDNA, clone ChEST186p12 / 603209548F1 CSEQRBN11 Gallus gallus cDNA clone ChEST186p12 5', mRNA sequence / Finished cDNA, clone ChEST186p12 / --- / --- / --- / --- / cyclin M2; / --- / ---
RIGG00167	1.918	0.0451	AGTCTTCCATGCTTCAAGTGGATTCGAAAATAACAATTAAGAGCTGTGGATTTGGTTTCTTCAGGCCTCTG	Gallus gallus mRNA for hypothetical protein, clone 9o18	Gallus gallus mRNA for hypothetical protein, clone 9o18 / RF|NP_001026325.1|71896627|NM_001031154 FIP1-like 1 {Gallus gallus} (exp=-1; wgp=0; cg=0), complete / Gallus gallus mRNA for hypothetical protein, clone 9o18 / Hypothetical protein, clone 9o18 / --- / --- / --- / Hypothetical protein, clone 9o18; / --- / Gallus gallus mRNA for hypothetical protein, clone 9o18 / Hypothetical protein, clone 9o18
RIGG00279	1.916	0.0247	GTCGAGAGGAAGGATCTTCGTTTTGTGCAACAGCTCAGTTTGGTTCTTAGGTAGGCAGAGGACAAGGTGG	Gallus gallus mRNA for hypothetical protein, clone 23a7	Gallus gallus mRNA for hypothetical protein, clone 23a7 / homologue to UP|Q86WE3_HUMAN (Q86WE3) Sarcoma antigen NY-SAR-91 (Fragment), partial (96%) / Gallus gallus mRNA for hypothetical protein, clone 23a7 / Similar to hypothetical protein FLJ11730 (LOC419617), mRNA / --- / PREDICTED: Gallus gallus similar to hypothetical protein FLJ11730 (LOC419617), mRNA. / PREDICTED: similar to hypothetical protein FLJ11730; / --- / --- / Gallus gallus mRNA for hypothetical protein, clone 23a7 / Similar to hypothetical protein FLJ11730 (LOC419617), mRNA
RIGG01985	1.914	0.00123	GTGATGCATCTGTGTCTGAGCTACACTGGGTGAGAAGTGTTTTGCTTTTCCTCTGGTCCATACCTCCCAG	Genome Hit Contig55.10	Genome Hit Contig55.10 / Gallus gallus finished cDNA, clone ChEST269e23 / Gallus gallus finished cDNA, clone ChEST269e23 / Finished cDNA, clone ChEST269e23 / --- / --- / --- / --- / --- / Gallus gallus finished cDNA, clone ChEST269e23 / Finished cDNA, clone ChEST269e23

Gene Name	Fold Change	p-value	SEQUENCE	Array Description	Blast/Database Description
RIGG11367	1.912	0.00764	AATTCTCTGCTTACTTACCAGCTCACTGAAGACTCATCCTTCTCTCTGGCGATGAAGGTGAGCAAGGGAG		ENSGALT00000004552.1 / --- / Gallus gallus gammaA-like protocadherin precursor, mRNA, complete cds / GammaA-like protocadherin precursor / similar to protocadherin gamma B5, partial / PREDICTED: Gallus gallus similar to Protocadherin beta 1 precursor (PCDH-beta1) (LOC429549), partial mRNA.PREDICTED: Gallus gallus similar to Protocadherin beta 15 precursor (LOC430703), partial mRNA.PREDICTED: Gallus gallus similar to Protocadherin beta 15 precursor (LOC431306), partial mRNA. / PREDICTED: similar to Protocadherin beta 1 precursor (PCDH-beta1), partial;PREDICTED: similar to Protocadherin beta 15 precursor, partial;PREDICTED: similar to Protocadherin beta 15 precursor, partial; / --- / --- / --- / ---
RIGG15618	1.91	0.0434	AGAGAACTTTGTTTCCTTCCATTTCTAACTTATTCGTTCGTTGTACTGTTGTGTAGCACTAAAACACCCGCG	Acylphosphatase organ-common type isozyme (EC 3.6.1.7) (Acylphosphate phosphohydrolase) (Isozyme CH2). [Source:SWISSPROT;Acc:P07032] organ-common type isozyme (EC 3.6.1.7) (Acylphosphate phosphohydrolase) (Isozyme CH2). [Source:SWISSPROT;Acc:P07032]	Acylphosphatase organ-common type isozyme (EC 3.6.1.7) (Acylphosphate phosphohydrolase) (Isozyme CH2). [Source:SWISSPROT / UP|ACYP1_CHICK (P07032) Acylphosphatase, organ-common type isozyme (Acylphosphate phosphohydrolase) (Isozyme CH2) , complete / --- / --- / ACYP1: Acylphosphatase-1 / PREDICTED: Gallus gallus similar to Acylphosphatase, organ-common type isozyme (Acylphosphate phosphohydrolase) (Isozyme CH2) (LOC423361), mRNA. / PREDICTED: similar to Acylphosphatase, organ-common type isozyme (Acylphosphate phosphohydrolase) (Isozyme CH2); / --- / --- / --- / ---
RIGG01170	1.908	0.0227	GGAACACGCTGTGCTTTAGCTTATCTTTCTTTTCACACACATGCTCCTTGATTATTCTCTTGTCCATGAG	Weakly similar to Q9N0Y9 (Q9N0Y9) Ubiquitous tropomodulin U-Tmod	Weakly similar to Q9N0Y9 (Q9N0Y9) Ubiquitous tropomodulin U-Tmod / Gallus gallus finished cDNA, clone ChEST161f8 / Gallus gallus finished cDNA, clone ChEST161f8 / Finished cDNA, clone ChEST63d8 / --- / --- / --- / --- / leiomodin 3 (fetal); / Gallus gallus finished cDNA, clone ChEST161f8 / Finished cDNA, clone ChEST63d8
RIGG00296	1.907	0.0077	TCACAGAAAGAAAACAGAAAATGGTCAGCATTCCTCTGAGAAAACTAGCCATAGAGGTGGATATCCCCAT	Gallus gallus mRNA for hypothetical protein, clone 24f18	Gallus gallus mRNA for hypothetical protein, clone 24f18 / weakly similar to UP|LAS1L_HUMAN (Q9Y4W2) LAS1-like protein, partial (23%) / 603120588F1 CSEQCHL22 Gallus gallus cDNA clone ChEST81p22 5', mRNA sequence / Hypothetical protein, clone 24f18 / --- / --- / --- / --- / --- / 603120588F1 CSEQCHL22 Gallus gallus cDNA clone ChEST81p22 5', mRNA sequence / Hypothetical protein, clone 24f18
RIGG03719	1.905	0.00824	TTGTGCTGCTGGAGTTTGTGACCAAGGCACGGATGCTGTATGATGTCCTGGATAGCGGTGCCGGT	Similar to Q96GE2 (Q96GE2) Hypothetical protein (Fragment)	Similar to Q96GE2 (Q96GE2) Hypothetical protein (Fragment) / Gallus gallus finished cDNA, clone ChEST538d11 / 603007719F1 CSEQCHL23 Gallus gallus cDNA clone ChEST28e5 5', mRNA sequence / Finished cDNA, clone ChEST538d11 / --- / --- / --- / --- / --- / 603007719F1 CSEQCHL23 Gallus gallus cDNA clone ChEST28e5 5', mRNA sequence / Finished cDNA, clone ChEST538d11
RIGG17447	1.898	0.0418	GACCCTTTTCTCTGAAAGACGCAGAACGAGAAGATAGTCACTTACAATTAACTCCATCAATGTCTCCTCCT		ENSGALT00000022041.1 / --- / --- / --- / --- / --- / --- / --- / --- / --- / ---

Gene Name	Fold Change	p-value	SEQUENCE	Array Description	Blast/Database Description
RIGG02106	1.897	0.00205	CATGTTGAATCAACTGTCCCAGTTAAACCAGCTTTCTCAGATCTCCCAGTTACAGCGGTTGTTGGCTCAG	Similar to Q8NDV7 (Q8NDV7) EDIE protein	Similar to Q8NDV7 (Q8NDV7) EDIE protein / --- / Gallus gallus finished cDNA, clone ChEST287g16 / Finished cDNA, clone ChEST287g16 / similar to EDIE protein / --- / --- / Finished cDNA, clone ChEST287g16; / trinucleotide repeat containing 6A; / Gallus gallus finished cDNA, clone ChEST287g16 / Finished cDNA, clone ChEST287g16
RIGG06496	1.896	0.0295	GTTAAACACCATCACCAGGAAAAGCTGCTTCTTTGCACATTTTCCAAGTTAGCATTCTAGACCAACTGTG	Genome Hit Contig10.37	Genome Hit Contig10.37 / weakly similar to UP|Q68HK6_PICAN (Q68HK6) Peroxin 2, partial (7%) / Gallus gallus finished cDNA, clone ChEST904o3 / Finished cDNA, clone ChEST904o3 / --- / --- / --- / --- / --- / Gallus gallus finished cDNA, clone ChEST904o3 / Finished cDNA, clone ChEST904o3
RIGG03284	1.896	0.00268	ACATTTGCAGGTGTCAGAAGCAGCCACCAGCCAACTGACATGTCAACAATAGAGGAAATGACTATTTTGTAAA	Contig Hit 355039.2	Contig Hit 355039.2 / Gallus gallus finished cDNA, clone ChEST473p4 / Gallus gallus mRNA for hypothetical protein, clone 29l7 / Heterogeneous nuclear ribonucleoprotein D-like (HNRPDL), mRNA / --- / --- / --- / --- / --- / --- / ---
RIGG06226	1.89	0.0013	CCCACCTCAGTCTTGCTACAATGGCACTGAACTTTCACAGGTCCACAACTTTTGATTTTAAAGGTCTCTAA	Contig Hit 051023.1	Contig Hit 051023.1 / homologue to GB|AAF14858.1|6523797|AF110775 adrenal gland protein AD-002 {Homo sapiens} (exp=-1; wgp=0; cg=0), complete / Gallus gallus finished cDNA, clone ChEST864b4 / Finished cDNA, clone ChEST864b4 / --- / --- / --- / --- / --- / Gallus gallus finished cDNA, clone ChEST864b4 / Finished cDNA, clone ChEST864b4
RIGG03256	1.886	0.0228	TCCCTTGTTCTCATTGTTTTACATGGAGACTTTCGGGTCACCCTAATAAGGACATGCAGTTAAATAACCT	Genome Hit Contig3.1036	Genome Hit Contig3.1036 / --- / 603524766F1 CSEQCHN68 Gallus gallus cDNA clone ChEST469d18 5', mRNA sequence / Denervation associated mRNA sequence / --- / --- / --- / --- / --- / 603524766F1 CSEQCHN68 Gallus gallus cDNA clone ChEST469d18 5', mRNA sequence / Denervation associated mRNA sequence
RIGG20256	1.885	0.0215	GGAGTCACAGTAGCGGTAAGAGCGGTGTGAGTGTCCCAAGAACTTCCTTCAATGTTTCAGTGTACAAGAT	Gallus gallus similar to adenosine kinase isoform a; adenosine 5-phosphotransferase (LOC423735), mRNA	Gallus gallus similar to adenosine kinase isoform a; adenosine 5-phosphotransferase (LOC423735), mRNA / RF|NP_001006501.1|57529848|NM_001006501 adenosine kinase {Gallus gallus} (exp=-1; wgp=0; cg=0), complete / Gallus gallus mRNA for hypothetical protein, clone 1l21 / Adenosine kinase (ADK), mRNA / RCJMB04_1l21: Hypothetical protein / PREDICTED: Gallus gallus similar to adenosine kinase isoform a; adenosine 5-phosphotransferase (LOC423735), mRNA. / PREDICTED: similar to adenosine kinase isoform a; adenosine 5-phosphotransferase; / Adenosine kinase (ADK), mRNA; / --- / Gallus gallus mRNA for hypothetical protein, clone 1l21 / Adenosine kinase (ADK), mRNA
RIGG03345	1.883	0.0222	CCCATTGTGCGTGGTAGTACGTGTATGTTCCTGTATTCTTCTTTTCCCTGTGAAATAGTGCACGGTGGTG	Weakly similar to Q8NAV0 (Q8NAV0) Hypothetical protein FLJ34737	Weakly similar to Q8NAV0 (Q8NAV0) Hypothetical protein FLJ34737 / homologue to UP|SCAM5_HUMAN (Q8TAC9) Secretory carrier-associated membrane protein 5 (Secretory carrier membrane protein 5), partial (55%) / Gallus gallus finished cDNA, clone ChEST650c20 / Finished cDNA, clone ChEST650c20 / similar to LOC508510 protein / --- / --- / --- / --- / Gallus gallus finished cDNA, clone ChEST650c20 / Finished cDNA, clone ChEST650c20

Gene Name	Fold Change	p-value	SEQUENCE	Array Description	Blast/Database Description
RIGG06633	1.882	0.0454	AGAGACTGCAGATACCATTGCTAAAGAAGCCAAGAAAGCTACAGTGAACTTGCTGGGTGAGGAGAAGAAG	Similar to Q921E4 (Q921E4) Deleted in polyposis 1	Similar to Q921E4 (Q921E4) Deleted in polyposis 1 / Gallus gallus finished cDNA, clone ChEST919o23 / gonad_EST09156 Embryonic gonad cDNA Library Gallus gallus cDNA 5', mRNA sequence / Finished cDNA, clone ChEST919o23 / --- / --- / --- / --- / receptor accessory protein 5; / gonad_EST09156 Embryonic gonad cDNA Library Gallus gallus cDNA 5', mRNA sequence / Finished cDNA, clone ChEST919o23
RIGG05566	1.878	0.0447	CACTGAGTTGCCTCACACTGAGCTTACAGGTCTTTCTGGTCAAGCTCCCTATACACTTAACTTCAGCTTTGA	Similar to Q9ERD4 (Q9ERD4) Ankyrin repeat-rich membrane-spanning prote	Similar to Q9ERD4 (Q9ERD4) Ankyrin repeat-rich membrane-spanning prote / Gallus gallus finished cDNA, clone ChEST75f10 / Gallus gallus finished cDNA, clone ChEST76l18 / Finished cDNA, clone ChEST76l18 / --- / --- / --- / --- / --- / Gallus gallus finished cDNA, clone ChEST76l18 / Finished cDNA, clone ChEST76l18
RIGG08531	1.876	0.0175	TCATGAGAATGGCCTCACACTGCTTCACTTCGTGGTGATCCAAGGAAACGTGGGAAAGGTGAAGTTTCTC		ENSGALG00000007815.1 / --- / Gallus gallus mRNA for hypothetical protein, clone 2g14 / Ankyrin repeat domain 44 (ANKRD44), mRNA / similar to X-kinase / --- / --- / --- / --- / --- / ---
RIGG11235	1.876	0.0249	AGAATATTTGATAAAGTGGTTTCTGTTAGTCTTGGCCCAAAGAAAACGTCTTAGCTCTTTGCTAGAACCGGG		ENSGALT00000004189.1 / RF|NP_001006198.1|57525219|NM_001006198 WD repeat domain 5 {Gallus gallus} (exp=-1; wgp=0; cg=0), complete / Gallus gallus mRNA for hypothetical protein, clone 18o16 / WD repeat domain 5 (WDR5), mRNA / RCJMB04_18o16: Hypothetical protein / PREDICTED: Gallus gallus similar to Zgc:56591 protein (LOC417144), mRNA. / PREDICTED: similar to Zgc:56591 protein; / WD repeat domain 5 (WDR5), mRNA; / --- / Gallus gallus mRNA for hypothetical protein, clone 18o16 / WD repeat domain 5 (WDR5), mRNA
RIGG09423	1.872	0.0282	GTTTGGGGTCAACACATTCATTGCCTTAGCACTTCAGACTTTACTCACGTTGATTGTTGTGGATGCCAGT		ENSGALG00000015218.1 / --- / --- / --- / --- / --- / --- / --- / solute carrier family 19 (thiamine transporter), member 2; / --- / ---
RIGG17963	1.871	0.0296	CTACTTATCTGTGCTTTGAAAGCTTTAGTGATAACATGTGGCAGAAGAACCTCTTCCCTCTGGTCATCCT		ENSGALT00000023341.1 / similar to UP|GPR92_HUMAN (Q9H1C0) Probable G-protein coupled receptor 92, partial (60%) / pha008_a11 PHA-activated splenocytes Gallus gallus cDNA, mRNA sequence / Transcribed locus, weakly similar to XP_001063300.1 PREDICTED: similar to Probable G-protein coupled receptor 92 [Rattus norvegicus] / similar to Gpr92 protein / PREDICTED: Gallus gallus similar to putative G protein-coupled receptor 92 (LOC418279), mRNA. / PREDICTED: similar to putative G protein-coupled receptor 92; / --- / --- / --- / ---
RIGG05918	1.869	0.0171	TGTTGGTTCTCTGAGTGACGTCTTGATTTCAATAGCTAGAGTTTTCCCTGAATCTACTAGAAGGGGTATCAGT	Genome Hit Contig34.110	Genome Hit Contig34.110 / homologue to UP|Q95CJ4_9ERIC (Q95CJ4) NADH dehydrogenase subunit F (Fragment), partial (6%) / Gallus gallus finished cDNA, clone ChEST819i4 / Finished cDNA, clone ChEST819i4 / --- / --- / --- / --- / --- / Gallus gallus finished cDNA, clone ChEST819i4 / Finished cDNA, clone ChEST819i4

Gene Name	Fold Change	p-value	SEQUENCE	Array Description	Blast/Database Description
RIGG03396	1.858	0.0473	TTTGGTTCGATGTTAACGCGATGAC TACAGTGAGATGTTGGGGTTACATA GGTCTCTCATCTCCTGATGG	Genome Hit Contig229.3	Genome Hit Contig229.3 / Gallus gallus finished cDNA, clone ChEST491n23 / Contig2589 WL/RJ Phraped ESTs Gallus gallus cDNA 5', mRNA sequence / Finished cDNA, clone ChEST581l11 / --- / --- / --- / --- / --- / Contig2589 WL/RJ Phraped ESTs Gallus gallus cDNA 5', mRNA sequence / Finished cDNA, clone ChEST581l11
RIGG08945	1.857	0.0303	CGACGTACAGAGTTCTGACTCCACT ACGGTTTTCCACAGTAGGGAATGTT CAGCTAAACATACAGGTCCT		ENSGALG00000011115.1 / --- / --- / --- / similar to WD repeat domain 78 / --- / --- / --- / WD repeat domain 78; / --- / ---
RIGG03424	1.851	0.0378	CTGCAGAGGCTGCTAAACACAAACC CCTTATTTAAAGAGGCCTTGAAACA GATTGAAAGGAAACCAGAAT	Similar to Q8VCE8 (Q8VCE8) Hypothetical 53.1 kDa protein (Fragment)	Similar to Q8VCE8 (Q8VCE8) Hypothetical 53.1 kDa protein (Fragment) / Gallus gallus finished cDNA, clone ChEST495b14 / Gallus gallus mRNA for hypothetical protein, clone 13f18 / Neuronal guanine nucleotide exchange factor (NGEF), mRNA / similar to putative SH3 domain-containing guanine exchange factor SGEF / --- / Src homology 3 domain-containing guanine nucleotide exchange factor; / --- / --- / --- / ---
RIGG18541	1.851	0.0166	ACTCTAGTGCAGAATTAGATCTGAC AAAACCTGAAACATTCCGTGACCTT AGTAAACCAATTGGTGCCCT		ENSGALT00000024908.1 / Gallus gallus finished cDNA, clone ChEST459k17 / 603520227F1 CSEQCHN67 Gallus gallus cDNA clone ChEST462e8 5', mRNA sequence / Finished cDNA, clone ChEST459k17 / --- / PREDICTED: Gallus gallus similar to Protein FAN (Factor associated with N-SMase activation) (Factor associated with neutral-sphingomyelinase activation) (LOC426261), partial mRNA.PREDICTED: Gallus gallus similar to Protein FAN (Factor associated with N-SMase activation) (Factor associated with neutral-sphingomyelinase activation) (LOC431430), partial mRNA. / PREDICTED: similar to Protein FAN (Factor associated with N-SMase activation) (Factor associated with neutral-sphingomyelinase activation), partial;PREDICTED: similar to Protein FAN (Factor associated with N-SMase activation) (Factor associated with neutral-sphingomyelinase activation), partial; / --- / --- / 603520227F1 CSEQCHN67 Gallus gallus cDNA clone ChEST462e8 5', mRNA sequence / Finished cDNA, clone ChEST459k17
RIGG04361	1.849	0.0358	ACCTTTCCTTCTTCGGTGTGGACAT TAACTTCTATTGGGACATAAATGCCA TCTTCAACTCCCTCTTGCC	Weakly similar to CG24_MOUSE (Q9D7X8) Protein C7orf24 homolog	Weakly similar to CG24_MOUSE (Q9D7X8) Protein C7orf24 homolog / Gallus gallus finished cDNA, clone ChEST620h14 / Gallus gallus finished cDNA, clone ChEST620h14 / Finished cDNA, clone ChEST792d1 / --- / --- / --- / --- / --- / Gallus gallus finished cDNA, clone ChEST620h14 / Finished cDNA, clone ChEST792d1

Gene Name	Fold Change	p-value	SEQUENCE	Array Description	Blast/Database Description
RIGG05635	1.849	0.0266	AAATCCAATCTCATGCCGTCTTCGTTCTTTAAGTAGTCTTTGCAGTCTCAGAGGATCTCGGCTCGCAGGG	Same gene D42149; Chicken mRNA for cadherin-6B, complete cds	Same gene D42149; Chicken mRNA for cadherin-6B, complete cds / Gallus gallus finished cDNA, clone ChEST783i5 / Gallus gallus cadherin 6, type 2, K-cadherin (fetal kidney) (CDH6), mRNA / Cadherin-6B / CDH6: Cadherin-6 precursor / PREDICTED: Gallus gallus cadherin 6B (CDH6), mRNA.PREDICTED: Gallus gallus similar to chicken cadherin-6B (LOC431392), partial mRNA. / PREDICTED: cadherin 6B;PREDICTED: similar to chicken cadherin-6B, partial; / Cadherin-6B; / --- / Gallus gallus cadherin 6, type 2, K-cadherin (fetal kidney) (CDH6), mRNA / Cadherin-6B
RIGG13321	1.848	0.0337	CCAGCCCCTTTCTACCCTTCCTCTTCTCCAAAACCTTAACTCATTGTGAAGCAGAACATCCCTTATTTTA	histone macroH2A1.2 [Gallus gallus]. [Source:RefSeq;Acc:NM_205007]	histone macroH2A1.2 [Gallus gallus]. [Source:RefSeq / Gallus gallus finished cDNA, clone ChEST62o10 / Gallus gallus histone macroH2A1.2 (LOC395858), mRNA / Truncated histone macroH2A1 / H2AFY: Core histone macro-H2A.1 / Gallus gallus histone macroH2A1.2 (LOC395858), mRNA. / histone macroH2A1.2; / Truncated histone macroH2A1; / --- / Gallus gallus histone macroH2A1.2 (LOC395858), mRNA / Truncated histone macroH2A1
RIGG04150	1.847	0.035	TCATATTGCTGGTTGTTCCTGTTCTTCGGATCTCCGTGACACATCCTGAGCATTAAAATTATCCACCCCT	Genome Hit Contig925.3	Genome Hit Contig925.3 / Gallus gallus finished cDNA, clone ChEST446g5 / Gallus gallus finished cDNA, clone ChEST53p13 / Finished cDNA, clone ChEST446g5 / --- / --- / --- / --- / --- / Gallus gallus finished cDNA, clone ChEST53p13 / Finished cDNA, clone ChEST446g5
RIGG02767	1.846	0.0289	TAAACAATGAAAGCAGTTGTGTTAAATGAACGCGAGGATTTAGCTACGTGAGACCTGACCCAGAAGAAAG	Genome Hit Contig109.2	Genome Hit Contig109.2 / Gallus gallus finished cDNA, clone ChEST386e7 / 603488127F1 CSEQCHN62 Gallus gallus cDNA clone ChEST386e7 5', mRNA sequence / Finished cDNA, clone ChEST386e7 / --- / --- / --- / --- / --- / --- / ---
RIGG01727	1.842	0.0351	TCAGGAGGCGGAGAACTTTCTCAGCGTGATGACAACCAAGGCCACGAGCACAGATGTACCCAGGA	Weakly similar to Q9CXL7 (Q9CXL7) 3110079O15Rik protein	Weakly similar to Q9CXL7 (Q9CXL7) 3110079O15Rik protein / Gallus gallus finished cDNA, clone ChEST234b3 / --- / --- / --- / --- / hypothetical protein LOC389084; / --- / --- / --- / ---
RIGG00079	1.838	0.0132	TAAGCCCTCTTCAGCATTTTGCACTCCACTGACCTCCACTGTTGGTTTTAAACACCACTAAACTAATGTT	Gallus gallus mRNA for hypothetical protein, clone 4i12	Gallus gallus mRNA for hypothetical protein, clone 4i12 / RF|NP_001025766.1|71895419|NM_001030595 Rho guanine nucleotide exchange factor 3 {Gallus gallus} (exp=-1; wgp=0; cg=0), complete / Gallus gallus mRNA for hypothetical protein, clone 4i12 / Rho guanine nucleotide exchange factor (GEF) 3 (ARHGEF3), mRNA / RCJMB04_4i12: Hypothetical protein / PREDICTED: Gallus gallus similar to Rho guanine nucleotide exchange factor 3; RhoGEF protein; 59.8 kDA protein; exchange factor found in platelets and leukemic and neuronal tissues, XPLN (LOC416000), mRNA. / PREDICTED: similar to Rho guanine nucleotide exchange factor 3; RhoGEF protein; 59.8 kDA protein; exchange factor found in platelets and leukemic and neuronal tissues, XPLN; / Rho guanine nucleotide exchange factor (GEF) 3 (ARHGEF3), mRNA; / --- / Gallus gallus mRNA for hypothetical protein, clone 4i12 / Rho guanine nucleotide exchange factor (GEF) 3 (ARHGEF3), mRNA

Gene Name	Fold Change	p-value	SEQUENCE	Array Description	Blast/Database Description
RIGG00149	1.837	0.0126	ACGCTAAATGAGTCTTCCAGGATTG TGATCTAGGATATTAAACACTTGAGA GGGCTTTACCACCATCCATTC	Gallus gallus mRNA for hypothetical protein, clone 8f5	Gallus gallus mRNA for hypothetical protein, clone 8f5 / homologue to UP|Q59H81_HUMAN (Q59H81) BRCA1 associated protein variant (Fragment), partial (80%) / Gallus gallus mRNA for hypothetical protein, clone 8f5 / Hypothetical protein, clone 8f5 / / --- / --- / Hypothetical protein, clone 8f5; / --- / Gallus gallus mRNA for hypothetical protein, clone 8f5 / Hypothetical protein, clone 8f5
RIGG02599	1.835	0.0132	GTTGTACTTGGGGATCTGAAAGGTC TTTGCAACCAGAATGATTCCACAATT GTATGACTTGGGGCAAAGG	Weakly similar to Q9UMY3 (Q9UMY3) 3-phosphoglycerate dehydrogenase (Fragment)	Weakly similar to Q9UMY3 (Q9UMY3) 3-phosphoglycerate dehydrogenase (Fragment) / Gallus gallus finished cDNA, clone ChEST368p18 / --- / --- / / PREDICTED: Gallus gallus similar to 3-phosphoglycerate dehydrogenase (LOC424381), mRNA. / PREDICTED: similar to 3-phosphoglycerate dehydrogenase; / --- / --- / --- / ---
RIGG01509	1.833	0.0315	CCATTTTGTTCACAGGTTGTCAAAG GGGAAAAGAATTCGAACTTGTCTGT TACCCCATTGCATAAACAAG	Weakly similar to O35413 (O35413) SH3-containing protein p4015	Weakly similar to O35413 (O35413) SH3-containing protein p4015 / Gallus gallus finished cDNA, clone ChEST206j8 / Gallus gallus finished cDNA, clone ChEST206j8 / Finished cDNA, clone ChEST206j8 / --- / --- / --- / --- / --- / Gallus gallus finished cDNA, clone ChEST206j8 / Finished cDNA, clone ChEST206j8
RIGG20026	1.833	0.0493	GCCAGAGTCTGTCAGTGATCAGTGT GTTCAGTTTGTGGAACAGTATGAAC CTGTGGTTGTGCAACTCTTG	Proactivator polypeptide precursor [Contains: Saposin A; Saposin B; Saposin C; Saposin D]. [Source:SWISSPROT;Acc:O13035]	Proactivator polypeptide precursor [Contains: Saposin A / RF|NP_990142.1|45382219|NM_204811 prosaposin {Gallus gallus} (exp=-1; wgp=0; cg=0), complete / Gallus gallus prosaposin (variant Gaucher disease and variant metachromatic leukodystrophy) (PSAP), mRNA / Prosaposin / PSAP: Proactivator polypeptide precursor [Contains: Saposin A; Saposin B; Saposin C; Saposin D] / Gallus gallus prosaposin (LOC395602), mRNA. / --- / Prosaposin; / --- / Gallus gallus prosaposin (variant Gaucher disease and variant metachromatic leukodystrophy) (PSAP), mRNA / Prosaposin
RIGG11755	1.83	0.0348	GGCAGCCCTCTTTGTGGTGTTTCTG CTCATCTACGTCATCACCCTTTCAG GCAATTTGGGGATAATCATC	Olfactory receptor 4 (Fragment). [Source:SPTREMBL;Acc:Q90808]	Olfactory receptor 4 (Fragment). [Source:SPTREMBL / UP|Q90808_CHICK (Q90808) Olfactory receptor 4 (Fragment), complete / Gallus gallus olfactory receptor 4 (cor4), mRNA / Olfactory receptor 4 (cor4), mRNA / cor4: Olfactory receptor 4 (Fragment) / PREDICTED: Gallus gallus similar to olfactory receptor 4 (LOC428818), mRNA.PREDICTED: Gallus gallus similar to olfactory receptor 4 (LOC428819), mRNA. / PREDICTED: similar to olfactory receptor 4;PREDICTED: similar to olfactory receptor 4; / Olfactory receptor 4 (cor4), mRNA; / --- / Gallus gallus olfactory receptor 4 (cor4), mRNA / Olfactory receptor 4 (cor4), mRNA

Gene Name	Fold Change	p-value	SEQUENCE	Array Description	Blast/Database Description
RIGG00669	1.828	0.0194	CACTTCTTCCTGGTCAATGTGTTCG TGATGGGAATGCTCAAAACCTTTGG CATTTTCTTCGTGGCATTCC	Similar to MOT5_HUMAN (O15374) Monocarboxylate transporter 5 (MCT 5) (	Similar to MOT5_HUMAN (O15374) Monocarboxylate transporter 5 (MCT 5) (/ Gallus gallus finished cDNA, clone ChEST1015m15 / gPGC_EST08538 Embryonic gonadal PGC cDNA Library Gallus gallus cDNA 5', mRNA sequence / Finished cDNA, clone ChEST48n23 / --- / PREDICTED: Gallus gallus similar to Solute carrier family 16 (monocarboxylic acid transporters), member 4 (LOC419809), mRNA. / PREDICTED: similar to Solute carrier family 16 (monocarboxylic acid transporters), member 4; / --- / --- / gPGC_EST08538 Embryonic gonadal PGC cDNA Library Gallus gallus cDNA 5', mRNA sequence / Finished cDNA, clone ChEST48n23
RIGG02434	1.827	0.0322	GGTTCAGTCCTGCTAGGTGAAGAAG GGGAATTGTCATTCTTCTAGGTTACT TATAAATAAACTGATGTGGAGCCT	Genome Hit Contig70.98	Genome Hit Contig70.98 / Gallus gallus finished cDNA, clone ChEST342b8 / 603500630F1 CSEQCHN65 Gallus gallus cDNA clone ChEST417k8 5', mRNA sequence / Finished cDNA, clone ChEST342b8 / --- / --- / --- / --- / --- / --- / ---
RIGG05834	1.826	0.0231	CAGTCCCAGCCACTTCACGTAAGGT ACCTTAGTCTGAGAAATCAAATCTC CAGCTGCTTTCTAAACCTGC	Weakly similar to BAC86241 (BAC86241) CDNA FLJ43687 fis, clone TBAES2002197	Weakly similar to BAC86241 (BAC86241) CDNA FLJ43687 fis, clone TBAES2002197 / Gallus gallus finished cDNA, clone ChEST808i15 / Gallus gallus finished cDNA, clone ChEST808i15 / Finished cDNA, clone ChEST808i15 / --- / --- / --- / --- / --- / Gallus gallus finished cDNA, clone ChEST808i15 / Finished cDNA, clone ChEST808i15
RIGG04337	1.826	0.0357	CCATGTCTCACAACAGCAGCTATAA TTAATGACAACTGAAATATTAAACAC AGCACCTAGTTCAGGGCGCT	Genome Hit Contig9.122	Genome Hit Contig9.122 / Gallus gallus finished cDNA, clone ChEST618b5 / Gallus gallus finished cDNA, clone ChEST618b5 / Finished cDNA, clone ChEST618b5 / --- / --- / --- / --- / --- / Gallus gallus finished cDNA, clone ChEST618b5 / Finished cDNA, clone ChEST618b5
RIGG16448	1.825	0.00733	CTGTACTCTTGTCCACAGCACTGAC GTTCAGGATACCATTAGCATCTATG TCAAAAGTGACCTCGATCTG		ENSGALT00000019143.1 / UP|Q7SX63_CHICK (Q7SX63) Heat shock protein 70 (Heat shock protein Hsp70), complete / Gallus gallus heat shock protein 70 (HSP70), mRNA / Heat shock protein 70 (HSP70), mRNA / Heat shock 70 kDa protein / PREDICTED: Gallus gallus similar to heat shock protein 70 (LOC423504), mRNA. / PREDICTED: similar to heat shock protein 70; / Heat shock protein 70 (HSP70), mRNA; / --- / Gallus gallus heat shock protein 70 (HSP70), mRNA / Heat shock protein 70 (HSP70), mRNA
RIGG06668	1.824	0.0324	TTCTGCCATCCCTAAATCAAAGTGA AAATCCTTCGGGTGAATGAGCAGGT CACAGAGGGAAACCACAGTC	Genome Hit Contig11.306	Genome Hit Contig11.306 / Gallus gallus finished cDNA, clone ChEST925n6 / 603956723F1 CSEQRBN09 Gallus gallus cDNA clone ChEST925n6 5', mRNA sequence / Finished cDNA, clone ChEST925n6 / --- / --- / --- / --- / --- / 603956723F1 CSEQRBN09 Gallus gallus cDNA clone ChEST925n6 5', mRNA sequence / Finished cDNA, clone ChEST925n6
RIGG19272	1.821	0.0139	AATCAGAAATAACACAGTGATTGGC CTTTCTGCAACGGATGGTTTATCCA ACATTGAGACTCTGTCACCA		ENSGALT00000026915.1 / --- / --- / --- / --- / --- / --- / --- / --- / 603811813F1 CSEQCHN74 Gallus gallus cDNA clone ChEST799j4 5', mRNA sequence / Finished cDNA, clone ChEST799j4

Gene Name	Fold Change	p-value	SEQUENCE	Array Description	Blast/Database Description
RIGG00160	1.811	0.0441	GGAGAACGCTGCAGAAAACATTTAA GGGGTCAATATTTGCGGTTTTGATA GTGGTTGAATCTGGCCAAGA	Gallus gallus mRNA for hypothetical protein, clone 9h4	Gallus gallus mRNA for hypothetical protein, clone 9h4 / homologue to UP|Q8QHI5_CHICK (Q8QHI5) Untranslated region binding-protein, partial (82%) / Gallus gallus finished cDNA, clone ChEST63o22 / Untranslated region binding-protein (UBP) / UBP: Untranslated region binding-protein / Gallus gallus untranslated region binding-protein (UBP), mRNA. / --- / --- / --- / --- / ---
RIGG12179	1.808	0.0439	TTTACATTGCCAGTACCTTGGTCAG CTAAAATAATTCTAACCATGGTTCCA GTTTAACAATGAAGCCAGCGC		ENSGALT00000006899.1 / Gallus gallus finished cDNA, clone ChEST365h9 / 603478436F1 CSEQCHN71 Gallus gallus cDNA clone ChEST365h9 5', mRNA sequence / Finished cDNA, clone ChEST365h9 / --- / --- / --- / --- / --- / 603478436F1 CSEQCHN71 Gallus gallus cDNA clone ChEST365h9 5', mRNA sequence / Finished cDNA, clone ChEST365h9
RIGG15648	1.807	0.0158	CTCAAATGTTCTTCACGGTGTCTTG GATGTTTCCTCCGACTGGCGTCTCA ATGACCGTCATTTCTCACCG		ENSGALT00000016866.1 / Gallus gallus finished cDNA, clone ChEST43i5 / Gallus gallus finished cDNA, clone ChEST43i5 / Finished cDNA, clone ChEST43i5 / --- / PREDICTED: Gallus gallus similar to MUF1 protein; likely ortholog of mouse MUF1; elongin BC-interacting leucine-rich repeat protein (LOC424612), mRNA. / PREDICTED: similar to MUF1 protein; likely ortholog of mouse MUF1; elongin BC-interacting leucine-rich repeat protein; / Finished cDNA, clone ChEST43i5; / --- / Gallus gallus finished cDNA, clone ChEST43i5 / Finished cDNA, clone ChEST43i5
RIGG01600	1.804	0.0446	TCACGTTCTCTTATGAAGTACAAAAG TTTATCTGCTGAATCATCTCACACCA GCTCGGACATCTCACAGAA	Weakly similar to BAC87247 (BAC87247) CDNA FLJ46164 fis, clone TESTI4002799,	Weakly similar to BAC87247 (BAC87247) CDNA FLJ46164 fis, clone TESTI4002799, / Gallus gallus finished cDNA, clone ChEST215f20 / 603221175F1 CSEQRBN10 Gallus gallus cDNA clone ChEST215f20 5', mRNA sequence / Finished cDNA, clone ChEST215f20 / --- / --- / --- / --- / polymerase (RNA) I polypeptide B, 128kDa; / 603221175F1 CSEQRBN10 Gallus gallus cDNA clone ChEST215f20 5', mRNA sequence / Finished cDNA, clone ChEST215f20
RIGG03969	1.801	0.0258	AGAGCAAATTACTCCTTGAATGTGT ATTTAATGGCTTGTTTAATGGGAAG GACTTGTGCATTGGGGTCCA	Genome Hit Contig40.207	Genome Hit Contig40.207 / similar to UP|Q94XF1_9SAUR (Q94XF1) NADH dehydrogenase subunit II (Fragment), partial (5%) / Gallus gallus finished cDNA, clone ChEST567f19 / Finished cDNA, clone ChEST567f19 / --- / --- / --- / --- / --- / Gallus gallus finished cDNA, clone ChEST567f19 / Finished cDNA, clone ChEST567f19
RIGG19869	0.555	0.0304	CCTTAAAGGAGACATGTTTATTGTC CATAATGAATTGGAAGATGGCTGGA TGTGGGTTACAAACCTACGGAC		ENSGALT00000028411.1 / --- / --- / --- / similar to GTPase-activating protein / PREDICTED: Gallus gallus similar to Ras GTPase-activating protein 1 (GTPase-activating protein) (GAP) (Ras p21 protein activator) (p120GAP) (RasGAP) (LOC425409), partial mRNA. / PREDICTED: similar to Ras GTPase-activating protein 1 (GTPase-activating protein) (GAP) (Ras p21 protein activator) (p120GAP) (RasGAP), partial; / --- / --- / testis_EST04890 Testis cDNA Library Gallus gallus cDNA 3', mRNA sequence / Finished cDNA, clone ChEST231m8

Gene Name	Fold Change	p-value	SEQUENCE	Array Description	Blast/Database Description
RIGG11221	0.555	0.0408	GGGGCTGGGCAGAAATGAGATAAC ACGGGGATTGATCTGTCCAAAGGTT AAGGAGCTGAACTTATTTAACT		ENSGALT00000004151.1 / Gallus gallus finished cDNA, clone ChEST202d21 / 603216082F1 CSEQRBN14 Gallus gallus cDNA clone ChEST202d21 5', mRNA sequence / Finished cDNA, clone ChEST202d21 / --- / --- / --- / --- / --- / 603216082F1 CSEQRBN14 Gallus gallus cDNA clone ChEST202d21 5', mRNA sequence / Finished cDNA, clone ChEST202d21
RIGG18328	0.554	0.0345	TCCCTCGCTTTCTTGAGATGTCTTCA GTTTCTGTATTATCTTACACGTGTTC TTGGCTGCATTTTGCTGC		ENSGALT00000024334.1 / Gallus gallus finished cDNA, clone ChEST920b1 / Gallus gallus finished cDNA, clone ChEST920b1 / Finished cDNA, clone ChEST830a20 / --- / --- / --- / --- / --- / Gallus gallus finished cDNA, clone ChEST920b1 / Finished cDNA, clone ChEST830a20
RIGG10908	0.553	0.0254	CAGCCTGTTCAAGAAGGGACGGAG GAAAACGGTTGTCAGGAACCTGGG GAAAATCATCTATTACTCCAAG		ENSGALT00000003315.1 / --- / --- / --- / --- / --- / --- / --- / pleckstrin homology domain containing, family N member 1; / --- / ---
RIGG17894	0.552	0.0305	CCCAGAAATACAGACGCACTGCCCA AAATGCACAGAAATGGCGCCAAATA GACTCACAACACACAGAAAC		ENSGALT00000023140.1 / similar to UP|Q411A7_KINRA (Q411A7) Malate:quinone-oxidoreductase , partial (3%) / 603105564F1 CSEQCHN04 Gallus gallus cDNA clone ChEST44o15 5', mRNA sequence / Transcribed locus, strongly similar to XP_428472.1 PREDICTED: similar to guanylate cyclase-activating protein 1 [Gallus gallus] / --- / --- / --- / --- / --- / --- / ---
RIGG00602	0.551	0.0347	ACTCTTTAGGTGTGGCATACTGCAG TTGTTATACTGTACTACTGTAGCAGC TCCTTTGGAAACATAACTCTT	Similar to Y297_HUMAN (O15040) Hypothetical protein KIAA0297/KIAA0329	Similar to Y297_HUMAN (O15040) Hypothetical protein KIAA0297/KIAA0329 / Gallus gallus finished cDNA, clone ChEST1010c2 / gPGC_EST01593 Embryonic gonadal PGC cDNA Library Gallus gallus cDNA 5', mRNA sequence / Finished cDNA, clone ChEST930g3 / --- / --- / --- / --- / KIAA0329; / --- / ---
RIGG09358	0.551	0.0124	TTCCAGGCCATGAGCAGCGTTGAAA AGGAAACCAAAACTCAGTGTGTTCG AATAGCTACCAAAGCAGCAG		ENSGALG00000014783.1 / --- / Gallus gallus receptor tyrosine phosphatase mRNA, complete cds / Phosphoprotein phosphatase / / --- / --- / --- / protein tyrosine phosphatase, receptor type, K; / --- / ---

Gene Name	Fold Change	p-value	SEQUENCE	Array Description	Blast/Database Description
RIGG19319	0.55	0.047	GACAAACGCTTAGAGGATGGTTACTTCAGAATAGGGAAGTTTGAAAACGGTGTAGCTGAAGGAACGGTTG		ENSGALT00000027052.1 / Gallus gallus finished cDNA, clone ChEST31i22 / Gallus gallus mRNA for hypothetical protein, clone 3o8 / Similar to mannosyl (alpha-1,3-)-glycoprotein beta-1,4-N-acetylglucosaminyltransferase, isoenzyme A; UDP-N-acetylglucosamine:alpha1,3-d-mannoside beta1,4-N-acetylglucosaminyltransferase; alpha-1,3-mannosyl-glycoprotein beta-1,4-N-acetyl ... / RCJMB04_3o8: Hypothetical protein / PREDICTED: Gallus gallus similar to mannosyl (alpha-1,3-)-glycoprotein beta-1,4-N-acetylglucosaminyltransferase, isoenzyme A; UDP-N-acetylglucosamine:alpha1,3-d-mannoside beta1,4-N-acetylglucosaminyltransferase; alpha-1,3-mannosyl-glycoprotein beta-1,4-N-PREDICTED: Gallus gallus similar to mannosyl (alpha-1,3-)-glycoprotein beta-1,4-N-acetylglucosaminyltransferase, isoenzyme A; UDP-N-acetylglucosamine:alpha1,3-d-mannoside beta1,4-N-acetylglucosaminyltransferase; alpha-1,3-mannosyl-glycoprotein beta-1,4-N- / PREDICTED: similar to mannosyl (alpha-1,3-)-glycoprotein beta-1,4-N-acetylglucosaminyltransferase, isoenzyme A; UDP-N-acetylglucosamine:alpha1,3-d-mannoside beta1,4-N-acetylglucosaminyltransferase; alpha-1,3-mannosyl-glycoprotein beta-1,4-N-acetylglucosam;PREDICTED: similar to mannosyl (alpha-1,3-)-glycoprotein beta-1,4-N-acetylglucosaminyltransferase, isoenzyme A; UDP-N-
RIGG12951	0.548	0.015	AACATACATCGAGCAGAAGGACAACGTGCAGGACATGGCTGAGTTCTCCTTGACTGGCATTAAACAGGAG		ENSGALT00000009085.1 / GB|AJ833636.1|CAH55757.1 immunoglobulin-like receptor CHIR-B1 precursor [Gallus gallus] / Gallus gallus immunoglobulin-like receptor CHIR-B mRNA, complete cds / Immunoglobulin-like receptor CHIR-B / --- / PREDICTED: Gallus gallus similar to immunoglobulin-like receptor CHIR-B (LOC429196), partial mRNA.PREDICTED: Gallus gallus similar to immunoglobulin-like receptor CHIR-B (LOC429586), partial mRNA. / PREDICTED: similar to immunoglobulin-like receptor CHIR-B, partial;PREDICTED: similar to immunoglobulin-like receptor CHIR-B, partial; / --- / --- / --- / ---
RIGG08387	0.548	0.011	TCCTGTCACCCTTGGACTTCTACACCTTCGTGATTCCTTTTGAGATGGAAGGAGACACCACAGAGACACC		ENSGALG00000006566.1 / similar to UP|Q640P7_MOUSE (Q640P7) Tbccd1 protein, partial (89%) / Gallus gallus mRNA for hypothetical protein, clone 9e22 / TBCC domain containing 1 (TBCCD1), mRNA / --- / PREDICTED: Gallus gallus similar to hypothetical protein FLJ10560 (LOC424869), mRNA. / PREDICTED: similar to hypothetical protein FLJ10560; / TBCC domain containing 1 (TBCCD1), mRNA; / --- / Gallus gallus mRNA for hypothetical protein, clone 9e22 / TBCC domain containing 1 (TBCCD1), mRNA
RIGG10690	0.547	0.0293	ATGAAGAGTCAGAGTCAGAAATCCTCATCTCGGCAGACGATGAGATGGAGGAATCGGACGTGGAAGAGGA		ENSGALT00000002638.1 / --- / Gallus gallus mRNA for hypothetical protein, clone 2h7 / Similar to MGC69149 protein (LOC419112), mRNA / --- / --- / --- / --- / --- / --- / ---

Gene Name	Fold Change	p-value	SEQUENCE	Array Description	Blast/Database Description
RIGG20311	0.546	0.00347	ATGCCATTCAACCAGGATCAATCAAATATGACCTCCTGAAAACAGAGGATCTTAACGATGAGGAGAAGCT	Gallus gallus similar to L-plastin (Lymphocyte cytosolic protein 1) (LCP-1) (LC64P) (LOC418852), mRNA	Gallus gallus similar to L-plastin (Lymphocyte cytosolic protein 1) (LCP-1) (LC64P) (LOC418852), mRNA / RF|NP_001008440.1|56605886|NM_001008440 lymphocyte cytosolic protein 1 (L-plastin) {Gallus gallus} (exp=-1; wgp=0; cg=0), complete / Gallus gallus mRNA for hypothetical protein, clone 4k19 / Lymphocyte cytosolic protein 1 (L-plastin) (LCP1), mRNA / RCJMB04_4k19: Hypothetical protein / PREDICTED: Gallus gallus similar to L-plastin (Lymphocyte cytosolic protein 1) (LCP-1) (LC64P) (LOC418852), mRNA. / PREDICTED: similar to L-plastin (Lymphocyte cytosolic protein 1) (LCP-1) (LC64P); / Lymphocyte cytosolic protein 1 (L-plastin) (LCP1), mRNA; / --- / Gallus gallus mRNA for hypothetical protein, clone 4k19 / Lymphocyte cytosolic protein 1 (L-plastin) (LCP1), mRNA
RIGG17311	0.545	0.0444	GAGGACTACTCCAAAAGACTGTCAGAGATCTGGAGAGGGGCGGTGGAGACCTACAAGGCAGAGGT		ENSGALT00000021616.1 / UP|O57613_CHICK (O57613) Paranemin, complete / Gallus gallus nestin (NES), mRNA / Cytostructural gene {3'region} [chickens, embryo, mRNA Partial, 1711 nt] / Transitin / --- / --- / --- / nestin; / Gallus gallus nestin (NES), mRNA / Cytostructural gene {3'region} [chickens, embryo, mRNA Partial, 1711 nt]
RIGG10373	0.545	0.0185	CAGTGAGATGCGCTATCACCGGAAGCTGACTTGGAGGTCGAACCAGCACGACATCAGCATCTGCC		ENSGALT00000001774.1 / --- / --- / --- / --- / --- / --- / --- / sema domain, transmembrane domain (TM), and cytoplasmic domain, (semaphorin) 6B; / --- / ---
RIGG15813	0.545	0.0165	GGATGAGCTCGATCTTCTGTGGACCCGAAAGCAGAATGTGATGTACAACTTATTTGACTGGCCAACTCAAA		ENSGALT00000017286.1 / RF|NP_001026457.1|71896805|NM_001031286 origin recognition complex, subunit 1 {Gallus gallus} (exp=-1; wgp=0; cg=0), complete / Gallus gallus mRNA for hypothetical protein, clone 2i19 / Origin recognition complex, subunit 1-like (yeast) (ORC1L), mRNA / RCJMB04_2i19: Hypothetical protein / --- / --- / Origin recognition complex, subunit 1-like (yeast) (ORC1L), mRNA; / --- / Gallus gallus mRNA for hypothetical protein, clone 2i19 / Origin recognition complex, subunit 1-like (yeast) (ORC1L), mRNA
RIGG10601	0.544	0.0323	ATCGACACAAATTCAAGAAACTGTTGAAGAGAAGGAAGTTTGTGAGAAGGAGGATAAAGGAAGGGCGCAA		ENSGALT00000002364.1 / similar to UP|Q4S5U8_TETNG (Q4S5U8) Chromosome 9 SCAF14729, whole genome shotgun sequence. (Fragment), partial (52%) / --- / --- / --- / --- / --- / --- / --- / 603473293F1 CSEQCHN70 Gallus gallus cDNA clone ChEST352j23 5', mRNA sequence / Transcribed locus
RIGG01237	0.542	0.0326	ACAAGCCTTTTGTAGTCAGTGTGGGTAAGAGGAGCTGGGACACAACTGTAAATATTTCAATAAAACATGC	Weakly similar to Q9P7K8 (Q9P7K8) Similar to Spt4 transcription initiation pr	Weakly similar to Q9P7K8 (Q9P7K8) Similar to Spt4 transcription initiation pr / homologue to UP|SPT41_MOUSE (P63271) Transcription elongation factor SPT4 1 (DRB sensitivity-inducing factor small subunit 1) (DSIF small subunit 1), complete / --- / --- / / --- / --- / --- / --- / --- / ---

Gene Name	Fold Change	p-value	SEQUENCE	Array Description	Blast/Database Description
RIGG08718	0.542	0.0373	CACTTGCAAGGGAAATACTGTGGGACAACGAAGCAAGAGACTGTGTACAAATTTCTGACAGCTGCCTCAG		ENSGALG00000009347.1 / --- / gPGC_EST09496 Embryonic gonadal PGC cDNA Library Gallus gallus cDNA 5', mRNA sequence / Transcribed locus, weakly similar to NP_570106.1 ankyrin repeat, SAM and basic leucine zipper domain containing 1 [Rattus norvegicus] / --- / PREDICTED: Gallus gallus similar to Gasz (LOC417768), mRNA. / PREDICTED: similar to Gasz; / --- / --- / gPGC_EST09496 Embryonic gonadal PGC cDNA Library Gallus gallus cDNA 5', mRNA sequence / Transcribed locus, weakly similar to NP_570106.1 ankyrin repeat, SAM and basic leucine zipper domain containing 1 [Rattus norvegicus]
RIGG20195	0.54	0.0429	GTGAACTTGAAGAAACAGCCATAAGAAAAGTGAGGGGAGTCTAGCTTATGTGAAAAGGAGGTCTCAGCAC	Gallus gallus similar to Sec5 protein; homolog of yeast Sec5; exocyst complex component Sec5; exocyst complex component 2 (LOC420889), mRNA	Gallus gallus similar to Sec5 protein; homolog of yeast Sec5; exocyst complex component Sec5; exocyst complex component 2 (LOC420889), mRNA / --- / 603843215F1 CSEQRBN22 Gallus gallus cDNA clone ChEST828m3 5', mRNA sequence / Similar to Sec5 protein; homolog of yeast Sec5; exocyst complex component Sec5; exocyst complex component 2 (LOC420889), mRNA / --- / PREDICTED: Gallus gallus similar to Sec5 protein; homolog of yeast Sec5; exocyst complex component Sec5; exocyst complex component 2 (LOC420889), mRNA. / --- / Similar to Sec5 protein; homolog of yeast Sec5; exocyst complex component Sec5; exocyst complex component 2 (LOC420889), mRNA; / --- / --- / ---
RIGG00697	0.539	0.0315	GTTTACTTCTGTGCGGAGAAACTCACGTTTTCTGTACACCGAAACCTATCGTCATGCAGGTATCTGTGGG	Genome Hit Contig103.78	Genome Hit Contig103.78 / Gallus gallus finished cDNA, clone ChEST1017l21 / Gallus gallus finished cDNA, clone ChEST1017l21 / Finished cDNA, clone ChEST981f9 / --- / --- / --- / --- / --- / Gallus gallus finished cDNA, clone ChEST1017l21 / Finished cDNA, clone ChEST981f9
RIGG19720	0.538	0.0388	AGGAGACAAGCATGACCTTGAAGCAAAGAAGGCATACTCATACCTCCAAACAATCACTCTGCTGAGGACT		ENSGALT00000028093.1 / similar to GB|AAF00105.1|6002708|AF131862 mutant natriuretic peptide receptor 3 {Mus musculus} (exp=-1; wgp=0; cg=0), partial (52%) / 603477304F1 CSEQCHN71 Gallus gallus cDNA clone ChEST363g1 5', mRNA sequence / Transcribed locus, moderately similar to XP_855442.1 PREDICTED: similar to Atrial natriuretic peptide clearance receptor precursor (ANP-C) (ANPRC) (NPR-C) (Atrial natriuretic peptide C-type receptor) [Canis familiaris] / --- / --- / --- / Transcribed locus, moderately similar to XP_855442.1 PREDICTED: similar to Atrial natriuretic peptide clearance receptor precursor (ANP-C) (ANPRC) (NPR-C) (Atrial natriuretic peptide C-type receptor) [Canis familiaris]; / natriuretic peptide receptor C/guanylate cyclase C (atrionatriuretic peptide receptor C); / 603477304F1 CSEQCHN71 Gallus gallus cDNA clone ChEST363g1 5', mRNA sequence / Transcribed locus, moderately similar to XP_855442.1 PREDICTED: similar to Atrial natriuretic peptide clearance receptor precursor (ANP-C) (ANPRC) (NPR-C) (Atrial natriuretic peptide C-type receptor) [Canis familiaris]

Gene Name	Fold Change	p-value	SEQUENCE	Array Description	Blast/Database Description
RIGG10347	0.538	0.0167	ACTGGTTTGAGCTGAGAGGTGGAG GCCGATGTGCATACCTGAACGGGG ACAGGATCAGCTCATCC		ENSGALT00000001702.1 / homologue to UP|Q802S8_CHICK (Q802S8) C-type lectin, partial (80%) / Gallus gallus finished cDNA, clone ChEST162o11 / Finished cDNA, clone ChEST650i15 / similar to C-type lectin isoform 2 / PREDICTED: Gallus gallus similar to C-type lectin (LOC417078), partial mRNA. / PREDICTED: similar to C-type lectin, partial; / Finished cDNA, clone ChEST650i15; / --- / Gallus gallus finished cDNA, clone ChEST63k22 / Finished cDNA, clone ChEST63k22
RIGG09586	0.536	0.0494	CCATGATAACCGGCAGAAGTTCTCT ACCATCAGCACCATCAGCGACATCT CAGTCCTGAAGCTGAAAGTG		ENSGALG00000016605.1 / --- / --- / --- / --- / --- / --- / --- / KIAA0953; / --- / ---
RIGG09516	0.535	0.0172	AGCCAGACAGCAGAGCAGACAAGA AAACTCACGGATGTTGAAACGCAGG TGCTAAATCAAACATCCAGACT		ENSGALG00000016083.1 / homologue to GB|BAA02793.2|14133267|HUMRSC192 {Homo sapiens} (exp=-1; wgp=0; cg=0), partial (85%) / 603104010F1 CSEQCHN03 Gallus gallus cDNA clone ChEST40c4 5', mRNA sequence / Angiopoietin-1 (ANG-1 gene) / / PREDICTED: Gallus gallus Angiopoietin-1 (ANG-1), mRNA. / PREDICTED: similar to Angiopoietin-1 precursor (ANG-1); / Angiopoietin-1 (ANG-1 gene); / --- / 603104010F1 CSEQCHN03 Gallus gallus cDNA clone ChEST40c4 5', mRNA sequence / Angiopoietin-1 (ANG-1 gene)
RIGG18654	0.533	0.0156	GAAAATGTCTACACAACAGCAGAGA GGGTCCGCAAAGAGGTTGGAGAGG TTTCCGTCCTGGTTAACAATG		ENSGALT00000025215.1 / homologue to UP|Q8IZV5_HUMAN (Q8IZV5) Retinol dehydrogenase 10 (RDH10), partial (86%) / Gallus gallus finished cDNA, clone ChEST382n13 / Finished cDNA, clone ChEST382n13 / / PREDICTED: Gallus gallus similar to retinol dehydrogenase 10 (LOC420183), mRNA. / PREDICTED: similar to retinol dehydrogenase 10; / Finished cDNA, clone ChEST382n13; / --- / Gallus gallus finished cDNA, clone ChEST382n13 / Finished cDNA, clone ChEST382n13
RIGG00354	0.533	0.02	CATGCTGTCTGTGCTCATTAATCACT GCCTTGTCAACTAAGATGAATGGTT ACTACATGGTGATTTTCGCT	Gallus gallus mRNA for hypothetical protein, clone 34i5	Gallus gallus mRNA for hypothetical protein, clone 34i5 / acetyl-Coenzyme A acetyltransferase 2 (acetoacetyl Coenzyme A thiolase) [Gallus gallus] / Gallus gallus mRNA for hypothetical protein, clone 34i5 / Acetyl-Coenzyme A acetyltransferase 2 (acetoacetyl Coenzyme A thiolase) (ACAT2), mRNA / --- / --- / --- / Acetyl-Coenzyme A acetyltransferase 2 (acetoacetyl Coenzyme A thiolase) (ACAT2), mRNA; / --- / Gallus gallus mRNA for hypothetical protein, clone 34i5 / Acetyl-Coenzyme A acetyltransferase 2 (acetoacetyl Coenzyme A thiolase) (ACAT2), mRNA

Gene Name	Fold Change	p-value	SEQUENCE	Array Description	Blast/Database Description
RIGG10315	0.532	0.0127	AGGGGTCACGGTGGCCTTGATCATGCAGATCTACTTTGGAGACCCTCAGCTCTTCCAGCGTGGAG		ENSGALT00000001589.1 / similar to UP|GGT4_MOUSE (Q99JP7) Gamma-glutamyltransferase 4 precursor (Gamma-glutamyltranspeptidase 4) (Gamma-glutamyltransferase-like 3) [Contains: Gamma-glutamyltransferase 4 heavy chain; Gamma-glutamyltransferase 4 light chain] , partial (30%) / --- / --- / similar to gamma-glutamyltransferase-like 3 / --- / --- / --- / gamma-glutamyltransferase-like 3; / --- / ---
RIGG16467	0.532	0.0413	GGTCTCCAGAGCAATACAGACACAACTTCAGCTTCAAATATTTTCACTTTTACCTAACGACAGCCATCCAG		ENSGALT00000019175.1 / --- / Gallus gallus ADP-ribosyltransferase (CHAT1), mRNA / ADP-ribosyltransferase / similar to Dombrock blood group carrier molecule / PREDICTED: Gallus gallus similar to Dombrock blood group; ADP-ribosyltransferase 4 (LOC427879), mRNA. / PREDICTED: similar to Dombrock blood group; ADP-ribosyltransferase 4; / --- / --- / --- / ---
RIGG20385	0.531	0.0123	GGTCTCTGATGGAAGGCTGGAAGATGGTCTGCGAAGAAGTCTTTTGGCAGCACAACTGTTACTCA	Sox11 transcription factor (SOX11), mRNA	Sox11 transcription factor (SOX11), mRNA / --- / --- / --- / SOX11: Transcription factor SOX-11 / --- / --- / --- / --- / Gallus gallus SRY (sex determining region Y)-box 11 (SOX11), mRNA / Sox11 transcription factor (Sox11)
RIGG11267	0.531	0.0125	TTTCCATGCTTCTGGATAGGAGCTGCCCTTTTACCATCGTGTTTTGCTGATCCAGTTGTGTGTGGGTTTG		ENSGALT00000004286.1 / --- / --- / --- / --- / --- / --- / --- / --- / --- / ---
RIGG08154	0.53	0.0272	AGAAAAGGAAAGGAAAGGCAAACTGTGACACTCAATTCTCTCATGTCACTTGGTTCAAAGTGTTGACAGCCATT		ENSGALG00000004930.1 / --- / Gallus gallus cholinergic receptor, muscarinic 5 (CHRM5), mRNA / Cholinergic receptor, muscarinic 5 (CHRM5), mRNA / similar to histamine H1 receptor - bovine / PREDICTED: Gallus gallus similar to histamine H1 receptor - bovine (LOC427580), mRNA. / PREDICTED: similar to histamine H1 receptor - bovine; / --- / --- / --- / ---
RIGG12747	0.529	0.0331	CGGTACCAGCCAAGAGTGCTAATCTTCATAACAGGACCAGGAAAATCAAAAGTGGACCAGCCTGCTAGAA		ENSGALT00000008517.1 / RF|NP_001025894.1|71897037|NM_001030723 amyloid beta precursor protein-binding protein 2 {Gallus gallus} (exp=-1; wgp=0; cg=0), complete / Gallus gallus mRNA for hypothetical protein, clone 5o24 / Amyloid beta precursor protein (cytoplasmic tail) binding protein 2 (APPBP2), mRNA / RCJMB04_5o24: Hypothetical protein / PREDICTED: Gallus gallus similar to amyloid beta precursor protein-binding protein 2; amyloid beta precursor protein (cytoplasmic tail)-binding protein 2; protein interacting with APP tail 1 (LOC417645), mRNA. / PREDICTED: similar to amyloid beta precursor protein-binding protein 2; amyloid beta precursor protein (cytoplasmic tail)-binding protein 2; protein interacting with APP tail 1; / Amyloid beta precursor protein (cytoplasmic tail) binding protein 2 (APPBP2), mRNA; / --- / Gallus gallus mRNA for hypothetical protein, clone 5o24 / Amyloid beta precursor protein (cytoplasmic tail) binding protein 2 (APPBP2), mRNA

Gene Name	Fold Change	p-value	SEQUENCE	Array Description	Blast/Database Description
RIGG12887	0.528	0.00742	CCAGTGGGATAATTCTGCAGAGAAGAGGAACAGATAAAGTAGCACAACAGGATCATCAGGATGCGGAAGC		ENSGALT00000008922.1 / Gallus gallus finished cDNA, clone ChEST930a11 / gonad_EST10120 Embryonic gonad cDNA Library Gallus gallus cDNA 5', mRNA sequence / Finished cDNA, clone ChEST930a11 / --- / PREDICTED: Gallus gallus similar to Mitochondrial ribosomal protein S23 (LOC417666), mRNA. / PREDICTED: similar to Mitochondrial ribosomal protein S23; / --- / --- / gonad_EST10120 Embryonic gonad cDNA Library Gallus gallus cDNA 5', mRNA sequence / Finished cDNA, clone ChEST930a11
RIGG13335	0.527	0.00684	GACCGCCTGATTCAGCAGATTGAAATGGAGAATACTGTGGATGTGTATGGAGTGGTGTACGATCTTCGAATGC	supporting-cell antigen [Gallus gallus]. [Source:RefSeq;Acc:NM_204621]	supporting-cell antigen [Gallus gallus]. [Source:RefSeq / UP|Q9W6V5_CHICK (Q9W6V5) Supporting-cell antigen precursor, complete / Gallus gallus protein tyrosine phosphatase, receptor type, J (PTPRJ), mRNA / Supporting-cell antigen / Supporting-cell antigen precursor / --- / --- / --- / --- / protein tyrosine phosphatase, receptor type, J; / Gallus gallus protein tyrosine phosphatase, receptor type, J (PTPRJ), mRNA / Supporting-cell antigen
RIGG07445	0.526	0.0163	TTAACAGAGGCATTTTCCTTTTGTTCGAGTCTCTCTGCAGCAGAAAGCCTTAGGAAACACTTGGAAGTCC		Contig_28_forward / Gallus gallus finished cDNA, clone ChEST631o11 / Gallus gallus finished cDNA, clone ChEST631o11 / Finished cDNA, clone ChEST763g21 / --- / --- / --- / --- / --- / Gallus gallus finished cDNA, clone ChEST631o11 / Finished cDNA, clone ChEST763g21
RIGG17887	0.526	0.0335	CTTGCCTTCAACGCCTTGATCCACAGACACAGACCAGAGCTCATCGAGTACGACAAACTCAGAAAGGATG		ENSGALT00000023128.1 / UP|ACTN4_CHICK (Q90734) Alpha-actinin-4 (Non-muscle alpha-actinin 4) (F-actin cross linking protein), complete / Gallus gallus actinin, alpha 4 (ACTN4), mRNA / Alpha-actinin / ACTN4: Alpha-actinin-4 / Gallus gallus actinin, alpha 1 (ACTN1), mRNA. / actinin, alpha 1; / Alpha-actinin; / --- / Gallus gallus actinin, alpha 4 (ACTN4), mRNA / Alpha-actinin
RIGG09180	0.525	0.0253	TGGCCTTAGCTATCTATATCCCCTTTCCAGAAGATGATTCCAATGCCACCAATTCCAATCTGATATGTGAA		ENSGALG00000013012.1 / --- / Gallus gallus L-type voltage-gated calcium channel alpha1D subunit ChCaChA1D (LOC395895), mRNA / L-type voltage-gated calcium channel alpha1D subunit ChCaChA1D / / --- / --- / --- / --- / --- / ---
RIGG16249	0.525	0.0399	ACAGTCTTTCCTTTGTCGGACAAGTTGAGTTTCAGTCAAGGAGCAGCGATTGCAATACCCTACTTCACTG		ENSGALT00000018533.1 / similar to GB|AAH03800.1|13277837|BC003800 Cryz protein {Mus musculus} (exp=-1; wgp=0; cg=0), partial (92%) / Gallus gallus mRNA for hypothetical protein, clone 33h20 / Hypothetical protein, clone 33h20 / RCJMB04_33h20: Hypothetical protein / --- / --- / Hypothetical protein, clone 33h20; / --- / Gallus gallus mRNA for hypothetical protein, clone 33h20 / Hypothetical protein, clone 33h20

Gene Name	Fold Change	p-value	SEQUENCE	Array Description	Blast/Database Description
RIGG16631	0.524	0.0445	TACTTGGAATGTGGAGAACCAAAAC TGTCACTAAAATGTCTGTTTCAGTCA AAGGAGTTCCGACTTTGTGC		ENSGALT00000019695.1 / Gallus gallus finished cDNA, clone ChEST924b10 / Gallus gallus finished cDNA, clone ChEST924b10 / Finished cDNA, clone ChEST924b10 / similar to KIAA0342 protein / PREDICTED: Gallus gallus similar to KIAA0342 protein (LOC420728), mRNA. / PREDICTED: similar to KIAA0342 protein; / Finished cDNA, clone ChEST924b10;Transcribed locus, weakly similar to XP_473751.1 OSJNBa0083N12.5 [Oryza sativa (japonica cultivar-group)]; / --- / Gallus gallus finished cDNA, clone ChEST924b10 / Finished cDNA, clone ChEST924b10
RIGG08391	0.524	0.0288	TGAGGGGAGGATCCAGGTGCTTGA GAAGGAGCTGAGCCGTTACATGAG GGCAAACCAGGAGCTGA		ENSGALG00000006611.1 / similar to UP|Q8IW72_HUMAN (Q8IW72) KIF7 protein (Fragment), partial (33%) / --- / --- / --- / --- / --- / --- / kinesin family member 7; / --- / ---
RIGG11696	0.524	0.0396	GACGGCACTGAGGTCACGGTGAAG CCAGATGTCCAGGACCACTGCTTTT ACCAAGGCCATGTTGA		ENSGALT00000005542.1 / --- / 603470339F1 CSEQRBN22 Gallus gallus cDNA clone ChEST345n10 5', mRNA sequence / Transcribed locus, strongly similar to XP_421552.1 PREDICTED: similar to ADAM 8 precursor (A disintegrin and metalloproteinase domain 8) (Cell surface antigen MS2) (CD156a antigen) (CD156) [Gallus gallus] / --- / --- / --- / --- / ADAM metallopeptidase domain 8; / --- / ---
RIGG17746	0.523	0.00524	AGAGACATCTGAATATGAACTACAA GAGCGAAGGACAAGGACCATTAGCT GTATCTGAGATACTGGATGC		ENSGALT00000022775.1 / --- / 603218770F1 CSEQRBN14 Gallus gallus cDNA clone ChEST209l18 5', mRNA sequence / Finished cDNA, clone ChEST209l18 / --- / PREDICTED: Gallus gallus similar to KIAA0802 protein (LOC426050), partial mRNA.PREDICTED: Gallus gallus similar to KIAA0802 protein (LOC429927), partial mRNA. / PREDICTED: similar to KIAA0802 protein, partial;PREDICTED: similar to KIAA0802 protein, partial; / --- / --- / --- / ---
RIGG10977	0.523	0.0182	GGTGACACAAGGAGCATCTCAGGAA AGCAGTCCTCAGGGAAGCGGTGAC ATGGACGTGAACACGG		ENSGALT00000003499.1 / similar to UP|Q6P038_BRARE (Q6P038) High mobility group AT-hook 2, partial (35%) / --- / --- / / --- / --- / --- / --- / --- / ---
RIGG13444	0.521	0.036	TGACTGACACCATGAAGGACATGAA CAAGTTCCCTTGCCTGGCTTGGGTA GACCTCGGCAACAAC		ENSGALT00000010637.1 / Gallus gallus finished cDNA, clone ChEST759p7 / --- / --- / hypothetical protein XP_415239 / PREDICTED: Gallus gallus similar to hypothetical protein LOC192734 (LOC416946), mRNA. / PREDICTED: hypothetical protein XP_415239; / --- / --- / --- / ---
RIGG18457	0.521	0.0342	CAATAAAGATGCTACGAGCAGACCA GCAGACAACGTTCAGATACCACAGC TAATCAGAGAAATTGGTGGC		ENSGALT00000024708.1 / homologue to GB|AAH48287.1|29387254|BC048287 SEC63-like protein {Homo sapiens} (exp=-1; wgp=0; cg=0), partial (64%) / --- / --- / similar to SEC63 homolog / PREDICTED: Gallus gallus similar to Translocation protein SEC63 homolog (LOC421774), mRNA. / PREDICTED: similar to Translocation protein SEC63 homolog; / --- / --- / --- / ---
RIGG14021	0.521	0.0493	CTGGGTTGCAGCGGTGTGTCCTACA ATCATAGAACAGTTTGAGTTGGAAG GGATCCTTAAGGGCCATCTG		ENSGALT00000012308.1 / Gallus gallus finished cDNA, clone ChEST133k2 / Gallus gallus finished cDNA, clone ChEST133k2 / Finished cDNA, clone ChEST133k2 / --- / --- / --- / --- / --- / Gallus gallus finished cDNA, clone ChEST133k2 / Finished cDNA, clone ChEST133k2

Gene Name	Fold Change	p-value	SEQUENCE	Array Description	Blast/Database Description
RIGG14809	0.52	0.000452	TGAAATGGAGCTGGGAAAATATGGTCAGGAATCTGAATTTCTATGCCTGGAGTTTGATGAGGCCAAGGTG		ENSGALT00000014515.1 / Gallus gallus finished cDNA, clone ChEST497m3 / --- / --- / --- / --- / --- / --- / copper metabolism (Murr1) domain containing 1; / --- / ---
RIGG05989	0.519	0.0293	TATGGAGGTGGGAGGTTTGGGTTTGCTTCTGCTGAGGTGGATCAATCAGAGAATGGTTTGGGATAGAGAC	Genome Hit Contig2.857	Genome Hit Contig2.857 / Gallus gallus finished cDNA, clone ChEST829k24 / --- / --- / --- / --- / --- / --- / --- / ---
RIGG10783	0.519	0.00506	CACCGTAATACTTTGCTACGCCAGGGAAGGAAGGAAATTACACTGATAGTGGAACAGTTACAGATAGAGAT		ENSGALT00000002937.1 / Gallus gallus finished cDNA, clone ChEST201b18 / Gallus gallus finished cDNA, clone ChEST201b18 / Finished cDNA, clone ChEST599e2 / --- / --- / --- / --- / --- / Gallus gallus finished cDNA, clone ChEST201b18 / Finished cDNA, clone ChEST599e2
RIGG07483	0.518	0.0232	ACACAGAAAACGCAACAAAGGAAACAACACTCCACACAACGACCACAACAAGGGATCCAGAGGTG		Contig_46_forward / --- / --- / --- / --- / --- / --- / --- / --- / --- / ---
RIGG15141	0.517	0.00835	CATCGAGGATCTGAGCGAGAAGGACAAACTGAAAATGGAAGTGGAGCAGCTCCGGAAAGAAGTGAAGCTG		ENSGALT00000015493.1 / similar to UP|GBG11_HUMAN (P61952) Guanine nucleotide-binding protein G(I)/G(S)/G(O) gamma-11 subunit precursor, complete / RJA031F04.ab1 RJbrain Gallus gallus cDNA 5', mRNA sequence / Transcribed locus, moderately similar to NP_071791.1 guanine nucleotide binding protein gamma 11 [Rattus norvegicus] / / --- / --- / Transcribed locus, moderately similar to NP_071791.1 guanine nucleotide binding protein gamma 11 [Rattus norvegicus]; / guanine nucleotide binding protein (G protein), gamma 11; / RJA031F04.ab1 RJbrain Gallus gallus cDNA 5', mRNA sequence / Transcribed locus, moderately similar to NP_071791.1 guanine nucleotide binding protein gamma 11 [Rattus norvegicus]
RIGG17886	0.517	0.0258	TCCCTTGGAAGCACAACGCCAGGAAGGATGTCACCAGCAGCGACGTGGAGATCTTCAAGGCCTGG		ENSGALT00000023115.1 / UP|IRF3_CHICK (Q90643) Interferon regulatory factor 3 (IRF-3), complete / Gallus gallus interferon regulatory factor 7 (IRF7), mRNA / Interferon regulatory factor 3 (cIRF-3) / IRF3: Interferon regulatory factor 3 / Gallus gallus interferon regulatory factor 3 (LOC396330), mRNA. / interferon regulatory factor 3; / --- / --- / Gallus gallus interferon regulatory factor 7 (IRF7), mRNA / Interferon regulatory factor 3 (cIRF-3)

Gene Name	Fold Change	p-value	SEQUENCE	Array Description	Blast/Database Description
RIGG20417	0.516	0.0485	CGTCTCTCCAGATATATCACGACTC AAATCTCTTCCCCAGTGTAATATCCT CTCATCAGATGGAGATTCTGGT	similar to Receptor-interacting serine/threonine-protein kinase 2 (RIP-like interacting CLARP kinase) (Receptor-interacting protein 2) (RIP-2) (CARD-containing interleukin-1 beta converting enzyme associated kinase) (CARD-containing IL-1 beta ICE-k... (LOC420215), mRNA	similar to Receptor-interacting serine/threonine-protein kinase 2 (RIP-like interacting CLARP kinase) (Receptor-interacting protein 2) (RIP-2) (CARD-containing interleukin-1 beta converting enzyme associated kinase) (CARD-containing IL-1 beta ICE-k... (LOC420215), mRNA / RF|NP_001026114.1|71896457|NM_001030943 receptor-interacting serine-threonine kinase 2 {Gallus gallus} (exp=-1; wgp=0; cg=0), complete / Gallus gallus mRNA for hypothetical protein, clone 15n4 / Receptor-interacting serine-threonine kinase 2 (RIPK2), mRNA / RCJMB04_15n4: Hypothetical protein / --- / --- / Receptor-interacting serine-threonine kinase 2 (RIPK2), mRNA; / --- / Gallus gallus mRNA for hypothetical protein, clone 15n4 / Receptor-interacting serine-threonine kinase 2 (RIPK2), mRNA
RIGG19868	0.516	0.0318	CCCCGAAGAGGTGTGGAAGTCAGC AGAGATCACCCAGGATTACAAAGAC GGCGATCGGAGCCTCC		ENSGALT00000028410.1 / --- / Gallus gallus myosin VA (heavy polypeptide 12, myoxin) (MYO5A), mRNA / Dilute mRNA for myosin I heavy chain isoform / --- / --- / --- / --- / --- / --- / ---
RIGG09139	0.516	0.0153	TCCTGGAAGAAAATGGATTCACTAC TCAGCAGTCGGAGGTGATTGTGTCT GCATTCGTGAAAATCGTGAA		ENSGALG00000012699.1 / --- / AJ446712 riken1 Gallus gallus cDNA clone 14g5r1, mRNA sequence / Transcribed locus, moderately similar to XP_001060245.1 PREDICTED: hypothetical protein [Rattus norvegicus] / --- / PREDICTED: Gallus gallus similar to C6orf79 protein (LOC420837), mRNA. / PREDICTED: similar to C6orf79 protein; / --- / --- / AJ446712 riken1 Gallus gallus cDNA clone 14g5r1, mRNA sequence / Transcribed locus, moderately similar to XP_001060245.1 PREDICTED: hypothetical protein [Rattus norvegicus]
RIGG18839	0.516	0.0113	GGATGCTTATTCATTCTTTGTGATCA CGAAAGACAAGGGAGGAGAACTGG TGGACAAACCTCTGAAAATT		ENSGALT00000025702.1 / --- / --- / --- / similar to intestinal peptide-associated transporter HPT-1 / PREDICTED: Gallus gallus similar to Cadherin-17 precursor (Liver-intestine-cadherin) (LI-cadherin) (Intestinal peptide-associated transporter HPT-1) (LOC420225), mRNA. / PREDICTED: similar to Cadherin-17 precursor (Liver-intestine-cadherin) (LI-cadherin) (Intestinal peptide-associated transporter HPT-1); / Finished cDNA, clone ChEST496n5; / --- / Gallus gallus finished cDNA, clone ChEST150c3 / Finished cDNA, clone ChEST496n5
RIGG01002	0.514	0.0445	AGGTGTGGGGTAGAGTGTGTAAAGC TACTGTGTGTTAAACTGATGGGATG TTACTGAAAGGTACAGTATTC	Weakly similar to Q8IH42 (Q8IH42) GH25305p (Fragment)	Weakly similar to Q8IH42 (Q8IH42) GH25305p (Fragment) / GB|AAB02656.1|1381181|OCU58652 ubiquitin-conjugating enzyme E2-32k {Oryctolagus cuniculus} (exp=-1; wgp=0; cg=0), partial (96%) / Gallus gallus finished cDNA, clone ChEST129g2 / Finished cDNA, clone ChEST146n3 / RCJMB04_34a5: Hypothetical protein / PREDICTED: Gallus gallus similar to ubiquitin-conjugating enzyme E2-32k (LOC427021), partial mRNA. / PREDICTED: similar to ubiquitin-conjugating enzyme E2-32k, partial; / Ubiquitin-conjugating enzyme E2R 2 (UBE2R2), mRNA; / --- / Gallus gallus finished cDNA, clone ChEST129g2 / Finished cDNA, clone ChEST146n3

Gene Name	Fold Change	p-value	SEQUENCE	Array Description	Blast/Database Description
RIGG16202	0.514	0.0333	TGCTTGGACATTTTAAAAGACAACT GGAGTCCAGCATTAACCATTTCTAA AGTTCTCCTCTCCATCTGCTC		ENSGALT00000018414.1 / homologue to UP|Q6AY98_RAT (Q6AY98) Ube2e2 protein (Fragment), partial (82%) / Gallus gallus finished cDNA, clone ChEST21k18 / Finished cDNA, clone ChEST460n14 / similar to ubiquitin-conjugating enzyme UbcH6 isoform 1 / --- / --- / --- / ubiquitin-conjugating enzyme E2E 1 (UBC4/5 homolog, yeast); / Gallus gallus finished cDNA, clone ChEST21k18 / Finished cDNA, clone ChEST460n14
RIGG11288	0.511	0.0248	TCCCATCCAAAAGTAATACAGCCAA AGTGTTCATTGAAGTGAAGGATGAA AACGATCATGCGCCTGTCTT		ENSGALT00000004345.1 / --- / Gallus gallus / Gallus gallus / Protocadherin-15-CD3 isoform 1 / --- / --- / --- / --- / Gallus gallus / Gallus gallus
RIGG09801	0.507	0.00579	GGATGCATCAGAACTAGGCAAACGG AGGACAAGGAAAGAAGGGAATGAAT ATAAGATGATGCAGAACAGAA		ENSGALT00000000242.1 / homologue to UP|Q96A21_HUMAN (Q96A21) WIG-1/PAG608 protein (Zinc finger protein WIG1), partial (51%) / --- / --- / / PREDICTED: Gallus gallus similar to p53 target zinc finger protein isoform 2; zinc finger protein WIG1; WIG-1/PAG608 protein (LOC425428), partial mRNA. / PREDICTED: similar to p53 target zinc finger protein isoform 2; zinc finger protein WIG1; WIG-1/PAG608 protein, partial; / --- / --- / --- / ---
RIGG08697	0.504	0.0426	CAATGAGCTGGTGAGGTATGTGAAA ACACAAGAGGACACTGTGGCTGAAG CTGAAGCCGATGTGGAGATC		ENSGALG00000009167.1 / --- / --- / --- / --- / --- / --- / --- / --- / --- / ---
RIGG04290	0.502	0.0473	CTGAAATGCTGGATGAGCTCTTGGA GAAGAGGAGAGAGAAACTGGACTC CGTCATTGAGTTCAGCATCCC	Similar to Q9U915 (Q9U915) DAK2 protein (SD09634p) (CG3140 protein)	Similar to Q9U915 (Q9U915) DAK2 protein (SD09634p) (CG3140 protein) / Gallus gallus finished cDNA, clone ChEST512l15 / gPGC_EST00600 Embryonic gonadal PGC cDNA Library Gallus gallus cDNA 5', mRNA sequence / Finished cDNA, clone ChEST211a22 / / --- / --- / --- / adenylate kinase 2; / gPGC_EST00600 Embryonic gonadal PGC cDNA Library Gallus gallus cDNA 5', mRNA sequence / Finished cDNA, clone ChEST211a22
RIGG12896	0.501	0.0444	GAGAGAGAGGATCAGAGGAGTGAG CTTAGACAAGGAAGAAGTTGAGAAA ATCGAGCAAGTAGCAATACAA		ENSGALT00000008944.1 / --- / --- / --- / similar to kinase A anchor protein / PREDICTED: Gallus gallus similar to A-kinase anchor protein 1 isoform 1 precursor; A-kinase anchor protein, 149kD; spermatid A-kinase anchor protein 84; protein kinase A anchoring protein 1; dual-specificity A-kinase anchoring protein 1 (LOC417668), mRNA. / PREDICTED: similar to A-kinase anchor protein 1 isoform 1 precursor; A-kinase anchor protein, 149kD; spermatid A-kinase anchor protein 84; protein kinase A anchoring protein 1; dual-specificity A-kinase anchoring protein 1; / --- / --- / --- / ---
RIGG10875	0.501	0.0178	GCCAAGCCATGACCCTGCTTTATTC ACACAGCACAGAGCACATCACTACA TTGATCAAATCCCAGCAGAG		ENSGALT00000003223.1 / Gallus gallus finished cDNA, clone ChEST541e2 / Gallus gallus finished cDNA, clone ChEST40g24 / Finished cDNA, clone ChEST541e2 / --- / --- / --- / --- / --- / Gallus gallus finished cDNA, clone ChEST40g24 / Finished cDNA, clone ChEST541e2

Gene Name	Fold Change	p-value	SEQUENCE	Array Description	Blast/Database Description
RIGG20359	0.501	0.0059	GGGTTCTACATCTGCATGAACAAGAAGGGGAAACTGATCGGCAAGAGTAACGGCAAAGGCAAGGA	fibroblast growth factor 8 (FGF-8), mRNA	fibroblast growth factor 8 (FGF-8), mRNA / UP|Q4R0X9_CHICK (Q4R0X9) Fibroblast growth factor 8, complete / Gallus gallus fibroblast growth factor 8 FGF8 mRNA, partial cds / Fibroblast growth factor 8 FGF8 / FGF8: Fibroblast growth factor 8 precursor / --- / --- / Fibroblast growth factor 8 FGF8; / --- / Gallus gallus fibroblast growth factor 8 FGF8 mRNA, partial cds / Fibroblast growth factor 8 FGF8
RIGG19859	0.5	0.015	GTGCACTACAATAAAAGTGGAGGGTCTCACACACTGCAGAAGATGTTTGGCTGTGACATCCTGGAGGACG		ENSGALT00000028391.1 / similar to UP|Q9BCW3_CHICK (Q9BCW3) MHC Rfp-Y class I alpha chain, complete / Gallus gallus finished cDNA, clone ChEST952a22 / MHC class I antigen (YFV), mRNA / MHC class I antigen / PREDICTED: Gallus gallus similar to MHC Rfp-Y class I alpha chain (LOC417065), partial mRNA. / PREDICTED: similar to MHC Rfp-Y class I alpha chain, partial; / --- / --- / --- / ---
RIGG09796	0.497	0.0132	CCCTCCTATTTCTCATCGATAGCATCACCCATATTCACAAAGGACTAATCAGCAAGTACAGCTCTGTGCT		ENSGALT00000000231.1 / --- / --- / --- / --- / --- / PREDICTED: similar to DKFZP727M111 protein, partial; / --- / --- / --- / ---
RIGG00978	0.497	0.0327	AATGGAGAAAGACCTTGCTAATATTTTGGACAAGGCCACAAACAGATACTTGCCAGTCTTTGAGAAGGTT	Same gene AF133252; Gallus gallus clone pGCLA5 glutathione S-transferase class-alpha mRNA, partial	Same gene AF133252; Gallus gallus clone pGCLA5 glutathione S-transferase class-alpha mRNA, partial / UP|Q9W6J3_CHICK (Q9W6J3) Glutathione S-transferase class-alpha (Fragment), complete / gonad_EST06783 Embryonic gonad cDNA Library Gallus gallus cDNA 5', mRNA sequence / Clone pGCLA5 glutathione S-transferase class-alpha / Glutathione S-transferase / PREDICTED: Gallus gallus glutathione S-transferase (GST), mRNA. / PREDICTED: glutathione S-transferase; / Glutathione S-transferase (GST); / --- / gonad_EST06783 Embryonic gonad cDNA Library Gallus gallus cDNA 5', mRNA sequence / Clone pGCLA5 glutathione S-transferase class-alpha
RIGG13992	0.496	0.0383	CCCACATGGTCGGAGTGTACACAAACAGGTGTGGTGAGAGATGGGAACTCCAGTTGAAGGGCTCA		ENSGALT00000012230.1 / --- / --- / --- / --- / PREDICTED: Gallus gallus similar to Selenoprotein O (LOC420491), mRNA. / PREDICTED: similar to Selenoprotein O; / --- / --- / --- / ---
RIGG07219	0.495	0.0125	TGCTGAGACTGAGCAAGAGGAGAGGTGGTAATGTAGGTTGGGAATATACATGTAAGGTGCTATTTGTAATT	Contig Hit 505315.1	Contig Hit 505315.1 / Gallus gallus finished cDNA, clone ChEST995b20 / Gallus gallus finished cDNA, clone ChEST995b20 / Finished cDNA, clone ChEST995b20 / --- / --- / --- / --- / --- / Gallus gallus finished cDNA, clone ChEST995b20 / Finished cDNA, clone ChEST995b20
RIGG14425	0.494	0.0499	TGCAGGATTGTGACCAACACACAACTTTCTTGAATGTACTGCCAGTTCTCTGTATCTATCAGTTTCCTGAA	P37NB protein (Fragment). [Source:SPTREMBL;Acc:Q9DEQ5]	P37NB protein (Fragment). [Source:SPTREMBL / UP|Q9DEQ5_CHICK (Q9DEQ5) P37NB protein (Fragment), complete / Gallus gallus finished cDNA, clone ChEST65l10 / P37NB protein / P37NB: P37NB protein (Fragment) / PREDICTED: Gallus gallus p37NB protein (P37NB), mRNA. / PREDICTED: similar to p37NB protein; / P37NB protein; / --- / Gallus gallus finished cDNA, clone ChEST65l10 / P37NB protein

Gene Name	Fold Change	p-value	SEQUENCE	Array Description	Blast/Database Description
RIGG20383	0.494	0.0468	GTGGCAAAGAAACTGGGAGAGATGTGGAACAACACCGCAGCAGATGATAAACAGCCTTACGAAAAGAAGG	High mobility group protein 4 (HMG-4) (High mobility group protein 2a) (HMG-2a). [Source:SWISSPROT;Acc:P40618]	High mobility group protein 4 (HMG-4) (High mobility group protein 2a) (HMG-2a). [Source:SWISSPROT / GB|CAA76978.1|4140289|GGA17968 high mobility group 1 protein {Gallus gallus} (exp=-1; wgp=0; cg=0), complete / Gallus gallus high-mobility group box 1 (HMGB1), mRNA / High mobility group 1 protein / HMG1, RCJMB04_15a21: High mobility group 1 protein / Gallus gallus high mobility group 1 protein (HMG1), mRNA. / high mobility group 1 protein; / High mobility group 1 protein; / --- / Gallus gallus high-mobility group box 1 (HMGB1), mRNA / High mobility group 1 protein
RIGG02519	0.489	0.0178	ATCGACGAGCAATCCAAGGAGAAGAATGAGAAAGCCGGGATCAAGAGGAAAGCAGAAGATGTTTTGGAGG	Similar to BAC26466 (BAC26466) 0 day neonate head cDNA, RIKEN full-len	Similar to BAC26466 (BAC26466) 0 day neonate head cDNA, RIKEN full-len / Gallus gallus finished cDNA, clone ChEST1013f22 / Gallus gallus finished cDNA, clone ChEST69e7 / Finished cDNA, clone ChEST738g7 / --- / --- / --- / --- / --- / --- / ---
RIGG03325	0.489	0.0175	ATTTTCTGTATTGGATTCTGGGACTCAATTCCCCACGACAAGGGTCTCCGTGATGGCTGTGCTGG	Contig Hit 342028.3	Contig Hit 342028.3 / Gallus gallus finished cDNA, clone ChEST481h16 / --- / --- / --- / --- / --- / --- / --- / --- / ---
RIGG17237	0.488	0.00695	TGTCCCTCAAGAATGGATGATGCTGAATGCAGAGAAGTCAATTGCAAGAGCCACCATTTTGGGGTTCAAC		ENSGALT00000021412.1 / --- / Gallus gallus finished cDNA, clone ChEST49o7 / Finished cDNA, clone ChEST49o7 / similar to PI3-kinase / --- / --- / Transcribed locus, weakly similar to XP_520775.1 PREDICTED: phosphoinositide-3-kinase, class 2, gamma polypeptide [Pan troglodytes]; / phosphoinositide-3-kinase, class 2, gamma polypeptide; / --- / ---
RIGG14826	0.482	0.03	AGATGCTACAGAGAAAGAGAGGAAGGCGAAAAGATCATTCAGGATTTTCTCTCTCAGGTGAAAGCACTGC		ENSGALT00000014573.1 / similar to UP|MSH2_HUMAN (P43246) DNA mismatch repair protein Msh2 (MutS protein homolog 2), partial (91%) / Gallus gallus cMSH2 mRNA for hypothetical protein, partial cds / CMSH2 mRNA for hypothetical protein / cMSH2: Hypothetical protein cMSH2 (Fragment) / --- / --- / CMSH2 mRNA for hypothetical protein; / --- / Gallus gallus cMSH2 mRNA for hypothetical protein, partial cds / CMSH2 mRNA for hypothetical protein
RIGG00335	0.481	0.00911	CTCAGGTGGATGAGAAGAGGAAGAAGCGTGACATCCCTGACTACCTATGTGGGAAGATCAGTTTTGAGCT	Gallus gallus mRNA for hypothetical protein, clone 32b21	Gallus gallus mRNA for hypothetical protein, clone 32b21 / UP|STUB1_CHICK (Q5ZHY5) STIP1 homology and U box-containing protein 1 (STIP1 homology and U-box-containing protein 1) , complete / Gallus gallus mRNA for hypothetical protein, clone 32b21 / STIP1 homology and U-box containing protein 1 (STUB1), mRNA / STUB1, RCJMB04_32b21: STIP1 homology and U box-containing protein 1 / --- / --- / STIP1 homology and U-box containing protein 1 (STUB1), mRNA; / --- / Gallus gallus mRNA for hypothetical protein, clone 32b21 / STIP1 homology and U-box containing protein 1 (STUB1), mRNA

Gene Name	Fold Change	p-value	SEQUENCE	Array Description	Blast/Database Description
RIGG20083	0.481	0.0343	GTGTCCTCCTGAAAGAACCTGTGGTTACAGAATCAGAAAACAACAGCCAAATCAAGACAGCAGCCAAACT	Gallus gallus similar to RIKEN cDNA 2810413N20 (LOC415637), mRNA	Gallus gallus similar to RIKEN cDNA 2810413N20 (LOC415637), mRNA / RF|NP_001005834.1|57524844|NM_001005834 cytokine induced apoptosis inhibitor 1 {Gallus gallus} (exp=-1; wgp=0; cg=0), complete / Gallus gallus mRNA for hypothetical protein, clone 19l6 / Cytokine induced apoptosis inhibitor 1 (CIAPIN1), mRNA / --- / PREDICTED: Gallus gallus similar to RIKEN cDNA 2810413N20 (LOC415637), mRNA. / PREDICTED: similar to RIKEN cDNA 2810413N20; / Cytokine induced apoptosis inhibitor 1 (CIAPIN1), mRNA; / --- / Gallus gallus mRNA for hypothetical protein, clone 19l6 / Cytokine induced apoptosis inhibitor 1 (CIAPIN1), mRNA
RIGG05963	0.481	0.00815	CGACCCAATACGTACGTGATCAAACTGTTTGACCGCAGCGTGGATTTGGGGCAGTTCTCGGAGGG	Weakly similar to Q96GY3 (Q96GY3) Similar to RIKEN cDNA 1810054G18 gene	Weakly similar to Q96GY3 (Q96GY3) Similar to RIKEN cDNA 1810054G18 gene / Gallus gallus finished cDNA, clone ChEST825c18 / --- / --- / --- / --- / --- / --- / --- / BX269218 AGENAE Gallus gallus multi-tissues normalized library (gcag) Gallus gallus cDNA clone gcag0018.i.15 3prim, mRNA sequence / Finished cDNA, clone ChEST825c18
RIGG14430	0.48	0.022	TTTCATGATCGTTGGTTACGTGGATGGCGAATTCTTCGGGAAGTACGACAGTAAGAGCCGGTGGG		ENSGALT00000013523.1 / similar to UP|Q9BCW3_CHICK (Q9BCW3) MHC Rfp-Y class I alpha chain, complete / RJA003F02.ab1 RJbrain Gallus gallus cDNA 5', mRNA sequence / MHC class I antigen (YF) mRNA, YFw*16 allele / similar to MHC Rfp-Y class I alpha chain, partial / PREDICTED: Gallus gallus similar to MHC Rfp-Y class I alpha chain (LOC417055), mRNA.PREDICTED: Gallus gallus similar to MHC Rfp-Y class I alpha chain (LOC417075), partial mRNA.PREDICTED: Gallus gallus similar to MHC Rfp-Y class I alpha chain (LOC417076), mRNA.PREDICTED: Gallus gallus similar to MHC Rfp-Y class I alpha chain (LOC417077), partial mRNA. / PREDICTED: similar to MHC Rfp-Y class I alpha chain, partial;PREDICTED: similar to MHC Rfp-Y class I alpha chain;PREDICTED: similar to MHC Rfp-Y class I alpha chain, partial; / MHC class I antigen (YF) mRNA, YFw*16 allele; / --- / --- / ---
RIGG00921	0.48	0.0473	TTCTGTCCAAATTCTATGAAGGCCACGATCCCTGAAACAGATTCTGTGTCCTCCTGTCTTGTATTACAAAT	Similar to NB4M_MOUSE (Q9CQZ5) NADH-ubiquinone oxidoreductase B14 subu	Similar to NB4M_MOUSE (Q9CQZ5) NADH-ubiquinone oxidoreductase B14 subu / homologue to UP|NDUA6_MOUSE (Q9CQZ5) NADH dehydrogenase [ubiquinone] 1 alpha subcomplex subunit 6 (NADH-ubiquinone oxidoreductase B14 subunit) (Complex I-B14) (CI-B14) , partial (85%) / Contig2163 WL/RJ Phraped ESTs Gallus gallus cDNA 5', mRNA sequence / Finished cDNA, clone ChEST976o17 / --- / --- / --- / Finished cDNA, clone ChEST976o17; / NADH dehydrogenase (ubiquinone) 1 alpha subcomplex, 6, 14kDa; / Contig2163 WL/RJ Phraped ESTs Gallus gallus cDNA 5', mRNA sequence / Finished cDNA, clone ChEST976o17

Gene Name	Fold Change	p-value	SEQUENCE	Array Description	Blast/Database Description
RIGG02798	0.48	0.0368	TCGATGCTCTATGGGGATATTTCACGTGTTTGTCTCAGACACAACTTGTAGCGTAACCTCATCAGCTCTG	Weakly similar to Q08525 (Q08525) Reverse transcriptase	Weakly similar to Q08525 (Q08525) Reverse transcriptase / Gallus gallus finished cDNA, clone ChEST38l18 / Gallus gallus finished cDNA, clone ChEST38l18 / Finished cDNA, clone ChEST736p7 / --- / --- / --- / --- / --- / Gallus gallus finished cDNA, clone ChEST38l18 / Finished cDNA, clone ChEST736p7
RIGG01534	0.48	0.0351	TCCTCTCCTTCTAAGGCAGAGAGAGACATGTTGAAAAGAAGTGAGGAACTGGAGAGAGAGGTTGTGGTAG	Similar to EPLI_HUMAN (Q9UHB6) Epithelial protein lost in neoplasm	Similar to EPLI_HUMAN (Q9UHB6) Epithelial protein lost in neoplasm / Gallus gallus finished cDNA, clone ChEST208i24 / Gallus gallus finished cDNA, clone ChEST208i24 / Finished cDNA, clone ChEST208i24 / / --- / --- / --- / LIM domain and actin binding 1; / Gallus gallus finished cDNA, clone ChEST208i24 / Finished cDNA, clone ChEST208i24
RIGG01008	0.475	0.0313	CATAGCACTGGTGGCTGTCGTCCTTCTGCTCGTGATCATCATACCCATAGTCCTGAAGTACCATACCTGA	Weakly similar to BAC37151 (BAC37151) Adult male medulla oblongata cDNA, RIKE	Weakly similar to BAC37151 (BAC37151) Adult male medulla oblongata cDNA, RIKE / homologue to UP|VAMP4_HUMAN (O75379) Vesicle-associated membrane protein 4 (VAMP-4), complete / gPGC_EST05224 Embryonic gonadal PGC cDNA Library Gallus gallus cDNA 5', mRNA sequence / Finished cDNA, clone ChEST12e2 / hypothetical protein / --- / --- / --- / vesicle-associated membrane protein 4; / gPGC_EST05224 Embryonic gonadal PGC cDNA Library Gallus gallus cDNA 5', mRNA sequence / Finished cDNA, clone ChEST12e2
RIGG16546	0.472	0.0497	ACCTGGAAAGCAGAGTAGAAGAGATCATAAAGAAGCAGGGTGAAGGAGTGCCAGACTTAGTCCACGTGAT		ENSGALT00000019443.1 / homologue to UP|PP2CA_RAT (P20650) Protein phosphatase 2C isoform alpha (PP2C-alpha) (IA) (Protein phosphatase 1A) , partial (95%) / spleen_EST01223 Spleen cDNA Library Gallus gallus cDNA 3', mRNA sequence / Transcribed locus, strongly similar to NP_066283.1 protein phosphatase 1A isoform 1 [Homo sapiens] / / PREDICTED: Gallus gallus similar to protein phosphatase 1A isoform 1; protein phosphatase 2C alpha isoform (LOC423525), mRNA. / PREDICTED: similar to protein phosphatase 1A isoform 1; protein phosphatase 2C alpha isoform; / --- / --- / spleen_EST01223 Spleen cDNA Library Gallus gallus cDNA 3', mRNA sequence / Transcribed locus, strongly similar to NP_066283.1 protein phosphatase 1A isoform 1 [Homo sapiens]
RIGG18718	0.471	0.045	TGGATTTTGCCAAGAATACACCCAATGACACTGTACTTCTCAGTATTTGCTAAGATCCGTACCATGGGCC		ENSGALT00000025371.1 / --- / --- / --- / --- / --- / --- / --- / --- / --- / ---
RIGG12834	0.47	0.0454	ATCCAATGGCAGCACTCAGGAAGTCGTCGAACAATAGGAACAAGGAGAATGAGTTCAGAGAAGGTGGTGG		ENSGALT00000008757.1 / Gallus gallus finished cDNA, clone ChEST206j6 / 602554346F1 CSEQCHL01 Gallus gallus cDNA clone ChEST2k12 5', mRNA sequence / Finished cDNA, clone ChEST206j6 / --- / PREDICTED: Gallus gallus hypothetical gene supported by CR391242 (LOC423752), mRNA. / PREDICTED: hypothetical protein XP_430091; / --- / --- / --- / ---

Gene Name	Fold Change	p-value	SEQUENCE	Array Description	Blast/Database Description
RIGG05982	0.469	0.0387	TTCTTGCTGAAGTGATTGTTCCTAG CAAAAGAGAAGATTGGCAGTTTATA GCTCTGCTTTTACCTCTCCT	Genome Hit Contig10.240	Genome Hit Contig10.240 / similar to GB|AAH73707.1|49116980|BC073707 LOC443688 protein {Xenopus laevis} (exp=-1; wgp=0; cg=0), partial (4%) / Gallus gallus finished cDNA, clone ChEST828e18 / Finished cDNA, clone ChEST828e18 / --- / --- / --- / --- / --- / Gallus gallus finished cDNA, clone ChEST828e18 / Finished cDNA, clone ChEST828e18
RIGG14660	0.468	0.013	GCTGTACCTCTGTGTTGTATTCCAG CAGCACTCTGAGTTTTCTGTTCTTCA GAATAAGGTCACAAGTCTTG		ENSGALT00000014163.1 / Gallus gallus finished cDNA, clone ChEST1022f6 / Gallus gallus finished cDNA, clone ChEST1017g15 / Finished cDNA, clone ChEST1017g15 / similar to cubilin / --- / --- / Transcribed locus, weakly similar to NP_445784.1 cubilin [Rattus norvegicus]; / --- / --- / ---
RIGG07351	0.468	0.0474	AGGCCTAGGAGGTTAATGGAGAGG AGTATTAGGATAAGTGAGGTGAGTA GGAGGGCTCATTTGTGACC		Contig_15_reverse / similar to gb|AF069429.1|SCMITSEQ1 Struthio camelus tRNA-Phe gene, partial sequence; 12S ribosomal RNA gene, complete sequence; tRNA-Val gene, complete sequence; 16S ribosomal RNA gene, complete sequence; tRNA-Leu gene, complete sequence; NADH dehydrogenase subunit 1 gene, complete cds; tRNA-Ile, tRNA-Gln, and tRNA-Met genes, complete sequence; NADH dehydrogenase subunit 2 gene, complete cds; tRNA-Trp, tRNA-Ala, tRNA-Asn, tRNA-Cys, and tRNA-Tyr genes, complete sequence; cytochrome oxidase subunit 1 gene, complete cds; tRNA-Ser and tRNA-Asn genes, complete sequence; cytochrome oxidase subunit 2 gene, complete cds; tRNA-Lys gene, complete sequence; ATPase 8 and ATPase 6 genes, complete cds; and cytochrome oxidase subunit 3 gene, partial cds, mitochondrial genes for mitochondrial products, partial (5%) / Gallus gallus mRNA for hypothetical protein, clone 7p19 / Serine/threonine kinase receptor associated protein (STRAP), mRNA / --- / PREDICTED: Gallus gallus similar to UNR-interacting protein (Serine-threonine kinase receptor-associated protein) (LOC418175), mRNA. / PREDICTED: similar to UNR-interacting protein (Serine-threonine kinase receptor-associated protein); / Serine/threonine kinase receptor associated protein (STRAP), mRNA; / --- / --- / ---
RIGG16298	0.465	0.0405	ACCCTGAGGCCAGAAGAGAAACAGA GGCACACAGAAAATCAGGTACCCAT GGCAAGCTGGATGCT	Ribonuclease CL2 (EC 3.1.27.-) (Poly C preferential ribonuclease) (RNase CL2). [Source:SWISSPROT;Acc:P81476]	Ribonuclease CL2 (EC 3.1.27.-) (Poly C preferential ribonuclease) (RNase CL2). [Source:SWISSPROT / UP|Q5GAL6_CHICK (Q5GAL6) Ribonuclease CL2 (Liver ribonuclease A precursor), complete / gPGC_EST10393 Embryonic gonadal PGC cDNA Library Gallus gallus cDNA 5', mRNA sequence / Liver ribonuclease A precursor / Ribonuclease CL2 / PREDICTED: Gallus gallus similar to RNase CL2=poly C preferential ribonuclease (LOC422633), mRNA. / PREDICTED: similar to RNase CL2=poly C preferential ribonuclease; / Liver ribonuclease A precursor; / --- / --- / ---

Gene Name	Fold Change	p-value	SEQUENCE	Array Description	Blast/Database Description
RIGG10978	0.464	0.0487	GCATATCTGAACAGGACCTCGACGAAATGAACATTGAAATCATTCGCAACACACTGTATAAGGCATACCT		ENSGALT00000003500.1 / homologue to GB|AAC83085.1|3955100|MMU13840 vacuolar adenosine triphosphatase subunit D {Mus musculus} (exp=-1; wgp=0; cg=0), partial (77%) / Gallus gallus mRNA for hypothetical protein, clone 37b12 / ATPase, H+ transporting, lysosomal 38kDa, V0 subunit D2 (ATP6V0D2), mRNA / / PREDICTED: Gallus gallus similar to Ac39/physophilin (LOC415674), mRNA. / PREDICTED: similar to Ac39/physophilin; / --- / --- / gPGC_EST05651 Embryonic gonadal PGC cDNA Library Gallus gallus cDNA 5', mRNA sequence / Finished cDNA, clone ChEST822i10
RIGG15585	0.463	0.0441	TGTCATGGTGGTTGTGTGCTTGATGGTGTTGCTCAGCTCAGCTCTTTTCTTCTCCTTTCCCAACAGATATG		ENSGALT00000016664.1 / --- / --- / --- / similar to keratin 6 irs3 / --- / --- / --- / --- / --- / ---
RIGG17421	0.463	0.0338	AAGTTGAGGAAGAAGGAGAAATTGTTATGGTAAAGGAACACAGGGAGCTGGATCGCAGTGGAACCAGAAA		ENSGALT00000021957.1 / --- / --- / --- / hypothetical protein / PREDICTED: Gallus gallus similar to Rap guanine nucleotide exchange factor 6 (PDZ domain containing guanine nucleotide exchange factor 2) (PDZ-GEF2) (RA-GEF-2) (LOC426124), partial mRNA.PREDICTED: Gallus gallus similar to Rapgef6 protein (LOC430086), partial mRNA.PREDICTED: Gallus gallus similar to Rapgef6 protein (LOC431469), partial mRNA. / PREDICTED: similar to Rap guanine nucleotide exchange factor 6 (PDZ domain containing guanine nucleotide exchange factor 2) (PDZ-GEF2) (RA-GEF-2), partial;PREDICTED: similar to Rapgef6 protein, partial;PREDICTED: similar to Rapgef6 protein, partial; / Transcribed locus, weakly similar to XP_553211.1 ENSANGP00000025467 [Anopheles gambiae str. PEST];Transcribed locus, moderately similar to XP_220428.4 PREDICTED: similar to Rap guanine nucleotide exchange factor (GEF) 6 [Rattus norvegicus];Transcribed locus, weakly similar to NP_001023389.1 PDZ eXchange Factor family member (pxf-1) [Caenorhabditis elegans]; / --- / --- / ---
RIGG14650	0.463	0.0238	ACTACCTCCATGACCGGAACATCATACACCGTGACCTCAAGTGTGAGAACATACTGCTCAGTGCTGATGG		ENSGALT00000014138.1 / --- / testis_EST00117 Testis cDNA Library Gallus gallus cDNA 3', mRNA sequence / Transcribed locus, weakly similar to NP_001024018.1 abnormal embryonic PARtitioning of cytoplasm family member (par-1) [Caenorhabditis elegans] / similar to serine/threonine kinase FKSG82 / PREDICTED: Gallus gallus similar to serine/threonine protein kinase SSTK (LOC428720), mRNA. / PREDICTED: similar to serine/threonine protein kinase SSTK; / --- / --- / --- / ---

Gene Name	Fold Change	p-value	SEQUENCE	Array Description	Blast/Database Description
RIGG12944	0.461	0.0133	CGACAAGGTGCAGAAAGATGACATT GAGGTGCGGTTCTATGAGGATGACG AGAACGGCTGGCAGG		ENSGALT00000009068.1 / PRF|2006281A|741021|2006281A p50B/p97 transcription factor. {Gallus gallus} (exp=-1; wgp=-1; cg=-1), complete / Gallus gallus p50B mRNA for p50B/p97 (Lyt-10) transcription factor, complete cds / NF-kB p52/p100 (NFKB2) mRNA complete cds / NFKB2: Nuclear factor NF-kappa-B p100 subunit / Gallus gallus nuclear factor of kappa light polypeptide gene enhancer in B-cells 2 (p49/p100) (NFKB2), mRNA. / nuclear factor of kappa light polypeptide gene enhancer in B-cells 2 (p49/p100); / NF-kB p52/p100 (NFKB2) mRNA complete cds; / --- / Gallus gallus p50B mRNA for p50B/p97 (Lyt-10) transcription factor, complete cds / NF-kB p52/p100 (NFKB2) mRNA complete cds
RIGG11102	0.46	0.014	ACGCACGTGAGAAAGTACTGAAGGC AGTGGCTGTGCTGATGGAGTTCTGC AGGGAGCGCTTCCGC		ENSGALT00000003874.1 / weakly similar to UP|SIL1_HUMAN (Q9H173) Nucleotide exchange factor SIL1 precursor (BiP-associated protein) (BAP), partial (63%) / --- / --- / similar to SIL1 protein / PREDICTED: Gallus gallus similar to endoplasmic reticulum chaperone SIL1, homolog of yeast; BiP-associated protein (LOC416185), mRNA. / PREDICTED: similar to endoplasmic reticulum chaperone SIL1, homolog of yeast; BiP-associated protein; / --- / --- / --- / ---
RIGG19827	0.458	0.0463	ATGACGAGCGAGGTGGTGGAGAAT GAGTTTGAGTTTTACTCCAAGGCAG AGAAGTACTGGAAGGATGTGC		ENSGALT00000028312.1 / Gallus gallus finished cDNA, clone ChEST56a22 / 603597664F1 CSEQCHN73 Gallus gallus cDNA clone ChEST567o16 5', mRNA sequence / Finished cDNA, clone ChEST56a22 / --- / PREDICTED: Gallus gallus similar to RIKEN cDNA 2610205E22 (LOC425539), partial mRNA. / PREDICTED: similar to RIKEN cDNA 2610205E22, partial; / --- / --- / 603597664F1 CSEQCHN73 Gallus gallus cDNA clone ChEST567o16 5', mRNA sequence / Finished cDNA, clone ChEST56a22
RIGG06661	0.457	0.0417	GTTGGGTTGGATGCAAATGGTTTCT TTCTGGTCATGTCTTTGTTGGAATG CTGAAGCAGTGAGGAGGTGC	Genome Hit Contig1178.2	Genome Hit Contig1178.2 / Gallus gallus finished cDNA, clone ChEST924b24 / 603606625F1 CSEQCHN55 Gallus gallus cDNA clone ChEST589b7 5', mRNA sequence / Finished cDNA, clone ChEST924b24 / --- / --- / --- / --- / --- / 603606625F1 CSEQCHN55 Gallus gallus cDNA clone ChEST589b7 5', mRNA sequence / Finished cDNA, clone ChEST924b24
RIGG19235	0.456	0.0469	ATACTGAGAAGGAATTGCTTGGAAC TTACATCTGCCAAGGCAAGGAGCTT ATTCCACTTGGGGAGGCGTT		ENSGALT00000026829.1 / weakly similar to UP|Q6GLH7_XENTR (Q6GLH7) MGC69346 protein (PDLIM1 interacting kinase 1 like), partial (88%) / Gallus gallus mRNA for hypothetical protein, clone 18m15 / Similar to BC027088 protein (LOC426385), mRNA / similar to serine/threonine kinase 35; CLP-36 interacting kinase / --- / --- / --- / --- / Gallus gallus mRNA for hypothetical protein, clone 18m15 / Similar to BC027088 protein (LOC426385), mRNA

Gene Name	Fold Change	p-value	SEQUENCE	Array Description	Blast/Database Description
RIGG20114	0.456	0.00734	GAAGAAGAGGAAATTAACAGCTAAAAGGAAGCGTGAAGAGGGCTCTGATATGGAAGATGAGGACATGGAAG	Gallus gallus similar to DEAD (Asp-Glu-Ala-Asp) box polypeptide 55 (LOC416820), mRNA	Gallus gallus similar to DEAD (Asp-Glu-Ala-Asp) box polypeptide 55 (LOC416820), mRNA / RF|NP_001006185.1|57525164|NM_001006185 DEAD (Asp-Glu-Ala-Asp) box polypeptide 55 {Gallus gallus} (exp=-1; wgp=0; cg=0), complete / Gallus gallus mRNA for hypothetical protein, clone 5g4 / DEAD (Asp-Glu-Ala-Asp) box polypeptide 55 (DDX55), mRNA / DDX55, RCJMB04_5g4: ATP-dependent RNA helicase DDX55 / PREDICTED: Gallus gallus similar to DEAD (Asp-Glu-Ala-Asp) box polypeptide 55 (LOC416820), mRNA. / PREDICTED: similar to DEAD (Asp-Glu-Ala-Asp) box polypeptide 55; / DEAD (Asp-Glu-Ala-Asp) box polypeptide 55 (DDX55), mRNA; / --- / Gallus gallus mRNA for hypothetical protein, clone 5g4 / DEAD (Asp-Glu-Ala-Asp) box polypeptide 55 (DDX55), mRNA
RIGG08465	0.455	0.0299	CTTACATGTTTACACCTGGCTGCTGAGGGAGGTCACATTGACTGTGTAAAGTTGCTCTTGGAAGCAGGTG		ENSGALG00000007252.1 / similar to UP|Q495B2_HUMAN (Q495B2) ANKDD1A protein, partial (28%) / 603738814F1 CSEQCHN56 Gallus gallus cDNA clone ChEST633k3 5', mRNA sequence / Transcribed locus, strongly similar to XP_413894.1 PREDICTED: hypothetical protein XP_413894 [Gallus gallus] / / PREDICTED: Gallus gallus similar to hypothetical protein LOC348094 (LOC415525), mRNA. / PREDICTED: hypothetical protein XP_413894; / Transcribed locus, strongly similar to XP_413894.1 PREDICTED: hypothetical protein XP_413894 [Gallus gallus]; / --- / --- / ---
RIGG11872	0.452	0.0317	CCTTCAAGGTCCACAAGTTTTCCCTATAACCCGAGTTCCAATTTCTGAGTTCTGTCAAATTTAGAGGAAGG		ENSGALT00000006015.1 / --- / --- / --- / --- / --- / --- / --- / --- / --- / ---
RIGG02013	0.445	0.0221	GGTCAAAGACTTCACAGAAGAAAGGCACACCAAGAAAAGGTCAGAGGCCAAAAGCAATAGAAGTTGGTCT	Similar to Q8TEB4 (Q8TEB4) Hypothetical protein FLJ23706	Similar to Q8TEB4 (Q8TEB4) Hypothetical protein FLJ23706 / Gallus gallus finished cDNA, clone ChEST272b7 / gPGC_EST01775 Embryonic gonadal PGC cDNA Library Gallus gallus cDNA 5', mRNA sequence / Finished cDNA, clone ChEST272b7 / --- / PREDICTED: Gallus gallus similar to BCL-6 corepressor long isoform (LOC418574), mRNA. / PREDICTED: similar to BCL-6 corepressor long isoform; / Transcribed locus, weakly similar to XP_521009.1 PREDICTED: similar to BCL-6 corepressor long isoform [Pan troglodytes]; / --- / --- / ---
RIGG12248	0.443	0.0302	GGGCTAACATCCATGCAGAAAATGCCACATCACTCTTGCCAGCTGTAGAAAATGTCACTTTAAGTGCACT		ENSGALT00000007073.1 / Gallus gallus finished cDNA, clone ChEST869c14 / Gallus gallus finished cDNA, clone ChEST869c14 / Finished cDNA, clone ChEST869c14 / / --- / --- / Finished cDNA, clone ChEST869c14; / solute carrier family 13 (sodium-dependent dicarboxylate transporter), member 3; / Gallus gallus finished cDNA, clone ChEST869c14 / Finished cDNA, clone ChEST869c14

Gene Name	Fold Change	p-value	SEQUENCE	Array Description	Blast/Database Description
RIGG11611	0.442	0.0108	CGTGTACAGCGCTGCCATCCTCGAGTATCTCACTGCTGAGGTGGGTCCTGGAGTTGGCAGGCAAC		ENSGALT00000005303.1 / GB|CAA32094.1|63445|GGH2AF histone H2A.F {Gallus gallus} (exp=-1; wgp=0; cg=0), partial (98%) / --- / --- / --- / PREDICTED: Gallus gallus similar to H2A histone family, member Z (LOC426361), partial mRNA. / PREDICTED: similar to H2A histone family, member Z, partial; / --- / --- / --- / ---
RIGG09186	0.441	0.00133	ACATGGTGATGAAGAAATGCACAACTCTGGAAGAAATTAAGGGCATGAGTCAGTGGGAGCAAAGCATTAC		ENSGALG00000013051.1 / --- / --- / --- / --- / --- / --- / --- / --- / --- / ---
RIGG07235	0.44	0.037	ATAGGAAGGGAAGGCTAGCTGACAACCGTGTAGTTATATTTGTGCTACAACCCAAACTGCATAATTGTTT	Genome Hit Contig15.191	Genome Hit Contig15.191 / --- / Gallus gallus finished cDNA, clone ChEST996n12 / Finished cDNA, clone ChEST996n12 / --- / --- / --- / --- / --- / Gallus gallus finished cDNA, clone ChEST996n12 / Finished cDNA, clone ChEST996n12
RIGG08415	0.438	0.0328	TCGTCTTTGCGGAGGTCAATGCTGACGTGGTGTACTGGATCGTTCAGAGTGACGGTGAGAAGAAG	Protein-glutamine gamma-glutamyltransferase (EC 2.3.2.13) (Tissue transglutaminase) (TGase C) (TGC) (TG(C)) (Tranglutaminase 2). [Source:SWISSPROT;Acc:Q01841]	Protein-glutamine gamma-glutamyltransferase (EC 2.3.2.13) (Tissue transglutaminase) (TGase C) (TGC) (TG(C)) (Tranglutaminase 2). [Source:SWISSPROT / RF|NP_990779.1|45382075|NM_205448 transglutaminase 2 (C polypeptide, protein-glutamine-gamma-glutamyltransferase) {Gallus gallus} (exp=-1; wgp=0; cg=0), complete / Gallus gallus transglutaminase 2 (C polypeptide, protein-glutamine-gamma-glutamyltransferase) (TGM2), mRNA / Transglutaminase / TGL2: Protein-glutamine gamma-glutamyltransferase 2 / Gallus gallus transglutaminase (LOC396432), mRNA. / --- / Transglutaminase; / --- / Gallus gallus transglutaminase 2 (C polypeptide, protein-glutamine-gamma-glutamyltransferase) (TGM2), mRNA / Transglutaminase
RIGG00033	0.433	0.0244	TCCAGAGCCGTATACCAGAGGATAGCAGAGATAGAAGATCTACCACCTCTTTATACACTCAACAGACCTTTACTT	adenosine deaminase RNA-specific B1 (RED1 homolog rat) [Gallus gallus]. [Source:RefSeq;Acc:NM_204240]	adenosine deaminase RNA-specific B1 (RED1 homolog rat) [Gallus gallus]. [Source:RefSeq / RF|NP_989571.1|45383650|NM_204240 adenosine deaminase, RNA-specific, B1 (RED1 homolog rat) {Gallus gallus} (exp=-1; wgp=0; cg=0), complete / Gallus gallus adenosine deaminase mRNA, complete cds, alternatively spliced / Adenosine deaminase mRNA, complete cds, alternatively spliced / Adenosine deaminase / Gallus gallus adenosine deaminase, RNA-specific, B1 (RED1 homolog rat) (ADARB1), mRNA. / adenosine deaminase, RNA-specific, B1 (RED1 homolog rat); / --- / --- / Gallus gallus adenosine deaminase mRNA, complete cds, alternatively spliced / Adenosine deaminase mRNA, complete cds, alternatively spliced
RIGG04995	0.429	0.00988	TCCACCAAATCTGAAGAGGACAAAGAAAATGCAAGGCAGGCAAGGAGACATCAGGAGCTCTAGATACAAG	Weakly similar to Q9H9G9 (Q9H9G9) Hypothetical protein FLJ12760	Weakly similar to Q9H9G9 (Q9H9G9) Hypothetical protein FLJ12760 / similar to RF|NP_728647.2|28574997|NM_167907 CG13929-PA, isoform A {Drosophila melanogaster} (exp=-1; wgp=0; cg=0), partial (30%) / Gallus gallus finished cDNA, clone ChEST698n20 / Finished cDNA, clone ChEST698n20 / --- / PREDICTED: Gallus gallus similar to BC004636 protein (LOC424154), mRNA. / PREDICTED: similar to BC004636 protein; / --- / --- / Gallus gallus finished cDNA, clone ChEST698n20 / Finished cDNA, clone ChEST698n20

Gene Name	Fold Change	p-value	SEQUENCE	Array Description	Blast/Database Description
RIGG04335	0.428	0.0477	ATGTCCAGCACAACAGATTTTATCA CGAGGAGCTAAATGCACCGGTGAG AAGGAATAAAGAAGAGCCAAA	Similar to Q9NT39 (Q9NT39) Hypothetical protein (Fragment)	Similar to Q9NT39 (Q9NT39) Hypothetical protein (Fragment) / homologue to UP|CI078_HUMAN (Q9NZ63) Protein C9orf78 (Hepatocellular carcinoma-associated antigen 59), partial (76%) / 603138036F1 CSEQCHL25 Gallus gallus cDNA clone ChEST125g10 5', mRNA sequence / Finished cDNA, clone ChEST617p10 / --- / PREDICTED: Gallus gallus similar to chromosome 9 open reading frame 78; hepatocellular carcinoma-associated antigen 59 (LOC417189), mRNA. / PREDICTED: similar to chromosome 9 open reading frame 78; hepatocellular carcinoma-associated antigen 59; / Finished cDNA, clone ChEST617p10; / --- / 603138036F1 CSEQCHL25 Gallus gallus cDNA clone ChEST125g10 5', mRNA sequence / Finished cDNA, clone ChEST617p10
RIGG11947	0.421	0.034	TCTCGGAAGTGCAGAGCATACAGGA AGCTCAGTTAAAAGACTCTCTGCCT GAACATAAGGACTCAGCCAG		ENSGALT00000006246.1 / homologue to GB|AAA59964.2|13249985|HUMOCRL Lowe oculocerebrorenal syndrome protein {Homo sapiens} (exp=-1; wgp=0; cg=0), partial (27%) / --- / --- / similar to ocrl1 / --- / --- / Finished cDNA, clone ChEST497l12; / --- / AJ451117 riken1 Gallus gallus cDNA clone 27k6r1, mRNA sequence / Finished cDNA, clone ChEST497l12
RIGG15772	0.42	0.00528	AGCCATGAGATTTGCAATGAGGTCG CTGATGAGGTGACAACTATGGACAA CTTGATTGCTGAACTCCAGG		ENSGALT00000017153.1 / Gallus gallus finished cDNA, clone ChEST21e10 / Gallus gallus mRNA for hypothetical protein, clone 15c1 / Tetratricopeptide repeat domain 27 (TTC27), mRNA / RCJMB04_15c1: Hypothetical protein / PREDICTED: Gallus gallus similar to hypothetical protein FLJ20272 (LOC421462), mRNA. / PREDICTED: similar to hypothetical protein FLJ20272; / Tetratricopeptide repeat domain 27 (TTC27), mRNA; / --- / Gallus gallus mRNA for hypothetical protein, clone 15c1 / Tetratricopeptide repeat domain 27 (TTC27), mRNA
RIGG00960	0.42	0.00723	CACAGAGCATGCACATGTTATGTAT GTGTAACAGCACTGTGCACATGACC ACAGCCCCGTGCATG	Genome Hit Contig50.13	Genome Hit Contig50.13 / Gallus gallus finished cDNA, clone ChEST122l16 / Gallus gallus finished cDNA, clone ChEST122l16 / Finished cDNA, clone ChEST122l16 / --- / --- / --- / --- / --- / Gallus gallus finished cDNA, clone ChEST122l16 / Finished cDNA, clone ChEST122l16
RIGG08095	0.418	0.0496	GTCAGTTCGTTTGGATCCTGTCTTC AGCATGGTATCTCAAACAACATGGA TTTATCCCTGAGTGAGAAGC		ENSGALG00000004515.1 / --- / --- / --- / --- / PREDICTED: Gallus gallus similar to Kinetochore-associated protein 1 (Rough deal homolog) (hRod) (HsROD) (Rod) (LOC416866), mRNA. / PREDICTED: similar to Kinetochore-associated protein 1 (Rough deal homolog) (hRod) (HsROD) (Rod); / --- / --- / --- / ---
RIGG19510	0.417	0.0437	AATGATGCTTCAAGTAGATGTAGAT CTCCGATTCTCAGCCTTGCAAGTTC TTGAACATCCATGGGTTAAT		ENSGALT00000027554.1 / --- / Gallus gallus finished cDNA, clone ChEST916a14 / Finished cDNA, clone ChEST916a14 / / --- / --- / --- / doublecortin and CaM kinase-like 1; / --- / ---

Gene Name	Fold Change	p-value	SEQUENCE	Array Description	Blast/Database Description
RIGG08773	0.412	0.0387	TAAAGAAAGAGACTCCAAACACAGC AGACATGATGAGAAGAGAAGATCAA GAAGCAAAGAGAGGGACCGT		ENSGALG00000009839.1 / weakly similar to UP|PPIG_HUMAN (Q13427) Peptidyl-prolyl cis-trans isomerase G (Peptidyl-prolyl isomerase G) (PPIase G) (Rotamase G) (Cyclophilin G) (Clk-associating RS-cyclophilin) (CARS-cyclophilin) (CARS-Cyp) (SR-cyclophilin) (SRcyp) (SR-cyp) (CASP10) , partial (15%) / --- / --- / --- / PREDICTED: Gallus gallus similar to peptidyl-prolyl isomerase G (cyclophilin G); Clk-associating RS-cyclophilin (LOC424162), mRNA. / PREDICTED: similar to peptidyl-prolyl isomerase G (cyclophilin G); Clk-associating RS-cyclophilin; / --- / --- / --- / ---
RIGG00584	0.409	0.0493	AGATGGTCTCAGTATTGCAATATTGT GTGTGTTTGTTTCTGTTATGCCAGC AGAGTTTTATCACGGTGAG	Weakly similar to Q9DDJ7 (Q9DDJ7) Retinoblastoma tumor suppressor (Fragment)	Weakly similar to Q9DDJ7 (Q9DDJ7) Retinoblastoma tumor suppressor (Fragment) / Gallus gallus finished cDNA, clone ChEST1009j1 / --- / --- / --- / --- / --- / --- / --- / --- / ---
RIGG02167	0.404	0.0456	TGCTATCAGAACTTTGGTGTTACAG CACTGTACTTTGAGGAAAGATAGGC AGTGAAGGTCTGTAAGAGAAA	Genome Hit Contig77.43	Genome Hit Contig77.43 / Gallus gallus finished cDNA, clone ChEST299j6 / Gallus gallus finished cDNA, clone ChEST299j6 / Finished cDNA, clone ChEST299j6 / --- / --- / --- / --- / --- / Gallus gallus finished cDNA, clone ChEST299j6 / Finished cDNA, clone ChEST299j6
RIGG00930	0.4	0.0217	CATAAAGAGCATGACCGACAGCAGT GTGTACTTCAAGAGCATTGACAGTC TGCTCAAGCATGCCATTGCC	Weakly similar to Q96FH0 (Q96FH0) Similar to RIKEN cDNA 2310045N01 gene	Weakly similar to Q96FH0 (Q96FH0) Similar to RIKEN cDNA 2310045N01 gene / Gallus gallus finished cDNA, clone ChEST1021b13 / 603488301F1 CSEQCHN62 Gallus gallus cDNA clone ChEST386b2 5', mRNA sequence / Finished cDNA, clone ChEST566h1 / --- / PREDICTED: Gallus gallus similar to putative nuclear protein of bilaterial origin (16.8 kD) (1P391) (LOC426622), mRNA. / PREDICTED: similar to putative nuclear protein of bilaterial origin (16.8 kD) (1P391); / --- / --- / 603543850F1 CSEQCHN61 Gallus gallus cDNA clone ChEST517c14 5', mRNA sequence / Finished cDNA, clone ChEST964p20
RIGG17212	0.398	0.023	TGTCATTCCCAAGTCTGACAAACAG CAACGCATTAAGGAAAACATGCAGG TGTTTGACTTCGAGCTGTCT	aldo-keto reductase [Gallus gallus]. [Source:RefSeq;Acc:NM_204629]	aldo-keto reductase [Gallus gallus]. [Source:RefSeq / UP|Q90W83_CHICK (Q90W83) Aldo-keto reductase , complete / Gallus gallus aldo-keto reductase family 1, member B10 (aldose reductase) (AKR1B10), mRNA / Aldo-keto reductase (akr gene) / akr: Aldo-keto reductase / Gallus gallus aldo-keto reductase (AKR), mRNA. / aldo-keto reductase; / Aldo-keto reductase (akr gene); / --- / Gallus gallus aldo-keto reductase family 1, member B10 (aldose reductase) (AKR1B10), mRNA / Aldo-keto reductase (akr gene)
RIGG01486	0.397	0.00951	AAGGCAATGTAGACACAGCAGTTTA AAAGGAAACAGATTAAAGATGGTCA TGTGAGTACAGCTGGGTCCTA	Contig Hit 034352.1	Contig Hit 034352.1 / --- / Gallus gallus finished cDNA, clone ChEST203p3 / Finished cDNA, clone ChEST203p3 / --- / --- / --- / --- / --- / Gallus gallus finished cDNA, clone ChEST203p3 / Finished cDNA, clone ChEST203p3

Gene Name	Fold Change	p-value	SEQUENCE	Array Description	Blast/Database Description
RIGG17941	0.392	0.000774	GATGGAGCTGAGTGATGACAAGTCTGAGCATGCTGTCAGTGTGGCCACGGTGGATCTGCTGCAGG		ENSGALT00000023282.1 / similar to UP|Q6NRG4_XENLA (Q6NRG4) MGC83833 protein, partial (66%) / naw04b01.y1 Chicken eye (hatched). Unnormalized (naw) Gallus gallus cDNA clone naw04b01 5', mRNA sequence / Transcribed locus, strongly similar to XP_428522.1 PREDICTED: similar to lethal giant larvae homolog 1; Lethal giant larvae (Drosophila) homolog 1, partial [Gallus gallus] / --- / --- / --- / --- / lethal giant larvae homolog 1 (Drosophila); / naw04b01.y1 Chicken eye (hatched). Unnormalized (naw) Gallus gallus cDNA clone naw04b01 5', mRNA sequence / Transcribed locus, strongly similar to XP_428522.1 PREDICTED: similar to lethal giant larvae homolog 1; Lethal giant larvae (Drosophila) homolog 1, partial [Gallus gallus]
RIGG17790	0.39	0.0021	AGTATCTGTTTATCTTTTGGGTGCTTTGTATTCATTGTTGTGTCCTATGTGCAGATCTTCAGGGCCGTGC		ENSGALT00000022869.1 / --- / Gallus gallus finished cDNA, clone ChEST533c24 / Finished cDNA, clone ChEST533c24 / similar to Olfactory receptor 5U1 (Hs6M1-28), partial / PREDICTED: Gallus gallus similar to Olfactory receptor 1F12 (Hs6M1-35P) (LOC430065), partial mRNA. / PREDICTED: similar to Olfactory receptor 1F12 (Hs6M1-35P), partial;PREDICTED: similar to Olfactory receptor 5BF1, partial; / --- / --- / --- / ---
RIGG04093	0.383	0.0201	AAGGTCTTTATGCTGAGGTTCTGCTGTGTCTTACTTCCCTTTCTGGTCTCTTTTCAGGGTTTTGTATGGG	Genome Hit Contig7.63	Genome Hit Contig7.63 / similar to UP|Q5LXN5_STRT1 (Q5LXN5) Acetyltransferase, GNAT family, partial (11%) / Gallus gallus finished cDNA, clone ChEST583p18 / Finished cDNA, clone ChEST583p18 / --- / --- / --- / --- / --- / Gallus gallus finished cDNA, clone ChEST583p18 / Finished cDNA, clone ChEST583p18
RIGG05211	0.381	0.00987	AACCCACACAGCAATGCAATGTTATCAGTATTGACCACAAGCCTGTAACATTTCTACACATGCTAATGTC	Similar to Q9D520 (Q9D520) 2610318G18Rik protein	Similar to Q9D520 (Q9D520) 2610318G18Rik protein / Gallus gallus finished cDNA, clone ChEST72i21 / Gallus gallus finished cDNA, clone ChEST72i21 / Finished cDNA, clone ChEST94h8 / --- / PREDICTED: Gallus gallus similar to RIKEN cDNA 2610318G18 (LOC421151), mRNA. / PREDICTED: similar to RIKEN cDNA 2610318G18; / Finished cDNA, clone ChEST94h8; / --- / Gallus gallus finished cDNA, clone ChEST72i21 / Finished cDNA, clone ChEST94h8
RIGG01062	0.381	0.0457	TCTGTGGTGGCGAGGATTGAAGGATTCCTGCTGTATGTCCTGCATTATGGGAAGGAGGGGATTTCTTAAT	Weakly similar to Q7ZXM2 (Q7ZXM2) Hypothetical protein	Weakly similar to Q7ZXM2 (Q7ZXM2) Hypothetical protein / Gallus gallus finished cDNA, clone ChEST142h11 / Gallus gallus finished cDNA, clone ChEST142h11 / Finished cDNA, clone ChEST360m16 / RCJMB04_6f1: Hypothetical protein / --- / --- / --- / --- / Gallus gallus finished cDNA, clone ChEST142h11 / Finished cDNA, clone ChEST360m16

Gene Name	Fold Change	p-value	SEQUENCE	Array Description	Blast/Database Description
RIGG20391	0.38	0.00183	TTGGAGAACTTACATGAGGTCTTGG AGATCAAATTCAAAGGCATTGACAG TGAAGATGACTATGGCCGTG	Transforming growth factor beta 3 precursor (TGF-beta 3). [Source:SWISSPROT;Acc:P16047]	Transforming growth factor beta 3 precursor (TGF-beta 3). [Source:SWISSPROT / RF|NP_990785.1|45382047|NM_205454 transforming growth factor, beta 3 {Gallus gallus} (exp=-1; wgp=0; cg=0), complete / Gallus gallus transforming growth factor, beta 3 (TGFB3), mRNA / Transforming growth factor beta (TGF-beta-3) / TGFB3: Transforming growth factor beta-3 precursor / --- / transforming growth factor beta (5' end could be at 109); / --- / --- / Gallus gallus transforming growth factor, beta 3 (TGFB3), mRNA / Transforming growth factor beta (TGF-beta-3)
RIGG19486	0.379	0.0138	CCCACGGGTTAATATCAGAATATATT ACTGAAGAGCTGAGTAAGGAGCTGT CAAAATACCTAGGTCTCCCAGAA		ENSGALT00000027483.1 / Gallus gallus finished cDNA, clone ChEST333k20 / --- / --- / --- / PREDICTED: Gallus gallus similar to FLJ11712 protein (LOC418874), mRNA. / PREDICTED: similar to FLJ11712 protein; / --- / --- / Gallus gallus mRNA for hypothetical protein, clone 13a19 / Deleted in lymphocytic leukemia 8 (DLEU8), mRNA
RIGG08979	0.373	0.002	TGATAGTCATCAGTCTGACTCTGAC GACTACTCTGAGCTGAGCATAGAAG ATGATGAAGAAGGGAAGGTG		ENSGALG00000011368.1 / similar to UP|Q86TU1_HUMAN (Q86TU1) Full-length cDNA clone CS0DC008YJ16 of Neuroblastoma of Homo sapiens (human) (Fragment), partial (21%) / esa022_g09 Eimeiria tenella-infected caecal tonsil Gallus gallus cDNA, mRNA sequence / Transcribed locus, weakly similar to XP_918760.1 PREDICTED: hypothetical protein LOC72805 [Mus musculus] / hypothetical protein / --- / --- / Transcribed locus, weakly similar to XP_918760.1 PREDICTED: hypothetical protein LOC72805 [Mus musculus]; / --- / 603108742F1 CSEQCHN04 Gallus gallus cDNA clone ChEST52c11 5', mRNA sequence / Transcribed locus, weakly similar to XP_576102.2 PREDICTED: hypothetical protein [Rattus norvegicus]
RIGG18276	0.37	0.0343	AAATGTTGCAGATCACAAGAAAGGA GCAAAGAAAGCTAGGATAGAGGAAG ACAAGAAAGAGACAGAGGAC	Histone deacetylase 2 (HD2). [Source:SWISSPROT;Acc:P56519]	Histone deacetylase 2 (HD2). [Source:SWISSPROT / UP|HDAC2_CHICK (P56519) Histone deacetylase 2 (HD2), complete / Gallus gallus histone deacetylase 2 (HDAC2), mRNA / Histone deacetylase-2 / HDAC2: Histone deacetylase 2 / Gallus gallus histone deacetylase-2 (LOC395635), mRNA. / histone deacetylase-2; / Histone deacetylase-2; / --- / Gallus gallus histone deacetylase 2 (HDAC2), mRNA / Histone deacetylase-2

Gene Name	Fold Change	p-value	SEQUENCE	Array Description	Blast/Database Description
RIGG00751	0.364	0.0263	AGAACGTGATGAGTGGATTGAGGCAATAAGGACTAGCATCACACAGAATCCTTTCTATGATCTCGTCTCT	Similar to BAA87927 (BAA87927) Cytohesin-2	Similar to BAA87927 (BAA87927) Cytohesin-2 / RF|NP_001012833.1|61098043|NM_001012815 pleckstrin homology, Sec7 and coiled-coil domains 4 {Gallus gallus} (exp=-1; wgp=0; cg=0), complete / Gallus gallus mRNA for hypothetical protein, clone 26f20 / Pleckstrin homology, Sec7 and coiled-coil domains 4 (PSCD4), mRNA / RCJMB04_26f20: Hypothetical protein / PREDICTED: Gallus gallus similar to Cytohesin 4 (LOC418043), mRNA. / PREDICTED: similar to Cytohesin 4; / Pleckstrin homology, Sec7 and coiled-coil domains 4 (PSCD4), mRNA; / --- / Gallus gallus mRNA for hypothetical protein, clone 26f20 / Pleckstrin homology, Sec7 and coiled-coil domains 4 (PSCD4), mRNA
RIGG00263	0.36	0.0154	GCCACAGACTTGCACATTTTGAATCCAGAGAAGATCATTACCTTGACAAATAGCAGATGCTGTACACAAAG	Gallus gallus mRNA for hypothetical protein, clone 20d15	Gallus gallus mRNA for hypothetical protein, clone 20d15 / homologue to UP|Q2M1U9_HUMAN (Q2M1U9) Na+/H+ exchanger isoform 8, partial (98%) / Gallus gallus mRNA for hypothetical protein, clone 20d15 / Solute carrier family 9 (sodium/hydrogen exchanger), member 8 (SLC9A8), mRNA / RCJMB04_20d15: Hypothetical protein / --- / --- / --- / --- / Gallus gallus mRNA for hypothetical protein, clone 20d15 / Solute carrier family 9 (sodium/hydrogen exchanger), member 8 (SLC9A8), mRNA
RIGG04734	0.348	0.036	GCCTGCGATTTCACATTTCTGCTACACCTTGTAAACCTACACATAGCTTGATCTGTAAAGTCAGGCTTAA	Genome Hit Contig61.252	Genome Hit Contig61.252 / --- / Gallus gallus finished cDNA, clone ChEST663l17 / Finished cDNA, clone ChEST663l17 / --- / --- / --- / --- / --- / Gallus gallus finished cDNA, clone ChEST663l17 / Finished cDNA, clone ChEST663l17
RIGG04051	0.348	0.0153	AGATTGACTTTCAGCAAATGGACATAGCAGGTGATGAGGACAACAGGAAACGGATGAGAGAGAATGTTCCTGG	Weakly similar to SH3N_HUMAN (Q9H299) SH3 domain-binding glutamic acid-rich-l	Weakly similar to SH3N_HUMAN (Q9H299) SH3 domain-binding glutamic acid-rich-l / Gallus gallus finished cDNA, clone ChEST57a18 / Gallus gallus finished cDNA, clone ChEST58k10 / Finished cDNA, clone ChEST862i9 / --- / PREDICTED: Gallus gallus similar to SH3 domain-binding glutamic acid-rich protein (SH3BGR protein) (21-glutamic acid-rich protein) (21-GARP) (LOC418523), mRNA. / PREDICTED: similar to SH3 domain-binding glutamic acid-rich protein (SH3BGR protein) (21-glutamic acid-rich protein) (21-GARP); / Finished cDNA, clone ChEST862i9; / --- / Gallus gallus finished cDNA, clone ChEST58k10 / Finished cDNA, clone ChEST862i9
RIGG09287	0.348	0.0182	TCTGGAAGGGAAGACATTTTACTCCAAGAAGGACAAGCCGCTGTGCAAGAGCCATGCTTTCTCCC		ENSGALG00000014056.1 / UP|PDLI7_CHICK (Q679P3) PDZ and LIM domain protein 7 (LIM mineralization protein), complete / Gallus gallus LIM mineralizing protein (LMP) mRNA, complete cds / Hypothetical protein, clone 14g20 / PDLIM7, LMP, RCJMB04_14g20: PDZ and LIM domain protein 7 / --- / --- / --- / --- / Gallus gallus LIM mineralizing protein (LMP) mRNA, complete cds / Hypothetical protein, clone 14g20
RIGG01402	0.347	0.0262	TGATCCTCCCAATAGCACGGAATCTCACTGCGCTTCTTCTGAAGAGAGAAAGAAAGGAAGGGTCACGGAA	Weakly similar to BAC65623 (BAC65623) MKIAA0663 protein (Fragment)	Weakly similar to BAC65623 (BAC65623) MKIAA0663 protein (Fragment) / Gallus gallus finished cDNA, clone ChEST196l14 / --- / --- / --- / --- / --- / --- / --- / --- / ---

Gene Name	Fold Change	p-value	SEQUENCE	Array Description	Blast/Database Description
RIGG10743	0.342	0.0415	TTACTAATGAGAAGCCAAGTGCAATCTTTAAAATTTCTGGTTTGAAATCTGGGAAGGGAAGCCAGCACCC		ENSGALT00000002817.1 / weakly similar to UP|O60175_SCHPO (O60175) SPBC21H7.06c protein, partial (36%) / 603006451F1 CSEQCHL01 Gallus gallus cDNA clone ChEST24f13 5', mRNA sequence / Similar to hypothetical protein HSPC138 (LOC427034), mRNA / --- / PREDICTED: Gallus gallus similar to Hypothetical protein HSPC138 (LOC425181), mRNA.PREDICTED: Gallus gallus similar to hypothetical protein HSPC138 (LOC427034), partial mRNA. / PREDICTED: similar to Hypothetical protein HSPC138;PREDICTED: similar to hypothetical protein HSPC138, partial; / --- / --- / 603006451F1 CSEQCHL01 Gallus gallus cDNA clone ChEST24f13 5', mRNA sequence / Similar to hypothetical protein HSPC138 (LOC427034), mRNA
RIGG00342	0.341	0.0339	CCTCTCACCTCTGGTCTACTCGAATTTTCCTGCTGCTTTATTGCTTACTTACTTACCCATACTGCCTTGT	Gallus gallus mRNA for hypothetical protein, clone 33b23	Gallus gallus mRNA for hypothetical protein, clone 33b23 / weakly similar to UP|CP2J2_HUMAN (P51589) Cytochrome P450 2J2 (CYPIIJ2) (Arachidonic acid epoxygenase) , partial (73%) / Gallus gallus mRNA for hypothetical protein, clone 33b23 / Hypothetical protein, clone 33b23 / RCJMB04_33b23: Hypothetical protein / --- / --- / Hypothetical protein, clone 33b23; / --- / Gallus gallus mRNA for hypothetical protein, clone 33b23 / Hypothetical protein, clone 33b23
RIGG02540	0.338	0.0167	AATGGGAGCAAGGGAGACAGCAAAGCAAACAGAAGCTGGGAAAGAGACACAAAGAGAAAGCAGCCTTTTC	Genome Hit Contig62.165	Genome Hit Contig62.165 / GB|CR386851.1|CR386851.1 Gallus gallus finished cDNA, clone ChEST35m1 / Gallus gallus finished cDNA, clone ChEST35m1 / Finished cDNA, clone ChEST35m1 / --- / --- / --- / --- / --- / Gallus gallus finished cDNA, clone ChEST35m1 / Finished cDNA, clone ChEST35m1
RIGG03818	0.335	0.0269	CTGGCAAGCGAGCGTGTTTAAAAGGAGGCCAGTGAAAATGCAGTCCCTCTTGAATTGCATAATAGAGAGA	Weakly similar to AAH05645 (AAH05645) Ets variant gene 1	Weakly similar to AAH05645 (AAH05645) Ets variant gene 1 / Gallus gallus finished cDNA, clone ChEST548i16 / Gallus gallus finished cDNA, clone ChEST548i16 / Finished cDNA, clone ChEST548i16 / ERM: Ets domain protein (Fragment) / PREDICTED: Gallus gallus similar to ets variant gene 5 (ets-related molecule) (LOC424840), mRNA. / PREDICTED: similar to ets variant gene 5 (ets-related molecule); / Ets domain protein (ERM); / --- / Gallus gallus finished cDNA, clone ChEST548i16 / Finished cDNA, clone ChEST548i16
RIGG08088	0.335	0.0389	CGGTGAGTGTCTCTGCTATGAGAGCTACATGAAGGACCCTGTCCATAAGCATCTCTGCATCCGGAATGAG		ENSGALG00000004472.1 / --- / --- / --- / --- / --- / --- / --- / astrotactin 1; / --- / ---

Gene Name	Fold Change	p-value	SEQUENCE	Array Description	Blast/Database Description
RIGG13985	0.333	0.0457	AAGAGAGTAAGAGGAGAGGAGCCAATGTCTTCTTACTGATCTGGAATTGTGTTCCTTTAGCCTGGCCAAG		ENSGALT00000012215.1 / --- / Gallus gallus mRNA for hypothetical protein, clone 6n2 / RAB22A, member RAS oncogene family (RAB22A), mRNA / RCJMB04_6n2: Hypothetical protein / PREDICTED: Gallus gallus similar to Rab22a protein (LOC419318), mRNA. / PREDICTED: similar to Rab22a protein; / RAB22A, member RAS oncogene family (RAB22A), mRNA; / --- / Gallus gallus mRNA for hypothetical protein, clone 6n2 / RAB22A, member RAS oncogene family (RAB22A), mRNA
RIGG08489	0.332	0.0184	CAAGGCCTTCGGCATCCGCTTTGATGTGTTGGTGTATGGCAACGCTGGGAAATTCGGCATTGTGC		ENSGALG00000007501.1 / --- / Gallus gallus purinergic receptor P2X, ligand-gated ion channel, 4 (P2RX4), mRNA / P2X ATP receptor (P2XCE) / similar to P2X3 / PREDICTED: Gallus gallus similar to P2X purinoceptor 3 (ATP receptor) (P2X3) (Purinergic receptor) (LOC428856), mRNA. / PREDICTED: similar to P2X purinoceptor 3 (ATP receptor) (P2X3) (Purinergic receptor); / --- / --- / --- / ---
RIGG13940	0.324	0.0119	ACAAGCCCAAGTTGATGAACTGAAAGGAATCATGGTTCGAAACATAGACCTTGTGGCACAGAGAGGAGAG		ENSGALT00000012085.1 / RF|NP_001026292.1|71895505|NM_001031121 synaptobrevin-like 1 {Gallus gallus} (exp=-1; wgp=0; cg=0), complete / Gallus gallus mRNA for hypothetical protein, clone 7f19 / Synaptobrevin-like 1 (SYBL1), mRNA / RCJMB04_7f19: Hypothetical protein / PREDICTED: Gallus gallus similar to Synaptobrevin-like protein 1 (LOC422297), mRNA. / --- / Synaptobrevin-like 1 (SYBL1), mRNA; / --- / Gallus gallus mRNA for hypothetical protein, clone 7f19 / Synaptobrevin-like 1 (SYBL1), mRNA
RIGG03852	0.306	0.00131	GGATACAGTGGAGCACGAAAGTCACTCAACATGTCCAGGGAAGGATGAAGGGAAATGCCCCAGTACAAAG	Weakly similar to Q9NHW2 (Q9NHW2) Flagelliform silk protein (Fragment)	Weakly similar to Q9NHW2 (Q9NHW2) Flagelliform silk protein (Fragment) / Gallus gallus finished cDNA, clone ChEST552c16 / 603590058F1 CSEQCHN74 Gallus gallus cDNA clone ChEST552c16 5', mRNA sequence / Finished cDNA, clone ChEST552c16 / --- / --- / --- / --- / --- / --- / ---
RIGG01026	0.299	0.00803	AGTGAAGATGTTGATAGAGTTTTCTTACCAGCCTTCCTAGAGCCAGTATTTCAGGAAAAGACATTGCAGC	Weakly similar to CA1A_CHICK (P08125) Collagen alpha 1(X) chain precursor	Weakly similar to CA1A_CHICK (P08125) Collagen alpha 1(X) chain precursor / Gallus gallus finished cDNA, clone ChEST1011b23 / Gallus gallus finished cDNA, clone ChEST133p18 / Finished cDNA, clone ChEST1011b23 / --- / PREDICTED: Gallus gallus similar to collagenous repeat-containing sequence of 26kDa protein (LOC427430), mRNA. / PREDICTED: similar to collagenous repeat-containing sequence of 26kDa protein; / Finished cDNA, clone ChEST1011b23; / --- / Gallus gallus finished cDNA, clone ChEST133p18 / Finished cDNA, clone ChEST1011b23
RIGG08166	0.299	0.00654	GTCTGACAGTTTCAAAGCCAAACAGATGGAAAACCATCAGCTCATAAAGGAGGCTGTGGAGATGAAATCT		ENSGALG00000005016.1 / --- / --- / --- / similar to KIAA1281 protein / PREDICTED: Gallus gallus similar to KIAA1281 protein (LOC426810), mRNA. / PREDICTED: similar to KIAA1281 protein; / --- / --- / --- / ---

Gene Name	Fold Change	p-value	SEQUENCE	Array Description	Blast/Database Description
RIGG03436	0.297	0.0185	CAATCCAGGTGAGCACAGTCAGATCGCAGCAGTATTTAGCTCTCGTTTCTGCATGTTTCTTTTCCTGCCC	Similar to Q9A7J6 (Q9A7J6) Pyruvate dehydrogenase complex, E1 componen	Similar to Q9A7J6 (Q9A7J6) Pyruvate dehydrogenase complex, E1 componen / homologue to UP|P79932_XENLA (P79932) Pyruvate dehydrogenase E1-beta subunit (Fragment), partial (50%) / Gallus gallus finished cDNA, clone ChEST131b5 / Finished cDNA, clone ChEST747b7 / --- / PREDICTED: Gallus gallus similar to Pyruvate dehydrogenase E1 component beta subunit, mitochondrial precursor (PDHE1-B) (LOC416066), mRNA. / PREDICTED: similar to Pyruvate dehydrogenase E1 component beta subunit, mitochondrial precursor (PDHE1-B); / Finished cDNA, clone ChEST747b7; / --- / --- / ---
RIGG19870	0.296	0.0384	GCTGCACTCAGTGCCTATGACATGGTTCTGGTGGAAGATAAAGAAGTGAACAGAATGCATGAAAGCCTTC		ENSGALT00000028413.1 / --- / Gallus gallus cone-type transducin alpha subunit (LOC395425), mRNA / Cone-type transducin alpha subunit / / PREDICTED: Gallus gallus similar to Guanine nucleotide-binding protein G(t), alpha-3 subunit (Gustducin alpha-3 chain) (LOC427851), mRNA.PREDICTED: Gallus gallus similar to Guanine nucleotide-binding protein G(t), alpha-3 subunit (Gustducin alpha-3 chain) (LOC430608), partial mRNA. / PREDICTED: similar to Guanine nucleotide-binding protein G(t), alpha-3 subunit (Gustducin alpha-3 chain);PREDICTED: similar to Guanine nucleotide-binding protein G(t), alpha-3 subunit (Gustducin alpha-3 chain), partial; / --- / --- / --- / ---
RIGG17329	0.295	0.0363	CACTTCAGCATCCGTGACCTGCAGATCAACGTGGAGCACTTAACTGAAAAGATGAAGACGACGGTGAAGC		ENSGALT00000021684.1 / UP|Q90YA3_CHICK (Q90YA3) 6-phosphofructokinase, partial (32%) / Gallus gallus phosphofructokinase, muscle (PFKM), mRNA / Pfk mRNA for 6-phosphofructokinase / pfk: 6-phosphofructokinase / Gallus gallus phosphofructokinase, liver (PFKL), mRNA. / liver phosphofructokinase; / Pfk mRNA for 6-phosphofructokinase; / --- / Gallus gallus phosphofructokinase, muscle (PFKM), mRNA / Pfk mRNA for 6-phosphofructokinase
RIGG00088	0.281	0.0338	GGGAGAAACCTATCACCTGTTCAGTAGCATTTGAAGTGGAAAGTGAAGCTTGAGTCATTCAGATTCTTCTG	Gallus gallus mRNA for hypothetical protein, clone 5c3	Gallus gallus mRNA for hypothetical protein, clone 5c3 / similar to UP|Q6P0A9_BRARE (Q6P0A9) Rbm35b protein, partial (85%) / Gallus gallus mRNA for hypothetical protein, clone 5c3 / RNA binding motif protein 35B (RBM35B), mRNA / RCJMB04_5c3: Hypothetical protein / PREDICTED: Gallus gallus similar to Zgc:77254 (LOC415710), partial mRNA. / PREDICTED: similar to Zgc:77254, partial; / RNA binding motif protein 35B (RBM35B), mRNA; / --- / Gallus gallus mRNA for hypothetical protein, clone 5c3 / RNA binding motif protein 35B (RBM35B), mRNA
RIGG17422	0.276	0.044	GGAGACAGAGAGGAGAGTGCTTTAAATTCTGAGCGCATGGAAAACCCAGAAGAATCCGAGCTGCCTTACG	Lymphoid transcription factor. [Source:SPTREMBL;Acc:Q9PU55]	Lymphoid transcription factor. [Source:SPTREMBL / UP|Q9PU55_CHICK (Q9PU55) Lymphoid transcription factor, complete / Gallus gallus lymphoid transcription factor (AIOLOS), mRNA / Aiolos lymphoid transcription factor / Aiolos: Lymphoid transcription factor / --- / --- / --- / --- / Gallus gallus lymphoid transcription factor (AIOLOS), mRNA / Aiolos lymphoid transcription factor

Gene Name	Fold Change	p-value	SEQUENCE	Array Description	Blast/Database Description
RIGG00656	0.272	0.028	GTGATTGTTGAGGGCAAAACAGCGGCAGCAGTAACGCAAGGTGATTCTGACAAACCAAAACAAGTTCCTC	Same gene U14555; Gallus gallus zinc-finger protein mRNA, partial cds	Same gene U14555; Gallus gallus zinc-finger protein mRNA, partial cds / Gallus gallus finished cDNA, clone ChEST1014h24 / Gallus gallus finished cDNA, clone ChEST1014h24 / Finished cDNA, clone ChEST1014h24 / ZBTB17: Zinc finger and BTB domain-containing protein 17 (Fragment) / --- / --- / --- / --- / Gallus gallus finished cDNA, clone ChEST1014h24 / Finished cDNA, clone ChEST1014h24
RIGG19608	0.263	0.0189	GAAGGCCAGAACAAAGCGAGTGAAGTCAGCTGGAGTGGTTGAAGTAAAGAAGAGTGCTGCCGATAAGTGA		ENSGALT00000027834.1 / Gallus gallus finished cDNA, clone ChEST298o19 / Gallus gallus finished cDNA, clone ChEST911k8 / Finished cDNA, clone ChEST790m6 / --- / PREDICTED: Gallus gallus similar to RIKEN cDNA 4930553M18 (LOC419002), mRNA. / PREDICTED: similar to RIKEN cDNA 4930553M18; / --- / --- / Gallus gallus finished cDNA, clone ChEST911k8 / Finished cDNA, clone ChEST790m6
RIGG00217	0.262	0.0325	TACTTGCTTCTTTGAGAAGTTAGTTCATTAAGCTTCCCCTCCAGTACTGTTGCATGGTCACAGAAGGTATTGCCT	Gallus gallus mRNA for hypothetical protein, clone 15b14	Gallus gallus mRNA for hypothetical protein, clone 15b14 / homologue to UP|BR44L_MOUSE (P63030) Brain protein 44-like protein, partial (93%) / Gallus gallus mRNA for hypothetical protein, clone 15b14 / Similar to brain protein 44-like (LOC428592), mRNA / --- / --- / --- / Similar to brain protein 44-like (LOC428592), mRNA; / --- / Gallus gallus mRNA for hypothetical protein, clone 15b14 / Similar to brain protein 44-like (LOC428592), mRNA
RIGG10913	0.257	0.0365	GAGGTGACCTTCACAGACAGGCGAGGCGAGGTGTCACAGCTGGACGAGATCTTTGTGACCGGCAG		ENSGALT00000003324.1 / similar to UP|LSM10_MOUSE (Q8QZX5) U7 snRNA-associated Sm-like protein LSm10, partial (94%) / 603213847F1 CSEQRBN13 Gallus gallus cDNA clone ChEST197m12 5', mRNA sequence / Transcribed locus, moderately similar to XP_001056695.1 PREDICTED: similar to U7 snRNP-specific Sm-like protein LSM10 [Rattus norvegicus] / / PREDICTED: Gallus gallus similar to U7 snRNP-specific Sm-like protein LSM10 (LOC419621), mRNA. / PREDICTED: similar to U7 snRNP-specific Sm-like protein LSM10; / Transcribed locus, moderately similar to XP_001056695.1 PREDICTED: similar to U7 snRNP-specific Sm-like protein LSM10 [Rattus norvegicus]; / --- / 603213847F1 CSEQRBN13 Gallus gallus cDNA clone ChEST197m12 5', mRNA sequence / Transcribed locus, moderately similar to XP_001056695.1 PREDICTED: similar to U7 snRNP-specific Sm-like protein LSM10 [Rattus norvegicus]
RIGG20419	0.255	0.047	TGTGGGTGTTCCTACTTAATTCAGCGAGCAATAGCATCCAGCACTTGGTGAAATAAGAAATTTTACTGG	similar to Myeloid differentiation primary response protein MyD88 (LOC420420), mRNA	similar to Myeloid differentiation primary response protein MyD88 (LOC420420), mRNA / RF|NP_001026133.1|71896606|NM_001030962 myeloid differentiation primary response gene (88) {Gallus gallus} (exp=-1; wgp=0; cg=0), complete / Gallus gallus mRNA for hypothetical protein, clone 14h3 / Myeloid differentiation primary response gene (88) (MYD88), mRNA / RCJMB04_14h3: Hypothetical protein / --- / --- / --- / --- / Gallus gallus mRNA for hypothetical protein, clone 14h3 / Myeloid differentiation primary response gene (88) (MYD88), mRNA

Gene Name	Fold Change	p-value	SEQUENCE	Array Description	Blast/Database Description
RIGG05222	0.248	0.048	GGACATGAGGATATCAGTAATTGGT GCTTCATTTCACAGATGTTTCCATGT GCTTTGGCTGTTTGAGTTA	Genome Hit Contig52.228	Genome Hit Contig52.228 / --- / Gallus gallus finished cDNA, clone ChEST731b13 / Finished cDNA, clone ChEST731b13 / --- / --- / --- / --- / --- / Gallus gallus finished cDNA, clone ChEST731b13 / Finished cDNA, clone ChEST731b13
RIGG13801	0.247	0.0198	GGATGATTCCCCAAGAATAGCCATG TGATGCTTAAAAGAAAGGTTCGCGT ATTTCTGCACTTGGACTCTG		ENSGALT00000011721.1 / --- / --- / --- / / --- / --- / --- / --- / --- / ---
RIGG05178	0.245	0.0348	CCCAGGAAGTCAGTTTGGATCATAA ATCTGCCAAGCCATTATGGAATCAC AGTATGGTTTGAGTAGGAAG	Genome Hit Contig18.411	Genome Hit Contig18.411 / Gallus gallus finished cDNA, clone ChEST725p5 / Gallus gallus finished cDNA, clone ChEST725p5 / Finished cDNA, clone ChEST725p5 / --- / --- / --- / --- / --- / Gallus gallus finished cDNA, clone ChEST251h16 / Finished cDNA, clone ChEST251h16
RIGG15309	0.237	0.0061	TGCTGTGCACAGACTCAAGGAGATG GAGTGTGGTGAGGTTGTGTGAGAAG CTTCCAGCCCATGAGC		ENSGALT00000015931.1 / --- / --- / --- / / --- / --- / --- / --- / --- / ---
RIGG17478	0.234	0.033	AGTCCCGCTGTGACTGCACTCTACG ATAAGCTCTATGAGAACTTCGTGGA GGAGATCGACGCCAT		ENSGALT00000022117.1 / similar to UP|Q5BKF2_XENTR (Q5BKF2) LOC594885 protein (Fragment), partial (82%) / --- / --- / --- / PREDICTED: Gallus gallus similar to MYG1 protein (LOC426187), mRNA. / PREDICTED: similar to MYG1 protein; / --- / --- / gPGC_EST09019 Embryonic gonadal PGC cDNA Library Gallus gallus cDNA 5', mRNA sequence / Finished cDNA, clone ChEST645f16
RIGG20024	0.233	0.0381	GTTAGGTGGGTGGATGGATGGATG GATGGAAGGTTGGATTGGATGGATG GATGGAGGGTTGGATTCATAG	Natural resistance-associated macrophage protein 1 (NRAMP 1). [Source:SWISSPROT;Acc:P51027]	Natural resistance-associated macrophage protein 1 (NRAMP 1). [Source:SWISSPROT / --- / --- / --- / SLC11A1, NRAMP1: Natural resistance-associated macrophage protein 1 / --- / --- / --- / --- / --- / ---
RIGG00967	0.229	0.0162	ATCTCACTCTGTAGTCTTCAGGAGA AGTTTTCCCAAATTGACATCTTGATT CTCATGACCGCAGCAGTGT	Weakly similar to Q86UI0 (Q86UI0) Phosphodiesterase 5A (EC 3.1.4.17)	Weakly similar to Q86UI0 (Q86UI0) Phosphodiesterase 5A (EC 3.1.4.17) / Gallus gallus finished cDNA, clone ChEST124i12 / Gallus gallus finished cDNA, clone ChEST124i12 / Finished cDNA, clone ChEST124i12 / / PREDICTED: Gallus gallus similar to phosphodiesterase 9A isoform a; CGMP-specific 3,5-cyclic phosphodiesterase type 9 (LOC418538), mRNA.PREDICTED: Gallus gallus similar to phosphodiesterase 9A isoform a; CGMP-specific 3,5-cyclic phosphodiesterase type 9 (LOC418539), mRNA. / PREDICTED: similar to phosphodiesterase 9A isoform a; CGMP-specific 3,5-cyclic phosphodiesterase type 9;PREDICTED: similar to phosphodiesterase 9A isoform a; CGMP-specific 3,5-cyclic phosphodiesterase type 9; / --- / --- / Gallus gallus finished cDNA, clone ChEST124i12 / Finished cDNA, clone ChEST124i12

Gene Name	Fold Change	p-value	SEQUENCE	Array Description	Blast/Database Description
RIGG19633	0.228	0.032	AGCTGCTAAATTACTGGGTTCTATG CAACAAGTGAGCTCCCATTTCTTAG AGCAGACCCTTGACAAGAAG		ENSGALT00000027915.1 / homologue to UP|Q96G32_HUMAN (Q96G32) INT4 protein, partial (72%) / Gallus gallus finished cDNA, clone ChEST634p7 / Finished cDNA, clone ChEST283h14 / similar to Integrator complex subunit 4 / PREDICTED: Gallus gallus similar to RIKEN cDNA 2610034N24 (LOC419028), partial mRNA.PREDICTED: Gallus gallus similar to RIKEN cDNA 2610034N24 (LOC426722), partial mRNA.PREDICTED: Gallus gallus similar to RIKEN cDNA 2610034N24 (LOC429595), partial mRNA. / PREDICTED: similar to RIKEN cDNA 2610034N24, partial;PREDICTED: similar to RIKEN cDNA 2610034N24, partial;PREDICTED: similar to RIKEN cDNA 2610034N24, partial; / Finished cDNA, clone ChEST283h14; / --- / Gallus gallus finished cDNA, clone ChEST634p7 / Finished cDNA, clone ChEST283h14
RIGG17589	0.224	0.0348	CAGAAGAAGGAACTGCCAAGTTGAT AGAGTTCAGTCCTCTGAGGGCAACT GATGTTAGACTTCCAAGCGG		ENSGALT00000022390.1 / RF|NP_001025728.1|71895613|NM_001030557 galactokinase 2 {Gallus gallus} (exp=-1; wgp=0; cg=0), complete / Gallus gallus mRNA for hypothetical protein, clone 1i18 / Galactokinase 2 (GALK2), mRNA / RCJMB04_1i18: Hypothetical protein / PREDICTED: Gallus gallus similar to Galk2-prov protein (LOC425150), mRNA. / PREDICTED: similar to Galk2-prov protein; / Galactokinase 2 (GALK2), mRNA; / --- / Gallus gallus mRNA for hypothetical protein, clone 1i18 / Galactokinase 2 (GALK2), mRNA
RIGG16908	0.211	0.0304	TAAGCTGTGTAAGGACTGTCAGGTG ATAGATGGGAAGAATGTGACAATCA CAGATGTTGACATTGTCTTCAGT		ENSGALT00000020444.1 / Gallus gallus finished cDNA, clone ChEST631j16 / Gallus gallus finished cDNA, clone ChEST631j16 / Finished cDNA, clone ChEST631j16 / --- / PREDICTED: Gallus gallus similar to P25 protein (LOC420800), mRNA. / PREDICTED: similar to P25 protein; / Finished cDNA, clone ChEST631j16; / --- / Gallus gallus finished cDNA, clone ChEST631j16 / Finished cDNA, clone ChEST631j16
RIGG00388	0.211	0.0461	AGGATGCTCTCGCTCTCATCAGATT AGACGACCTTTTCTTAGAATCATTTG AAGTTACGGATGTCAAACCT	Gallus gallus mRNA for hypothetical protein, clone 3n12	Gallus gallus mRNA for hypothetical protein, clone 3n12 / similar to UP|Q53G13_HUMAN (Q53G13) Putatative 28 kDa protein variant (Fragment), partial (80%) / gPGC_EST06337 Embryonic gonadal PGC cDNA Library Gallus gallus cDNA 5', mRNA sequence / Hypothetical protein, clone 3n12 / RCJMB04_3n12: Hypothetical protein / --- / --- / --- / --- / gPGC_EST06337 Embryonic gonadal PGC cDNA Library Gallus gallus cDNA 5', mRNA sequence / Hypothetical protein, clone 3n12
RIGG11878	0.198	0.00524	TGCGCATAAAGTGGCCAAGTTCTGC TATGCTGACAAGGACCTCATTAACA AAGCCATTAATGCTGCCGTG		ENSGALT00000006036.1 / Gallus gallus finished cDNA, clone ChEST75k6 / Gallus gallus finished cDNA, clone ChEST75k6 / Finished cDNA, clone ChEST75k6 / similar to Ba1-651 / PREDICTED: Gallus gallus similar to aldehyde dehydrogenase 4 family, member A1 (LOC419467), mRNA. / PREDICTED: similar to aldehyde dehydrogenase 4 family, member A1; / Finished cDNA, clone ChEST75k6; / --- / Gallus gallus finished cDNA, clone ChEST75k6 / Finished cDNA, clone ChEST75k6

Gene Name	Fold Change	p-value	SEQUENCE	Array Description	Blast/Database Description
RIGG06435	0.196	0.0348	AAGAAGTTCGCTGAGAACCAACCCA AGAAGGACAGCGTGAAGAAGGAGA GGAAGGAGAAGGAAAAGGAAA	Homologue of AAP35323 (AAP35323) Eukaryotic translation elongation facto	Homologue of AAP35323 (AAP35323) Eukaryotic translation elongation facto / Gallus gallus finished cDNA, clone ChEST895d8 / gPGC_EST04368 Embryonic gonadal PGC cDNA Library Gallus gallus cDNA 5', mRNA sequence / Eukaryotic translation elongation factor 1 / Eukaryotic translation elongation factor 1 / --- / --- / --- / --- / gPGC_EST04368 Embryonic gonadal PGC cDNA Library Gallus gallus cDNA 5', mRNA sequence / Eukaryotic translation elongation factor 1
RIGG17054	0.195	0.00607	TGCCTTGCAAATACTGGTTTGTAAC CTTCCATATGTGAACCAAAACTGGT GATGCCTCAAGTAATACAGT		ENSGALT00000020851.1 / Gallus gallus finished cDNA, clone ChEST186f11 / gPGC_EST01625 Embryonic gonadal PGC cDNA Library Gallus gallus cDNA 5', mRNA sequence / Finished cDNA, clone ChEST1026d3 / --- / PREDICTED: Gallus gallus similar to Multisynthetase complex auxiliary component p18 (LOC420865), mRNA. / PREDICTED: similar to Multisynthetase complex auxiliary component p18; / Finished cDNA, clone ChEST1026d3; / --- / gPGC_EST01625 Embryonic gonadal PGC cDNA Library Gallus gallus cDNA 5', mRNA sequence / Finished cDNA, clone ChEST1026d3
RIGG07084	0.195	0.0275	CAGACCTCAGCATTTGTAATTTGAG AGAGGAGACGTACCCAGTACTTCAG CTTGTTTCTCTGCAAAAGAAA	Contig Hit 504063.2	Contig Hit 504063.2 / Gallus gallus finished cDNA, clone ChEST983n10 / gPGC_EST02022 Embryonic gonadal PGC cDNA Library Gallus gallus cDNA 5', mRNA sequence / Finished cDNA, clone ChEST983n10 / --- / --- / --- / --- / --- / gPGC_EST02022 Embryonic gonadal PGC cDNA Library Gallus gallus cDNA 5', mRNA sequence / Finished cDNA, clone ChEST983n10
RIGG13807	0.182	0.024	CCAATATGGCTATTCCTTTGTCTTAG CCTGGATTGCATTTGCCTTTACTCT GATCAGTGGTGTTATGTACCTAG		ENSGALT00000011753.1 / Gallus gallus finished cDNA, clone ChEST64i18 / gonad_EST09169 Embryonic gonad cDNA Library Gallus gallus cDNA 5', mRNA sequence / Finished cDNA, clone ChEST69d9 / / PREDICTED: Gallus gallus similar to Epithelial membrane protein-2 (EMP-2) (XMP protein) (LOC416636), mRNA. / PREDICTED: similar to Epithelial membrane protein-2 (EMP-2) (XMP protein); / --- / --- / gonad_EST09169 Embryonic gonad cDNA Library Gallus gallus cDNA 5', mRNA sequence / Finished cDNA, clone ChEST69d9
RIGG05135	0.171	0.0117	TGGGGAAGGGTAAGGAATGATGGA AACTGTGTTAAGTGTTTCAGCAGCC TATTGTGTCTCAAAATGCATC	Weakly similar to Q8WY00 (Q8WY00) Histamine H3 receptor isoform 3	Weakly similar to Q8WY00 (Q8WY00) Histamine H3 receptor isoform 3 / Gallus gallus finished cDNA, clone ChEST718e20 / Gallus gallus finished cDNA, clone ChEST718e20 / Finished cDNA, clone ChEST718e20 / similar to histamine H3 receptor H3S / --- / --- / --- / --- / Gallus gallus finished cDNA, clone ChEST718e20 / Finished cDNA, clone ChEST718e20
RIGG16308	0.163	0.0488	AGATCAAACGATCCCTGTTCAATTA CCACGACACCAGAATTCATGCCTGC CTGTATTTCATTGCACCAAC		ENSGALT00000018712.1 / Gallus gallus finished cDNA, clone ChEST964e9 / Gallus gallus mRNA for hypothetical protein, clone 3d14 / Septin 6 (SEPT6), mRNA / similar to Septin 11 / --- / --- / --- / septin 11; / --- / ---

Gene Name	Fold Change	p-value	SEQUENCE	Array Description	Blast/Database Description
RIGG16679	0.161	0.0116	CAGATGTTCCATACCTAACAGGGCATCAAAATCAAATTCCTTCAGATCCATCTTCTCCACCATCCACTCC		ENSGALT00000019823.1 / UP|Q9W610_CHICK (Q9W610) ATF4, partial (53%) / Gallus gallus activating transcription factor 4 (tax-responsive enhancer element B67) (ATF4), mRNA / ATF4 / ATF4: ATF4 / --- / ATF4 protein; / --- / --- / Gallus gallus activating transcription factor 4 (tax-responsive enhancer element B67) (ATF4), mRNA / ATF4
RIGG19582	0.16	0.0339	TCGGAAAGTTATGGTTCTGTCCGCTTATTTGATCATGCACTAGATTTTGTACAGCACCACTTCTCCCTTCT		ENSGALT00000027743.1 / Gallus gallus finished cDNA, clone ChEST29a19 / 603858829F1 CSEQCHN75 Gallus gallus cDNA clone ChEST867i7 5', mRNA sequence / Finished cDNA, clone ChEST29a19 / hypothetical protein / PREDICTED: Gallus gallus similar to Kelch repeat and BTB domain containing protein 3 (BTB and kelch domain containing protein 3) (LOC418976), mRNA. / PREDICTED: similar to Kelch repeat and BTB domain containing protein 3 (BTB and kelch domain containing protein 3); / Finished cDNA, clone ChEST29a19; / --- / 603858829F1 CSEQCHN75 Gallus gallus cDNA clone ChEST867i7 5', mRNA sequence / Finished cDNA, clone ChEST29a19
RIGG14687	0.143	0.0498	ACTTATGTTCCTGGACCTTTGAAGGCAAGAAACAACATGCAGTGGGAATGAAATCTAAGCCTTGCTGCAC		ENSGALT00000014237.1 / --- / Gallus gallus finished cDNA, clone ChEST619k5 / Finished cDNA, clone ChEST619k5 / --- / --- / --- / --- / --- / Gallus gallus finished cDNA, clone ChEST619k5 / Finished cDNA, clone ChEST619k5
RIGG10664	0.127	0.0413	GTGGAGAACCTCAGCCATTACAAGAAAACCATCCTAGAGATCCACAGTGCCGAGGGCAGGGAACG		ENSGALT00000002550.1 / similar to UP|PTN9_MOUSE (O35239) Tyrosine-protein phosphatase non-receptor type 9 (Protein-tyrosine phosphatase MEG2) (PTPase-MEG2) , partial (47%) / 603222024F1 CSEQRBN10 Gallus gallus cDNA clone ChEST217p5 5', mRNA sequence / Transcribed locus, weakly similar to NP_572576.1 lethal (1) G0232 CG32697-PA, isoform A [Drosophila melanogaster] / similar to protein-tyrosine phosphatase / PREDICTED: Gallus gallus similar to Protein-tyrosine phosphatase, non-receptor type 9 (Protein-tyrosine phosphatase MEG2) (PTPase-MEG2) (LOC429832), partial mRNA. / PREDICTED: similar to Protein-tyrosine phosphatase, non-receptor type 9 (Protein-tyrosine phosphatase MEG2) (PTPase-MEG2), partial; / Transcribed locus, weakly similar to NP_572576.1 lethal (1) G0232 CG32697-PA, isoform A [Drosophila melanogaster]; / --- / 603222024F1 CSEQRBN10 Gallus gallus cDNA clone ChEST217p5 5', mRNA sequence / Transcribed locus, weakly similar to NP_572576.1 lethal (1) G0232 CG32697-PA, isoform A [Drosophila melanogaster]
RIGG18036	0.109	0.0139	ACTTCTTGGCAGTCTGCAATCCTGACTGGTCCAAGGTGAACTGCTCCATCTATGTGCAGCTGGAG		ENSGALT00000023510.1 / Gallus gallus finished cDNA, clone ChEST270j4 / Gallus gallus finished cDNA, clone ChEST62d9 / Finished cDNA, clone ChEST62d9 / --- / --- / --- / --- / --- / --- / ---

Gene Name	Fold Change	p-value	SEQUENCE	Array Description	Blast/Database Description
RIGG19630	0.108	0.0374	GTACCTTCTCTAGGCCTGCCTTTAACCTGAAGAAACCCTATAAGTACTGTAACTGGAAATGCGCTGCCCT		ENSGALT00000027896.1 / --- / Gallus gallus teneurin 2 (TEN2), transcript variant 1, mRNA / Teneurin-2, short splice variant (ten2 gene) / --- / PREDICTED: Gallus gallus similar to KIAA1302 protein (LOC429230), partial mRNA. / PREDICTED: similar to KIAA1302 protein, partial; / --- / --- / --- / ---
RIGG19177	0.101	0.00715	GGTGATCTCAGGGATCCTCACACAAGGGCGCTGTGATGCTGATGAGTGGATGACCAAATACAGCG		ENSGALT00000026665.1 / --- / --- / --- / similar to retinoschisis (X-linked, juvenile) 1 / --- / --- / --- / retinoschisis (X-linked, juvenile) 1; / --- / ---
RIGG17606	0.1	0.00575	TGAGCTTCTCACTCACATACCTAGGCATAAATTGAATACTTTTCATGTGCGAATGGAAGACGATGCCCAG		ENSGALT00000022435.1 / homologue to UP|HDC_HUMAN (Q9UBI9) Headcase protein homolog (hHDC), partial (75%) / --- / --- / --- / PREDICTED: Gallus gallus similar to Headcase protein homolog (hHDC) (LOC421677), mRNA. / PREDICTED: similar to Headcase protein homolog (hHDC); / --- / --- / --- / ---
RIGG19593	0.0736	0.00284	TAACAAGTGCTCAGTCCACCTTACACATGTTCTCTACTTCCTCATCCTCTGTAACATTGCTTTTGGTACT	FAR-2 protein [Gallus gallus]. [Source:RefSeq;Acc:NM_204612]	FAR-2 protein [Gallus gallus]. [Source:RefSeq / UP|CNTN5_CHICK (Q90W79) Contactin-5 precursor (F11 axonin-1-related protein 2) (FAR-2), complete / Gallus gallus contactin 5 (CNTN5), mRNA / FAR-2 protein fragment / CNTN5, FAR2: Contactin-5 precursor / Gallus gallus FAR-2 protein (FAR-2), mRNA. / FAR-2 protein; / FAR-2 protein fragment; / --- / Gallus gallus contactin 5 (CNTN5), mRNA / FAR-2 protein fragment
RIGG09072	0.06	0.018	AAACTATCATGAATATTCCTGGCTTCTACTTGGTGCGTCGGGAAAACCCAGAGTACTTTCCCCGTGGGAG		ENSGALG00000012144.1 / --- / Gallus gallus finished cDNA, clone ChEST551f12 / Finished cDNA, clone ChEST551f12 / --- / PREDICTED: Gallus gallus similar to hypothetical protein FLJ20232 (LOC418012), mRNA.PREDICTED: Gallus gallus similar to hypothetical protein FLJ20232 (LOC425686), partial mRNA. / PREDICTED: similar to hypothetical protein FLJ20232;PREDICTED: similar to hypothetical protein FLJ20232, partial; / --- / --- / --- / ---
RIGG18277	0.0563	0.0301	GGTTATTGGGCAGAATGGTATCTTGTCCACACCTGCCGTTTCATGTATTATTCGAAAGATAAAAGCAGCTG		ENSGALT00000024183.1 / --- / Gallus gallus phosphoglucomutase 1 mRNA, complete cds / Phosphoglucomutase 1 (PGM1), mRNA / similar to phosphoglucomutase 5, partial / --- / --- / --- / --- / --- / ---
RIGG19767	0.0495	0.0388	GTCAGATTCGTATGCAGTCTACTCCCTCAGTGAAGGTTCAATATAGGAACCCTACCACTGGCATGTGGGA		ENSGALT00000028177.1 / weakly similar to GB|AAK11553.1|13021822|AF298587 polymerase {Homo sapiens} (exp=-1; wgp=0; cg=0), partial (4%) / gonad_EST08940 Embryonic gonad cDNA Library Gallus gallus cDNA 5', mRNA sequence / Transcribed locus, weakly similar to XP_426852.1 PREDICTED: similar to pol protein, partial [Gallus gallus] / --- / --- / --- / --- / --- / gonad_EST08940 Embryonic gonad cDNA Library Gallus gallus cDNA 5', mRNA sequence / Transcribed locus, weakly similar to XP_426852.1 PREDICTED: similar to pol protein, partial [Gallus gallus]

Gene Name	Fold Change	p-value	SEQUENCE	Array Description	Blast/Database Description
RIGG16454	0.0453	0.0441	AACATGCAAAACGCCGAGTACAAGAGGAGGAACACAAAAGCGAAATCACACGGCAAGAAGGAAGCACATC		ENSGALT00000019154.1 / --- / --- / --- / --- / --- / --- / --- / --- / --- / ---
RIGG09051	0.0436	0.00161	AAAGAGAAGGAGCACATCAAAGCCTATGTTCTCTTTCAAAGAAGCACCCAAAGTAAGTCCGAATCCTGCC		ENSGALG00000011994.1 / --- / --- / --- / similar to synaptopodin 2 / PREDICTED: Gallus gallus similar to Myopodin protein (LOC428768), mRNA. / PREDICTED: similar to Myopodin protein; / --- / --- / --- / ---
RIGG02449	0.0388	0.0172	TTAGAGCTGCTGAGAATTAATGTTTGTAATGAGCGTCTGGTGATTGCCTTAAACATCTCTGCATTTTGCG	Weakly similar to Q9NTW6 (Q9NTW6) DJ777D9.1.1 (Hypothalamus protein HT013, is	Weakly similar to Q9NTW6 (Q9NTW6) DJ777D9.1.1 (Hypothalamus protein HT013, is / Gallus gallus finished cDNA, clone ChEST347j23 / Gallus gallus mRNA for hypothetical protein, clone 13o7 / Similar to uncharacterized hypothalamus protein HT013 (LOC421237), mRNA / --- / PREDICTED: Gallus gallus similar to uncharacterized hypothalamus protein HT013 (LOC421237), mRNA. / PREDICTED: similar to uncharacterized hypothalamus protein HT013; / --- / --- / --- / ---
RIGG16831	0.0369	0.0162	CCATTTGGGATACAAAGAGAGGCAGTGGAGAAGTTGGATTATCGCACACATCATCTGGATGCTCCTAAGG		ENSGALT00000020244.1 / Gallus gallus finished cDNA, clone ChEST771g3 / Gallus gallus finished cDNA, clone ChEST771g3 / Finished cDNA, clone ChEST771g3 / --- / --- / --- / --- / --- / Gallus gallus finished cDNA, clone ChEST771g3 / Finished cDNA, clone ChEST771g3
RIGG09134	0.0367	0.0203	GAAGCTCGCCGAGTTTATGGTGATGTCATTCCAGCATCTGCAAAAGATAGAAGAATCCTGGTGCTGAAAC		ENSGALG00000012663.1 / similar to UP|SSDH_HYLLA (Q3MSM3) Succinate semialdehyde dehydrogenase, mitochondrial precursor (NAD(+)-dependent succinic semialdehyde dehydrogenase) , partial (86%) / --- / --- / similar to succinic semialdehyde dehydrogenase / PREDICTED: Gallus gallus similar to aldehyde dehydrogenase 5A1 precursor isoform 2; mitochondrial succinate semialdehyde dehydrogenase; NAD(+)-dependent succinic semialdehyde dehydrogenase (LOC420818), mRNA. / PREDICTED: similar to aldehyde dehydrogenase 5A1 precursor isoform 2; mitochondrial succinate semialdehyde dehydrogenase; NAD(+)-dependent succinic semialdehyde dehydrogenase; / Transcribed locus, moderately similar to XP_545368.2 PREDICTED: similar to aldehyde dehydrogenase 5A1 precursor, isoform 2 [Canis familiaris]; / --- / --- / ---
RIGG11869	0.0361	0.0101	CCAGGATTTGTCCCAGAGAAAGTGGGGATCTAAAGACTTGCAGATTAAACCAGGAGAGACTCTTGAAGTTATACA		ENSGALT00000006010.1 / homologue to RF|NP_001026622.1|71895395|NM_001031451 FYN binding protein (FYB-120/130) {Gallus gallus} (exp=-1; wgp=0; cg=0), partial (40%) / Gallus gallus mRNA for hypothetical protein, clone 32b13 / FYN binding protein (FYB-120/130) (FYB), mRNA / --- / --- / --- / FYN binding protein (FYB-120/130) (FYB), mRNA; / --- / Gallus gallus mRNA for hypothetical protein, clone 32b13 / FYN binding protein (FYB-120/130) (FYB), mRNA

Gene Name	Fold Change	p-value	SEQUENCE	Array Description	Blast/Database Description
RIGG05099	0.036	0.0152	ACTCTCTCGTACGGACTGCACCAGT TATTTATTGTTGCCTTTTCACGTTTC CTTCATCCACACATGCACA	Genome Hit Contig4293.3	Genome Hit Contig4293.3 / Gallus gallus finished cDNA, clone ChEST711a9 / Gallus gallus serum response factor (SRF) mRNA, MADS box to COOH terminus, partial cds / Serum response factor (SRF) mRNA, MADS box to COOH terminus / --- / --- / --- / --- / --- / Gallus gallus serum response factor (SRF) mRNA, MADS box to COOH terminus, partial cds / Serum response factor (SRF) mRNA, MADS box to COOH terminus
RIGG18333	0.0329	0.0313	GGACGTGTGGAAAAGCTTGAAGGC GAATGCGATGAAAATGGAGGAACAA GGGGATCTCAGCTTTTG		ENSGALT00000024356.1 / Gallus gallus finished cDNA, clone ChEST784d1 / 603805902F1 CSEQCHN57 Gallus gallus cDNA clone ChEST784d1 5', mRNA sequence / Finished cDNA, clone ChEST784d1 / --- / --- / --- / Finished cDNA, clone ChEST531a9;Finished cDNA, clone ChEST784d1; / chromosome 9 open reading frame 94; / 603805902F1 CSEQCHN57 Gallus gallus cDNA clone ChEST784d1 5', mRNA sequence / Finished cDNA, clone ChEST784d1
RIGG09996	0.0318	0.00141	TCACGTATGCAGACTTGGCTGTGAT CGTCCCGTTAAAACCTTTCAAGGAC ACACAAATGAGGTTAATGCTATCA		ENSGALT00000000704.1 / homologue to UP|TBLX_MOUSE (Q9QXE7) F-box-like/WD-repeat protein TBL1X (Transducin beta-like 1X protein), complete / Gallus gallus mRNA for hypothetical protein, clone 8j10 / Transducin (beta)-like 1X-linked receptor 1 (TBL1XR1), mRNA / RCJMB04_8j10: Hypothetical protein / PREDICTED: Gallus gallus similar to transducin (beta)-like 1 X-linked; transducin (beta)-like 1 (LOC426045), partial mRNA.PREDICTED: Gallus gallus similar to transducin (beta)-like 1 X-linked; transducin (beta)-like 1 (LOC426686), partial mRNA.PREDICTED: Gallus gallus similar to transducin (beta)-like 1 X-linked; transducin (beta)-like 1 (LOC426944), partial mRNA. / PREDICTED: similar to transducin (beta)-like 1 X-linked; transducin (beta)-like 1, partial;PREDICTED: similar to transducin (beta)-like 1 X-linked; transducin (beta)-like 1, partial;PREDICTED: similar to transducin (beta)-like 1 X-linked; transducin (beta)-like 1, partial; / Finished cDNA, clone ChEST866e14; / --- / --- / ---
RIGG14675	0.0317	0.0153	TTCCCTCGCAGTTTAGTCTCAGAAC AACATGTCTATCAAGAAGCAAATAA GACCATTCCTACAACGGACA	Otoraplin precursor. [Source:SWISSPROT;Acc:Q9I8P6]	Otoraplin precursor. [Source:SWISSPROT / UP|OTOR_CHICK (Q9I8P6) Otoraplin precursor, complete / Gallus gallus otoraplin (OTOR), mRNA / Otoraplin (Otor) / --- / Gallus gallus otoraplin (OTOR), mRNA. / otoraplin; / Otoraplin (Otor); / --- / Gallus gallus otoraplin (OTOR), mRNA / Otoraplin (Otor)
RIGG17940	0.031	0.0154	GGGATTAGACGCTTTAGTTCAACTG AGTGCTGTTGTTGGCCCATCTCTTA ACGACCATCTTAAGCATCTG		ENSGALT00000023273.1 / Gallus gallus finished cDNA, clone ChEST973h15 / 603595916F1 CSEQCHN73 Gallus gallus cDNA clone ChEST563o3 5', mRNA sequence / Finished cDNA, clone ChEST973h15 / --- / --- / --- / --- / --- / 603595916F1 CSEQCHN73 Gallus gallus cDNA clone ChEST563o3 5', mRNA sequence / Finished cDNA, clone ChEST973h15

Gene Name	Fold Change	p-value	SEQUENCE	Array Description	Blast/Database Description
RIGG16429	0.0304	0.000698	CAATGGCGCGATCGGATTCAAAATG TTTTGTCCTGGCATTGGTTTCTAACT GAAATAAATTGCATCTCTG		ENSGALT00000019112.1 / Gallus gallus finished cDNA, clone ChEST815k14 / Gallus gallus finished cDNA, clone ChEST815k14 / Finished cDNA, clone ChEST815k14 / --- / PREDICTED: Gallus gallus similar to BC022687 protein (LOC423498), mRNA. / PREDICTED: similar to BC022687 protein; / Finished cDNA, clone ChEST815k14; / --- / Gallus gallus finished cDNA, clone ChEST815k14 / Finished cDNA, clone ChEST815k14
RIGG18046	0.0299	0.0465	GCTGTAACAGATGGATGCAAAGGGA CCTCAGCATTTAAAGAAAGAACAAC GGAGAAAATCTCTACATCTGC		ENSGALT00000023555.1 / --- / --- / --- / --- / --- / --- / --- / --- / --- / ---
RIGG05141	0.0283	0.0382	GGGAGGACTTGCATATGGAAATCTT TCAGAGGAGGCATAAGAGGAACAGA AATTGCTAATCAAGTTTTGCA	Weakly similar to Q21389 (Q21389) K09C8.4 protein	Weakly similar to Q21389 (Q21389) K09C8.4 protein / --- / Gallus gallus glycosyltransferase-like 1A mRNA, complete cds / Like-glycosyltransferase (LARGE), mRNA / LARGE, LARGE1: Glycosyltransferase-like protein LARGE1 / PREDICTED: Gallus gallus similar to acetylglucosaminyltransferase-like protein (LOC418061), mRNA. / PREDICTED: similar to acetylglucosaminyltransferase-like protein; / Like-glycosyltransferase (LARGE), mRNA; / --- / --- / ---
RIGG15909	0.0281	0.0408	GTACTCACCCTGCATTGCGTGAAAT GTGTAAGTTCTGAAGAGTGAGACTA GTAGAGAAGAGCATACACAT		ENSGALT00000017548.1 / --- / --- / --- / --- / --- / --- / --- / --- / --- / ---
RIGG10013	0.0214	0.00598	GAGCTTTCAGCCAACGAGCTCAGCA TCTATGACAAGCTATCAGAGACAAT TGATCTGGTGAGACAGACTG		ENSGALT00000000767.1 / similar to UP|Q8IZ35_HUMAN (Q8IZ35) C6orf89 protein (Fragment), partial (34%) / Gallus gallus mRNA for hypothetical protein, clone 35h15 / Similar to CDNA sequence BC004004 (LOC419817), mRNA / --- / PREDICTED: Gallus gallus similar to CDNA sequence BC004004 (LOC419817), mRNA. / PREDICTED: similar to CDNA sequence BC004004; / Similar to CDNA sequence BC004004 (LOC419817), mRNA; / --- / Gallus gallus mRNA for hypothetical protein, clone 35h15 / Similar to CDNA sequence BC004004 (LOC419817), mRNA
RIGG18072	0.0208	0.0115	TGGTGTCTCATTTCCCTCTTCTCCAA GTGGAATGTCTTTTGAGTTTACATCT TCTCTTCATGCCATTGAGG		ENSGALT00000023635.1 / --- / --- / --- / --- / --- / --- / --- / chromosome 5 open reading frame 36; / --- / ---
RIGG16220	0.0204	0.0199	AATTTATAGATGAACATGCCTTCAAA GGGGTGGCAGAAACTTTGCAGACTC TGGATTTGTCCGACAACCG		ENSGALT00000018458.1 / homologue to UP|LRC3B_HUMAN (Q96PB8) Leucine-rich repeat-containing protein 3B precursor (Leucine-rich repeat protein LRP15), complete / Gallus gallus finished cDNA, clone ChEST127l8 / Finished cDNA, clone ChEST127l8 / similar to LRP15 / PREDICTED: Gallus gallus similar to hypothetical protein LRP15 (LOC420657), mRNA. / PREDICTED: similar to hypothetical protein LRP15; / Finished cDNA, clone ChEST127l8; / --- / Gallus gallus finished cDNA, clone ChEST127l8 / Finished cDNA, clone ChEST127l8

Gene Name	Fold Change	p-value	SEQUENCE	Array Description	Blast/Database Description
RIGG10022	0.0204	0.0284	AAATACCCCTCTGCTACCCCACACC CTTTGAATTCTCGTTGCCATACATCT CCTCCCAGCAGACA		ENSGALT00000000782.1 / --- / --- / --- / --- / --- / --- / --- / --- / --- / ---
RIGG05522	0.0202	0.0134	CTGACTCAGAACTCACCAAGTCCCG ATTTTCACAATAATCTTTAGCTCTCT TAGTGAGAAACATGCAGCT	Genome Hit Contig37.16	Genome Hit Contig37.16 / Gallus gallus finished cDNA, clone ChEST764e10 / Gallus gallus finished cDNA, clone ChEST764e10 / Finished cDNA, clone ChEST764e10 / --- / --- / --- / --- / --- / Gallus gallus finished cDNA, clone ChEST764e10 / Finished cDNA, clone ChEST764e10
RIGG11147	0.0193	0.0059	GAAGGTGCAGCAGTTTTACAGACTC TGCCAGCAGAACATGGATCCCAACG CATTCCCGGTGCCCA		ENSGALT00000003979.1 / --- / --- / --- / / --- / --- / --- / --- / --- / ---
RIGG16532	0.0192	0.00703	CTTCGCAAGGCCATGAAGGGAATGG GGACTGATGAAGAGACAATTCTGAA GATCCTTACCAGCAGAAATAA	Annexin A5 (Annexin V) (Lipocortin V) (Endonexin II) (Calphobindin I) (CBP-I) (Placental anticoagulant protein I) (PAP-I) (PP4) (Thromboplastin inhibitor) (Vascular anticoagulant-alpha) (VAC-alpha) (Anchorin CII). [Source:SWISSPROT;Acc:P17153]	Annexin A5 (Annexin V) (Lipocortin V) (Endonexin II) (Calphobindin I) (CBP-I) (Placental anticoagulant protein I) (PAP-I) (PP4) (Thromboplastin inhibitor) (Vascular anticoagulant-alpha) (VAC-alpha) (Anchorin CII). [Source:SWISSPROT / GB|1YII_A|62738641|1YII_A Chain A, Crystal Structures Of Chicken Annexin V In Complex With Ca2+. {Gallus gallus} (exp=-1; wgp=0; cg=0), complete / gonad_EST08070 Embryonic gonad cDNA Library Gallus gallus cDNA 5', mRNA sequence / Anchorin CII mRNA, 3' end / ANXA5, ANX5: Annexin A5 / --- / --- / --- / --- / gonad_EST08070 Embryonic gonad cDNA Library Gallus gallus cDNA 5', mRNA sequence / Anchorin CII mRNA, 3' end
RIGG19372	0.0185	0.0441	GAGTGGAGATGAGCGCCTATGCCA GCATAGACTTTCTGTCTCACAACCT GAAAGAAGCAAGCGCT		ENSGALT00000027194.1 / --- / --- / --- / --- / PREDICTED: Gallus gallus similar to insulin receptor substrate 2 (LOC428017), mRNA. / PREDICTED: similar to insulin receptor substrate 2; / --- / --- / --- / ---
RIGG19606	0.0182	0.0406	CTGAAAACATCATCCTTTGGAACATT GTCCTTTTCTCTATTCTCCTGGCCAT TGGTGTGATTGAAGCAAT		ENSGALT00000027824.1 / Gallus gallus finished cDNA, clone ChEST155l12 / Gallus gallus finished cDNA, clone ChEST155l12 / Finished cDNA, clone ChEST155l12 / hypothetical protein isoform 1 / PREDICTED: Gallus gallus similar to TM4SF1 protein (LOC418999), mRNA. / PREDICTED: similar to TM4SF1 protein; / Finished cDNA, clone ChEST155l12; / --- / Gallus gallus finished cDNA, clone ChEST155l12 / Finished cDNA, clone ChEST155l12
RIGG18145	0.0161	0.00249	ATATGGGAAGCAACAAAAGGCCAAC TTTATCTCTGAGATGAAACTGGTCAT CGTTTTAAGTCCGGCCCGA		ENSGALT00000023881.1 / --- / --- / --- / --- / --- / --- / --- / --- / --- / ---

Gene Name	Fold Change	p-value	SEQUENCE	Array Description	Blast/Database Description
RIGG10342	0.0152	0.0271	AGATTGAAGCCAAATACCCCGACGA GTTTGCCTTGAGGGATCAGGAGAAA TACCTCTATCGCTATCCTGG		ENSGALT00000001686.1 / homologue to GB|AAB29678.1|544902|S67900 fructose 6-phosphate,2-kinase:fructose 2,6-bisphosphatase {Rattus sp.} (exp=-1; wgp=0; cg=0), partial (75%) / Gallus gallus 6-phosphofructo-2-kinase/fructose-2,6-biphosphatase 4 (PFKFB4), mRNA / 6-phosphofructo-2-kinase/fructose-2, 6-bisphosphatase / similar to 6-phosphofructo-2-kinase/fructose-2,6-biphosphatase 2 / PREDICTED: Gallus gallus similar to 6-phosphofructo-2-kinase/fructose-2,6-biphosphatase 2 (6PF-2-K/Fru-2,6-P2ASE heart-type isozyme) (PFK-2/FBPase-2) (LOC419850), mRNA. / PREDICTED: similar to 6-phosphofructo-2-kinase/fructose-2,6-biphosphatase 2 (6PF-2-K/Fru-2,6-P2ASE heart-type isozyme) (PFK-2/FBPase-2); / --- / --- / --- / ---
RIGG12269	0.0138	0.0322	CCATCGGTGATTCCAGTGCAGAAAG CCTCTATGTTCAAGGCATGGAGCTG TTTGAGGAGGCCCTG		ENSGALT00000007150.1 / Gallus gallus finished cDNA, clone ChEST385h7 / --- / --- / --- / PREDICTED: Gallus gallus similar to RIKEN cDNA 5730472N09 (LOC426768), partial mRNA. / PREDICTED: similar to RIKEN cDNA 5730472N09, partial; / --- / --- / --- / ---
RIGG13427	0.0137	0.0142	CTGATGGTATTCTAAATTCAGTCAAC CACACCTCTCCTATACTAGCAGTGG AACCTGTCTCAACTCCTCT		ENSGALT00000010589.1 / Gallus gallus finished cDNA, clone ChEST64i12 / Gallus gallus finished cDNA, clone ChEST64i12 / Finished cDNA, clone ChEST976j1 / --- / --- / --- / --- / WD repeat domain 60; / Gallus gallus finished cDNA, clone ChEST64i12 / Finished cDNA, clone ChEST976j1
RIGG15687	0.0136	0.0454	GAATATGATGTGTGTGCCATTTGTC TGGATGAATATGAGGATGGAGACAA GCTCAGAATCCTTCCATGCT	RING finger protein 13 (C-RZF). [Source:SWISSPROT;Acc:Q90972]	RING finger protein 13 (C-RZF). [Source:SWISSPROT / UP|RNF13_CHICK (Q90972) RING finger protein 13 (C-RZF), complete / Gallus gallus ring finger protein 13 (RNF13), mRNA / RING zinc finger / RNF13, RZF: RING finger protein 13 / Gallus gallus RING zinc finger protein (RZF), mRNA. / --- / RING zinc finger; / --- / Gallus gallus ring finger protein 13 (RNF13), mRNA / RING zinc finger
RIGG17563	0.0135	0.0243	TAACAGTTGATCCAGTTTCTGCCAG TGTCGTCCTTGCAAACTTCCCAGAG AACGAACCGGTGTCCATTTC		ENSGALT00000022337.1 / Gallus gallus finished cDNA, clone ChEST189c23 / --- / --- / / PREDICTED: Gallus gallus similar to Gem-associated protein 6 (Gemin6) (SIP2) (LOC426243), mRNA.PREDICTED: Gallus gallus similar to Gem-associated protein 6 (Gemin6) (SIP2) (LOC426266), mRNA. / PREDICTED: similar to Gem-associated protein 6 (Gemin6) (SIP2);PREDICTED: similar to Gem-associated protein 6 (Gemin6) (SIP2); / --- / --- / --- / ---
RIGG14194	0.0133	0.00691	GCTCCATGTTAGCTGTGATTCTCAA CAATATGCTTCCAACTTCCTCCAATG AACCACCCATAATAGTGCT		ENSGALT00000012851.1 / --- / --- / --- / / --- / --- / --- / --- / --- / ---
RIGG14126	0.0131	0.00013	TCTTCCACAATGTCTCTGAACACCA GTTCTCCATCGCTAAAGAAGGTCAT TTTGTCCTTGTAGGTCTGCA		ENSGALT00000012625.1 / UP|SRCA_CHICK (Q90577) Sarcalumenin precursor, complete / G.domesticus mRNA for 53 kDa glycoprotein / 53 kDa glycoprotein / SRL: Sarcalumenin precursor / --- / --- / --- / --- / G.domesticus mRNA for 53 kDa glycoprotein / 53 kDa glycoprotein

Gene Name	Fold Change	p-value	SEQUENCE	Array Description	Blast/Database Description
RIGG10747	0.0128	0.00132	GCAATCAGATATCTGAATCTAAGGG CAGAGTTTAATGTGAAGTAGAAAAG TGGTCTGTAGCCAGGCTCTC		ENSGALT00000002834.1 / Gallus gallus finished cDNA, clone ChEST845a6 / Gallus gallus mRNA for hypothetical protein, clone 9c11 / Hypothetical protein, clone 9c11 / --- / PREDICTED: Gallus gallus similar to adaptor protein with pleckstrin homology and src homology 2 domains (LOC417508), mRNA. / PREDICTED: similar to adaptor protein with pleckstrin homology and src homology 2 domains; / Hypothetical protein, clone 9c11; / --- / Gallus gallus mRNA for hypothetical protein, clone 9c11 / Hypothetical protein, clone 9c11
RIGG16764	0.0128	0.0142	AATAGCTGGTCCGCATGTACATATG TCCACATACCTACACAGAGATATGT CTACTTGCGTCCTGCACCCA	Monocarboxylate transporter 3 (MCT 3) (Retinal epithelial membrane protein). [Source:SWISSPROT;Acc:Q90632]	Monocarboxylate transporter 3 (MCT 3) (Retinal epithelial membrane protein). [Source:SWISSPROT / UP|MOT3_CHICK (Q90632) Monocarboxylate transporter 3 (MCT 3) (Retinal epithelial membrane protein), complete / Gallus gallus solute carrier family 16, member 8 (monocarboxylic acid transporter 3) (SLC16A8), mRNA / Retinal epithelial membrane protein / SLC16A3, MCT3, REMP: Monocarboxylate transporter 3 / Gallus gallus retinal epithelial membrane protein (LOC396041), mRNA. / retinal epithelial membrane protein; / Retinal epithelial membrane protein; / --- / Gallus gallus solute carrier family 16, member 8 (monocarboxylic acid transporter 3) (SLC16A8), mRNA / Retinal epithelial membrane protein
RIGG17173	0.0123	0.0112	CTTCCTCTTCTTCACGCGCATTCCA ACTGTCTCTATTTCTCTATCTCTGTC TCACACATACGTCTTGTTG	CHUNK-1 protein [Gallus gallus]. [Source:RefSeq;Acc:NM_204836]	CHUNK-1 protein [Gallus gallus]. [Source:RefSeq / UP|Q9PW10_CHICK (Q9PW10) CHUNK-1 protein, complete / Gallus gallus CHUNK-1 protein (CHUNK-1), mRNA / CHUNK-1 protein / --- / Gallus gallus CHUNK-1 protein (CHUNK-1), mRNA. / CHUNK-1 protein; / CHUNK-1 protein; / --- / Gallus gallus CHUNK-1 protein (CHUNK-1), mRNA / CHUNK-1 protein
RIGG18085	0.0122	0.0068	TTGACCATCACGTTGCTGCACTATG GATGTGCCTCCTTCATGTACATTCG TCCCAAGTCCAGTTACTCAC		ENSGALT00000023665.1 / --- / Gallus gallus olfactory receptor 4 (cor4), mRNA / Olfactory receptor 4 (cor4), mRNA / similar to olfactory receptor MOR0-2 like protein / PREDICTED: Gallus gallus similar to olfactory receptor Olr804 (LOC427948), mRNA. / PREDICTED: similar to olfactory receptor Olr804; / --- / --- / --- / ---
RIGG19599	0.0112	0.0174	TCCCCTATGATGTGGGAAGGGTCAT CACCAACATCTTCTTGGGCTTTCTG GGAATTATTCGAGATGTGGC		ENSGALT00000027811.1 / --- / --- / --- / similar to Endonuclease domain containing 1 / PREDICTED: Gallus gallus similar to RIKEN cDNA 2310067E08 (LOC428090), mRNA. / PREDICTED: similar to RIKEN cDNA 2310067E08; / --- / --- / --- / ---
RIGG17260	0.0111	0.0443	GATCAAATTGAGGACATACAAGATC AGTTGGAGGATATGATGGAAGAAGC CAATGAAGTCCAGGAAGCAC		ENSGALT00000021491.1 / homologue to UP|CHMP5_HUMAN (Q9NZZ3) Charged multivesicular body protein 5 (Chromatin-modifying protein 5) (Vacuolar protein sorting 60) (Vps60) (hVps60) (SNF7 domain-containing protein 2), complete / gonad_EST10077 Embryonic gonad cDNA Library Gallus gallus cDNA 5', mRNA sequence / Finished cDNA, clone ChEST215g2 / / PREDICTED: Gallus gallus similar to HSPC177 (LOC420968), mRNA. / PREDICTED: similar to HSPC177; / --- / --- / gonad_EST10077 Embryonic gonad cDNA Library Gallus gallus cDNA 5', mRNA sequence / Finished cDNA, clone ChEST215g2

Gene Name	Fold Change	p-value	SEQUENCE	Array Description	Blast/Database Description
RIGG06371	0.0108	0.00873	ACCGAACATCTCCCTAAAGGCTATA GATTCAGCTTTGAGACGTTATCCTG GAGAGCAAATCAGTAAATGCA	Similar to S612_MOUSE (Q9JLR1) Protein transport protein Sec61 alpha s	Similar to S612_MOUSE (Q9JLR1) Protein transport protein Sec61 alpha s / GB|AAH05458.1|13529458|BC005458 Sec61a2 protein {Mus musculus} (exp=-1; wgp=0; cg=0), partial (32%) / Gallus gallus finished cDNA, clone ChEST769e24 / Finished cDNA, clone ChEST884d22 / / PREDICTED: Gallus gallus similar to Sec61 alpha isoform 2 (LOC426369), partial mRNA. / PREDICTED: similar to Sec61 alpha isoform 2, partial; / --- / --- / --- / ---
RIGG12637	0.00987	0.00413	TGATAAGCTGTCCTTGAGGTTTAAT GGGAGAGTGCTCTTTATAAAAGATG TGATTGGAGACGAGATCTGCT		ENSGALT00000008249.1 / homologue to UP|Q9H3F6_HUMAN (Q9H3F6) MSTP028 (Potassium channel tetramerisation domain containing 10) (ULR061), partial (87%) / Gallus gallus mRNA for hypothetical protein, clone 19f9 / Tumor necrosis factor, alpha-induced protein 1 (endothelial) (TNFAIP1), mRNA / similar to MSTP028 / PREDICTED: Gallus gallus similar to potassium channel tetramerisation domain containing 10; MSTP028 protein (LOC416900), mRNA. / PREDICTED: similar to potassium channel tetramerisation domain containing 10; MSTP028 protein; / --- / --- / --- / ---
RIGG19023	0.0098	0.0227	ACGGACAAGCACCAAAAGCATCAGT TATCCACTTGAGCATTCGCATGTGT TTGCTTAGGGCTTTGAGTGG		ENSGALT00000026203.1 / Gallus gallus finished cDNA, clone ChEST746a16 / Gallus gallus finished cDNA, clone ChEST746a16 / Finished cDNA, clone ChEST746a16 / --- / --- / --- / --- / --- / Gallus gallus finished cDNA, clone ChEST746a16 / Finished cDNA, clone ChEST746a16
RIGG18052	0.00942	0.0442	CAGTGACAGAGGGGTGCAGCAACA AACGAAGGTGCAGGAATCAGTTTTG CAATCCAGCTTTCCTGATAAA		ENSGALT00000023567.1 / similar to UP|Q96HU6_HUMAN (Q96HU6) Invasion inhibitory protein 45, isoform 1, partial (7%) / gPGC_EST05251 Embryonic gonadal PGC cDNA Library Gallus gallus cDNA 5', mRNA sequence / Finished cDNA, clone ChEST794i21 / --- / PREDICTED: Gallus gallus similar to IGFBP-2-Binding Protein, IIp45 (LOC419483), mRNA. / PREDICTED: similar to IGFBP-2-Binding Protein, IIp45; / Finished cDNA, clone ChEST794i21; / --- / gPGC_EST05251 Embryonic gonadal PGC cDNA Library Gallus gallus cDNA 5', mRNA sequence / Finished cDNA, clone ChEST794i21
RIGG16426	0.0091	0.00658	CAGTGTCTCGGAAAGAAGCAATAGA GATGTTAGAAAATAACCCTTTGTGT GGGAATTTGATCCTGCGTCC		ENSGALT00000019102.1 / --- / --- / --- / / --- / --- / --- / --- / --- / ---
RIGG17182	0.00902	0.0218	CCTCAACATGCTCTTCACTGGCCTC TTCACTGTCGAGATGGTCCTGAAGT TAATTGCCTTCAAACCCAAG		ENSGALT00000021256.1 / --- / Gallus gallus L-type voltage-gated calcium channel alpha1D subunit ChCaChA1D (LOC395895), mRNA / L-type voltage-gated calcium channel alpha1D subunit ChCaChA1D / CACNA1C: Voltage-dependent L-type calcium channel subunit alpha-1C (Fragment) / --- / --- / --- / --- / 603758505F1 CSEQCHN04 Gallus gallus cDNA clone ChEST673i2 5', mRNA sequence / Clone pSE1/3-2 L-type voltage-gated calcium channel alpha1C subunit ChCaChA1C

Gene Name	Fold Change	p-value	SEQUENCE	Array Description	Blast/Database Description
RIGG09075	0.00889	0.0178	GCTGAGAAAGCCCACTATTACAGACTCTATGTGTGATGTTGTCCTAGAGTGTGATGAAGATAGAATGGACTCAAC		ENSGALG00000012160.1 / --- / Gallus gallus mRNA for hypothetical protein, clone 5l15 / Similar to RIKEN cDNA 3000004C01 (LOC419968), mRNA / hypothetical protein / --- / --- / --- / kinesin family member 18A; / --- / ---
RIGG17188	0.0083	0.0129	GTCAATACTATGGTTTCCTCTATGAACCAGCAATGCACAAAGACCTCATATCTATCATCAAACCCAGGGA		ENSGALT00000021280.1 / --- / --- / --- / similar to protein kinase Bsk146 / PREDICTED: Gallus gallus similar to protein kinase Bsk146 (LOC420929), mRNA. / PREDICTED: similar to protein kinase Bsk146; / --- / --- / --- / ---
RIGG13397	0.00778	0.00691	TCCCACCTACATCGAGAGTCAACATTTCTAATCCGGGTCAAGTCTTACTGAAGAACCTCACTCAGATGAG		ENSGALT00000010518.1 / Gallus gallus finished cDNA, clone ChEST531d15 / Gallus gallus finished cDNA, clone ChEST531d15 / Finished cDNA, clone ChEST531d15 / --- / PREDICTED: Gallus gallus similar to adipocyte-specific adhesion molecule; CAR-like membrane protein (LOC419747), mRNA. / PREDICTED: similar to adipocyte-specific adhesion molecule; CAR-like membrane protein; / Finished cDNA, clone ChEST531d15; / --- / Gallus gallus finished cDNA, clone ChEST531d15 / Finished cDNA, clone ChEST531d15
RIGG13379	0.00738	0.00931	CTGGGCCCAAAGTGTATCCCTGCATTGAAAGCAAATGAATTTGTTGAGGCTTCAGTTTCTGAGGCTAATA		ENSGALT00000010477.1 / Gallus gallus finished cDNA, clone ChEST350d21 / Gallus gallus finished cDNA, clone ChEST350d21 / Finished cDNA, clone ChEST350d21 / --- / --- / --- / --- / --- / brain_EST02653 Brain cDNA Library Gallus gallus cDNA 3', mRNA sequence / Transcribed locus
RIGG18147	0.00692	0.00197	TAGTACCAGAGCGGCAGATCAGGATTACTGAAAAGCCAGGCATTCCAGATGATGCTTCAGATGAAGAGGG		ENSGALT00000023883.1 / similar to GB|CAI22655.1|56203388|HSJ757N13 ganglioside induced differentiation associated protein 2 {Homo sapiens} (exp=0; wgp=1; cg=0), partial (62%) / 603501484F1 CSEQCHN65 Gallus gallus cDNA clone ChEST419g22 5', mRNA sequence / Transcribed locus, weakly similar to XP_313968.2 ENSANGP00000009903 [Anopheles gambiae str. PEST] / --- / PREDICTED: Gallus gallus similar to ganglioside-induced differentiation-associated-protein 2 (LOC425913), partial mRNA. / PREDICTED: similar to ganglioside-induced differentiation-associated-protein 2, partial; / --- / --- / --- / ---
RIGG11171	0.00673	0.00165	CTGTGCCTCTCATCTTACTGTTGTGGCACTATTGTACATACCGCTGTTCTTCAATTATACACCTCCGTCTT		ENSGALT00000004045.1 / --- / Gallus gallus olfactory receptor 4 (cor4), mRNA / Olfactory receptor 4 (cor4), mRNA / / PREDICTED: Gallus gallus similar to bM332P19.2 (novel 7 transmembrane receptor (rhodopsin family) (olfactory receptor like) protein (mm17M1-13); ortholog of human DJ994E9.8 (HS6M1-20)) (LOC427485), partial mRNA. / PREDICTED: similar to bM332P19.2 (novel 7 transmembrane receptor (rhodopsin family) (olfactory receptor like) protein (mm17M1-13); ortholog of human DJ994E9.8 (HS6M1-20)), partial; / --- / --- / --- / ---

Gene Name	Fold Change	p-value	SEQUENCE	Array Description	Blast/Database Description
RIGG11523	0.00667	0.00493	GAACAATGTAGACCAAGTAATGCTG CAAAGTGCTGTCCCCGTTATCACCG CAGTCATTCAAGAGCACCTC		ENSGALT00000005036.1 / --- / Gallus gallus finished cDNA, clone ChEST727j13 / Finished cDNA, clone ChEST727j13 / --- / PREDICTED: Gallus gallus similar to Gene model 711, (NCBI) (LOC417154), mRNA. / PREDICTED: similar to Gene model 711, (NCBI); / Finished cDNA, clone ChEST727j13; / --- / Gallus gallus finished cDNA, clone ChEST727j13 / Finished cDNA, clone ChEST727j13
RIGG19369	0.00665	0.0238	TCATCACCTGATGAAGTTGCTTTAGT TGAAGGAATACAGAGGCTTGGTTAC ACCTATCTATGCCTGAAGGACAAT		ENSGALT00000027187.1 / --- / --- / --- / / PREDICTED: Gallus gallus similar to Potential phospholipid-transporting ATPase IH (ATPase class I type 11A) (ATPase IS) (LOC418749), mRNA. / PREDICTED: similar to Potential phospholipid-transporting ATPase IH (ATPase class I type 11A) (ATPase IS); / Finished cDNA, clone ChEST875n2; / --- / 603862207F1 CSEQCHN54 Gallus gallus cDNA clone ChEST875n2 5', mRNA sequence / Finished cDNA, clone ChEST875n2
RIGG10413	0.00646	0.0047	CACAGATTGGTGCAAACTCCCTGAT GACTATCGATGTTCATTCCGTATTCA TAATGGACAGAAGCTAGTT		ENSGALT00000001895.1 / similar to GB|AAF03702.1|6103643|AF176702 F-box protein FBX3 {Homo sapiens} (exp=-1; wgp=0; cg=0), partial (71%) / --- / --- / --- / PREDICTED: Gallus gallus similar to F-box only protein 3 isoform 1; F-box protein FBX3; F-box only protein 3 (LOC426965), partial mRNA.PREDICTED: Gallus gallus similar to F-box only protein 3 isoform 2; F-box protein FBA (LOC429495), partial mRNA. / PREDICTED: similar to F-box only protein 3 isoform 1; F-box protein FBX3; F-box only protein 3, partial;PREDICTED: similar to F-box only protein 3 isoform 2; F-box protein FBA, partial; / --- / --- / --- / ---
RIGG19396	0.00595	0.00116	CCAAGGGCATTTGTGTATTTATCTG GTTTCTTGTATTCAGCCAAACTTTCC CATTGCTTATACAGCCCATG		ENSGALT00000027271.1 / similar to UP|Q53F99_HUMAN (Q53F99) EBV-induced G protein-coupled receptor 2 variant (Fragment), partial (91%) / pgp1n.pk014.a22 Normalized Chicken Pituitary/Hypothalamus/Pineal Library Gallus gallus cDNA clone pgp1n.pk014.a22 5' similar to gi|4826706 ref|NP_004942.1| Epstein-Barr virus induced gene 2 (lymphocyte-specific G protein-coupled receptor) [Homo sapiens]> / Transcribed locus, weakly similar to XP_549098.2 PREDICTED: similar to G protein-coupled receptor 23 [Canis familiaris] / hypothetical protein / --- / --- / --- / Epstein-Barr virus induced gene 2 (lymphocyte-specific G protein-coupled receptor); / pgp1n.pk014.a22 Normalized Chicken Pituitary/Hypothalamus/Pineal Library Gallus gallus cDNA clone pgp1n.pk014.a22 5' similar to gi|4826706 ref|NP_004942.1| Epstein-Barr virus induced gene 2 (lymphocyte-specific G protein-coupled receptor) [Homo sapiens]> / Transcribed locus, weakly similar to XP_549098.2 PREDICTED: similar to G protein-coupled receptor 23 [Canis familiaris]
RIGG18643	0.00545	0.000331	GTTCACACTTGTAATTTCAGCTGTAT CTGTGCATCTAACACATTTGGTCTG CCCTTGGAGGAAATTCATG		ENSGALT00000025181.1 / --- / --- / --- / --- / --- / --- / --- / --- / --- / ---

Gene Name	Fold Change	p-value	SEQUENCE	Array Description	Blast/Database Description
RIGG13729	0.00537	0.0183	GGCAACTGAAGCTTAAAGGCCTTAT AACTGGCATTGGCTATGAGGTTATG CTTTATGGTTTTGCCAAGGG	Tenascin (Fragment). [Source:SPTREMBL;Acc:Q90996]	Tenascin (Fragment). [Source:SPTREMBL / GB|X73833.1|CAA52055.1 tenascin [Gallus gallus] / Chicken alternatively spliced tenascin 190, 200 and 230 kd variants mRNA, complete cds / Cytotactin 200kD / TNC: Tenascin precursor / --- / --- / --- / --- / --- / ---
RIGG17161	0.00519	0.0191	GCACTGTGAAAAGGAAACTAGATGG GGAGAAAGATTATGTCTTCGATAAG AGACTAAGATACAGCGTGCGTC		ENSGALT00000021177.1 / GB|BX934811.2|BX934811.2 Gallus gallus finished cDNA, clone ChEST561a19 / Gallus gallus finished cDNA, clone ChEST561a19 / Finished cDNA, clone ChEST561a19 / similar to chromodomain Y-like protein 2 / PREDICTED: Gallus gallus similar to chromodomain Y-like protein 2 (LOC425886), mRNA. / PREDICTED: similar to chromodomain Y-like protein 2; / Finished cDNA, clone ChEST561a19; / --- / Gallus gallus finished cDNA, clone ChEST561a19 / Finished cDNA, clone ChEST561a19
RIGG12684	0.00517	0.000883	ACCGAAGAGTGTCACACAATGTCAT ATCACACAGTAATTCTTCACGTGGA TGAATTCCCCAGAAGAAAGCCAATG		ENSGALT00000008400.1 / Gallus gallus finished cDNA, clone ChEST130e21 / Gallus gallus finished cDNA, clone ChEST130e21 / Finished cDNA, clone ChEST130e21 / --- / --- / --- / --- / --- / Gallus gallus finished cDNA, clone ChEST130e21 / Finished cDNA, clone ChEST130e21
RIGG12227	0.00396	0.000704	GTCTGTCTTCAGCTATCAGGTTGCA TCTACATTAAAGCAAGTGAAACACG ATCAACAAGTTGCGCGGATG		ENSGALT00000007029.1 / homologue to UP|CJ104_HUMAN (Q96DE5) Protein C10orf104, partial (88%) / 603368918F1 CSEQRBN19 Gallus gallus cDNA clone ChEST272d6 5', mRNA sequence / Transcribed locus, strongly similar to NP_079790.1 hypothetical protein LOC52717 [Mus musculus] / --- / PREDICTED: Gallus gallus similar to hypothetical protein D10Ertd641e (LOC423708), mRNA. / PREDICTED: similar to hypothetical protein D10Ertd641e; / --- / --- / --- / ---
RIGG13771	0.00328	0.00127	CTGGATTCAGCAGACTCCTTCCAAA GCTTTTACAGCCCTCACAAGGCGCA GATGAAGAACCCGAT	syntaxin 1-binding protein [Gallus gallus]. [Source:RefSeq;Acc:NM_206976]	syntaxin 1-binding protein [Gallus gallus]. [Source:RefSeq / UP|Q6R748_CHICK (Q6R748) Syntaxin 1-binding protein, complete / Gallus gallus syntaxin 1-binding protein mRNA, complete cds / Syntaxin binding protein 1 (STXBP1), mRNA / Syntaxin 1-binding protein / --- / --- / --- / --- / Gallus gallus syntaxin 1-binding protein mRNA, complete cds / Syntaxin binding protein 1 (STXBP1), mRNA
RIGG18623	0.00235	0.0457	GTTCTGGATGGGAGAGTTCATATTC GTGACTGGAGAAAAGAGAAACCATC AAGGAAAACGAAGCCTGAAG		ENSGALT00000025145.1 / --- / --- / --- / --- / PREDICTED: Gallus gallus similar to RIKEN cDNA 2310079F23 (LOC422871), mRNA. / PREDICTED: similar to RIKEN cDNA 2310079F23; / --- / --- / 603862029F1 CSEQCHN54 Gallus gallus cDNA clone ChEST875i7 5', mRNA sequence / Transcribed locus, weakly similar to NP_084484.1 hypothetical protein LOC78890 [Mus musculus]
RIGG11851	0.002	0.00478	GTAACTACGTCGCTGTTAAAGAAAG AGGCTCTGTGTCCTCGTTTGCGTAC AAAACGGGCCAGAAGCTAGC		ENSGALT00000005969.1 / --- / --- / --- / --- / --- / --- / --- / --- / --- / ---

Gene Name	Fold Change	p-value	SEQUENCE	Array Description	Blast/Database Description
RIGG16808	0.00196	0.0113	AAAATAGCTTTCCTCTGCTCATTCTGTACATTGCTCTTGTAAATGCTGATGATCTGTGAAGACCTGCAGA		ENSGALT00000020185.1 / GB|CR390163.1|CR390163.1 Gallus gallus finished cDNA, clone ChEST730k14 / gPGC_EST01169 Embryonic gonadal PGC cDNA Library Gallus gallus cDNA 5', mRNA sequence / Finished cDNA, clone ChEST730k14 / --- / --- / --- / --- / --- / 603491533F1 CSEQCHN63 Gallus gallus cDNA clone ChEST394b10 5', mRNA sequence / Finished cDNA, clone ChEST466p18
